# TIMP1 shapes an immunosuppressive microenvironment by regulating anoikis to promote the progression of clear cell renal cell carcinoma

**DOI:** 10.18632/aging.205005

**Published:** 2023-09-08

**Authors:** Qiang Li, Kai Wei, Xi Zhang, Yang Lv, Miao Li, Chenchao Zhou, Shifeng Su, Daorong Hou, Jianquan Hou

**Affiliations:** 1 Department of Urology, The First Affiliated Hospital of Soochow University, Suzhou 215006, China; 2 Department of Urology, The Affiliated Jiangsu Shengze Hospital of Nanjing Medical University, Suzhou 215228, China; 3 The State Key Lab of Reproductive, Department of Urology, The First Affiliated Hospital of Nanjing Medical University, Nanjing 210029, China; 4 Department of Urology, The Second Affiliated Hospital of Soochow University, Suzhou 215004, China; 5 Department of Urology, Dushu Lake Hospital Affiliated to Soochow University, Suzhou 215000, China; 6 Key Laboratory of Model Animal Research, Animal Core Facility of Nanjing Medical University, Nanjing Medical University, Nanjing 211166, China

**Keywords:** anoikis, tumor microenvironment, anoikis index, ccRCC, TIMP1

## Abstract

Background: The association between ccRCC and Anoikis remains to be thoroughly investigated.

Methods: Anoikis-related clusters were identified using NMF. To identify prognostic anoikis-related genes (ARGs) and establish an optimal prognostic model, univariate Cox and LASSO regression were employed. The E-MTAB-1980 cohort was utilized for external validation. Multiple algorithms were used to evaluate the immune properties of the model. GO, KEGG and GSVA analyses were employed to analyze biological pathway functions. qRT-PCR was employed to measure RNA levels of specific genes. Cell Counting Kit-8, wound healing, and Transwell chamber assays were performed to determine changes in the proliferative and metastatic abilities of A498 and 786-O cells.

Results: Based on the expression of 21 prognostic ARGs, we constructed anoikis-related clusters with different prognostic and immune characteristics. The cluster A1 showed a worse prognosis, higher infiltration of immunosuppressive cells and enrichment of several oncogenic pathways. We also calculated the Anoikis Index (AI). Patients in high AI group had a worse prognosis, higher infiltration of immunosuppressive cells and higher expression of immunosuppressive checkpoints. TIMP1 exerted a tumor-promoting role in ccRCC and was significantly associated with immunosuppressive cells and checkpoints. The downregulation of TIMP1 negatively regulated ccRCC cell proliferation and metastasis.

Conclusions: ARGs played crucial roles in tumorigenesis and progression and were positively associated with a poor prognosis. AI had great accuracy in predicting the prognosis and immune characteristics of ccRCC patients. TIMP1 was significantly associated with clinicopathological variables and the immunosuppressive microenvironment, which could be exploited to design novel immunotherapies for ccRCC patients.

## INTRODUCTION

Renal cell carcinoma (RCC) is a common malignant tumour of the urinary system that originates in the renal tubular epithelium [1, 2]. There are approximately 295,000 newly diagnosed cases of RCC and around 134,000 deaths worldwide annually, accounting for about 2% of all cancer-related deaths globally [3]. Clear cell renal cell carcinoma (ccRCC) is the predominant subtype of RCC, comprising approximately 70-80% of all RCC cases [4, 5]. The majority of ccRCC cases do not respond well to radiotherapy or chemotherapy. Therefore, early resection is considered the primary treatment for ccRCC patients [6]. However, even after tumour resection, recurrence or metastasis still occurs in nearly 30% of patients with localized ccRCC [7, 8]. The clinical outcomes for advanced ccRCC remain unsatisfactory despite significant advances in early detection, diagnosis and surgery [9]. Therefore, it is imperative to discover useful biomarkers for early diagnosis to improve the treatment of ccRCC patients.

The development and homeostasis of multicellular organisms rely largely on programmed cell death (PCD), which allows organisms to eliminate cells that could easily transform into tumors or be hijacked by pathogenic bacteria for replication [10, 11]. The known types of PCD include autophagy-dependent cell death, apoptosis, necrosis, pyrodegeneration, NETosis, and more [12]. Anoikis refers to the death of normal adherent cells that have been in a suspended state for an extended period due to "homelessness" [13]. Anoikis is a specific type of PCD that occurs when cells separate from the extracellular matrix, initiating a "suicidal" behavior that plays an important role in body development, tissue homeostasis, disease development, and tumor metastasis [14, 15]. The ability of transformed cells to survive under "isolated" growth conditions is a common feature of tumour development and growth [16]. This acquired ability, known as anoikis resistance, is associated with the loss of cellular homeostasis, cancer growth and metastasis [17]. Cancer cells with anti-anoikis properties can spread to distant tissues or organs through the peripheral circulation, causing cancer metastasis [18]. Anoikis resistance occurs when detached cells circumvent the death signaling pathway, enabling their survival through biochemical and molecular alterations within the cell milieu. These changes are characteristic of invasiveness, metastasis, therapy resistance, and relapse of cancer cells [19]. Cancer cells metastasize through successive processes of detachment from one another or the ECM, migration to distal points, promotion of reattachment, and proliferation at the new site [20]. Cancer cells employ various mechanisms to evade anoikis, promoting their invasiveness and metastasis. Cellular acidosis and changes in reactive oxygen species (ROS) generation have a significant impact on promoting anoikis resistance [21]. These changes activate oncogenic signals that induce pro-survival pathways, leading to stemness, proliferation, and invasion [22, 23]. While nutrient uptake is necessary for cellular growth, abnormal metabolism in cancer patients can lead to anoikis resistance and promote therapy resistance. Studying the molecular mechanisms that control anti-anoikis can provide insights into effective therapies for human malignancies.

TIMP1 is a member of the matrix metalloproteinase (MMP) inhibitors family, which includes four identified members, TIMP1, TIMP2, TIMP3 and TIMP4 [24]. In addition to inhibiting MMP-mediated degradation of the extracellular matrix, TIMP1 also interacts with other proteins and plays a critical role in regulating biological processes such as cell growth, apoptosis, and differentiation [25]. Studies have shown that overexpression of TIMP1 may significantly increase genes involved in proliferation, apoptosis, and signal transduction variants [26, 27]. TIMP1 may also bind to the CD63/integrin β1 complex, producing an anti-apoptotic effect [28]. Numerous clinical studies have demonstrated that abnormal expression of TIMP1 is associated with an adverse prognosis in various tumours. However, the exact function and underlying mechanism of TIMP1 in ccRCC remain to be further elucidated.

This present study systematically investigated the association of ARGs with immune infiltrating cells and clinicopathological variables in ccRCC. Additionally, an anoikis-related prognostic model was constructed to effectively identify the survival risk and predict the survival rate of ccRCC patients. The mechanism of TIMP1 in regulating anoikis in the tumor microenvironment may provide insights for the design of new immunotherapies for ccRCC.

## MATERIALS AND METHODS

### Data acquisition and study design

Gene expression profiles and clinical data of ccRCC patients were obtained from The Cancer Genome Atlas (TCGA, https://www.cancer.gov/tcga). A total of 434 ARGs were selected from Genecards according to the criterion of a correlation score > 0.4 (Supplementary Table 1). The E-MTAB-1980 cohort was used as an external validation cohort. It was downloaded from the ArrayExpress database (https://www.ebi.ac.uk/arrayexpress/). Additionally, gene expression data from GSE73731 [29], GSE66272 [30] and GSE67501 [31] downloaded from the Gene Expression Omnibus (GEO; http://www.ncbi.nlm.nih.gov/geo/) database, were used to validate the clinicopathological characteristics of the genes.

### Identification of prognosis related ARGs

Differentially expressed ARGs in ccRCC and normal kidney tissue were screened using the R package limma and Wilcoxon tests [32]. The screening criteria were |logFC|>1.5 and adjusted P < 0.05. Univariate Cox regression analysis was performed on the differentially expressed ARGs to further identify ARGs associated with prognosis, using a screening criterion of *p*-value < 0.05.

### Cluster analysis and bioinformatics analysis

Hierarchical clustering of ccRCC samples based on prognosis-related ARGs expression profiles was performed using the R package non-negative matrix factorization (NMF) [33]. Functional enrichment analysis was conducted to confirm the potential functions of the different clusters. The screening criteria for differential genes between clusters were P<0.001 and |logFC|>2. Gene Ontology (GO) and Kyoto Encyclopedia of Genes and Genomes (KEGG) enrichment analysis were performed to annotate functional genes and analyze gene function and related advanced genome function information. Gene set variation analysis (GSVA) enrichment analysis was conducted using the “GSVA” R package to explore the role of ARGs in biological pathways. The gene set “c2.cp.kegg.v7.4.symbols” from the MSigDB database was used for GSVA analysis.

### Construction and validation of the Anoikis index (AI)

Prognostic ARGs were identified and further screened using the least absolute shrinkage and selection operator (LASSO) Cox regression analysis with the R package glmnet [34]. The AI formula was obtained by the linear combination of gene expressions weighted regression coefficients. The algorithm was as follows: AI=Coef A * Gene A expression + Coef B * Gene B expression +Coef C * Gene C expression+......Coef N * Gene N expression, with Coef referring to coefficient calculated by LASSO and gene expression referring to expression of ARGs. The ccRCC patients with survival data were divided into Low- and High- AI groups according to the median AI. Time-dependent receiver operating characteristic (ROC) curve was performed and the area under the ROC curve (AUC) was performed using the survivalROC package. Univariate and multivariate Cox regression analyses were used to evaluate the independence of AI and various clinicopathological features.

### Immune microenvironment assessment and mutation analysis

Estimation of Stromal and Immune cells in Malignant Tumor tissues using Expression data (ESTIMATE) analysis was employed to quantify the immune scores, stromal scores, estimate scores, and tumor purity in the tumor microenvironment (TME) of each ccRCC sample. The relative abundance of each immune cell in the TME was quantitatively analysed using different algorithms (TIMER, CIBERSORT, QUANTISEQ, MCPCOUNTER, XCELL, EPIC and ssGSEA). Mutation data for ccRCC patients were downloaded from the TCGA database, and the Maftools package was used to illustrate the respective mutational profiling of the two risk levels by a waterfall plot [35]. Asterisks represented statistical p-values (*p < 0.05, **p < 0.01, ***p < 0.001).

### Identification of immune and clinical characteristics of TIMP1

The “ggplot2” package was used to analyze the differential expression of ARGs in different pathological grades and histological stages. Genes significantly associated with clinical features and playing key roles in the pathway were screened based on the correlation of ARGs expression with clinicopathological features. All ccRCC samples were divided into high and low TIMP1 expression groups according to the median expression level of TIMP1. The correlation analysis of TIMP1 expression with clinicopathological characteristics was validated in the GSE40435, GSE53757, and GSE73731 datasets. The Wilcoxon test was employed to verify differences in immune cells and checkpoints between the high and low TIMP1 expression groups. The “ggplot2” package and Spearman analyses were used to analyze the association of the TIMP1 with immune cells and checkpoints.

### Western blot assay

Total proteins were extracted from the RCC cell lines, and a Western blot assay was performed after the detection of protein concentration. 20μg of samples were separated on a 10% SDS-PAGE gel, then transferred to a PVDF membrane and blocked for 1 hour at room temperature. The membranes were incubated with primary antibodies (TIMP1 concentration, 0.5 μg/mL; GAPDH dilution rate, 1:500; Abcam) at 4° C overnight. The next day, the membranes were incubated with the secondary antibody (Abcam; dilution rate, 1:2000) at 24° C for 1 hour. Signals of targeted proteins were detected using an enhanced chemiluminescence detection system.

### Cell culture and cell transfection

Two human ccRCC cell lines (A498, 786-O) were obtained from the cell bank of the Chinese Academy of Sciences (Shanghai, China). All cells were cultured in RPMI 1640 medium (Thermo Fisher Scientific, Inc.) supplemented with 10% fetal bovine serum (FBS; Thermo Fisher Scientific, Inc.) at a constant temperature of 37° C in a humidified atmosphere containing 5% CO2.

Lentiviral shRNA plasmids targeting TIMP1 along with the nonspecific control shRNA were obtained from Dharmacon (Shanghai, China). Transfection of plasmid and shRNA was performed using Lipo3000 following the manufacturer's instructions.

### Cell counting kit-8 (CCK8) assay

A498 and 786-O cells after different interventions were incubated in 96-well plates (2x10^3) with 200 μL culture medium and conditioned in 37° C with 5% CO2. On days 1, 2, 3, 4 and 5, 20μL of CCK-8 solution was added into each well, and incubation was performed for 2 hours. Absorbance was measured at an optical density of 450 nm using a Microplate reader (Bio-Rad Laboratories, Inc.).

### Transwell assay

A498 and 786-O cells (with an incubation density of 2x10^5 cells) were incubated in the upper chambers (Corning). For the invasion assay, the upper chambers were pre-coated with Matrigel (BD Biosciences). Culture medium without and with 10% FBS was added to the upper and lower chambers, respectively. After 12 hours, non-migrated cells were wiped out, while migrated or invaded CRC cells were fixed, stained, and counted using an inverted microscope.

### Wound-healing assay

Cell migration was assessed by performing a wound healing assay. A498 and 786-O cells were transfected with TIMP1 were seeded at a density of approximately 2x10^6 cells in 6-well plates and cultured for 24 hours. Then, a yellow plastic pipette tip was used to create a wound by scraping the cells. Cell migration was monitored under a Nicon Eclipse microscope and photographed at 100×.

### Statistical analysis

All analyses were performed by using R 4.1.0. All statistical tests were two-tailed, and a P-value <0.05 was considered statistically significant unless otherwise noted. To analyse the association between ARGs and overall survival (OS) in ccRCC patients, Kaplan-Meier (KM) curves and the log-rank test were used. Univariate and multivariate Cox regression analyses were utilized to confirm independent impact factors associated with survival. The Benjamini-Hochberg (BH) method was used to adjust all adjusted P-values or false rate discovery (FDR). An adjusted P-value < 0.05 was considered statistically significant. Abbreviations and corresponding words and phrases used in this article (Supplementary Table 5).

### Data availability statement

All data used in this work can be acquired from (TCGA, (https://www.portal.gdc.cancer.gov/), GEO (https://www.ncbi.nlm.nih.gov/geo/).

## RESULTS

### Establishment and biological characteristics of anoikis clusters

**Figure 1:**
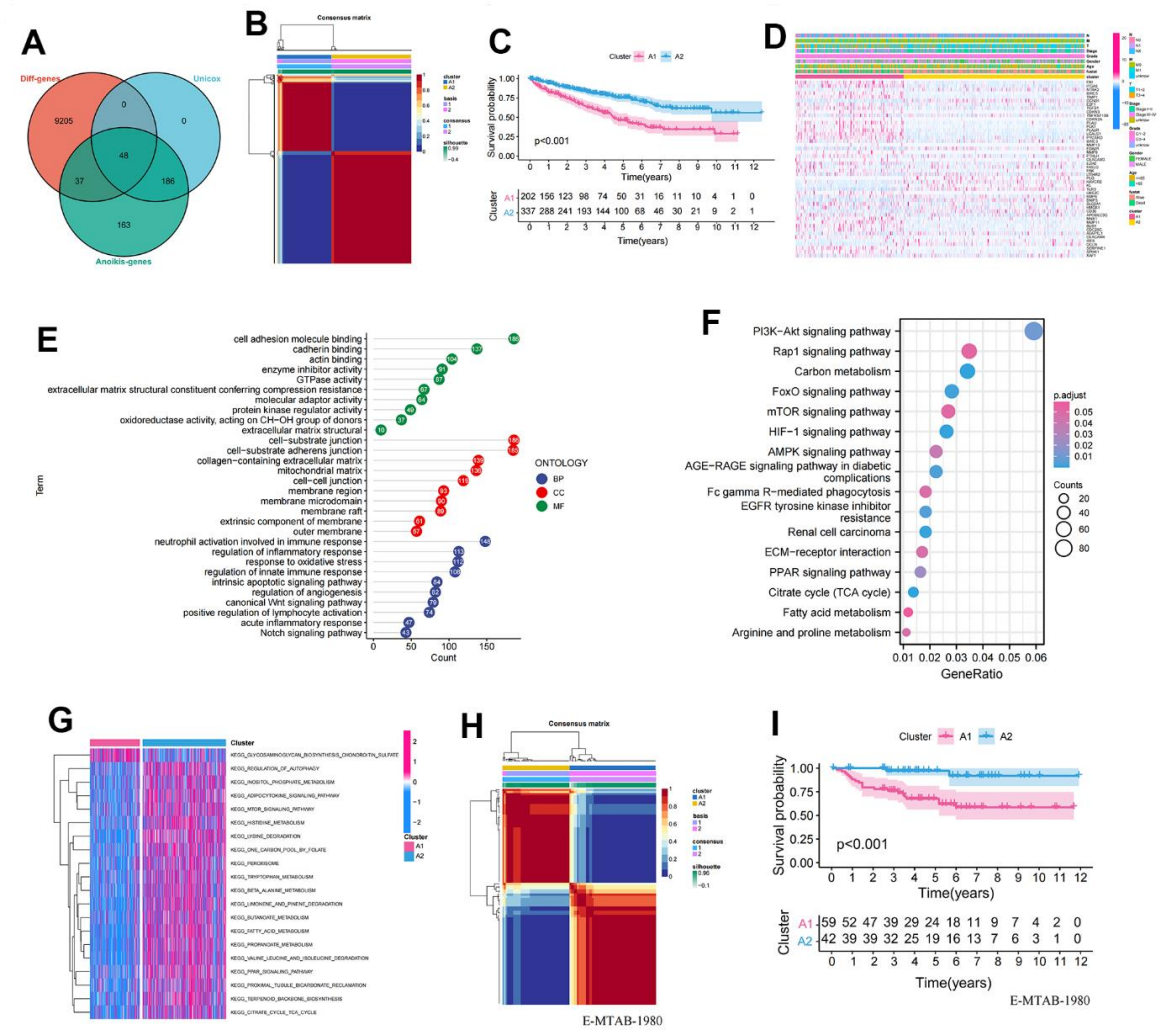
**Establishment and validation of anoikis-related clusters.** (**A**) Screening of 48 ARGs associated with prognosis and differentially expressed; (**B**) Heatmap plot indicating the consensus matrix of NMF clustering results utilizing the gene expression profile in TCGA KIRC cohort, colored by two ccRCC clusters; (**C**) KM survival curves revealing the prognosis difference of the two clusters (A1, A2); (**D**) The distribution of anoikis-related genes expression profile and clinicopathological characteristics in A1 and A2 clusters; (**E**) The results of GO biological process enrichment of differentially expressed genes; (**F**) The results of KEGG pathways analysis of differentially expressed genes; (**G**) Results of GSVA enrichment analysis between clusters; (**H**) Heatmap plot indicating the consensus matrix of NMF clustering results utilizing the gene expression profile in the E-MTAB-1980 cohort, colored by two ccRCC clusters; (**I**) KM survival curves revealing the prognosis difference of the two clusters (A1, A2).

In total, we included 434 ARGs, of which 85 genes exhibited differentially expression in ccRCC and paracancerous tissues, and 234 genes showed significant univariate associations (Supplementary Tables 1–3). Finally, we identified 48 differentially expressed ARGs with prognostic implications (Figure 1A). Next, we performed hierarchically clustering of ccRCC based on the expression profiles of these 48 prognosis-related ARGs, resulting in the division of samples into A1 and A2 clusters (Figure 1B). KM analysis demonstrated a significant difference in survival between the two clusters, with A1 showing a poor prognosis (Figure 1C). The heatmap displayed the distribution of gene expression and clinicopathological characteristics across the anoikis clusters (Figure 1D). To further explore the biological function differences between the two clusters, we identified 3,212 differentially expressed genes (P<0.001 and |logFC|>2) between them. These genes were subjected to GO and KEGG enrichment analyses. In the BP group, the genes were predominantly enriched in multiple immune regulatory pathways and regulation of inflammatory response, among others. In the CC group, the genes were mainly concentrated in the cell-substrate junction, extracellular matrix, and membrane region. In the MF group, the genes showed significant enrichment in the cell adhesion molecule binding, actin binding, and extracellular matrix structural (Figure 1E). The KEGG analysis indicated a concentration of genes in multiple cancer-promoting pathways, including the PI3K-Akt signaling pathway, mTOR signaling pathway, and AMPK signaling pathway (Figure 1F). Furthermore, GSVA analysis revealed a significant enrichment of metabolic pathways such as amino acid metabolism and fat metabolism in the A2 cluster (Figure 1G). To further validate the stability and accuracy of the 48 prognosis-related ARGs, we divided patients in the E-MTAB-1980 dataset into A1 and A2 clusters based on gene expression profiles (Figure 1H). Significant differences in survival were observed between the A1 and A2 clusters (Figure 1I).

### Identification of immunological characteristics of anoikis clusters

**Figure 2:**
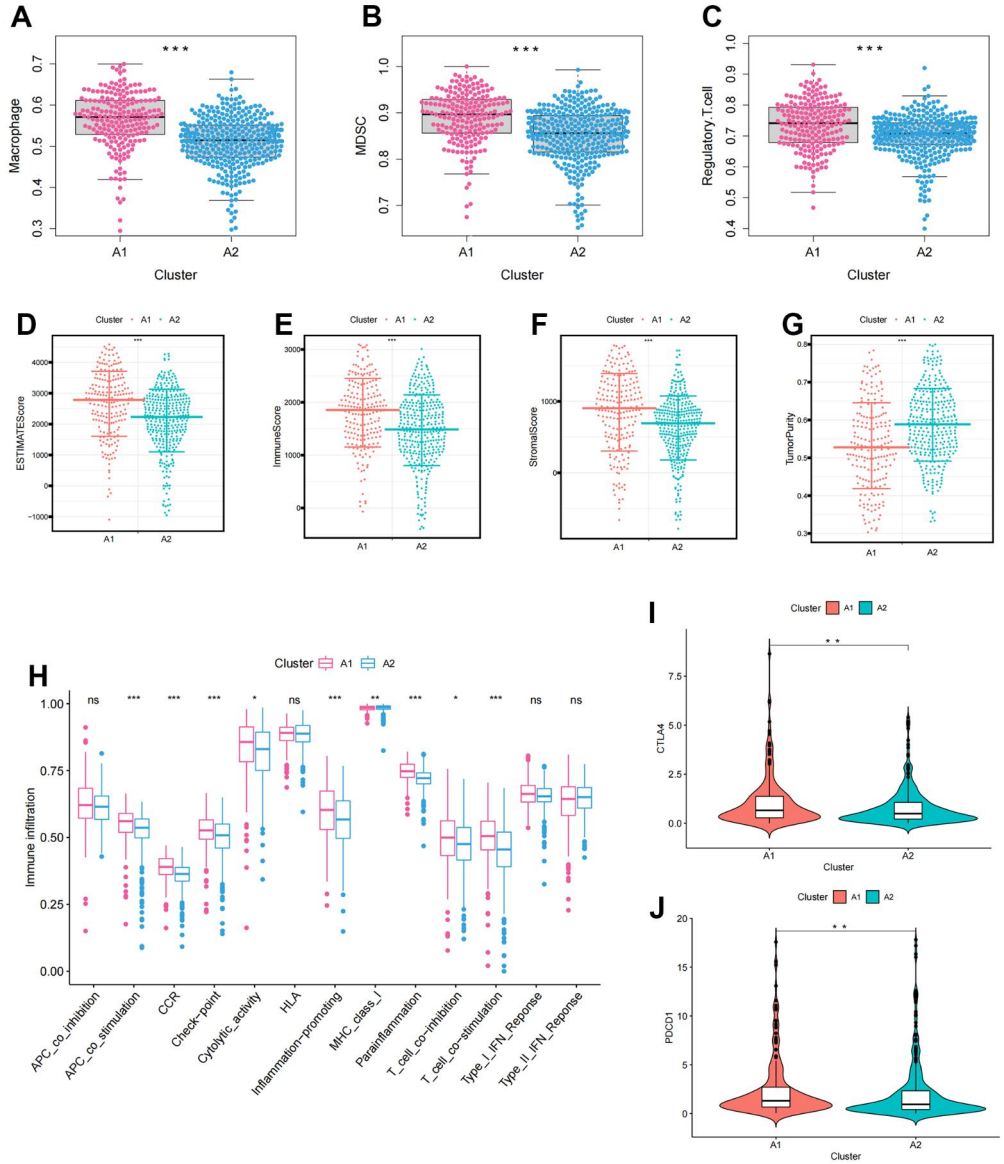
**The immune infiltration characteristics in anoikis-related clusters.** (**A**–**C**) The differential expression of immunosuppressive cells between clusters (**A**) Macrophage; (**B**) MDSC; (**C**) Regulatory.T.cell; (**D**–**G**) The differences in tumor microenvironment characteristics between clusters (**D**) ESTIMATEScore; (**E**) ImmuneScore; (**F**) StromalScore; (**G**) TumorPurity; (**H**) Boxplots showing the differences in immune function between clusters; (**I**, **J**) Differences in immune checkpoint expression between clusters (**I**) CTLA4; (**J**) PDCD1. The asterisks represented the statistical *p*-value (*p<0.05, **p<0.01, ***p<0.001).

The ssGSEA score was used to quantify the activities or abundances of immune signatures and immunosuppressive cells in the ccRCC samples. The results indicated that immunosuppressive cells (Macrophage, MDSC, and Regulatory.T.cell) were significantly overexpressed in the A1 cluster compared to the A2 cluster, consistent with the survival disadvantage of A1 cluster (Figure 2A–2C). Furthermore, we evaluated the differences in TME characteristics between A1 and A2 clusters. ESTIMATEScore, ImmuneScore, and StromalScore showed significant differences between A1 and A2 clusters and were highly expressed in A1 cluster (Figure 2D–2F). In contrast, tumourPurity had low expression in cluster A1 (Figure 2G). Additionally, APC co-stimulation, Checkpoint, Inflammation-promoting, and T cell co-stimulation were activated in the A1 cluster (Figure 2H). Moreover, immune checkpoints (CTLA4 and PDCD1) were significantly overexpressed in the A1 cluster (Figure 2I, 2J).

### Construction and validation of the Anoikis index (AI)

**Figure 3:**
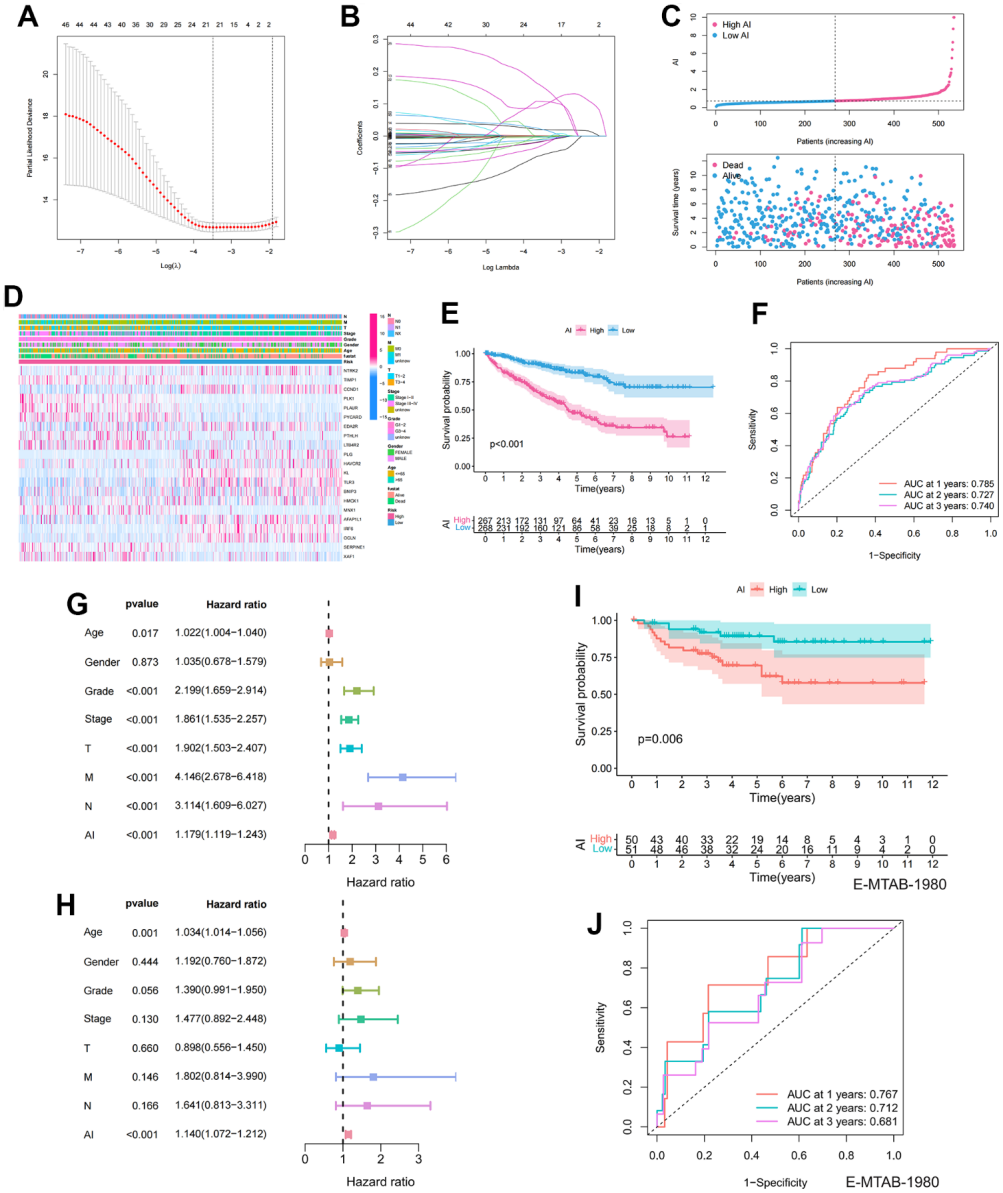
**Establishment and prognostic features of Anoikis index.** (**A**, **B**) Lasso analysis of prognostic ARGs with minimum lambda value; (**C**) The risk curve of each sample reordered by AI and the scatter plot of the sample survival overview. The blue and pink dots represent survival and death, respectively; (**D**) The distribution of anoikis-related genes expression profile and clinicopathological characteristics in AI; (**E**) Overall survival curve showing the prognostic difference between high and low AI group; (**F**) ROC curves about AI in 1,2,3 years; (**G**, **H**) The univariate and multivariate Cox regression analysis of AI, age, gender, grade, stage, TMN stage; (**I**) Differences in survival between high and low AI groups in the E-MTAB-1980 cohort; (**J**) ROC curves about AI in 1,2,3 years in the E-MTAB-1980 cohort.

Lasso regression analysis was applied to the 48 prognosis-related ARGs to improve robustness, resulting in the identification of 21 robust ARGs for constructing the Anoikis Index (AI) (Figure 3A, 3B). The Coefficients (Coef) for each modeled gene are presented in Supplementary Table 4. According to the median value of AI, the samples were divided into high and low AI groups. AI showed a negative correlation with patient survival based on the distribution of AI in ccRCC samples (Figure 3C). The heatmap revealed the distribution of gene expression and clinical characteristics (Figure 3D). The KM survival curve indicated that high AI was associated with a decreased chance of survival (Figure 3E). For 1, 2, and 3-year survival rates, the AUC predictive value of the AI was 0.785, 0.727, and 0.74, respectively (Figure 3F). To investigate the independent prognostic value of AI, univariate and multivariate Cox regression analysis was performed. In the univariate cox regression, age, grade, stage, TMN stage, and AI were linked to poor survival (Figure 3G). However, in the multivariate analysis, only age and AI were independent predictors of OS (Figure 3H). To further validate the accuracy of the model, the 101 patients in the E-MTAB-1980 were used as the validation set. The results indicated that AI was negatively correlated with patient survival in the E-MTAB-1980 dataset, consistent with the results of the TCGA analysis (Figure 3I). Furthermore, for 1-, 2- and 3-year survival rates, the AUC predictive value of the AI was 0.767, 0.712, and 0.681, respectively (Figure 3J).

### Identification of the tumor mutation burden (TMB) characteristics of the Anoikis index (AI)

**Figure 4:**
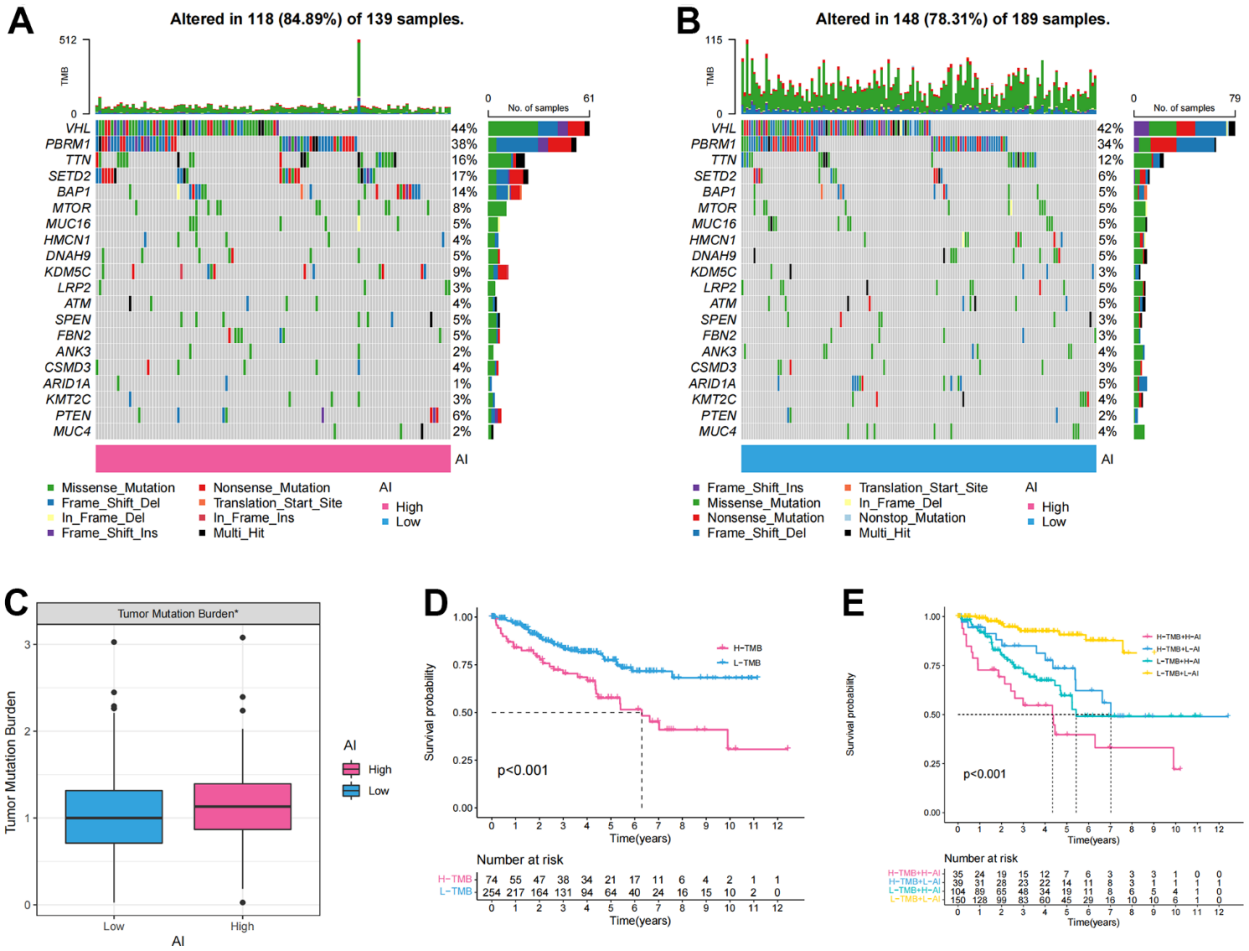
**The tumor mutation burden characteristics of Anoikis index.** (**A**, **B**) The waterfall chart showing the mutation frequency of the top 20 genes in the high and low AI groups; (**C**) Differences of the tumor mutation burden (TMB) between high and low AI groups; (**D**) KM survival curve showing the prognostic difference between high and low TMB groups; (**E**) KM survival curve showing OS of the combination of AI and TMB.

To determine the differences in cancer-related gene mutations between the High and Low AI groups, we first calculated the mutations in each group. The mutation rate in High AI group was 84.89% and that in Low AI group was 78.31%. Furthermore, in both high and low AI groups, the top five most mutated genes were: BAP1, SETD2, TTN, PBRM1, and VHL (Figure 4A, 4B). Additionally, significant differences in TMB were observed between the high and low AI groups (Figure 4C). The KM survival curve indicated that high TMB was associated with poor prognosis (Figure 4D). To further analyze the correlation between TMB and AI, combined mutation and AI survival analysis indicated that H-TMB+L-AI group had the worst prognosis, confirming the accuracy of the above results (Figure 4E).

### Identification of the immunological characteristics of the Anoikis index (AI)

**Figure 5:**
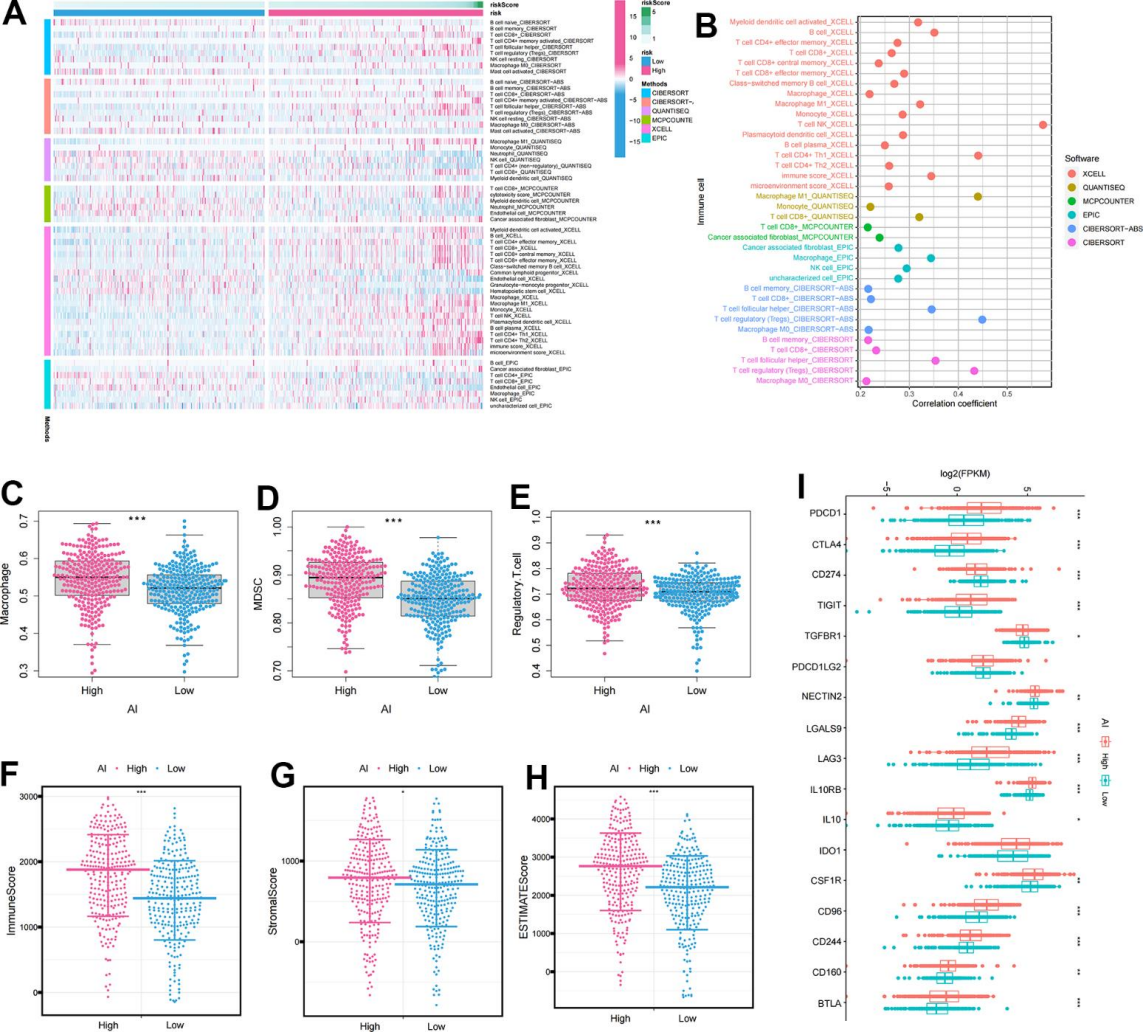
**The immune infiltration characteristics of Anoikis index.** (**A**) Distribution of immune cells in high and low AI groups under multiple algorithms; (**B**) The correlation between immune cells and AI under multiple algorithms; (**C**–**E**) The differential expression of immunosuppressive cells between high and low AI groups (**C**) Macrophage; (**D**) MDSC; (**E**) Regulatory.T.cell; (**F**–**H**) The differential expression of tumor microenvironment scores between high and low AI groups (**F**) ImmuneScore; (**G**) StromalScore; (**H**) ESTIMATEScore; (**I**) The differential expression of immune checkpoints between high and low AI groups. The asterisks represented the statistical *p*-value (*p<0.05, **p<0.01, ***p<0.001).

Figure 5A displayed a heatmap of immune responses based on CIBERSORT, QUANTISEQ, MCPCOUNTER, XCELL, CIBERSORT-ABS, TIMER, and EPIC algorithms, indicating a correlation between AI and immune cell infiltration in ccRCC. Specifically, AI exhibited a significant positive correlation with immune suppressive cells such as macrophages and Treg cells, according to various algorithms (Figure 5B). To further analyze the immune infiltration characteristics of AI, we investigated the differences in the expression of immune cell infiltration, immune checkpoints and TME between high and low AI groups. The results revealed significantly higher levels of immunosuppressive cells (Macrophage, MDSC, and Regulatory T cell) and TME characteristics (ESTIMATEScore, ImmuneScore, and StromalScore) in the High AI group (Figure 5C–5H). Similarly, the expression of immunosuppressive checkpoints was significantly higher in the High AI group than in the Low AI group (Figure 5I).

### Identification of the clinicopathological features and drug sensitivity of the Anoikis index (AI)

We first analyzed the proportions of various clinicopathological features between the High and Low AI groups. It was evident that the High AI group had a higher proportion of advanced clinicopathological stages compared to the Low AI group, and this difference was statistically significant (Supplementary Figure 1A–1E). Furthermore, we observed significant statistical differences in AI expression across diverse clinicopathological stages. Notably, AI expression increased as the clinicopathological stage advanced (Supplementary Figure 1F–1J). In-depth analysis of survival differences between the high and low AI groups in different clinicopathological stages indicated a poor prognosis for the high AI group, with AI showing a negative correlation with patient survival (Supplementary Figure 1K–1O). To assess the association between AI and ccRCC resistance, we screened nine major chemotherapeutic agents from the pRRophetic package. The Low AI group exhibited higher IC50 values for Bosutinib, Gefitinib, Nilotinib, Pazopanib, Sunitinib, Tipifarnib, Temsirolimus, Rapamycin, and Vorlistat, suggesting that these 9 drugs may be more suitable for patients with higher AI (Supplementary Figure 2).

### Identification and validation of the clinicopathological features of TIMP1

**Figure 6:**
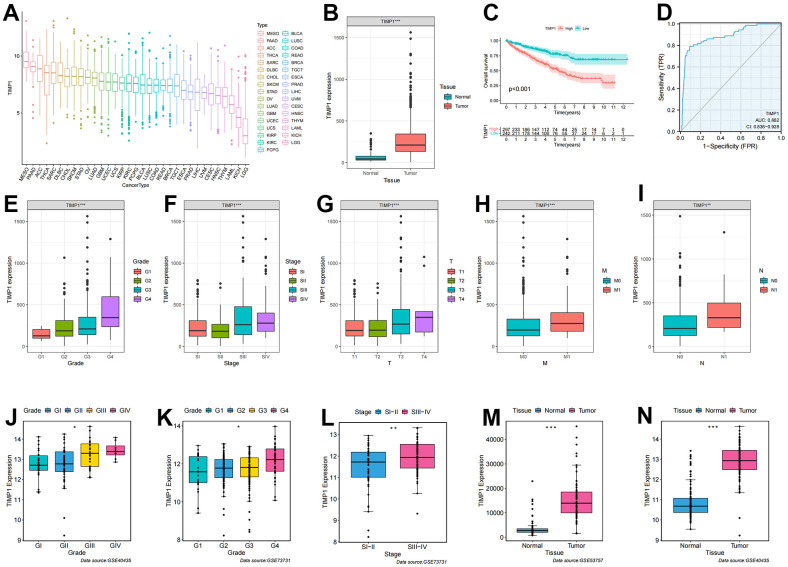
**Correlation of TIMP1 expression profile with clinicopathological characteristics in ccRCC.** (**A**) Expression profile of TIMP1 in 33 tumors; (**B**) Differential expression of TIMP1 in ccRCC and paracancerous tissues; (**C**) KM survival curves showing OS of TIMP1 in ccRCC; (**D**) ROC curves about TIMP1 in ccRCC; (**E**–**I**) Differences in TIMP1 expression profile among clinicopathological variables (**E**) Grade; (**F**) Stage; (**G**) T stage; (**H**) M stage; (**I**) N stage; (**J**–**N**) Differential expression of TIMP1 in different clinicopathological stages in the GEO validation datasets. (**J**, **N**) GSE40435; (**K**, **L**) GSE73731; (**M**) GSE53757.

To further analyze the key events leading to tumorigenesis and progression, we assessed the correlation between gene expression profiles and clinicopathological variables, revealing a significant association of TIMP1 with clinical features. Initially, we analyzed the expression level of TIMP1 in 33 tumors and observed a moderate expression level in KIRC (Figure 6A). Subsequently, we investigated the differential expression of TIMP1 between ccRCC and adjacent carcinoma tissues in TCGA database, finding a significant overexpression of TIMP1 in ccRCC (Figure 6B). The KM survival curve indicated a poor prognosis associated with TIMP1 (Figure 6C). Additionally, ROC curve analysis demonstrated a high prognostic value of TIMP1 in ccRCC patients (AUC=0.882) (Figure 6D). Further analysis involved examining the expression profile of TIMP1 across different clinicopathological stages. As depicted in Figure 6E–6I, the expression profile of TIMP1 exhibited a significant positively correlated with various clinical variables, including histological grade (P <0.001), pathological stage (P <0.001), T stage (P <0.001), M stage (P <0.001), and N stage (P <0.01). Furthermore, we investigated the expression profile of TIMP1 in various clinical variables across several GEO datasets, revealing its high expression in advanced clinicopathological stages and cancer tissues (Figure 6J–6N).

### Identification of the immunological characteristics of TIMP1

**Figure 7:**
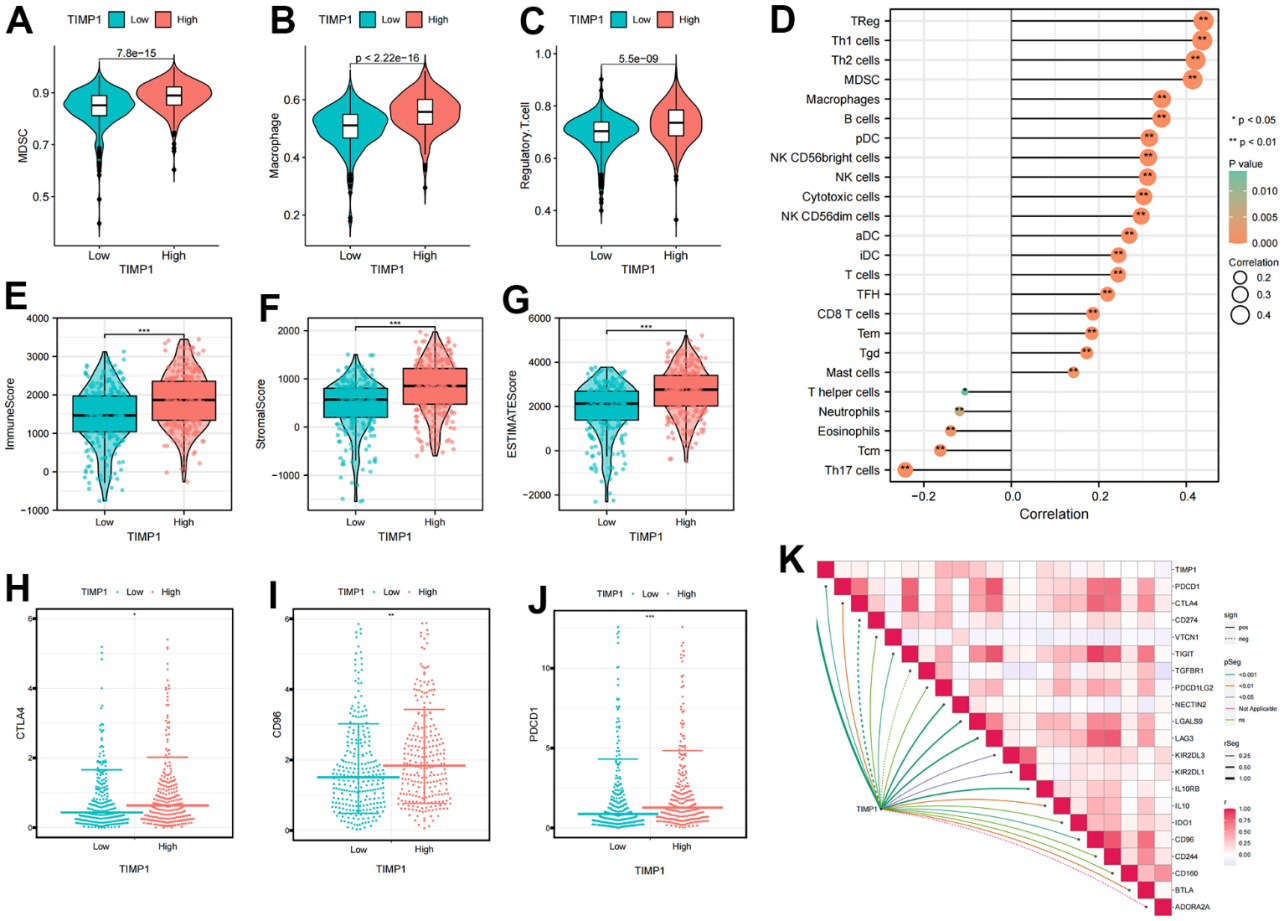
**Identification the immune infiltration characteristics of TIMP1 in ccRCC.** (**A**–**C**) The differential expression of immunosuppressive cells between high and low TIMP1 groups (**A**) MDSC; (**B**) Macrophage; (**C**) Regulatory.T.cell; (**D**) The correlation of TIMP1 expression profile with immune cells in ccRCC; (**E**–**G**) The differential expression of tumor microenvironment scores between high and low TIMP1 groups (**E**) ImmuneScore; (**F**) StromalScore; (**G**) ESTIMATEScore; (**H**–**J**) The differential expression of immune checkpoints between high and low TIMP1 groups (**H**) CTLA4; (**I**) CD96); (**J**) PDCD1; (**K**) The correlation of TIMP1 expression profile with immunosuppressive checkpoints in ccRCC.

We conducted further investigations into the correlation between TIMP1 expression profiles and immune cells as well as immune checkpoints. The ccRCC patients were categorized into high or low TIMP1 expression groups based on the median value of TIMP1 expression. It became apparent that the expression of immunosuppressive cells (such as Macrophage, MDSC, and Regulatory.T.cell) was higher in the high TIMP1 expression group compared to the low TIMP1 expression group (Figure 7A–7C). Subsequently, by analyzing the correlation between TIMP1 and immune cells, we observed a significant positive correlation between TIMP1 and immune suppressive cells, including Macrophage, MDSC, and Regulatory.T.cell (Figure 7D). Consequently, we proceeded to evaluate the immune microenvironmental characteristics of TIMP1 based on ImmuneScore and StromalScore in ccRCC tissues. The results demonstrated a significant positive correlation between TIMP1 and immune, stromal, and estimated scores (Figure 7E–7G). Additionally, we examined the correlation between TIMP1 expression profiles and immune-suppressive checkpoints. As depicted in the Figure 7H–7J, CTLA4, PDCD1, and CD96 were all expressed at higher levels in the high TIMP1 group. Furthermore, TIMP1 exhibited a significantly positively correlated with various immunosuppressive checkpoints (Figure 7K). Collectively, these findings indicate a close association between TIMP1 and the establishment of an immunosuppressive microenvironment.

### Validation of TIMP1 in tissues and cell lines

**Figure 8:**
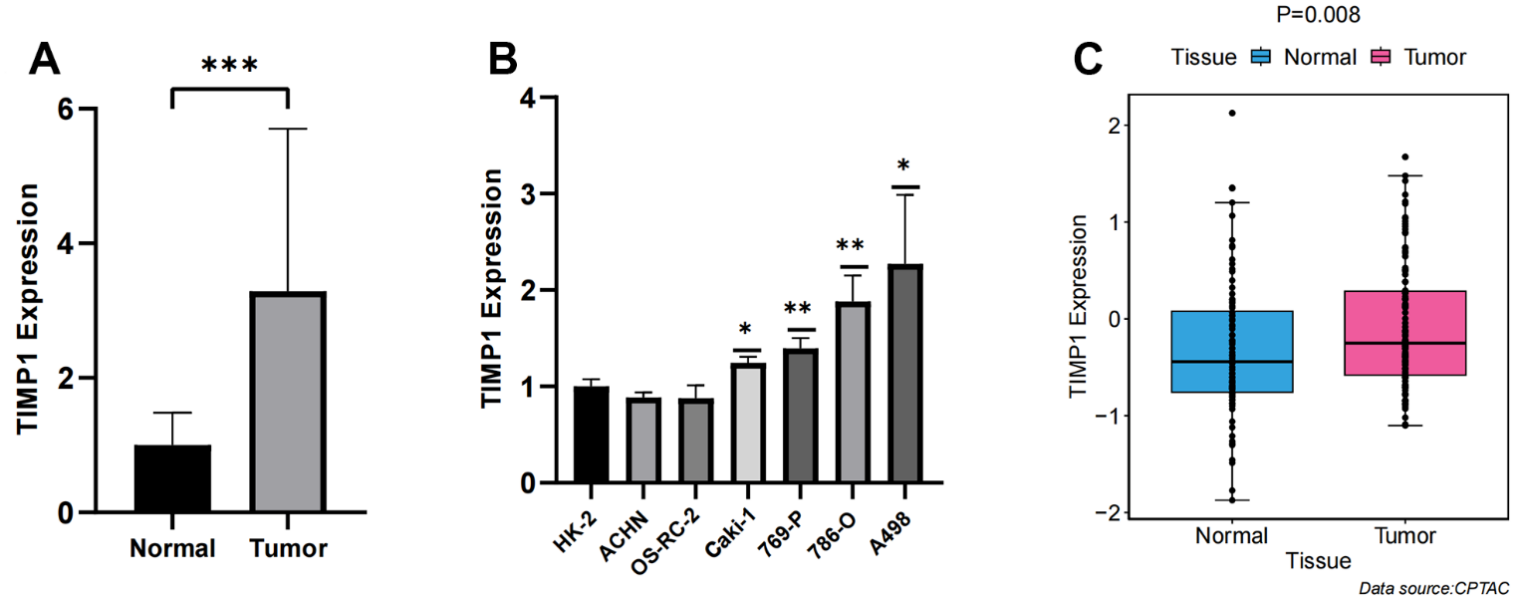
**Verification of TIMP1 expression differences between carcinoma and adjacent tissue.** (**A**) Bar plot for the relative expression of TIMP1 in ccRCC tissues and normal tissues; (**B**) Bar plot for the relative expression of TIMP1 in ccRCC and normal cell lines; (**C**) Difference of TIMP1 protein expression between ccRCC and adjacent tissues.

qRT-PCR was conducted on 18 pairs of ccRCC tissues and normal renal tissues, as well as 7 cell lines, including 6 tumor cell lines and 1 normal renal cell line. Significantly higher expression of TIMP1 was observed in tumor tissues compared to normal renal tissues (Figure 8A). Moreover, when compared to normal renal cell lines, the expression of TIMP1 was markedly elevated in tumour cell lines, with the highest expression observed in the A498 cell line (Figure 8B). These experimental results were in line with the predictions made using bioinformatics methods. Additionally, the CPTAC database provided explicit evidence that the protein expression levels of TIMP1 were significantly elevated in ccRCC tissues as compared to normal kidney tissues (Figure 8C).

### TIMP1-knockdown suppressed proliferation, migration and invasion in A498 and 786-O cells

**Figure 9:**
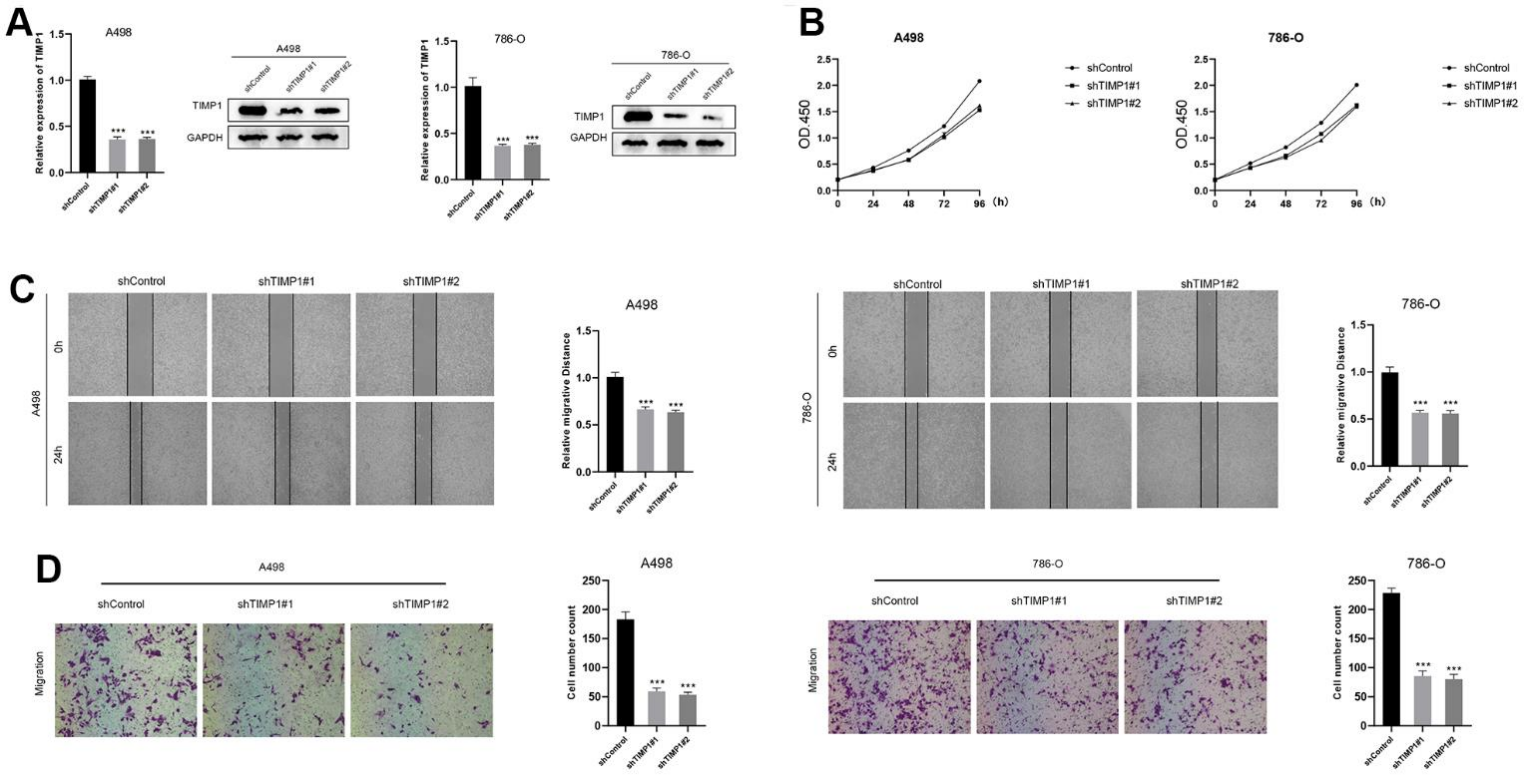
**Down-regulation of TIMP1 suppressed the progression of ccRCC *in vitro*.** (**A**) The expression of TIMP1 in A498 and 786-O cells was detected by RT-qPCR and Western blot; (**B**) TIMP1-knockdown suppressed ccRCC cell proliferation in A498 and 786-O cells; (**C**) Wound-healing tests demonstrated changes in ccRCC cell migration; (**D**) TIMP1-knockdown suppressed ccRCC cell metastasis in A498 and 786-O cells.

In the TIMP1-knockdown group, both mRNA and protein expression of TIMP1 were significantly down-regulated (Figure 9A). The CCK8 assay demonstrated a marked decrease in the proliferation of A498 and 786-O cells in the TIMP1-knockdown group (Figure 9B). Wound healing analysis indicated that the healing distance of A498 and 786-O cells in the TIMP1-knockdown group was lower than that in the control group after 24 hours (Figure 9C). Transwell experiments revealed a clear inhibition of migration in A498 and 786-O cells in the TIMP1-knockdown group (Figure 9D). Therefore, the expression of TIMP1 was found to be positively correlated with the proliferation, migration and invasion of ccRCC cells.

## DISCUSSION

Recent studies have revealed an alarming increase in the incidence and mortality rates of ccRCC, making it the most fatal renal malignancy in adults [36]. As the understanding of the underlying mechanisms of RCC occurrence and development has advanced, traditional histopathological features such as tumor size, stage, and grade may no longer suffice for accurate diagnosis and prognosis [7, 37]. Consequently, the identification of reliable prognostic biomarkers is crucial for enhancing prognosis prediction in ccRCC. Anoikis, a specific form of apoptosis triggered by cell detachment from the native extracellular matrix, has garnered significant attention in the scientific community due to its pivotal role in tumor angiogenesis and metastasis [38, 39]. Thus, to further explore the involvement of ARGs in tumor progression and prognosis, this study investigates prognosis-related ARGs in ccRCC and establishes a prognostic model.

Anoikis plays a crucial role in tumour progression, metastasis and spread of cancer cells, as it is associated with anchorage-independent growth and epithelial-mesenchymal transition [40]. Extensive research on the mechanisms underlying anoikis has revealed its diverse biological functions in the metastasis and progression of various tumors. For instance, studies have demonstrated that CPT1A-mediated fatty acid oxidation promotes metastasis of colorectal cancer cells by inhibiting anoikis [41]. Chun Fen et al. uncovered that IQGAP1 enhances anoikis resistance, migration and invasion of hepatoma cells by promoting Rac1-dependent ROS accumulation and activation of Src/FAK signaling [42]. Chang et al. showed that CCN2 inhibits lung cancer metastasis by promoting DAPK-dependent anoikis and inducing EGFR degradation [43, 44]. Additionally, Shan et al. developed a prognostic model based on ARG expression profiles in endometrial cancer, which accurately reflects immune status and facilitates the evaluation of immunotherapy, providing valuable insights for individualized treatment [44]. Liu et al. systematically constructed and evaluated a prognostic model of ccRCC by screening anoikis-related genes from multiple datasets, comprising 7 ARGs. Moreover, they investigated expression differences and immune characteristics associated with the modeled genes [45]. Our study differed from theirs in several aspects: firstly, we constructed anoikis-related clusters based on ARGs expression profiles, enabling significant discrimination of clinical and immune characteristics between the clusters. Secondly, we identify TIMP1 as a key regulator of anoikis and validate its clinical properties using various datasets and *in vitro* experiments.

As ccRCC is widely recognized as an immunoresponsive tumor with high heterogeneity and metastatic potential, it exhibits unique immunological characteristics that influence its pathogenesis and treatment [46]. Research has revealed that invasive immune cells within the TME play a pivotal role in tumor proliferation, metastasis, and anti-cancer immune regulation, making them valuable therapeutic targets [46]. Furthermore, ccRCC is highly immunogenic, leading to immune cell infiltration, including the presence of cytotoxic CD8+ T cells [47, 48]. Immunoinfiltration analysis, which examines the immune cell composition within tumors, is an essential component of bioinformatics analysis. Ghatalia et al. demonstrated that the recurrence of ccRCC following surgery was associated with lower T cell infiltrate, reduced adaptive immune response, and higher neutrophil gene expression, as determined through immunoinfiltration analysis [49]. Given the significant role of immunity in ccRCC, we evaluated the immune features of the prognostic models to understand the underlying reasons for survival differences. Additionally, we analyzed the implications of immunotherapy variations in the prognostic models to provide further guidance for clinical treatment decisions and implementation.

TIMP1 functions by forming non-covalent stoichiometric complexes to inhibit the proteolytic activity of matrix metalloproteinases, thereby regulating the balance of extracellular matrix degradation during matrix remodeling [27]. Apart from its inhibitory effect on several matrix metalloproteinases crucial for tumor invasion and metastasis, TIMP1 also plays a vital role in regulating cell populations and exerting anti-apoptotic functions [50]. Numerous studies have demonstrated that abnormal TIMP1 expression is associated with unfavourable prognosis in various tumors, such as gastric cancer [51], cutaneous melanoma [52], papillary thyroid carcinoma [53] and breast cancer [26]. Based on these findings, TIMP1 has been proposed as a potential biomarker for screening, diagnosis, prognosis and monitoring of these tumors. In our study, we found that TIMP1 was significantly overexpressed in ccRCC and correlated with poor prognosis. As a potent cytokine, TIMP1 interacts with various cell-surface receptors to regulate immune cells [54]. Our study also revealed that immunosuppressive cells (Macrophage, MDSC, and Regulatory.T.cell), most immunosuppressive checkpoints, and TME scores were significantly upregulated in the high-TIMP1 group. Additionally, there was a significant positive correlation between the expression of TIMP1 and immunosuppressive cells as well as most immunosuppressive checkpoints. Therefore, these findings suggested that TIMP1 may contribute to the formation of an immunosuppressive tumor microenvironment, thereby promoting cancer cell invasion and metastasis. Consequently, TIMP1 emerges as a promising molecular target for novel preventive and therapeutic strategies for ccRCC. However, further studies are necessary to fully elucidate the molecular mechanisms underlying TIMP1 dysregulation and its role in ccRCC progression.

Although the anoikis-related prognostic model we developed demonstrates high accuracy in predicting the prognosis of ccRCC patients, this study has certain limitations. Firstly, the data analysis in this paper relied solely on publicly available data. Including clinical samples would be beneficial in order to determine the expression pattern of ARGs through immunohistochemistry and other methods, providing additional validation. Furthermore, it is essential to investigate the biological mechanism of TIMP1 in remodeling the immunosuppressive microenvironment through *in vitro* and *in vivo* experimental analyses. These additional investigations will enhance our understanding of the role of TIMP1 and further contribute to the advancement of our knowledge in this field.

## CONCLUSIONS

Anoikis played a critical role in the development of TME in ccRCC patients. AI demonstrated remarkable accuracy in evaluating patient prognosis and immune characteristics. Moreover, TIMP1 appears to contribute to the establishment of an immunosuppressive TME and holds promise as a potential therapeutic target for ccRCC.

## Supplementary Material

Supplementary Figures

Supplementary Table 1

Supplementary Table 2

Supplementary Table 3

Supplementary Tables 4 and 5
