# Supplementary material for: TIMP1 shapes an immunosuppressive microenvironment by regulating anoikis to promote the progression of clear cell renal cell carcinoma
Source: Aging (Albany NY). 2023 Sep 8;15(17):8908–29. doi: 10.18632/aging.205005 (PMC10522382; doi:10.18632/aging.205005)
Supplement: Supplementary Figures [file aging-15-205005-s001.pdf]

SUPPLEMENTARY FIGURES

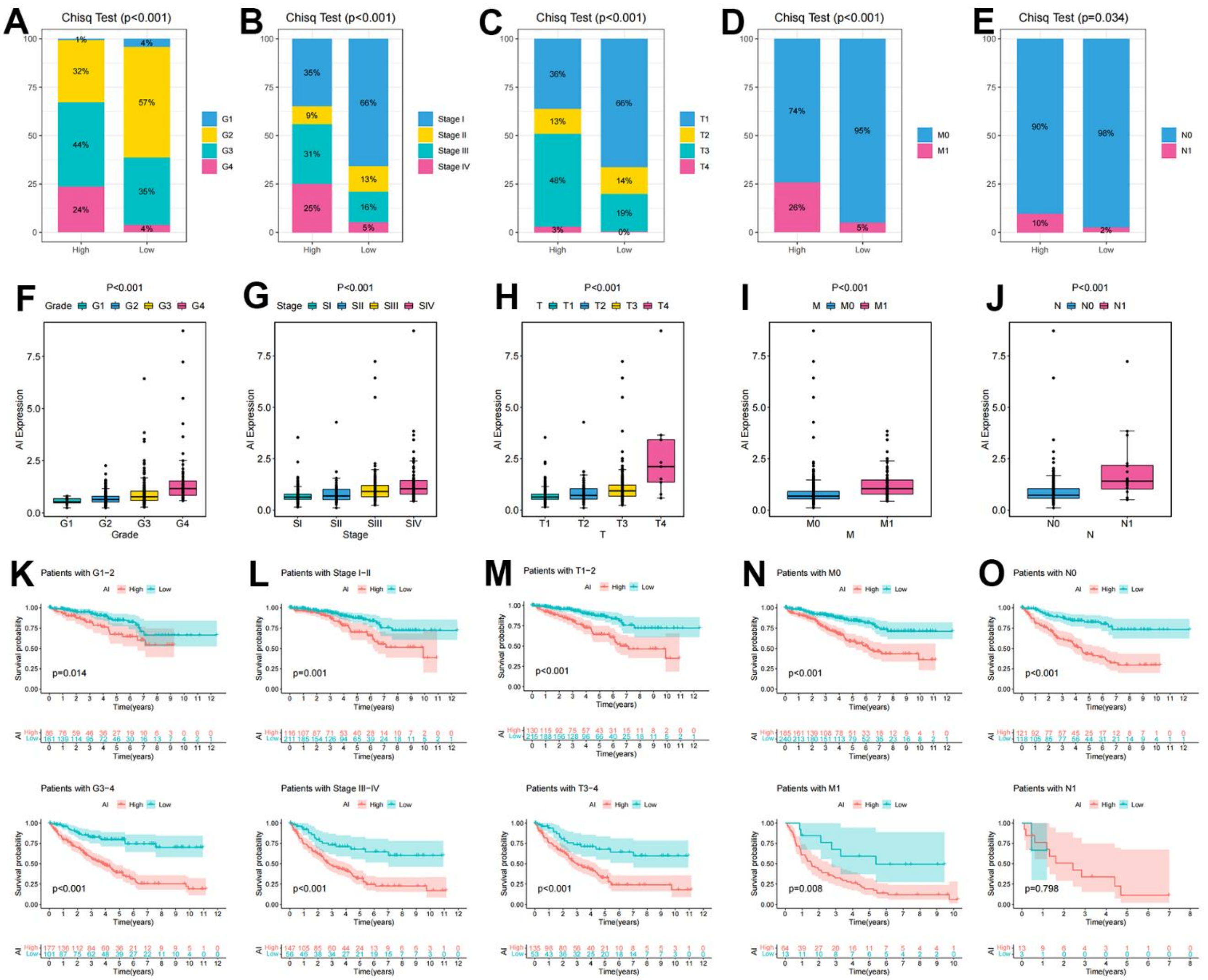

Supplementary Figure 1. Correlation of anoikis index with clinicopathological features in ccRCC. (**A**–**E**) The histogram showing the proportion of clinicopathological variables in the high and low AI groups (**A**) Grade; (**B**) Stage; (**C**) T stage; (**D**) M stage; (**E**) N stage; (**F**–**J**) Differences in AI among clinicopathological variables (**F**) Grade; (**G**) Stage; (**H**) T stage; (**I**) M stage; (**J**) N stage; (**K**–**O**) KM survival curves showing OS of AI in various clinicopathological characteristics, including TNM stage, pathological stage, age and gender of patients (**K**) Grade; (**L**) Stage; (**M**) T stage; (**N**) M stage; (**O**) N stage.

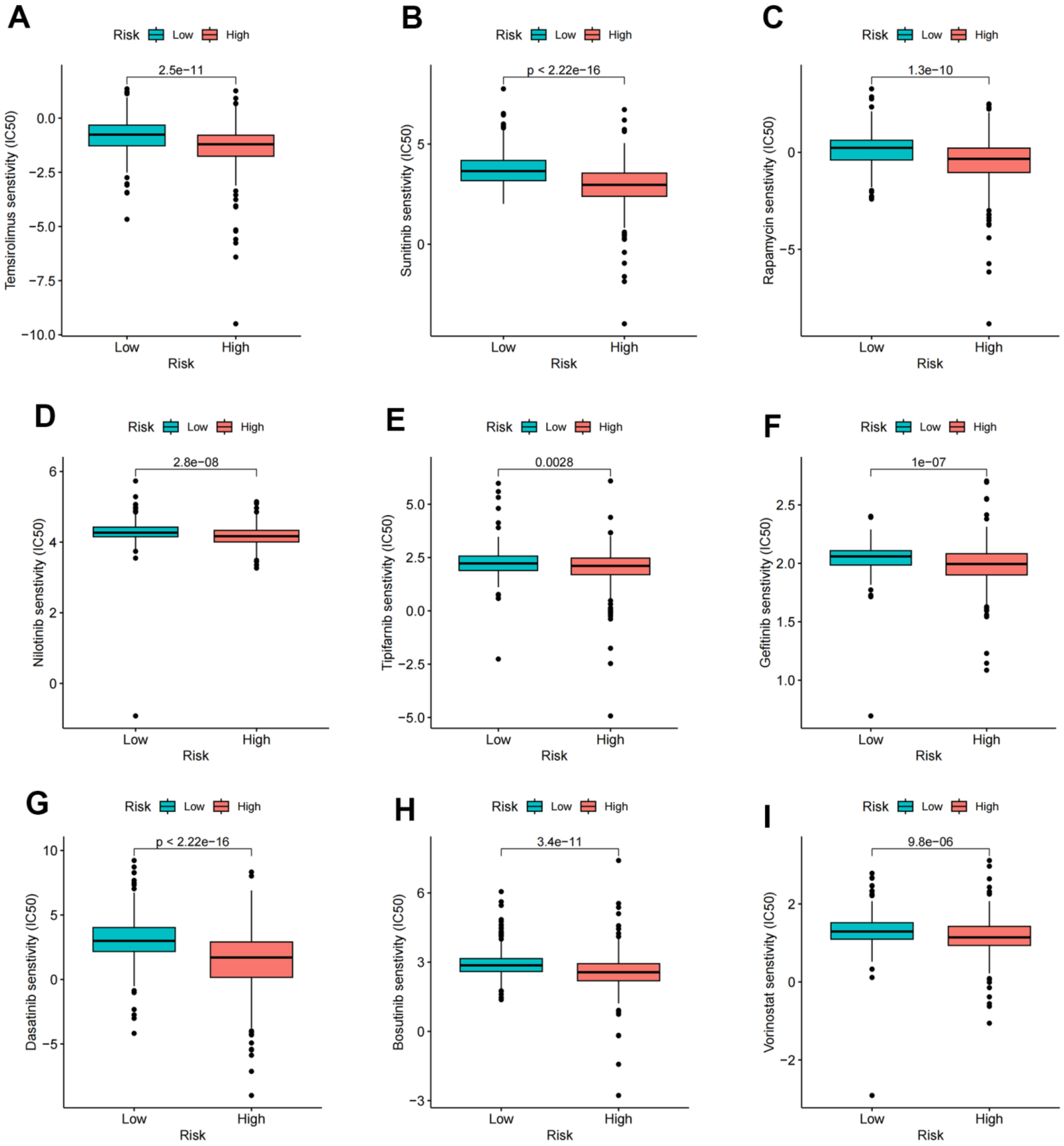

Supplementary Figure 2. Correlation between TIMP1 and drug sensitivity. (**A**–**I**) Correlation analysis of TIMP1 expression profile and drug sensitivity. (**A**) Temsirolimus; (**B**) Sunitinib; (**C**) Rapamycin; (**D**) Nilotinib; (**E**) Tipifarnib; (**F**) Gefitinib; (**G**) Dasatinib; (**H**) Bosutinib; (**I**) Vorlistat.
