# Supplementary material for: TIMP1 shapes an immunosuppressive microenvironment by regulating anoikis to promote the progression of clear cell renal cell carcinoma
Source: Aging (Albany NY). 2023 Sep 8;15(17):8908–29. doi: 10.18632/aging.205005 (PMC10522382; doi:10.18632/aging.205005)
Supplement: Supplementary Table 1 [file aging-15-205005-s002.docx]

| **Supplementary Table 1. The list of 434 anoikis-related genes (ARGs).** | |
| --- | --- |
| Gene Symbol | Relevance score |
| HSPB1 | 0.448463 |
| PCNA | 0.448463 |
| GSK3B | 0.448463 |
| NGF | 0.448463 |
| TP63 | 0.448463 |
| CTNNA1 | 0.448463 |
| KRT14 | 0.448463 |
| SPHK1 | 0.448463 |
| EHMT2 | 0.448463 |
| RAC3 | 0.448463 |
| SIRT6 | 0.448463 |
| OGT | 0.448463 |
| NDRG1 | 0.448463 |
| STK38 | 0.448463 |
| ACP1 | 0.448463 |
| FOXA1 | 0.448463 |
| RHOQ | 0.448463 |
| ONECUT1 | 0.448463 |
| S100A7 | 0.448463 |
| SRSF3 | 0.448463 |
| GKN1 | 0.448463 |
| MIR107 | 0.448463 |
| MIR630 | 0.448463 |
| RPS6KA3 | 0.488467 |
| CDC42 | 0.488467 |
| MAOA | 0.488467 |
| PIP5K1C | 0.488467 |
| JUP | 0.488467 |
| ATF2 | 0.488467 |
| NKX2-1 | 0.488467 |
| OCLN | 0.488467 |
| ID2 | 0.488467 |
| CRABP2 | 0.488467 |
| CEACAM8 | 0.488467 |
| PITPNC1 | 0.488467 |
| AFAP1L1 | 0.488467 |
| LDHA | 0.522192 |
| ANXA2 | 0.522192 |
| SPP1 | 0.522192 |
| SMARCE1 | 0.522192 |
| RBFOX2 | 0.522192 |
| QSOX1 | 0.522192 |
| PRKD1 | 0.551904 |
| MAP2K2 | 0.560494 |
| CSK | 0.560494 |
| PIK3C2B | 0.560494 |
| FOXC2 | 0.560494 |
| ARHGDIB | 0.560494 |
| ENDOG | 0.560494 |
| TAGLN | 0.560494 |
| FBLIM1 | 0.560494 |
| RACK1 | 0.560494 |
| CCDC80 | 0.560494 |
| ANGPTL2 | 0.560494 |
| HOTAIR | 0.560494 |
| SFRP1 | 0.575717 |
| GNE | 0.578766 |
| XAF1 | 0.578766 |
| NTRK3 | 0.600498 |
| TNC | 0.603468 |
| IRF6 | 0.603468 |
| CASP2 | 0.62646 |
| RHOC | 0.634223 |
| SMAD7 | 0.663935 |
| PDPK1 | 0.686462 |
| BIN1 | 0.690797 |
| TIAM1 | 0.690797 |
| ACTG1 | 0.70158 |
| ARHGDIA | 0.70158 |
| EZR | 0.70158 |
| SLC39A6 | 0.70158 |
| FOXO3 | 0.71893 |
| SERPINE1 | 0.720187 |
| IKBKG | 0.729213 |
| TFDP1 | 0.729213 |
| CRYBA1 | 0.729213 |
| ANKRD13C | 0.729213 |
| MALAT1 | 0.776761 |
| PXN | 0.78051 |
| CEACAM4 | 0.793718 |
| MAP3K1 | 0.827443 |
| CTBP1 | 0.827443 |
| F10 | 0.846184 |
| HSP90B1 | 0.846184 |
| F3 | 0.846184 |
| ADAMTSL1 | 0.846184 |
| SERPINB1 | 0.846184 |
| MIR181A1 | 0.846184 |
| MAP3K7 | 0.90721 |
| BAG4 | 0.908719 |
| TSC2 | 0.947214 |
| BUB1 | 0.972047 |
| CDC25C | 0.972047 |
| BUB3 | 0.972047 |
| FER | 0.972047 |
| SETD2 | 0.972047 |
| CDK1 | 0.972047 |
| ITGB5 | 0.972047 |
| TP73 | 0.972047 |
| MAD2L1 | 0.972047 |
| BCL2L2 | 0.972047 |
| SLCO1B3 | 0.972047 |
| DLG1 | 0.972047 |
| PDCD6IP | 0.972047 |
| SCRIB | 0.972047 |
| TDGF1 | 0.972047 |
| EDAR | 0.972047 |
| SH3GLB1 | 0.972047 |
| DYNLL2 | 0.972047 |
| SHC1 | 1.002452 |
| GLUD1 | 1.024181 |
| MYH9 | 1.024181 |
| NOTCH3 | 1.024181 |
| PTPN1 | 1.024181 |
| TPM1 | 1.024181 |
| FASN | 1.024181 |
| RPS6KB1 | 1.024181 |
| SIRT1 | 1.024181 |
| PPP2R1A | 1.024181 |
| CD151 | 1.024181 |
| CTNND1 | 1.024181 |
| MMP11 | 1.024181 |
| COL4A2 | 1.024181 |
| ARHGEF7 | 1.024181 |
| PPP2R5A | 1.024181 |
| BST2 | 1.024181 |
| PPP2R2D | 1.024181 |
| CCN1 | 1.024181 |
| CCDC178 | 1.024181 |
| MIR30C1 | 1.024181 |
| MIR30B | 1.024181 |
| MIR10A | 1.024181 |
| SNAI2 | 1.037513 |
| PTPN11 | 1.064185 |
| SLC2A1 | 1.064185 |
| HMOX1 | 1.064185 |
| PRKACA | 1.064185 |
| CBL | 1.064185 |
| PAK3 | 1.064185 |
| PIK3R2 | 1.064185 |
| CASP6 | 1.064185 |
| PPP2CA | 1.064185 |
| CD36 | 1.064185 |
| CDH3 | 1.064185 |
| LRP1 | 1.064185 |
| PTK6 | 1.064185 |
| EEF2K | 1.064185 |
| GLO1 | 1.064185 |
| PAK2 | 1.064185 |
| LPAR1 | 1.064185 |
| SFN | 1.064185 |
| ADCY10 | 1.064185 |
| TRAF2 | 1.064185 |
| PIK3R3 | 1.064185 |
| RBL2 | 1.064185 |
| SIRPA | 1.064185 |
| TNFRSF12A | 1.064185 |
| CEACAM1 | 1.064185 |
| BAG1 | 1.064185 |
| APOBEC3G | 1.064185 |
| GDF2 | 1.064185 |
| MNX1 | 1.064185 |
| VPS37A | 1.064185 |
| COL13A1 | 1.064185 |
| RAD9A | 1.064185 |
| IFI27 | 1.064185 |
| ITPRIP | 1.064185 |
| BCL2L15 | 1.064185 |
| TUBB3 | 1.084078 |
| PDGFRB | 1.097909 |
| TLR3 | 1.097909 |
| ROCK1 | 1.097909 |
| CPT1A | 1.097909 |
| NRAS | 1.097909 |
| PLAT | 1.097909 |
| CASP10 | 1.097909 |
| PAK4 | 1.097909 |
| VEGFA | 1.097909 |
| ZEB1 | 1.097909 |
| PIN1 | 1.097909 |
| UBE2C | 1.097909 |
| YWHAZ | 1.097909 |
| TWIST1 | 1.097909 |
| ELK1 | 1.097909 |
| BMP6 | 1.097909 |
| PRDX4 | 1.097909 |
| BNIP3 | 1.097909 |
| BNIP3L | 1.097909 |
| KDM3A | 1.097909 |
| LMO3 | 1.097909 |
| ZNF32 | 1.097909 |
| MIR200B | 1.097909 |
| MIR363 | 1.097909 |
| MIR525 | 1.097909 |
| KDR | 1.127622 |
| MDM2 | 1.127622 |
| PRKCI | 1.127622 |
| NFE2L2 | 1.127622 |
| RB1 | 1.127622 |
| FGF2 | 1.127622 |
| HK2 | 1.127622 |
| KL | 1.127622 |
| CRYAB | 1.127622 |
| EPHB6 | 1.127622 |
| IQGAP1 | 1.127622 |
| LTF | 1.127622 |
| SDCBP | 1.127622 |
| SPIB | 1.127622 |
| MGAT5 | 1.127622 |
| ABHD2 | 1.127622 |
| TRIM31 | 1.127622 |
| MIR1827 | 1.127622 |
| ELANE | 1.136212 |
| MET | 1.153404 |
| RAF1 | 1.154483 |
| PRKCQ | 1.154483 |
| BRCA2 | 1.154483 |
| PARP1 | 1.154483 |
| DOCK1 | 1.154483 |
| SP1 | 1.154483 |
| HAVCR2 | 1.154483 |
| VTN | 1.154483 |
| INHBB | 1.154483 |
| RANBP9 | 1.154483 |
| PDCD4 | 1.154483 |
| PRPF4B | 1.154483 |
| SESN1 | 1.154483 |
| SESN3 | 1.154483 |
| PHLDA2 | 1.154483 |
| ZBTB7A | 1.154483 |
| CD24 | 1.154483 |
| MIR141 | 1.154483 |
| PLG | 1.170042 |
| RHOB | 1.176215 |
| CCN2 | 1.176215 |
| PPP1R13B | 1.176215 |
| AKT3 | 1.179185 |
| RELA | 1.179185 |
| PRKCA | 1.179185 |
| TNFRSF1A | 1.179185 |
| FASLG | 1.179185 |
| AFP | 1.179185 |
| CEBPB | 1.179185 |
| SATB1 | 1.179185 |
| EEF1A1 | 1.179185 |
| ITGA8 | 1.179185 |
| PBK | 1.179185 |
| LTB4R2 | 1.179185 |
| CD63 | 1.179185 |
| NOX4 | 1.179185 |
| MAVS | 1.179185 |
| SERPINA1 | 1.196685 |
| TSG101 | 1.202178 |
| MIR200A | 1.202178 |
| CLU | 1.20994 |
| SPINK1 | 1.20994 |
| CPEB2 | 1.20994 |
| FBXW7-AS1 | 1.222176 |
| EPHA2 | 1.223772 |
| SOD2 | 1.223772 |
| SIRT3 | 1.223772 |
| OLFM3 | 1.223772 |
| CEMIP | 1.223772 |
| ZEB2 | 1.239652 |
| TLN1 | 1.239652 |
| EZH2 | 1.242513 |
| CDH2 | 1.244197 |
| CSNK2A1 | 1.244197 |
| EDIL3 | 1.244197 |
| EIF2AK3 | 1.263623 |
| LAMB3 | 1.263623 |
| LAMC2 | 1.263623 |
| LAMA3 | 1.263623 |
| LATS1 | 1.266514 |
| HTRA1 | 1.266514 |
| CEACAM3 | 1.266514 |
| ETV4 | 1.291216 |
| S100A4 | 1.291216 |
| NTF3 | 1.291216 |
| MIR21 | 1.291216 |
| MIR124-1 | 1.291216 |
| HMGA1 | 1.314208 |
| TNFSF10 | 1.314208 |
| SIK2 | 1.314208 |
| RIPK1 | 1.317119 |
| CXCR4 | 1.325617 |
| GLI2 | 1.335803 |
| PTHLH | 1.352479 |
| ABL1 | 1.356228 |
| MMP9 | 1.356228 |
| MAPK11 | 1.356228 |
| MYO5A | 1.374681 |
| EDA2R | 1.374681 |
| CCN6 | 1.374681 |
| FAS | 1.377181 |
| MTA1 | 1.377181 |
| MMP13 | 1.380174 |
| BIRC3 | 1.412019 |
| NQO1 | 1.414685 |
| RHOG | 1.417655 |
| CCAR2 | 1.417655 |
| NOTCH1 | 1.42001 |
| RAC1 | 1.442192 |
| GRHL2 | 1.442192 |
| CCR7 | 1.44841 |
| MSLN | 1.44841 |
| PPARG | 1.478122 |
| IL6 | 1.478122 |
| MIR145 | 1.478122 |
| XIAP | 1.497984 |
| CDK11B | 1.504984 |
| CDK11A | 1.504984 |
| HRAS | 1.514663 |
| BID | 1.529686 |
| THBS1 | 1.529686 |
| KRAS | 1.54763 |
| ITGB3 | 1.54763 |
| NTRK1 | 1.552652 |
| MYC | 1.552678 |
| PLAU | 1.552678 |
| PLK1 | 1.552678 |
| SMAD4 | 1.552678 |
| CDKN1A | 1.552678 |
| MUC1 | 1.552678 |
| PLAUR | 1.552678 |
| LGALS1 | 1.552678 |
| PYCARD | 1.552678 |
| SESN2 | 1.552678 |
| CDKN1B | 1.574273 |
| ATF4 | 1.574273 |
| KLF12 | 1.574273 |
| CDKN2A | 1.594698 |
| MAPK8 | 1.594698 |
| PIK3CB | 1.594698 |
| CLDN1 | 1.594698 |
| MIR26A1 | 1.594698 |
| MIR204 | 1.594698 |
| AR | 1.59767 |
| TNFRSF10B | 1.614124 |
| CXCL8 | 1.614124 |
| MIR200C | 1.614124 |
| CASP9 | 1.632686 |
| MTDH | 1.632686 |
| CDKN3 | 1.645226 |
| MMP2 | 1.650489 |
| TGFB1 | 1.66342 |
| HMCN1 | 1.66342 |
| ITGA4 | 1.66762 |
| FADD | 1.66762 |
| CD44 | 1.680662 |
| ABHD4 | 1.684149 |
| BAK1 | 1.706999 |
| LGALS3 | 1.71865 |
| CXCL12 | 1.753584 |
| MAP2K1 | 1.77256 |
| PIK3R1 | 1.797109 |
| DAPK1 | 1.821627 |
| ITGB4 | 1.831272 |
| YAP1 | 1.842585 |
| PIK3CG | 1.843222 |
| EGF | 1.846404 |
| E2F1 | 1.856835 |
| HGF | 1.858571 |
| SKP2 | 1.883073 |
| CHEK2 | 1.883697 |
| CDCP1 | 1.906433 |
| CALR | 1.919438 |
| CCND1 | 1.955544 |
| CTTN | 1.955544 |
| PTK2B | 1.969084 |
| ITGA3 | 1.971719 |
| TLE5 | 1.97341 |
| MYBBP1A | 1.995728 |
| DAP3 | 2.028509 |
| HIF1A | 2.037955 |
| RHOA | 2.041556 |
| CFLAR | 2.055758 |
| ILK | 2.055982 |
| ITGA6 | 2.082232 |
| IGF1R | 2.118469 |
| IGF1 | 2.119635 |
| STK11 | 2.12343 |
| AKT2 | 2.154229 |
| CSPG4 | 2.166408 |
| BSG | 2.166408 |
| BDNF | 2.178729 |
| ITGA2 | 2.192107 |
| TIMP1 | 2.215896 |
| MTOR | 2.221419 |
| BIRC5 | 2.226358 |
| ANXA5 | 2.236495 |
| BRAF | 2.304839 |
| CYCS | 2.308967 |
| ANGPTL4 | 2.367288 |
| ERBB2 | 2.401457 |
| PDK4 | 2.405831 |
| TLE1 | 2.447864 |
| PTEN | 2.48593 |
| BCAR1 | 2.503318 |
| FN1 | 2.508724 |
| BAX | 2.514714 |
| PTGS2 | 2.656932 |
| MAPK3 | 2.694114 |
| ITGAV | 2.837788 |
| PAK1 | 2.892794 |
| PIK3CA | 2.905329 |
| BAD | 2.92408 |
| CDH1 | 3.019521 |
| CASP3 | 3.079218 |
| BCL2L1 | 3.319262 |
| TP53 | 3.451738 |
| MCL1 | 3.533736 |
| ITGA5 | 3.630884 |
| BMF | 3.685458 |
| MAPK1 | 3.685743 |
| ZNF304 | 3.910815 |
| CTNNB1 | 3.94576 |
| DAPK2 | 3.958509 |
| SIK1 | 4.038517 |
| STAT3 | 4.086776 |
| PTRH2 | 4.139895 |
| EGFR | 4.384342 |
| CASP8 | 4.430302 |
| BCL2 | 4.483292 |
| CEACAM5 | 4.617144 |
| ITGB1 | 4.938623 |
| AKT1 | 5.331155 |
| CAV1 | 5.393483 |
| CEACAM6 | 6.054066 |
| SRC | 6.104873 |
| BCL2L11 | 6.624886 |
| NTRK2 | 7.183176 |
| PTK2 | 7.193454 |
