# Supplementary material for: TIMP1 shapes an immunosuppressive microenvironment by regulating anoikis to promote the progression of clear cell renal cell carcinoma
Source: Aging (Albany NY). 2023 Sep 8;15(17):8908–29. doi: 10.18632/aging.205005 (PMC10522382; doi:10.18632/aging.205005)
Supplement: Supplementary Table 2 [file aging-15-205005-s003.docx]

**Supplementary Table 2. Differentially expressed genes between cancer and normol tissues.**

Gene conMean treatMean logFC pValue fdr

AL390294.1 0.681515775 0.023148794 -4.879738134 6.85E-78 2.08E-73

SEMG2 0.900320841 0.004508262 -7.641724187 1.14E-75 1.27E-71

AC095060.1 0.093947619 0.004017706 -4.547412826 1.25E-75 1.27E-71

LINC01351 0.127591514 0.00827802 -3.94610279 2.60E-75 1.98E-71

LINC00307 0.201390927 0.044907318 -2.164976217 2.92E-74 1.77E-70

AC010872.1 0.155192895 0.00359749 -5.430927827 2.52E-71 1.27E-67

LINC01739 0.201955825 0.026993001 -2.903382459 1.65E-69 7.13E-66

AC025674.1 0.232245909 0.039054553 -2.572090514 3.40E-69 1.29E-65

AC016526.1 1.963628987 0.016483172 -6.896384648 9.07E-69 3.06E-65

AC009035.1 0.802489413 0.008121468 -6.62659804 2.25E-67 6.83E-64

AC090709.1 2.120159188 0.009765957 -7.7621955 4.17E-67 1.15E-63

AC091891.1 0.103203726 0.003919853 -4.718551859 3.49E-66 8.83E-63

LINC01665 0.14951468 0.01605355 -3.219322912 3.98E-66 9.29E-63

LINC02778 0.526030675 0.00492996 -6.737427187 1.07E-65 2.31E-62

LINC02121 11.54835293 0.039456535 -8.193207106 2.36E-65 4.77E-62

AC079310.1 2.567111602 0.005007551 -9.00182507 3.58E-65 6.78E-62

AC099552.3 0.953525007 0.046739262 -4.350563923 7.92E-65 1.41E-61

AC092078.1 0.897640769 0.012961251 -6.113861335 3.58E-64 6.04E-61

AL161793.2 1.789116692 0.070438836 -4.666732616 3.63E-63 5.80E-60

AC078918.1 0.120593423 0.00226875 -5.732109825 1.35E-62 2.06E-59

LINC00919 0.081345574 0.028360126 -1.520199901 1.53E-61 2.21E-58

AC092691.1 0.214389811 0.039242516 -2.449746884 1.93E-61 2.66E-58

AC107057.1 4.639346409 0.066037213 -6.13449853 3.24E-60 4.10E-57

Z82170.1 0.043087937 0.006164049 -2.805333725 4.83E-60 5.86E-57

UNCX 0.739912041 0.004894988 -7.239904687 6.90E-60 8.05E-57

SLC16A14P1 0.055560298 0.002601658 -4.41655101 4.47E-59 5.02E-56

AC104072.1 2.087981359 0.10882948 -4.26196752 5.45E-59 5.90E-56

AL390066.1 0.846606786 0.03684712 -4.52206827 6.50E-59 6.80E-56

WSPAR 0.218663713 0.010284457 -4.410176259 8.76E-59 8.86E-56

AC015522.1 0.597301022 0.016214856 -5.203070041 9.57E-59 9.36E-56

AC024132.3 0.17602742 0.023681612 -2.893960962 2.93E-58 2.78E-55

TMEM207 10.33718361 0.036153342 -8.159498463 6.98E-58 6.42E-55

PSKH2 0.174606978 0.009372287 -4.21956622 7.75E-58 6.91E-55

AC103563.8 0.229738871 0.015813256 -3.860788614 1.59E-57 1.37E-54

AC112243.1 2.510130556 0.00456142 -9.104063777 2.89E-57 2.44E-54

DEFB132 0.217217713 0.013522546 -4.005703068 1.31E-56 1.07E-53

IFNWP5 0.103322243 0.00109032 -6.566255743 3.74E-56 2.98E-53

AC062015.1 0.589007783 0.006299614 -6.546879534 3.85E-56 2.99E-53

AC009478.1 0.040452423 0.002333983 -4.115360322 1.19E-55 9.03E-53

FSHB 0.028091268 0.001752159 -4.002915885 1.63E-55 1.21E-52

AL512363.1 0.582540165 0.080782932 -2.850235109 2.21E-55 1.60E-52

HELT 0.461585281 0.012563621 -5.199273025 5.38E-55 3.79E-52

AC019055.1 0.078188798 0.00973353 -3.00592685 5.94E-55 4.09E-52

LINC02343 1.984954556 0.084774488 -4.549332002 2.74E-54 1.85E-51

BX842568.4 0.636007336 0.052078497 -3.610283676 3.78E-54 2.44E-51

AC022039.1 0.409515182 0.002774959 -7.205306845 4.98E-54 3.15E-51

LINC01983 2.205005878 0.035134937 -5.971732362 1.14E-53 7.07E-51

CST9 0.207311974 0.006563405 -4.981215193 2.19E-53 1.30E-50

CRISP2 0.437009228 0.043993733 -3.312293817 2.59E-53 1.51E-50

AC092851.1 0.699629138 0.128656505 -2.443065973 2.77E-53 1.59E-50

AC109439.1 0.184392107 0.031401326 -2.553879494 3.58E-53 2.01E-50

AC093904.2 0.5088987 0.075600472 -2.750911359 1.41E-52 7.65E-50

BMPR1B-DT 1.455253724 0.054169588 -4.747643773 4.55E-52 2.42E-49

AC061975.6 0.140730747 0.011548925 -3.607107098 7.34E-52 3.84E-49

SCHLAP1 0.186273985 0.021338302 -3.125908946 8.92E-52 4.59E-49

HCRTR2 0.304633979 0.006200425 -5.618566048 1.27E-51 6.40E-49

AP000697.1 0.789200033 0.024213765 -5.026491691 1.39E-51 6.90E-49

LINC02410 0.571313013 0.023414045 -4.608835299 1.49E-51 7.28E-49

AC007333.2 0.10638554 0.021216114 -2.32606976 1.78E-51 8.57E-49

AL079301.1 0.027103401 0.002198243 -3.624051057 2.40E-51 1.14E-48

AC093787.1 0.561227751 0.017361149 -5.01465199 3.70E-51 1.73E-48

AC073172.1 5.011077792 0.038402231 -7.027787015 4.22E-51 1.94E-48

LINC01976 1.911128415 0.059512246 -5.005094333 6.44E-51 2.92E-48

AL162400.2 0.939149524 0.046466656 -4.337087133 6.92E-51 3.09E-48

LINC02437 8.152251411 0.029143737 -8.127868857 1.22E-50 5.37E-48

LINC01889 0.082898686 0.002997713 -4.789415271 1.28E-50 5.53E-48

AC022424.2 0.184594335 0.04267123 -2.113022693 2.00E-50 8.57E-48

SEMG1 0.112842575 0.002920613 -5.271896604 2.72E-50 1.15E-47

LINC02149 0.036407422 0.002750653 -3.72638633 3.07E-50 1.28E-47

AP006333.2 0.1249973 0.00719627 -4.118503814 3.53E-50 1.45E-47

ODAM 0.974284077 0.116593312 -3.062857451 3.60E-50 1.46E-47

AC025154.2 1.175533546 0.063594381 -4.208272597 4.31E-50 1.72E-47

ARSFP1 0.176617248 0.010765216 -4.036177006 6.94E-50 2.73E-47

AC092078.2 2.525482063 0.022051541 -6.839535523 1.07E-49 4.18E-47

CAPZA3 0.101473449 0.015757217 -2.687017653 1.09E-49 4.19E-47

AL110505.1 0.172727548 0.010326537 -4.064069755 1.24E-49 4.70E-47

AP001207.3 0.655183013 0.029241867 -4.485790639 2.14E-49 8.02E-47

AL354863.1 0.911143784 0.039915811 -4.512646505 2.48E-49 9.19E-47

HTR3B 0.180277478 0.025521096 -2.820456977 2.78E-49 1.02E-46

AL161716.1 0.365134658 0.041240283 -3.14630247 5.50E-49 1.99E-46

POU3F4 0.835144528 0.156478307 -2.416063221 5.73E-49 2.05E-46

AC106892.1 0.333740325 0.011549832 -4.852782295 6.09E-49 2.15E-46

SIRLNT 1.948538931 0.046489317 -5.389349724 7.57E-49 2.64E-46

AC063926.1 0.134522206 0.01592212 -3.078739991 1.05E-48 3.63E-46

AC105398.2 0.402198171 0.048654294 -3.047267477 1.18E-48 4.02E-46

LINC01571 3.224633306 0.024914233 -7.016021123 1.75E-48 5.89E-46

AC007993.2 2.418381238 0.043957725 -5.781781148 1.79E-48 5.97E-46

AC104237.2 3.063654486 0.149280169 -4.359159176 3.58E-48 1.18E-45

LRRC52 0.571957406 0.092540825 -2.627745845 5.04E-48 1.65E-45

AC243773.1 0.218923416 0.038260078 -2.516514551 5.45E-48 1.76E-45

FLRT1 0.277995428 0.017321335 -4.004439115 7.69E-48 2.46E-45

OLFM3 0.244440244 0.006126902 -5.318180124 1.17E-47 3.67E-45

RNU6-911P 0.449346735 0.020736954 -4.437553231 1.60E-47 4.95E-45

AC092903.1 0.493034774 0.071341524 -2.788875471 1.67E-47 5.13E-45

ENOX1-AS2 0.048206418 0.007654488 -2.654847486 1.93E-47 5.84E-45

GAMTP2 0.088553382 0.013317418 -2.733232983 2.45E-47 7.36E-45

AC060834.1 0.333549907 0.012456846 -4.742891851 3.20E-47 9.50E-45

CHL1-AS2 1.097583107 0.105749349 -3.375609489 5.86E-47 1.73E-44

RNU7-159P 1.965865924 0.043700314 -5.491377477 6.61E-47 1.93E-44

PRR35 5.78884519 0.039927477 -7.179749839 7.00E-47 2.02E-44

NXPH2 2.683930411 0.205499622 -3.707139619 7.63E-47 2.19E-44

ERVE-1 3.415181925 0.098634548 -5.113725565 1.27E-46 3.60E-44

LINC01831 0.275826823 0.012066788 -4.514649103 1.42E-46 3.99E-44

AC016825.1 0.039076036 0.003462517 -3.496391194 1.45E-46 4.04E-44

AC106772.2 2.733021082 0.080478581 -5.085747917 2.42E-46 6.61E-44

LINC01612 3.150561376 0.338202929 -3.219647854 2.61E-46 7.00E-44

AL122058.1 0.055198496 0.009756077 -2.500255893 2.60E-46 7.00E-44

VGLL1 5.806579646 0.073854644 -6.296856142 3.31E-46 8.82E-44

AC087857.1 0.232266461 0.03943968 -2.558061085 3.37E-46 8.90E-44

AL138830.2 0.067379153 0.005401604 -3.640842624 4.97E-46 1.30E-43

LINC00507 0.028872321 0.004749853 -2.603732205 8.87E-46 2.30E-43

AC104237.3 5.981177639 0.248642781 -4.588283116 2.08E-45 5.34E-43

SRGAP3-AS4 0.351621848 0.048177874 -2.867582082 2.88E-45 7.34E-43

PPY2P 0.069510122 0.004354747 -3.996562318 3.64E-45 9.19E-43

C4orf54 1.001885577 0.008606728 -6.863037157 5.94E-45 1.49E-42

OR7E108P 0.064666678 0.004748983 -3.767332079 7.80E-45 1.94E-42

FAM245A 3.000613271 0.065796591 -5.511100753 1.07E-44 2.63E-42

AC024022.1 1.229372586 0.048231263 -4.671809832 1.12E-44 2.75E-42

AC090707.1 0.059601148 0.004641992 -3.682524131 1.63E-44 3.94E-42

BRINP3 0.9999353 0.050759399 -4.300087869 1.77E-44 4.25E-42

AC012123.2 0.113523962 0.010353422 -3.454817304 1.98E-44 4.73E-42

AL691403.1 0.114232936 0.028358281 -2.010136712 3.28E-44 7.77E-42

OR7E91P 0.330061376 0.020151582 -4.033769291 3.50E-44 8.23E-42

ATP6V1G3 18.67342761 5.808373219 -1.684780704 4.22E-44 9.84E-42

TAFA4 0.180767364 0.009502146 -4.249737067 4.33E-44 1.00E-41

AC026167.1 0.245958793 0.037253323 -2.722975592 4.98E-44 1.14E-41

GUCA1C 0.391727193 0.045102871 -3.118558098 5.07E-44 1.16E-41

AC011625.1 0.081466639 0.005018894 -4.020767891 6.71E-44 1.52E-41

EDDM3A 0.726525732 0.027133891 -4.742846025 8.93E-44 2.01E-41

ZNF209P 0.809387024 0.100985386 -3.002683196 9.38E-44 2.09E-41

FRMD7 1.402549824 0.10776412 -3.702103197 1.06E-43 2.34E-41

LINC01555 0.191132205 0.00654639 -4.867727583 1.18E-43 2.58E-41

KCNE1B 0.313196344 0.00880142 -5.153187248 1.37E-43 2.98E-41

ATP12A 4.346723687 0.029698188 -7.193409661 1.79E-43 3.88E-41

AQP2 790.8741995 1.563995998 -8.9820676 2.13E-43 4.55E-41

HMX2 0.703450176 0.017327803 -5.343287592 2.13E-43 4.55E-41

OR5E1P 0.026393354 0.001668292 -3.983731302 2.19E-43 4.64E-41

AL133320.2 0.064209851 0.006344276 -3.339267271 2.21E-43 4.65E-41

RBBP8NL 0.495467476 0.042701257 -3.536439927 3.00E-43 6.28E-41

AC004870.4 0.575458316 0.063892965 -3.170982437 3.79E-43 7.87E-41

PLA2G3 0.115454612 0.026275728 -2.135523192 4.10E-43 8.46E-41

CTSLP1 0.363592567 0.027684132 -3.715191507 4.53E-43 9.28E-41

ADAMTS19-AS1 0.12176259 0.005640632 -4.432070267 5.14E-43 1.05E-40

GPC5 4.265916853 0.074618444 -5.837179765 5.28E-43 1.07E-40

AL355612.1 0.194537582 0.02911849 -2.740041445 5.30E-43 1.07E-40

CEACAM7 0.092009993 0.031127058 -1.563621336 5.93E-43 1.18E-40

AL353699.1 0.925549902 0.316665739 -1.547349987 8.29E-43 1.64E-40

AC051618.1 0.335166832 0.021655253 -3.952090428 1.06E-42 2.08E-40

GP2 3.012645749 0.025188552 -6.902119031 1.11E-42 2.17E-40

GATA3-AS1 1.868654362 0.074523803 -4.648154642 1.15E-42 2.24E-40

AL139280.1 9.836985751 0.089441661 -6.781125519 1.18E-42 2.27E-40

DUSP9 46.69976037 0.385074571 -6.922133483 1.23E-42 2.37E-40

SLC9A4 12.84169881 0.161819483 -6.310306935 1.39E-42 2.65E-40

UMOD 2746.672191 6.621687491 -8.696270098 1.69E-42 3.20E-40

AL009178.1 0.063390009 0.006429324 -3.301516434 1.78E-42 3.35E-40

AC093904.4 0.413038233 0.044844779 -3.203263397 2.56E-42 4.80E-40

MUC15 28.05434097 0.18735572 -7.22630019 3.02E-42 5.61E-40

PVT1 0.124390722 3.131098603 4.653718156 3.04E-42 5.62E-40

ACP3 12.7747708 0.272862307 -5.54898048 3.10E-42 5.69E-40

XAGE2 0.238908361 0.048475947 -2.30111634 3.13E-42 5.73E-40

AL020994.1 0.233945841 0.060248198 -1.957184573 4.24E-42 7.71E-40

NELL1 8.886163536 0.154001832 -5.850541278 4.63E-42 8.36E-40

AP000696.1 3.178654573 0.055359656 -5.84343746 4.71E-42 8.46E-40

SLC12A1 255.6958626 0.835677082 -8.257267531 4.99E-42 8.90E-40

RALYL 3.662808025 0.072170966 -5.665387711 5.02E-42 8.90E-40

AC010329.1 0.400877572 0.025315899 -3.985046086 5.19E-42 9.16E-40

SERPINA5 93.15671236 2.961056878 -4.975475618 5.71E-42 1.00E-39

LINC01606 2.060511101 0.214272976 -3.265480419 7.24E-42 1.26E-39

CDKN2A 0.063563294 1.927830869 4.922640777 7.21E-42 1.26E-39

AC073336.1 1.319167483 0.002235157 -9.205035608 7.38E-42 1.27E-39

OVCH2 3.986696969 0.19003316 -4.390870859 7.66E-42 1.31E-39

ATP1A1 684.389025 98.81102022 -2.792072767 7.64E-42 1.31E-39

HRG 10.93005619 2.354339139 -2.21490676 7.84E-42 1.33E-39

CA10 3.692737373 0.116661506 -4.984290152 8.91E-42 1.50E-39

TMEM45B 18.58362124 1.20224281 -3.950231452 1.09E-41 1.83E-39

PAQR7 24.04133847 7.993242147 -1.588664519 1.20E-41 2.01E-39

CLDN16 29.83053597 0.535628503 -5.799413355 1.22E-41 2.01E-39

AP005432.2 13.71497378 0.141972591 -6.593995623 1.25E-41 2.06E-39

MIR200CHG 2.760586858 0.476473598 -2.534506816 1.27E-41 2.09E-39

KNG1 288.1662501 2.998896111 -6.586326023 1.30E-41 2.12E-39

IRX2 8.608547688 0.266990544 -5.010909314 1.31E-41 2.13E-39

AP006333.3 0.330198786 0.03891969 -3.08476268 1.36E-41 2.20E-39

AC124017.1 4.182311133 0.042685007 -6.614427172 1.43E-41 2.29E-39

AC044810.2 0.051810267 0.00411389 -3.654663035 1.68E-41 2.68E-39

FXYD4 287.305672 2.830038334 -6.665621069 1.73E-41 2.75E-39

Z98259.1 0.058954169 0.002051504 -4.844840163 1.74E-41 2.75E-39

AC087045.3 4.052397141 0.087205346 -5.538215175 1.77E-41 2.76E-39

ACOT12 1.010256045 0.050829479 -4.312911721 1.76E-41 2.76E-39

KCTD8 0.517419083 0.030465527 -4.086083649 2.17E-41 3.37E-39

CLDN19 32.00252953 0.627481467 -5.672469283 2.32E-41 3.59E-39

SCNN1G 63.76658065 2.217129484 -4.846035588 2.39E-41 3.69E-39

AC078980.1 0.497170879 0.063134865 -2.977232962 2.47E-41 3.79E-39

KCNJ16 83.59537583 28.69949732 -1.542397673 2.60E-41 3.97E-39

ESRRB 12.01685323 0.186421239 -6.010349107 3.15E-41 4.79E-39

CDH16 346.7096125 64.79555573 -2.419761067 3.44E-41 5.19E-39

TFAP2B 6.527445387 0.126779197 -5.686128545 3.64E-41 5.46E-39

AL163932.1 0.301231416 0.023975028 -3.651267807 3.89E-41 5.82E-39

GSTM3 60.00433532 5.961968865 -3.331205993 4.21E-41 6.20E-39

HSF4 0.362248841 19.79881145 5.772288941 4.21E-41 6.20E-39

MPPED2-AS1 0.935704936 0.075936537 -3.623187556 4.36E-41 6.39E-39

TMEM238L 6.459414075 0.046910823 -7.105338689 4.59E-41 6.70E-39

ELF5 11.67010111 0.047323242 -7.94605242 4.78E-41 6.94E-39

KCNJ1 103.415914 1.338793899 -6.271380518 4.86E-41 7.02E-39

HS6ST2 10.61975078 0.1425965 -6.218667533 5.22E-41 7.50E-39

TRPV6 1.318867929 0.041961692 -4.974083454 5.25E-41 7.51E-39

LINC02038 2.406519517 0.232967204 -3.368749347 5.50E-41 7.84E-39

ESRRG 9.957923889 1.152864747 -3.110621721 5.61E-41 7.95E-39

LINC00379 7.239985654 0.343279104 -4.398532892 5.85E-41 8.26E-39

KLHL14 5.137352938 0.361725161 -3.828059331 6.06E-41 8.51E-39

LHX1-DT 5.958081772 0.343124838 -4.118042455 6.11E-41 8.54E-39

EPB41L5 11.42652749 3.443815509 -1.730307274 6.42E-41 8.89E-39

AC099482.1 0.258946466 0.073029839 -1.826095912 7.12E-41 9.83E-39

WNT9B 3.030977214 0.160114388 -4.242608143 7.69E-41 1.06E-38

TMEM74B 0.221149585 2.937309341 3.731400768 8.63E-41 1.18E-38

AC103957.2 0.270222878 0.039860335 -2.76112408 9.08E-41 1.24E-38

EMX1 14.03607081 2.292337375 -2.614247837 9.41E-41 1.27E-38

PTGER1 9.081212405 0.265394724 -5.096673324 9.60E-41 1.29E-38

IRX1 9.419194583 0.396656897 -4.569640162 9.65E-41 1.29E-38

AC007342.8 0.757941965 0.1095484 -2.790518975 9.66E-41 1.29E-38

APOC1 2.677430229 88.20676067 5.041968351 9.59E-41 1.29E-38

AL391845.2 0.016768096 0.590421715 5.137955007 1.03E-40 1.35E-38

SFRP1 99.57998058 2.681934537 -5.214509806 1.10E-40 1.43E-38

ARHGEF39 0.150836148 1.005209646 2.736442298 1.10E-40 1.43E-38

COL4A6 2.555318328 0.127328038 -4.326880983 1.11E-40 1.44E-38

AL035604.1 0.459733842 0.022014302 -4.384285884 1.11E-40 1.44E-38

DBT 8.216476389 2.868898877 -1.518022714 1.14E-40 1.47E-38

ARMH4 7.352378422 0.628665983 -3.547845416 1.16E-40 1.49E-38

RASL11B 16.23551784 0.813254989 -4.319301823 1.18E-40 1.51E-38

ANXA9 21.50598792 5.687017928 -1.918994148 1.19E-40 1.52E-38

EHF 19.91221951 0.525110237 -5.244889908 1.22E-40 1.54E-38

WNK4 19.53498876 1.153112157 -4.082455678 1.26E-40 1.60E-38

PRR15 14.4380112 0.873984751 -4.04612011 1.31E-40 1.65E-38

SOWAHA 5.387074083 0.615433943 -3.129825984 1.31E-40 1.65E-38

FAM167A-AS1 0.166411575 0.012964989 -3.682062946 1.32E-40 1.65E-38

HOXD8 40.30395319 11.82344717 -1.769270632 1.64E-40 2.03E-38

GABRD 0.240042364 8.697100451 5.179173548 1.67E-40 2.06E-38

CASR 24.75163581 0.537988906 -5.523803641 1.70E-40 2.09E-38

DDB2 2.728486625 11.14791891 2.030601536 1.72E-40 2.11E-38

MCCD1 21.57575429 0.998619203 -4.433332538 1.78E-40 2.18E-38

SPAG4 1.464010193 21.43590853 3.872032062 1.83E-40 2.23E-38

AIF1L 235.4903638 25.14391291 -3.227386941 1.85E-40 2.25E-38

GPC3 48.85896707 3.517973163 -3.795806982 1.90E-40 2.30E-38

ERVMER34-1 4.557869131 0.177435501 -4.682992905 2.02E-40 2.41E-38

FAM3B 6.902502356 0.430629103 -4.002601748 2.02E-40 2.41E-38

CLDN8 70.92163653 5.737231068 -3.627799298 2.01E-40 2.41E-38

RPS6KA6 5.018454776 0.633164016 -2.986592043 2.14E-40 2.54E-38

EFHD1 97.58812811 15.57314109 -2.647645686 2.18E-40 2.58E-38

HSPA2 52.34832846 3.354695578 -3.963889711 2.22E-40 2.62E-38

RNF150 8.595075058 0.989696965 -3.118451479 2.26E-40 2.66E-38

MRPS9-AS2 0.662566081 0.096931969 -2.773019882 2.30E-40 2.70E-38

AC108860.2 1.185898127 0.062340055 -4.249676838 2.31E-40 2.70E-38

NRK 5.482930404 0.191085479 -4.842657106 2.34E-40 2.70E-38

GABRA2 2.319220942 0.083128469 -4.802153821 2.33E-40 2.70E-38

CDKN2B-AS1 0.008284723 0.172320544 4.378497477 2.34E-40 2.70E-38

CLUL1 1.971198492 0.177300591 -3.474803806 2.37E-40 2.72E-38

SLC52A3 7.860796167 0.926636243 -3.084600425 2.39E-40 2.74E-38

CYS1 128.2017628 30.65217619 -2.064354691 2.56E-40 2.92E-38

PRDM16 4.665680464 0.34676225 -3.750068752 2.58E-40 2.93E-38

MECOM 26.06859747 4.414945809 -2.561845331 2.68E-40 3.04E-38

PIK3C2G 3.18925628 0.209681767 -3.926946714 2.84E-40 3.20E-38

MAP6 3.296785568 0.64103857 -2.362576985 2.92E-40 3.28E-38

PRDM16-DT 13.26859927 0.316471523 -5.389796579 2.97E-40 3.33E-38

LINC02404 0.054429731 0.006804432 -2.99984824 3.01E-40 3.36E-38

PRKAR2B 7.447831625 1.567726298 -2.24814675 3.09E-40 3.44E-38

AC239801.1 0.534656421 0.044862986 -3.57501454 3.36E-40 3.72E-38

GGT6 29.37386549 0.964887929 -4.928027937 3.39E-40 3.73E-38

AC068631.1 0.455303673 0.022588444 -4.333172317 3.40E-40 3.73E-38

NCAPD2P1 0.085435985 0.008688704 -3.297630983 3.53E-40 3.87E-38

AC104407.1 0.044392585 0.002197351 -4.336481737 3.70E-40 4.04E-38

KCNJ10 26.30641911 0.373563246 -6.137918551 3.74E-40 4.07E-38

SLC16A1-AS1 0.065275 0.610393011 3.225135987 4.00E-40 4.33E-38

ANKRD62P1 0.04180122 0.007224403 -2.532594775 4.43E-40 4.79E-38

TREM2 1.409428622 23.41532467 4.054270721 4.52E-40 4.85E-38

SIGLEC8 0.134383141 2.851051209 4.407069888 4.56E-40 4.87E-38

DEPTOR 31.59801042 10.66494454 -1.566957258 4.61E-40 4.90E-38

FOXCUT 3.003648866 0.225931326 -3.732759946 4.94E-40 5.24E-38

LINC01762 3.893641994 0.161101776 -4.595075936 4.96E-40 5.24E-38

SFTA1P 0.055659013 1.74793422 4.972891763 5.13E-40 5.40E-38

KLHL13 7.601908514 1.672901179 -2.184009438 5.16E-40 5.42E-38

MTURN 43.96015165 5.315204534 -3.047999249 5.26E-40 5.50E-38

SCNN1B 28.31457642 1.66903298 -4.08446058 5.36E-40 5.55E-38

CLDN10 38.11023033 7.829517671 -2.283182988 5.36E-40 5.55E-38

PEG3 2.886837717 0.568261782 -2.344862413 5.67E-40 5.83E-38

SPTBN2 32.95805167 1.476465481 -4.480411427 5.84E-40 5.98E-38

EPCAM 147.6145346 19.71968476 -2.904126387 5.95E-40 6.07E-38

HLA-F 6.785350208 37.5298872 2.46754477 6.29E-40 6.41E-38

HADH 52.33342792 15.4898121 -1.756413117 6.35E-40 6.45E-38

HYKK 1.821707118 0.461538218 -1.980769009 6.66E-40 6.71E-38

KLRG2 5.777790892 0.161630276 -5.159748619 6.74E-40 6.77E-38

ARRB2 5.009289611 14.29244323 1.512574632 6.92E-40 6.93E-38

TMEM178A 11.01851029 1.18546425 -3.216405123 7.12E-40 7.10E-38

COL5A3 0.538646386 6.329459402 3.554671905 7.68E-40 7.63E-38

SLC15A2 6.131489517 0.749574899 -3.032093041 7.75E-40 7.68E-38

SPATA16 0.054740578 0.005235631 -3.386175227 8.30E-40 8.20E-38

NHLRC4 6.565288281 0.657599657 -3.31957691 8.36E-40 8.23E-38

AP1M2 36.51333944 6.079679493 -2.586356446 8.52E-40 8.36E-38

USP44 0.693559606 0.088814393 -2.965154474 8.60E-40 8.41E-38

RANBP3L 19.46878908 0.490052256 -5.312083747 9.27E-40 9.04E-38

AC012123.1 0.564725407 0.040609336 -3.79766619 9.46E-40 9.20E-38

AC108156.1 0.394823743 0.052841737 -2.901458966 9.51E-40 9.22E-38

BSND 15.37960198 1.253583121 -3.616888602 9.80E-40 9.45E-38

NOL3 3.572675417 38.74501045 3.43893378 9.81E-40 9.45E-38

PCARE 0.227484412 0.004686897 -5.600990685 9.92E-40 9.50E-38

AL049838.1 9.722105714 0.849197715 -3.517096428 9.90E-40 9.50E-38

RIMBP2 0.746323902 0.098982726 -2.914553206 1.06E-39 1.01E-37

WAKMAR2 0.132084163 0.704163061 2.414452053 1.07E-39 1.02E-37

CHL1 6.325989149 0.617426192 -3.35695249 1.08E-39 1.02E-37

AL031123.1 10.58834317 0.906174057 -3.54654486 1.09E-39 1.03E-37

SIM2 5.444617059 0.239558702 -4.506379457 1.10E-39 1.03E-37

LRRC2 3.562984708 0.32798995 -3.441362775 1.12E-39 1.05E-37

SLC2A12 3.539748913 0.332891282 -3.410524036 1.12E-39 1.05E-37

ERMP1 45.6789176 8.080264609 -2.499054022 1.13E-39 1.05E-37

TCF21 10.25957853 0.917941382 -3.482425626 1.15E-39 1.07E-37

YY1P2 0.043192477 0.003797476 -3.507667153 1.16E-39 1.08E-37

HLA-B 290.8432708 1113.972107 1.937399279 1.20E-39 1.11E-37

SCARB1 2.450124358 34.56733916 3.818482674 1.22E-39 1.12E-37

SIAH3 1.244283022 0.037220064 -5.063090311 1.24E-39 1.14E-37

MTCP1 0.330582161 3.209401257 3.279223393 1.25E-39 1.15E-37

PAK6 0.170943908 0.018156126 -3.234994701 1.29E-39 1.18E-37

ALDH3B2 0.780699234 0.052817701 -3.885673454 1.33E-39 1.21E-37

KSR2 0.899367931 0.098993688 -3.183503 1.36E-39 1.24E-37

FER1L6 1.134488088 0.164301779 -2.787621455 1.37E-39 1.24E-37

NAT8L 11.04399282 0.394484636 -4.807148933 1.42E-39 1.28E-37

TYRP1 5.27942938 0.141356967 -5.222967112 1.50E-39 1.34E-37

ARHGAP24 25.16775225 8.285874178 -1.602850556 1.57E-39 1.39E-37

PSMB8 23.5900901 72.09384719 1.61169521 1.60E-39 1.42E-37

TNNI1 1.875642287 0.119198764 -3.975943533 1.71E-39 1.50E-37

ZPBP 0.072916969 0.010469892 -2.800007958 1.71E-39 1.50E-37

ITGAD 0.021203334 0.600487968 4.823772315 1.71E-39 1.50E-37

CLCNKA 71.58846046 1.768176081 -5.339393199 1.73E-39 1.51E-37

LINC01685 0.026700944 0.001729512 -3.948453779 1.75E-39 1.52E-37

COL4A4 11.2928144 2.604077625 -2.116560721 1.76E-39 1.52E-37

AC136621.1 0.04500598 0.012605567 -1.836055752 1.75E-39 1.52E-37

NPTX2 1.978962318 67.65095748 5.095294306 1.76E-39 1.52E-37

NLRC5 1.011753493 4.915886327 2.280593726 1.78E-39 1.53E-37

SDS 0.257938154 5.425802914 4.394739548 1.86E-39 1.59E-37

RAB11FIP4 3.896452251 1.002758543 -1.95818688 1.88E-39 1.61E-37

MAL 329.7434364 29.54487768 -3.480364011 1.91E-39 1.63E-37

AL158847.1 0.523949656 0.045472069 -3.526375637 1.92E-39 1.64E-37

PROM2 28.94209932 2.420898455 -3.5795551 1.99E-39 1.68E-37

CACNA2D2 1.648251247 0.340372415 -2.275750149 2.08E-39 1.76E-37

HLA-F-AS1 0.33338156 1.13960865 1.773292265 2.12E-39 1.79E-37

ST8SIA4 0.6926729 8.452596733 3.609148485 2.24E-39 1.89E-37

CHGB 7.505851206 0.56755816 -3.725175547 2.27E-39 1.90E-37

KCNK13 5.168706111 0.823880061 -2.649296941 2.35E-39 1.97E-37

NOS1AP 0.758354234 0.111360277 -2.767637192 2.44E-39 2.04E-37

AL353152.1 2.991248158 0.170473751 -4.133126088 2.50E-39 2.08E-37

CCSER1 1.167511879 0.218842165 -2.41547459 2.54E-39 2.10E-37

EPN3 5.188636457 0.331475622 -3.968380778 2.56E-39 2.11E-37

LINC01230 2.447812963 0.748823498 -1.708795714 2.60E-39 2.14E-37

CCDC181 2.103362587 0.261561039 -3.007478008 2.66E-39 2.18E-37

GAS6-AS1 0.111491808 2.010099004 4.172256946 2.68E-39 2.19E-37

LINC00602 0.109631688 0.014562143 -2.912370296 2.70E-39 2.20E-37

FAM167A 7.074714942 0.626412058 -3.49748813 2.73E-39 2.22E-37

COL26A1 4.210523056 0.156993271 -4.745224834 2.74E-39 2.22E-37

CFAP74 0.007027264 0.118414015 4.074732838 2.85E-39 2.31E-37

PDE1A 25.68797689 3.46947926 -2.888302225 2.89E-39 2.32E-37

FGFBP1 0.808438407 0.129361457 -2.64373003 2.90E-39 2.32E-37

AGAP2 0.173813083 1.13906475 2.712241179 3.03E-39 2.42E-37

FER1L4 0.059214027 2.789463836 5.557905057 3.06E-39 2.44E-37

AC007906.2 25.22155967 1.575783928 -4.000515862 3.09E-39 2.44E-37

FECH 25.27341774 6.544823334 -1.949194619 3.09E-39 2.44E-37

LINC01543 6.51959216 0.063067521 -6.691740678 3.21E-39 2.53E-37

TENT5D 0.039370001 0.004579325 -3.103890025 3.27E-39 2.57E-37

ADH1C 15.59172784 1.413523316 -3.463413227 3.42E-39 2.68E-37

MFSD4A 81.5697549 2.037774673 -5.322967878 3.52E-39 2.75E-37

ADGRF3 1.530784345 0.130491041 -3.552248384 3.59E-39 2.78E-37

LILRB1 0.386682366 2.985894924 2.948942517 3.59E-39 2.78E-37

PIK3R6 0.137901625 1.443178438 3.387538325 3.59E-39 2.78E-37

AKAP3 1.564919536 0.316153421 -2.307391743 3.62E-39 2.80E-37

AC104794.5 6.585028931 1.4649556 -2.168332839 3.65E-39 2.81E-37

FAM222A 5.420689728 0.751000875 -2.851589937 3.79E-39 2.91E-37

C1orf226 5.661988175 0.800242463 -2.822799647 3.90E-39 2.99E-37

COL4A5 6.233219764 1.269149205 -2.296115892 3.94E-39 3.00E-37

FAM171A1 34.25282844 7.709377679 -2.151536812 3.94E-39 3.00E-37

FAM81A 3.655935089 0.581940543 -2.651296791 4.01E-39 3.05E-37

AL031726.1 2.981812449 0.069165083 -5.430001803 4.13E-39 3.13E-37

S100A2 44.94602435 3.644959574 -3.62421879 4.13E-39 3.13E-37

LINC00645 7.724902255 0.404416369 -4.255603382 4.19E-39 3.16E-37

KLHL1 0.054572695 0.005888185 -3.212284451 4.22E-39 3.18E-37

AL161668.4 1.775058229 0.103734943 -4.096892498 4.24E-39 3.19E-37

CCDC185 0.115188662 0.016289595 -2.821976079 4.32E-39 3.22E-37

CXCR4 13.70918847 94.82623863 2.790143139 4.33E-39 3.22E-37

COBLL1 21.57975581 4.087285029 -2.40046378 4.53E-39 3.37E-37

GATA3 25.44814668 2.867507597 -3.149691379 4.57E-39 3.39E-37

F11 9.220758745 0.457322088 -4.333602963 4.62E-39 3.41E-37

CPAMD8 5.930039749 0.854294691 -2.795236053 4.62E-39 3.41E-37

CGN 13.68303333 2.540734984 -2.429070285 4.75E-39 3.49E-37

MARVELD2 11.71952297 2.21833284 -2.401366098 4.75E-39 3.49E-37

DOK3 0.498618026 3.108907502 2.640400747 5.02E-39 3.68E-37

TMEM155 0.017928872 0.655659625 5.19259043 5.20E-39 3.80E-37

PCDH9 0.852202078 0.092576793 -3.202473076 5.26E-39 3.84E-37

TAP1 9.824433889 37.89533981 1.947574261 5.31E-39 3.86E-37

SLC15A4 4.914226486 17.68454055 1.847452483 5.57E-39 4.04E-37

AVPR2 7.926016222 0.725834401 -3.44888358 5.62E-39 4.07E-37

MRAP2 2.947445291 0.286314374 -3.363793027 5.77E-39 4.17E-37

PPP2R2B 0.950009132 0.19622742 -2.275414726 5.89E-39 4.24E-37

AC010501.2 1.006588373 0.185455042 -2.440332446 6.04E-39 4.34E-37

AP003559.1 0.137751191 0.017248734 -2.997502433 6.07E-39 4.35E-37

CNTN1 5.280299988 0.422711713 -3.6428739 6.24E-39 4.45E-37

AC138207.5 0.884341435 4.936060112 2.480684572 6.34E-39 4.52E-37

AMPH 1.783234529 0.198144619 -3.169870765 6.58E-39 4.66E-37

AL606760.1 1.255715813 0.370449339 -1.761161833 6.58E-39 4.66E-37

HILPDA 4.372160292 112.6098692 4.686843168 6.58E-39 4.66E-37

STAC3 0.440972936 2.161548379 2.293303105 6.65E-39 4.69E-37

BX255923.1 0.281950474 0.04081517 -2.788264401 6.81E-39 4.79E-37

PTPN13 16.96199097 4.817310848 -1.816005596 6.83E-39 4.79E-37

AC078883.1 0.372244354 1.589411205 2.094170548 6.83E-39 4.79E-37

SRARP 0.642903537 0.068446216 -3.231559606 7.14E-39 4.99E-37

CCNI2 1.595631393 0.210784715 -2.920285254 7.43E-39 5.18E-37

TSPAN8 25.3138838 1.662685825 -3.928341378 7.50E-39 5.21E-37

SUSD4 10.24236959 0.79297523 -3.691129911 7.50E-39 5.21E-37

AC006963.2 0.518726586 0.108657989 -2.255180064 7.83E-39 5.42E-37

TNNC1 5.631231013 0.540819838 -3.380230359 7.86E-39 5.43E-37

LDHB 428.8609028 146.1951428 -1.552614419 8.55E-39 5.88E-37

HTRA4 0.026596515 0.773371221 4.861851933 8.95E-39 6.15E-37

LIPH 9.909148458 0.766007959 -3.693329798 9.04E-39 6.16E-37

FAM83B 3.061280484 0.266432183 -3.522294969 9.03E-39 6.16E-37

SORT1 37.95197806 13.23821091 -1.51946692 9.04E-39 6.16E-37

OACYLP 0.023599548 2.564611281 6.763837138 9.12E-39 6.20E-37

AL135999.3 1.545612011 0.458942028 -1.751794378 9.49E-39 6.44E-37

AL133372.2 0.037469353 0.005859655 -2.676823388 9.56E-39 6.48E-37

LINC01987 0.084048899 0.022717635 -1.887416245 9.68E-39 6.54E-37

GAL3ST3 3.218246006 0.155703104 -4.369404999 9.80E-39 6.60E-37

NAALADL2 1.875741896 0.504957008 -1.893228859 1.00E-38 6.71E-37

TNFRSF14 5.158948625 23.1763763 2.167506029 1.00E-38 6.71E-37

CADPS2 17.98879646 6.013922565 -1.580720469 1.01E-38 6.75E-37

TPPP2 0.490052857 0.026620419 -4.202332193 1.03E-38 6.88E-37

EPB41L4B 3.736502288 0.31595678 -3.563889277 1.09E-38 7.24E-37

ATP4B 0.479437534 0.070748819 -2.760564884 1.09E-38 7.24E-37

AC093523.2 0.16040187 0.017277747 -3.214703981 1.17E-38 7.72E-37

LINC02554 0.207526261 0.048322339 -2.102531726 1.17E-38 7.72E-37

OTOAP1 0.027380156 0.238963714 3.125589016 1.23E-38 8.09E-37

LZTS3 14.71820363 2.958505495 -2.31466112 1.26E-38 8.30E-37

AC016526.3 0.131388074 0.011326426 -3.536069694 1.27E-38 8.33E-37

OVOL2 1.245520458 0.1128671 -3.464051803 1.27E-38 8.33E-37

RASSF10 3.562484723 0.418156987 -3.090767252 1.32E-38 8.62E-37

FREM1 4.800607591 0.465917184 -3.365071566 1.35E-38 8.76E-37

PAQR5 24.64039447 7.270684679 -1.760862219 1.39E-38 8.97E-37

AC084759.1 0.110982718 0.00631255 -4.135968251 1.39E-38 8.97E-37

PTCSC3 4.18756292 0.349949276 -3.580893141 1.40E-38 9.04E-37

FAM169A 2.59635319 0.288669016 -3.168998478 1.41E-38 9.06E-37

CDCA2 0.30070131 5.890745275 4.292047113 1.41E-38 9.06E-37

PLPPR1 8.164899235 0.404576817 -4.334949524 1.44E-38 9.17E-37

ADGRF1 10.86227787 0.589864301 -4.202799767 1.44E-38 9.17E-37

LHX1 3.020999611 0.296317473 -3.349810393 1.49E-38 9.51E-37

FABP6 0.390912925 19.45977802 5.637504156 1.50E-38 9.52E-37

F11-AS1 2.07185343 0.10841946 -4.25622631 1.54E-38 9.74E-37

IDH2-DT 1.404985816 0.201796626 -2.799581607 1.61E-38 1.02E-36

PAPPA 4.588428132 0.526282964 -3.12408941 1.67E-38 1.05E-36

ERBB4 5.04758368 0.34427227 -3.873971037 1.75E-38 1.10E-36

SAP30 2.616934819 14.65200164 2.485147871 1.83E-38 1.15E-36

SRGAP3 1.62807294 0.296547477 -2.456830332 1.92E-38 1.20E-36

MYO3B 5.068082386 0.368135252 -3.783132167 1.94E-38 1.21E-36

EYA4 2.782415578 0.246837565 -3.49470404 1.95E-38 1.22E-36

GAL3ST4 0.732551153 5.532738686 2.916992377 1.95E-38 1.22E-36

SLC1A4 1.440338522 5.588999879 1.956182216 2.03E-38 1.26E-36

MPP7 10.34143214 1.38458039 -2.900915267 2.14E-38 1.33E-36

ARHGAP22 0.187419152 1.230492509 2.714895583 2.16E-38 1.34E-36

MAPK4 2.663208699 0.163049901 -4.029780013 2.19E-38 1.35E-36

ANKRD34B 0.477865948 0.032332825 -3.885534483 2.25E-38 1.38E-36

AC007993.1 0.236817856 0.025310822 -3.225951589 2.30E-38 1.41E-36

NIPAL1 4.809330513 0.496577127 -3.275746361 2.37E-38 1.45E-36

CA8 5.054649058 0.489813877 -3.367305378 2.44E-38 1.49E-36

NAV2-AS3 0.451974583 0.084780681 -2.414434189 2.48E-38 1.51E-36

FRG2B 0.220812148 0.010472662 -4.398119486 2.52E-38 1.52E-36

LINC00443 0.25692248 0.027671844 -3.214842418 2.52E-38 1.52E-36

AC006058.3 0.369920103 0.074289824 -2.315977194 2.59E-38 1.56E-36

LY86-AS1 0.099055487 0.028224868 -1.81127006 2.63E-38 1.59E-36

CALCA 8.634989161 1.194736171 -2.853502298 2.69E-38 1.61E-36

CGNL1 51.16667194 11.82991647 -2.112764511 2.70E-38 1.62E-36

C1orf127 0.017800424 0.161217638 3.179026105 2.80E-38 1.67E-36

ITGAX 0.539689098 5.246232732 3.281081361 2.83E-38 1.69E-36

AC008771.1 3.743810764 1.154918865 -1.69671601 2.86E-38 1.70E-36

SOX11 0.017447551 0.749261977 5.424373749 2.94E-38 1.74E-36

ACSL4 37.34497875 13.09191682 -1.512237936 2.99E-38 1.77E-36

NETO2 1.020722718 10.1677074 3.316331506 3.05E-38 1.80E-36

SPTB 0.891068136 0.146329619 -2.606313938 3.19E-38 1.88E-36

PRRG2 7.308763661 0.954691029 -2.936521576 3.22E-38 1.89E-36

SLC25A33 4.438026464 1.33797974 -1.729862 3.22E-38 1.89E-36

AC008125.1 0.054794044 0.00608963 -3.16959268 3.34E-38 1.96E-36

CAV2 6.180266861 24.30287911 1.975386197 3.44E-38 2.01E-36

AC104984.5 3.682573955 0.462925451 -2.99186271 3.54E-38 2.07E-36

AQP5 1.125694435 0.172471152 -2.706388289 3.63E-38 2.11E-36

SSC4D 3.103315003 0.626701394 -2.307960039 3.63E-38 2.11E-36

HOMER1 4.600688806 0.983313921 -2.226125903 3.77E-38 2.19E-36

UQCRFS1P2 0.110709633 0.036011533 -1.620249825 3.81E-38 2.20E-36

TAF1D 2.674472222 7.676171724 1.521132767 3.84E-38 2.21E-36

EGF 74.50253505 1.589919098 -5.550264254 3.88E-38 2.23E-36

FOXC1 21.0564401 4.6665463 -2.173834433 3.95E-38 2.26E-36

CD300A 1.121980594 8.980388604 3.000730152 3.95E-38 2.26E-36

LINC01943 0.084571732 0.766338206 3.179733805 4.25E-38 2.43E-36

STC2 1.485266486 23.58730573 3.989216928 4.29E-38 2.45E-36

C9orf135 1.000287232 0.090875265 -3.460382854 4.32E-38 2.46E-36

ABCA4 5.704451366 0.223627705 -4.672917303 4.49E-38 2.53E-36

HSD11B2 206.4807 27.00716632 -2.934592757 4.49E-38 2.53E-36

AGBL4 0.485238793 0.09154329 -2.40616885 4.49E-38 2.53E-36

ABHD17C 9.568681139 2.25346936 -2.086172256 4.45E-38 2.53E-36

LTB4R 0.519578019 3.04305765 2.550109361 4.49E-38 2.53E-36

PARVG 0.340106073 2.536222293 2.898624527 4.53E-38 2.55E-36

INSYN1-AS1 1.006203196 0.272172674 -1.886327545 4.63E-38 2.60E-36

PLS3-AS1 0.710487518 0.110789213 -2.680991888 4.88E-38 2.73E-36

C10orf71 0.116396419 0.037758295 -1.624181162 4.88E-38 2.73E-36

RNVU1-32 0.08387602 1.43105331 4.092675203 4.90E-38 2.74E-36

HLA-A 258.0919347 835.7994612 1.69527177 4.97E-38 2.77E-36

VTCN1 17.16196353 1.604722277 -3.418819081 5.20E-38 2.89E-36

AC137723.1 0.365298829 0.051909041 -2.815019379 5.20E-38 2.89E-36

SIM1 10.17692438 1.235908632 -3.041657625 5.30E-38 2.94E-36

CA9 2.243136175 136.0519951 5.922497097 5.35E-38 2.96E-36

INHBB 1.785995579 25.1763071 3.817266267 5.40E-38 2.98E-36

AC010655.2 0.069154883 2.866902601 5.373517969 5.41E-38 2.98E-36

RNF43 3.682605844 0.636477177 -2.532546304 5.50E-38 3.03E-36

AC004870.1 0.611465486 0.067075453 -3.188414267 5.61E-38 3.08E-36

SLC4A1 48.75202 4.206311193 -3.534834504 5.81E-38 3.18E-36

TNFAIP6 0.569658114 35.32574177 5.954479713 5.81E-38 3.18E-36

ZFAS1 14.12676968 45.20327922 1.677995828 5.87E-38 3.20E-36

C2orf15 2.981314942 0.819515768 -1.863105175 6.14E-38 3.35E-36

TMPRSS4 3.195945704 0.2410174 -3.729033687 6.20E-38 3.36E-36

SYT7 6.772723254 0.571535449 -3.566821157 6.20E-38 3.36E-36

NDUFA4L2 13.10351732 580.0550561 5.468163815 6.20E-38 3.36E-36

PLXDC1 0.79305821 5.88724404 2.892093764 6.26E-38 3.38E-36

ANK3-DT 0.916691313 0.077315839 -3.567600106 6.42E-38 3.45E-36

THRB 6.16222875 1.629610372 -1.91892517 6.43E-38 3.45E-36

DANCR 19.82004889 5.901720696 -1.74775297 6.43E-38 3.45E-36

AL158817.1 0.132303665 0.006598065 -4.325666225 6.56E-38 3.52E-36

ODF3B 1.017547711 11.73498163 3.527647236 6.74E-38 3.60E-36

L3MBTL4-AS1 0.056628053 0.412246474 2.863918322 6.77E-38 3.62E-36

LINC02568 1.679222575 0.177566721 -3.241360339 6.99E-38 3.71E-36

MPPED2 3.329367488 0.576930047 -2.528779814 6.99E-38 3.71E-36

TNFRSF4 0.570296188 5.561031829 3.285569301 6.99E-38 3.71E-36

DACH2 0.242486048 0.027297001 -3.151087376 7.23E-38 3.83E-36

NUPR2 7.460512792 1.344576663 -2.472122781 7.44E-38 3.93E-36

PLCL1 16.17935939 2.606743224 -2.633834103 7.59E-38 4.01E-36

LAD1 24.96643559 3.950002032 -2.660064565 7.81E-38 4.10E-36

CER1 0.088529287 0.012990774 -2.768667401 7.82E-38 4.10E-36

RASAL3 0.619171098 4.023561188 2.700062937 8.02E-38 4.20E-36

CACNA2D3 1.874950892 0.436264911 -2.103576463 8.17E-38 4.27E-36

CLCNKB 102.1311302 8.54066354 -3.579930706 8.32E-38 4.33E-36

AC083862.1 0.826666775 3.469092316 2.069180424 8.32E-38 4.33E-36

TACSTD2 177.3748228 15.51523219 -3.515044051 8.40E-38 4.36E-36

LMO3 1.48910374 0.14533913 -3.356949184 8.48E-38 4.40E-36

TARID 0.268642388 0.043277392 -2.634001498 8.95E-38 4.63E-36

ENTPD3 1.0429266 0.111807125 -3.221553591 9.12E-38 4.71E-36

MAGEE2 0.161231979 0.025102425 -2.683239266 9.19E-38 4.73E-36

NR0B2 12.87589489 1.486644849 -3.114540763 9.70E-38 4.96E-36

SLC12A3 73.30503179 2.99324404 -4.614130419 9.91E-38 5.05E-36

RELT 0.328711904 1.41369825 2.104578605 9.91E-38 5.05E-36

PIK3R5 0.361425004 2.584320304 2.838016668 1.00E-37 5.09E-36

MAP3K15 1.456948697 0.199355109 -2.86953759 1.01E-37 5.12E-36

CD300LF 0.324951005 2.637871036 3.021079917 1.03E-37 5.21E-36

PSMB9 5.288747181 24.81906221 2.230450688 1.07E-37 5.39E-36

MECOM-AS1 0.65665951 0.147381221 -2.155592794 1.07E-37 5.40E-36

RHBDF2 2.104422932 8.122403312 1.948481989 1.08E-37 5.43E-36

AP006333.1 2.268199999 0.126036994 -4.169628703 1.09E-37 5.46E-36

NDST3 0.282085663 0.034714623 -3.022517943 1.09E-37 5.50E-36

AC241644.2 1.403160831 0.132846593 -3.400847245 1.13E-37 5.66E-36

FCGR3A 5.324274978 54.78629213 3.363157979 1.15E-37 5.74E-36

LINC01975 0.256171182 0.06511633 -1.976016885 1.18E-37 5.89E-36

ZPLD2P 1.902548204 0.437114804 -2.121848862 1.20E-37 5.97E-36

ZFHX2 0.905388688 0.27375043 -1.72567605 1.20E-37 5.97E-36

FATE1 0.164149668 1.919932372 3.547971753 1.20E-37 5.97E-36

Z99572.1 1.84244905 0.321199044 -2.520085222 1.21E-37 6.01E-36

SHMT2 11.77514142 46.41954134 1.978987881 1.22E-37 6.06E-36

DOC2A 0.077140151 5.516229564 6.160056725 1.25E-37 6.16E-36

AC026369.2 0.439149282 5.670779166 3.690763625 1.27E-37 6.25E-36

MYOZ2 0.427984234 0.051489571 -3.055205492 1.30E-37 6.37E-36

GJA8 0.201561847 0.02511 -3.004888629 1.32E-37 6.47E-36

PNCK 0.692857538 24.79488998 5.16134027 1.34E-37 6.56E-36

TAGLN3 2.34926915 0.261931528 -3.16495038 1.35E-37 6.61E-36

SEMA6D 9.514390299 1.26975477 -2.905561315 1.35E-37 6.61E-36

NR3C2 11.14803619 2.762160559 -2.012920503 1.39E-37 6.78E-36

AC091965.1 0.680509334 0.125897372 -2.434366779 1.44E-37 7.01E-36

CASP17P 0.239359198 1.632083566 2.769465778 1.47E-37 7.14E-36

DGCR9 0.208328068 3.437848526 4.04457685 1.50E-37 7.25E-36

DARS-AS1 0.113055018 1.029901889 3.18740998 1.51E-37 7.30E-36

P2RX7 0.568997307 3.722196398 2.709660451 1.53E-37 7.34E-36

CTH 5.554299703 1.53365613 -1.856629981 1.54E-37 7.40E-36

NAP1L2 7.8309385 1.674522319 -2.225435612 1.57E-37 7.52E-36

GRM1 1.026526231 0.095565601 -3.425135278 1.58E-37 7.58E-36

AC110774.1 0.4926992 0.089958637 -2.453373416 1.60E-37 7.67E-36

AL137186.2 0.188834699 0.90609508 2.262538559 1.61E-37 7.70E-36

MAGI2 2.910392611 0.96474223 -1.592998361 1.66E-37 7.90E-36

AC007326.1 0.045200322 5.410881725 6.903386843 1.68E-37 7.99E-36

AC137834.2 0.010499838 0.46886002 5.48071834 1.70E-37 8.08E-36

AL512785.2 0.179743151 0.023811343 -2.916215921 1.72E-37 8.15E-36

FMO5 7.16317225 1.359321053 -2.397712393 1.80E-37 8.52E-36

MYH7 0.068998263 0.009791084 -2.81701959 1.81E-37 8.55E-36

SCNN1A 70.01062319 8.597278019 -3.025621983 1.81E-37 8.56E-36

LINC02611 0.089014179 0.65232491 2.873483652 1.83E-37 8.61E-36

FAM78A 0.573603294 3.025175948 2.398893839 1.88E-37 8.83E-36

YPEL4 0.09611978 0.604062004 2.651791398 1.90E-37 8.89E-36

BMP7 3.334839914 0.378357432 -3.139795818 1.94E-37 9.07E-36

GRIK3 0.091674858 3.17478796 5.11399031 1.95E-37 9.11E-36

CCDC160 4.88782461 0.94864699 -2.365249281 1.97E-37 9.17E-36

CORO2B 5.227825319 1.304071421 -2.003188051 1.97E-37 9.17E-36

HMGA2-AS1 0.334025751 0.03494577 -3.256769596 2.03E-37 9.40E-36

CMTM4 27.75082024 7.570558575 -1.874058759 2.03E-37 9.40E-36

PROX1 6.693235572 0.318916123 -4.391454849 2.08E-37 9.64E-36

TMPRSS2 21.22980094 1.59799953 -3.731751955 2.14E-37 9.89E-36

EVL 2.491277997 8.103930572 1.701735796 2.16E-37 9.97E-36

ILDR1 10.87732019 2.614332423 -2.056808667 2.18E-37 1.00E-35

SLC16A3 3.891058964 31.64135822 3.023576782 2.24E-37 1.03E-35

TFCP2L1 38.44710229 4.12019332 -3.222090933 2.26E-37 1.04E-35

GAS1RR 0.205677675 0.050460071 -2.027171079 2.28E-37 1.05E-35

SNHG12 0.598911894 4.772296704 2.994268052 2.39E-37 1.09E-35

FOLR3 3.915703364 0.365672528 -3.420647327 2.43E-37 1.11E-35

TNFRSF10B 7.282567361 20.89170975 1.520411519 2.66E-37 1.21E-35

CWH43 5.494279625 1.088503335 -2.335584509 2.69E-37 1.22E-35

VEGFA 10.79031619 125.7155178 3.542353694 2.71E-37 1.23E-35

AC007342.9 0.848837257 0.142547386 -2.574046397 2.75E-37 1.24E-35

SLC13A2 18.49248723 0.775387295 -4.575878374 2.76E-37 1.24E-35

ESM1 8.264372046 103.5572073 3.647378951 2.79E-37 1.25E-35

SCN2A 0.997240903 0.117666152 -3.083242689 2.81E-37 1.26E-35

AC007406.3 0.325605199 3.523161651 3.435675028 2.81E-37 1.26E-35

AL139125.1 0.044896302 0.424510114 3.24113039 2.84E-37 1.27E-35

RRS1-AS1 0.184080676 0.029200869 -2.656254993 2.85E-37 1.27E-35

TAPBP 27.74980472 84.23953798 1.602019905 2.87E-37 1.28E-35

ADAP2 1.960937588 7.322837378 1.900859139 2.89E-37 1.29E-35

RAB42 0.50168346 14.45848814 4.84899552 2.97E-37 1.32E-35

GABARAPL1 84.36010986 28.69902076 -1.555559462 3.00E-37 1.33E-35

FAM242C 0.70794897 0.07356712 -3.26651236 3.00E-37 1.33E-35

MAL2 94.40825665 13.64566843 -2.790469972 3.03E-37 1.34E-35

TRPM2 0.35163474 2.409790999 2.776758511 3.05E-37 1.35E-35

KCNQ1 22.97739263 6.04657698 -1.92602454 3.14E-37 1.38E-35

PSMB8-AS1 3.218310639 9.132174165 1.504654789 3.14E-37 1.38E-35

KCNB2 0.04750729 0.008411042 -2.49779241 3.18E-37 1.39E-35

ZNF503 18.83643025 4.936847516 -1.931863591 3.20E-37 1.40E-35

MAGI3 5.32290669 1.544040017 -1.785504132 3.38E-37 1.48E-35

EDA2R 1.111160688 5.478233544 2.301643308 3.41E-37 1.49E-35

AC093840.1 0.498575275 0.070425184 -2.823648002 3.43E-37 1.50E-35

AC114803.1 0.088562714 2.628324025 4.891299902 3.59E-37 1.56E-35

SLC4A9 5.238483251 0.541384469 -3.274423749 3.61E-37 1.57E-35

SLC4A11 13.40310863 0.575731736 -4.5410271 3.70E-37 1.60E-35

CD68 0.22119933 1.935416552 3.129225182 3.70E-37 1.60E-35

AQP7P3 0.069190919 0.003335858 -4.374452898 3.78E-37 1.63E-35

SYP 1.469080383 0.496358527 -1.565458855 3.84E-37 1.65E-35

SLC16A5 22.0304321 2.609375861 -3.077721118 3.94E-37 1.69E-35

MLKL 1.190642764 4.617439406 1.955352412 3.94E-37 1.69E-35

C5orf46 0.444219216 13.49408672 4.924911725 4.00E-37 1.71E-35

DDX25 0.31096664 0.058874052 -2.401055984 4.01E-37 1.71E-35

WASF3 5.221801861 1.784134941 -1.549322981 4.05E-37 1.73E-35

KRBA1 1.820098521 12.81021815 2.815206595 4.16E-37 1.77E-35

DGKD 1.214062043 5.383360917 2.148665001 4.24E-37 1.80E-35

GNLY 0.490162003 4.338098701 3.145732321 4.28E-37 1.82E-35

RAB25 24.559332 1.952179182 -3.653113938 4.41E-37 1.87E-35

NEXMIF 0.826486109 0.109046004 -2.922053668 4.48E-37 1.89E-35

IL2RB 0.763775293 6.259662302 3.034864672 4.52E-37 1.91E-35

RPRM 1.424327524 0.141879374 -3.327544156 4.57E-37 1.92E-35

CASZ1 1.809800976 0.449121786 -2.010652441 4.64E-37 1.95E-35

KDF1 8.565026236 2.013979033 -2.088409001 4.69E-37 1.97E-35

TYMP 4.304598149 33.13173079 2.944263104 4.69E-37 1.97E-35

COLGALT1 8.855732306 29.15959154 1.719286993 4.73E-37 1.98E-35

CHP2 0.935822345 0.025105786 -5.220142886 4.84E-37 2.03E-35

SMIM5 7.909648513 0.765626949 -3.368900069 4.86E-37 2.03E-35

VIM 109.0103189 639.5199219 2.552524595 4.86E-37 2.03E-35

LINC02381 30.88938061 6.50874254 -2.246660189 4.90E-37 2.04E-35

ADM 12.07183515 80.42273438 2.735958378 4.95E-37 2.06E-35

AC019069.1 0.206618079 2.132048327 3.367201737 4.95E-37 2.06E-35

LINC00299 0.009166825 0.168756455 4.202376685 5.08E-37 2.11E-35

IFNA14 0.088492234 0.001846325 -5.582822314 5.11E-37 2.12E-35

AC069209.1 0.055201966 0.479997948 3.120236695 5.11E-37 2.12E-35

FABP7 1.387057525 84.19427879 5.923622676 5.27E-37 2.18E-35

ST6GAL1 62.30450014 13.3692088 -2.220422282 5.32E-37 2.20E-35

MIR4435-2HG 1.056913754 4.043424605 1.935720055 5.52E-37 2.27E-35

HOXB9 24.15763419 2.374513435 -3.346775351 5.57E-37 2.29E-35

MFSD6L 2.58066242 0.221457737 -3.542638129 5.62E-37 2.30E-35

CLEC2D 0.407064292 2.439841623 2.583458925 5.72E-37 2.34E-35

PPP1R1B 1.593889782 0.111119008 -3.842374342 5.75E-37 2.35E-35

SLC6A2 0.119809026 0.039380168 -1.60519542 5.81E-37 2.37E-35

DPEP2 0.361325213 1.798371289 2.315321076 5.88E-37 2.40E-35

WSCD2 0.783080446 0.160998506 -2.282113219 5.93E-37 2.41E-35

IGSF6 0.972910704 6.311128988 2.697518807 5.94E-37 2.41E-35

PCDHB1 0.253051316 0.037150299 -2.767984247 6.00E-37 2.43E-35

BTN3A1 4.190421903 15.89480638 1.923388032 6.04E-37 2.44E-35

MYEOV 0.056720445 7.543812285 7.055281122 6.10E-37 2.46E-35

CLDN14 3.549157783 0.457722301 -2.954932223 6.21E-37 2.51E-35

IL20RB 0.672176993 10.24814299 3.930377536 6.27E-37 2.53E-35

SIGLEC10 0.588047027 4.743193466 3.011855275 6.32E-37 2.54E-35

PHYHD1 13.34341421 2.781405091 -2.262242076 6.44E-37 2.58E-35

HS3ST2 0.088621338 2.57976382 4.863441071 6.44E-37 2.58E-35

TJP3 4.784781764 0.648501051 -2.883272307 6.62E-37 2.64E-35

LINC01094 0.277250445 1.963631265 2.824262361 6.62E-37 2.64E-35

OSTM1-AS1 0.004165584 2.009911219 8.914397199 6.74E-37 2.68E-35

SLC25A5-AS1 1.906289936 0.465586068 -2.033647768 7.05E-37 2.80E-35

PCCB 12.71099814 3.964180799 -1.680982655 7.18E-37 2.85E-35

LYPLAL1-DT 1.191361415 0.230203997 -2.371626348 7.24E-37 2.86E-35

RASD1 123.4203774 20.70849255 -2.575286168 7.31E-37 2.89E-35

AC104667.1 0.482360165 0.070754332 -2.769220388 7.68E-37 3.03E-35

LINC02332 0.660960067 0.082216886 -3.007056467 7.70E-37 3.03E-35

AC112198.2 0.331202477 0.054984106 -2.590626903 7.72E-37 3.03E-35

RAP1GAP 54.51348382 8.597448778 -2.664632602 7.93E-37 3.11E-35

PLPP4 3.884623211 0.630034783 -2.624271286 8.00E-37 3.14E-35

NPY5R 1.490346287 0.283573824 -2.393851313 8.53E-37 3.33E-35

MIR210HG 1.204069344 11.55013316 3.261919097 8.61E-37 3.36E-35

TCF24 0.469878807 0.015628419 -4.910044979 8.74E-37 3.41E-35

ENO2 5.282017392 47.76765384 3.176873062 8.85E-37 3.44E-35

CCDC88B 0.381502054 2.277299562 2.57756135 8.93E-37 3.47E-35

ATP8B3 0.162560979 1.714730632 3.39892906 9.01E-37 3.49E-35

C6orf52 1.326797647 0.374138231 -1.82630506 9.09E-37 3.51E-35

SLC25A35 3.426801074 1.130561837 -1.599822542 9.09E-37 3.51E-35

SOSTDC1 15.92105641 2.23066456 -2.835390579 9.17E-37 3.54E-35

TMEM72 62.32633997 7.618779573 -3.032210174 9.26E-37 3.56E-35

SLC26A7 16.02201235 3.761266146 -2.09076506 9.26E-37 3.56E-35

LYPD6B 3.542795703 0.472465929 -2.906606074 9.34E-37 3.59E-35

GOLGA7B 0.058314108 0.810004702 3.796013417 9.34E-37 3.59E-35

AC139769.1 2.007245099 0.479426569 -2.065835022 9.51E-37 3.64E-35

BCAM 151.2481679 46.54557323 -1.700201797 9.51E-37 3.64E-35

C1QTNF6 0.693557922 3.347356726 2.270934028 9.60E-37 3.67E-35

OXGR1 2.743162946 0.375384016 -2.869401197 9.61E-37 3.67E-35

WNT7A 0.408052472 0.051959419 -2.973297473 9.66E-37 3.68E-35

AC007342.4 1.316497929 0.173546817 -2.923308443 9.65E-37 3.68E-35

SLC9A2 4.189831609 0.463075474 -3.177573007 9.79E-37 3.73E-35

OPHN1 3.431152375 1.086067134 -1.659579912 9.86E-37 3.74E-35

TRPV2 1.881719088 7.448069868 1.984815335 9.86E-37 3.74E-35

CAND2 1.834939486 0.525710641 -1.803391644 1.01E-36 3.84E-35

OLFM4 7.463201099 0.545578076 -3.773936983 1.02E-36 3.85E-35

SLFN13 1.118439989 5.096339485 2.187973535 1.02E-36 3.86E-35

GRHL2 4.631645056 0.333207364 -3.797032505 1.05E-36 3.96E-35

AHNAK2 0.450615143 7.55171597 4.066836801 1.05E-36 3.96E-35

MAN1C1 7.811620074 1.671384156 -2.224578419 1.08E-36 4.05E-35

LINC02303 2.518065288 0.642811841 -1.96984728 1.08E-36 4.05E-35

PHYKPL 2.974751264 9.525280338 1.678992512 1.08E-36 4.05E-35

SEMA3B-AS1 6.09514734 0.821448215 -2.891419562 1.08E-36 4.06E-35

TMIGD3 0.498164241 3.882011731 2.962111105 1.11E-36 4.15E-35

ARMC4P1 0.493230887 0.048330117 -3.351268755 1.12E-36 4.20E-35

RHCG 62.55005826 20.79701356 -1.588634855 1.13E-36 4.22E-35

AL928921.1 0.665300456 0.229312225 -1.536692752 1.13E-36 4.23E-35

TDRD5 0.682013743 0.096254854 -2.824869613 1.16E-36 4.32E-35

OVOL1 3.546528335 0.689473556 -2.362840349 1.18E-36 4.39E-35

OTOA 0.016217249 0.124983699 2.946138973 1.18E-36 4.39E-35

PLA2G7 0.377565883 4.702069367 3.638495509 1.20E-36 4.46E-35

CD72 0.362359888 2.408438665 2.732603018 1.21E-36 4.50E-35

LINC01896 1.292712161 0.279952006 -2.207149655 1.22E-36 4.51E-35

ACOT11 4.084562972 1.04262638 -1.969959458 1.24E-36 4.55E-35

CHN2 2.039346169 0.69482304 -1.553389188 1.24E-36 4.55E-35

CORO7 0.567474408 1.642614053 1.533366309 1.24E-36 4.55E-35

SAMD12 3.19535639 0.881558896 -1.857847987 1.26E-36 4.63E-35

PSORS1C1 0.154431672 1.203787813 2.962540547 1.27E-36 4.67E-35

LINC01213 0.634512162 0.210699821 -1.590458729 1.28E-36 4.70E-35

MYO1F 0.810584683 4.694881575 2.534053948 1.29E-36 4.74E-35

TMEM213 69.45706511 7.87924815 -3.139991574 1.31E-36 4.81E-35

AC009084.2 0.040651844 2.242469683 5.785623888 1.35E-36 4.94E-35

B4GALNT3 5.299483011 1.053523503 -2.330629125 1.37E-36 4.98E-35

IL12RB1 0.281273986 1.74229676 2.63094234 1.39E-36 5.06E-35

PDP2 4.253299083 1.498487312 -1.505075438 1.43E-36 5.19E-35

TYMS 2.853996469 12.37620179 2.11651317 1.43E-36 5.19E-35

PLEKHD1 0.482511533 0.075650192 -2.673147777 1.44E-36 5.23E-35

LAPTM5 17.44166918 101.6070442 2.542390406 1.45E-36 5.27E-35

LINC02783 0.007711228 1.502525555 7.606213181 1.46E-36 5.29E-35

SUCLG1 64.30233056 19.33853684 -1.733392383 1.47E-36 5.30E-35

LINC01785 0.330986493 0.045760451 -2.85459918 1.47E-36 5.31E-35

LILRB4 0.450543951 3.973962832 3.140838624 1.48E-36 5.34E-35

LINC01561 0.351950162 0.043527442 -3.015374008 1.48E-36 5.34E-35

DNER 9.593789833 1.542210449 -2.637101188 1.49E-36 5.36E-35

SSTR5-AS1 0.928594314 0.060590064 -3.937895312 1.50E-36 5.37E-35

AC103563.7 5.17369644 0.597977582 -3.113032104 1.51E-36 5.38E-35

AL035661.1 29.66427933 2.555782522 -3.53688975 1.54E-36 5.51E-35

PRSS22 2.439815783 0.244630086 -3.31809847 1.54E-36 5.51E-35

NKG7 2.946645176 33.89984614 3.524133473 1.55E-36 5.53E-35

FGF9 9.149162983 1.075261352 -3.088952401 1.56E-36 5.56E-35

AP000757.1 8.871118132 1.183177989 -2.906448837 1.56E-36 5.56E-35

LAMA4 2.583336629 16.39142372 2.665633613 1.56E-36 5.56E-35

ANGPTL4 11.07169999 340.8253171 4.944083844 1.58E-36 5.60E-35

CAPSL 0.604011363 0.082385987 -2.874104806 1.59E-36 5.63E-35

MCF2L-AS1 2.411878111 0.498237863 -2.275250433 1.59E-36 5.63E-35

STAMBPL1 0.455687776 2.585089948 2.504096903 1.59E-36 5.63E-35

AL139275.2 0.793856698 0.141351525 -2.489591152 1.60E-36 5.66E-35

LINC00551 1.443060012 0.125846744 -3.519391497 1.62E-36 5.71E-35

TYROBP 15.28945163 96.42195363 2.656824997 1.62E-36 5.71E-35

VWA7 4.781047306 1.634967343 -1.548064861 1.65E-36 5.80E-35

RAPGEF3 7.533435167 2.285044075 -1.721085876 1.67E-36 5.85E-35

NKD1 0.944592378 0.129040163 -2.871871726 1.68E-36 5.88E-35

LINC00885 0.746540432 0.157909883 -2.241118931 1.68E-36 5.88E-35

TTC21B-AS1 0.037142902 13.27978033 8.481929016 1.68E-36 5.88E-35

CYP2B6 4.553810907 0.187378102 -4.603050117 1.70E-36 5.94E-35

ESRP1 7.651884918 0.827468709 -3.209038514 1.72E-36 6.00E-35

KLRD1 0.084454012 0.438419828 2.37607518 1.76E-36 6.13E-35

NXPH4 0.631326212 11.81941047 4.226628618 1.77E-36 6.17E-35

LCP2 1.60631535 7.624742511 2.246933469 1.79E-36 6.22E-35

VWA5B1 0.702445221 0.193473568 -1.860249244 1.81E-36 6.28E-35

RN7SL138P 0.738028744 3.747057668 2.344009269 1.82E-36 6.31E-35

AC107220.1 0.237977067 0.002360071 -6.655848298 1.83E-36 6.32E-35

BMPR1B 6.925853413 1.525887457 -2.182343294 1.89E-36 6.52E-35

LINC00271 0.905164494 0.280097088 -1.692253011 1.89E-36 6.52E-35

IKBIP 3.95600775 13.7519296 1.797516907 1.92E-36 6.64E-35

SLC6A17 1.308381495 0.187985077 -2.799093218 2.00E-36 6.85E-35

PPM1K 5.446468014 1.921652678 -1.502973353 2.00E-36 6.85E-35

PRDM1 1.253641832 6.253365249 2.318507559 2.00E-36 6.85E-35

LINC00380 0.445972282 0.042274393 -3.399098099 2.00E-36 6.86E-35

NUDT10 1.582994853 0.253464574 -2.64280054 2.03E-36 6.94E-35

RDH10 25.37267486 7.968975777 -1.670809403 2.05E-36 7.00E-35

ITLN1 2.467006215 0.196633673 -3.649179045 2.05E-36 7.00E-35

PLA2G4F 6.118355944 1.057549357 -2.532419044 2.09E-36 7.11E-35

TDGF1 4.941875346 0.500182846 -3.304531135 2.11E-36 7.17E-35

ATP6V1C2 7.384937193 1.767208491 -2.063113396 2.16E-36 7.34E-35

AC123023.1 0.105994921 0.014850982 -2.835364888 2.19E-36 7.41E-35

GCGR 5.430422043 0.7829299 -2.79410928 2.20E-36 7.43E-35

DUSP26 1.479492803 0.232901021 -2.667313809 2.26E-36 7.61E-35

BTC 4.092454764 1.157579386 -1.821855334 2.26E-36 7.62E-35

HS6ST1 33.10929818 7.272424258 -2.186728159 2.30E-36 7.74E-35

FCER1G 9.167727556 53.05744517 2.532919132 2.30E-36 7.74E-35

TDRD1 0.508937205 0.125978301 -2.014312407 2.35E-36 7.89E-35

GLDC 16.88547622 3.381029429 -2.320248389 2.37E-36 7.92E-35

HK2 1.589258743 17.0547609 3.423748599 2.37E-36 7.92E-35

AC011239.1 0.207929902 0.034410606 -2.595172025 2.39E-36 7.97E-35

RHBG 5.482258416 1.582699103 -1.792383332 2.40E-36 8.01E-35

IDO1 1.222919186 12.36748266 3.338150901 2.43E-36 8.10E-35

GAS1 6.467211603 1.508430201 -2.100095874 2.50E-36 8.31E-35

RASD2 0.602515363 4.563982581 2.921223353 2.57E-36 8.52E-35

TMEM30B 16.97602308 2.146878929 -2.983185783 2.64E-36 8.72E-35

MYORG 9.071014431 3.200948652 -1.502764365 2.64E-36 8.72E-35

FCGR1A 0.358387101 3.044816572 3.0867647 2.66E-36 8.79E-35

TCEAL2 6.656771994 0.792506831 -3.070327479 2.69E-36 8.88E-35

BSPRY 11.76628496 2.661294087 -2.144459031 2.73E-36 9.00E-35

LINP1 0.165611812 2.033279224 3.617930871 2.73E-36 9.00E-35

L1CAM 21.7755553 2.402221388 -3.180268489 2.76E-36 9.07E-35

ISG20 0.41655104 2.125562903 2.351279771 2.86E-36 9.36E-35

ZDHHC2 18.55405651 5.227870612 -1.827439301 2.89E-36 9.44E-35

ADAMTS7 0.340721421 1.895045366 2.475567829 2.91E-36 9.51E-35

LAIR1 0.892522766 5.657179956 2.664122191 2.97E-36 9.66E-35

DGCR5 0.418540462 11.01395087 4.717821168 2.97E-36 9.66E-35

AL356740.1 0.896657788 0.167994777 -2.416141103 3.00E-36 9.76E-35

LINC02048 0.02410485 1.285380424 5.736728126 3.02E-36 9.82E-35

C7 197.4646453 20.64507448 -3.257724844 3.10E-36 1.01E-34

ARL4D 33.24288156 4.751545976 -2.806576549 3.10E-36 1.01E-34

MAP3K9 1.292951718 0.409639468 -1.658241774 3.13E-36 1.01E-34

LPCAT1 7.316948347 42.07852589 2.523770185 3.13E-36 1.01E-34

RGS19 2.453337944 8.046626505 1.713638093 3.22E-36 1.04E-34

TMEM91 1.316735075 17.13261506 3.701708364 3.30E-36 1.06E-34

LUCAT1 0.03662434 1.489655172 5.34603183 3.36E-36 1.08E-34

GAS2L3 0.7374928 6.660265313 3.174878779 3.49E-36 1.12E-34

CLSTN2 4.477918213 0.906867522 -2.303864461 3.52E-36 1.12E-34

GADL1 0.482559229 0.022603521 -4.416086618 3.59E-36 1.15E-34

PCP4 17.94134801 3.034350321 -2.563828729 3.64E-36 1.16E-34

MYH10 31.39399342 9.501357385 -1.724283016 3.65E-36 1.16E-34

RHEBL1 0.216199959 0.831478964 1.943313517 3.65E-36 1.16E-34

APOBEC3H 0.215014276 1.599652516 2.895254191 3.68E-36 1.17E-34

AC007342.5 0.902256864 0.155843806 -2.533437397 3.76E-36 1.19E-34

TUBB8P7 0.291277834 0.030652665 -3.248311516 3.93E-36 1.25E-34

ANGPTL1 7.418793986 1.196912248 -2.631867294 3.95E-36 1.25E-34

CD1D 0.860631723 4.193998054 2.284858267 3.95E-36 1.25E-34

INPP5J 12.69138305 3.056462871 -2.05391435 3.99E-36 1.26E-34

GALNT3 8.438114076 1.431968301 -2.558921035 4.02E-36 1.27E-34

RBP2 2.037824387 0.139397015 -3.869758154 4.08E-36 1.29E-34

ROS1 0.440319808 0.076429539 -2.526349514 4.11E-36 1.29E-34

LINC01077 0.195286124 6.408140665 5.03624447 4.11E-36 1.29E-34

C15orf56 0.905764523 0.096310364 -3.233373072 4.24E-36 1.33E-34

SLC37A2 0.485046509 3.289541516 2.761691526 4.29E-36 1.34E-34

CNTN5 0.187642598 0.032800104 -2.516215075 4.31E-36 1.35E-34

RPEP6 0.030249434 0.009741466 -1.634697381 4.38E-36 1.37E-34

LINC01616 0.015621342 0.00208577 -2.904866214 4.40E-36 1.38E-34

NUDT4 14.57659069 4.183296724 -1.800941091 4.48E-36 1.40E-34

MS4A14 0.119300338 1.1070036 3.213989882 4.52E-36 1.41E-34

AC130371.2 4.471458525 0.744195501 -2.58699192 4.69E-36 1.46E-34

EZH2 0.504125022 1.857130958 1.881222083 4.69E-36 1.46E-34

PALD1 2.068786331 8.220696736 1.990476025 4.77E-36 1.48E-34

TMEM52B 64.95063176 1.942802689 -5.063132265 4.86E-36 1.51E-34

DLX5 0.13884758 1.46357556 3.397923289 4.90E-36 1.52E-34

CSF3R 0.361273153 2.497306914 2.789211182 4.99E-36 1.55E-34

LGALS9 2.135658654 9.548851391 2.160646128 5.08E-36 1.57E-34

CYFIP2 87.888915 18.27109236 -2.266118327 5.17E-36 1.60E-34

GRIK2 0.354205716 0.053510491 -2.72669381 5.22E-36 1.61E-34

AL049555.1 3.540425111 0.370257527 -3.257321632 5.29E-36 1.63E-34

OSCAR 0.427156025 2.537181639 2.570391769 5.36E-36 1.65E-34

C10orf82 0.576756314 0.08049668 -2.840960708 5.49E-36 1.69E-34

AL109615.1 0.054547336 7.016056543 7.007007833 5.72E-36 1.76E-34

IFFO1 1.449382218 5.079429094 1.809228254 5.76E-36 1.76E-34

RNASET2 3.348983639 33.49208626 3.322025013 5.76E-36 1.76E-34

AC020978.4 0.763127212 0.190394738 -2.002929968 5.80E-36 1.77E-34

LINC01544 0.180759875 0.028046106 -2.688202107 5.89E-36 1.80E-34

HOXB-AS3 5.586956483 0.940967277 -2.569846125 6.02E-36 1.83E-34

LINC01587 0.25374638 2.348123509 3.21004915 6.02E-36 1.83E-34

REEP6 9.289523868 1.158690633 -3.003109231 6.13E-36 1.86E-34

RFX8 0.05793485 0.649467406 3.48675378 6.13E-36 1.86E-34

CD40 6.298331111 22.51214435 1.837661976 6.24E-36 1.89E-34

PTHLH 0.312913128 14.43224251 5.527389487 6.30E-36 1.91E-34

TTPA 0.582520748 0.054762883 -3.411039126 6.32E-36 1.91E-34

FBXL8 0.709039438 2.693148207 1.925355846 6.47E-36 1.95E-34

LINC00487 0.01152573 0.184015382 3.996896279 6.47E-36 1.95E-34

LYPD6 1.175642684 0.178839566 -2.716711788 6.53E-36 1.97E-34

PLEKHB1 6.76544531 1.277988454 -2.404310095 6.65E-36 2.00E-34

ADGRV1 0.80948881 0.125403046 -2.690438748 6.71E-36 2.01E-34

LRP1B 0.228234836 0.040594284 -2.491170492 6.86E-36 2.06E-34

CLEC2B 1.543810914 7.493377704 2.27912011 7.08E-36 2.12E-34

HOXB1 0.101865957 0.023603355 -2.109608117 7.17E-36 2.14E-34

AC107021.2 0.347370031 2.442888311 2.81404271 7.27E-36 2.17E-34

MXD3 0.167789047 0.994298896 2.567031061 7.34E-36 2.19E-34

SELENBP1 34.06001181 10.23236685 -1.734939042 7.53E-36 2.24E-34

TMEM61 8.100909502 0.931356391 -3.120678653 7.55E-36 2.24E-34

ATP6V1B1 54.5326197 6.96854969 -2.968189127 7.60E-36 2.26E-34

DTX1 5.941631389 1.060120694 -2.48663058 7.74E-36 2.30E-34

PCED1B-AS1 0.477538906 3.126144024 2.710694064 7.88E-36 2.33E-34

MBOAT2 6.588774389 2.229418146 -1.563342896 8.02E-36 2.37E-34

ATP6V0A4 54.10487869 10.95331979 -2.304390493 8.07E-36 2.39E-34

MMP25-AS1 0.31537941 1.680744971 2.413940454 8.09E-36 2.39E-34

AC007255.1 0.589217771 0.115835559 -2.34672275 8.31E-36 2.45E-34

MYCNUT 0.051401997 0.013717824 -1.905772754 8.48E-36 2.50E-34

CENPM 0.391057447 1.765151577 2.174339612 8.69E-36 2.56E-34

SCN2B 0.525410486 0.113207619 -2.214473936 8.89E-36 2.61E-34

TBXAS1 1.432715124 5.04850144 1.817103435 8.93E-36 2.62E-34

CD70 1.815810682 36.13037075 4.31452636 9.13E-36 2.67E-34

EGLN3 4.107289625 79.23944973 4.269960274 9.17E-36 2.68E-34

NNMT 18.31632946 268.4626517 3.87351907 9.25E-36 2.70E-34

ZNF395 9.711918181 61.92087754 2.672597745 9.34E-36 2.72E-34

NAV2 6.161523861 2.043306737 -1.592381406 9.42E-36 2.74E-34

ANGPT2 2.2648326 19.27621162 3.089345219 9.42E-36 2.74E-34

TRIB3 2.151184735 18.04057482 3.06804198 9.85E-36 2.86E-34

AL359736.1 0.02694306 0.003515091 -2.938279588 9.89E-36 2.87E-34

ADGRE4P 0.022553551 0.416372826 4.206449401 1.01E-35 2.91E-34

SLC6A4 0.261975866 0.070643452 -1.890806167 1.03E-35 2.97E-34

OCLN 5.049051958 1.205095843 -2.066864634 1.05E-35 3.02E-34

AC025271.4 0.866756792 0.178520735 -2.279535586 1.05E-35 3.03E-34

SLC16A1 4.252015376 18.25550476 2.102112841 1.06E-35 3.04E-34

KLHL6 0.219051042 1.392989421 2.668845319 1.07E-35 3.06E-34

AL512383.1 0.134231983 0.017843097 -2.911290538 1.08E-35 3.11E-34

HLX 0.745428161 4.359822848 2.548128287 1.10E-35 3.13E-34

HIF1A-AS3 0.328371353 6.148215444 4.226767543 1.10E-35 3.13E-34

TNNT2 2.292270072 0.085305232 -4.747998989 1.11E-35 3.18E-34

AL391121.1 4.156416625 1.432769015 -1.536534229 1.12E-35 3.18E-34

RALGPS1 2.674809842 0.69178597 -1.951038672 1.14E-35 3.23E-34

RPL17P50 1.064843056 3.543465949 1.734520375 1.14E-35 3.23E-34

ITGA6-AS1 0.155973861 1.231354903 2.980870456 1.15E-35 3.25E-34

DUSP5P2 0.207842289 0.020109834 -3.369516114 1.16E-35 3.29E-34

FGF10 0.347601919 0.03076893 -3.497889862 1.16E-35 3.30E-34

CDH3 4.301000457 0.906115368 -2.246905631 1.17E-35 3.30E-34

FCGR1B 0.069804065 0.506991688 2.860579146 1.18E-35 3.33E-34

LINC01378 0.239012008 0.002648587 -6.495716134 1.18E-35 3.34E-34

BHLHE41 5.679504139 38.34068989 2.755039414 1.19E-35 3.35E-34

LDC1P 0.109460962 0.025242183 -2.116507876 1.20E-35 3.37E-34

AC090857.2 0.025571117 0.002417417 -3.402977156 1.21E-35 3.40E-34

AL136376.1 0.139949588 0.016148718 -3.115415673 1.23E-35 3.45E-34

RBM11 1.038053208 0.176092954 -2.559471306 1.25E-35 3.52E-34

GRIK5 3.975263275 0.47426275 -3.067291946 1.26E-35 3.54E-34

WFIKKN2 0.124672618 0.043532311 -1.517986133 1.26E-35 3.54E-34

PYGL 3.956743111 16.08835989 2.023631952 1.28E-35 3.57E-34

ACTRT3 2.281913744 0.660792448 -1.787975156 1.29E-35 3.60E-34

ANKRD2 10.63591439 1.067385606 -3.316790703 1.30E-35 3.62E-34

MBOAT4 0.062224595 0.334017476 2.424366738 1.36E-35 3.79E-34

CDT1 0.380483511 1.822894458 2.260325197 1.37E-35 3.81E-34

AC126323.1 0.086026447 0.016866782 -2.350595539 1.37E-35 3.82E-34

KCTD1 6.309072724 1.589934088 -1.988461022 1.39E-35 3.87E-34

DMRT2 11.78343835 3.130501005 -1.912295102 1.40E-35 3.88E-34

LINC02058 0.081490348 0.01011734 -3.009799142 1.41E-35 3.90E-34

TMCC1 3.925239806 13.59209978 1.791915648 1.41E-35 3.90E-34

C1orf116 6.549097431 0.887432857 -2.88358622 1.43E-35 3.95E-34

VASH1 2.009656497 9.254050724 2.203136079 1.43E-35 3.95E-34

TROAP 0.122923936 0.853685216 2.795938327 1.47E-35 4.06E-34

TMSB10 602.5835472 2548.010179 2.080137853 1.48E-35 4.09E-34

THSD4 5.258344167 1.327032486 -1.986404883 1.57E-35 4.30E-34

AJM1 0.428488755 1.840859533 2.103050298 1.59E-35 4.37E-34

PHKA2 3.605396111 16.81318925 2.221363735 1.61E-35 4.40E-34

AP001033.3 0.99297916 0.249925188 -1.990267134 1.63E-35 4.46E-34

PGF 1.895384371 29.09362359 3.940140642 1.71E-35 4.67E-34

SLC16A7 9.963696342 2.461580264 -2.017096272 1.74E-35 4.75E-34

KCNG3 0.122161458 0.035942862 -1.765012014 1.75E-35 4.77E-34

SPI1 3.241952369 16.63906764 2.359639795 1.76E-35 4.78E-34

TUBB2B 6.639621743 1.075469071 -2.626135018 1.77E-35 4.82E-34

ATP6V0D2 42.30824335 14.59175086 -1.535785783 1.79E-35 4.85E-34

SLC7A8 56.28882894 4.899947328 -3.522010488 1.81E-35 4.89E-34

TRG-AS1 0.110213554 0.712890984 2.693379825 1.82E-35 4.93E-34

PKMYT1 0.087302557 0.527427126 2.594875966 1.84E-35 4.97E-34

SHISA2 4.035382526 0.752719941 -2.422520341 1.87E-35 5.05E-34

SIX4 0.910923261 0.186472412 -2.288367316 1.89E-35 5.09E-34

APOBEC3G 1.122554896 5.688964697 2.34138013 1.89E-35 5.09E-34

FYB2 2.458147796 0.532220499 -2.207475676 1.90E-35 5.13E-34

SCN1B 1.072960089 4.328219516 2.012177258 1.92E-35 5.17E-34

AC073611.1 0.303039913 1.439374415 2.24786219 1.96E-35 5.25E-34

AC005264.1 0.014622459 0.237162394 4.019617426 1.96E-35 5.25E-34

ARHGAP9 0.499732457 3.005668544 2.588458095 1.97E-35 5.29E-34

TGM7 0.05575785 0.0196858 -1.502019581 1.98E-35 5.31E-34

C10orf71-AS1 0.099054167 0.028312031 -1.806802437 2.02E-35 5.39E-34

AC019197.1 2.144548209 0.331330829 -2.694329396 2.06E-35 5.51E-34

ESRP2 6.509789903 2.227626022 -1.547103932 2.08E-35 5.54E-34

PRELID2 0.421487728 1.795662 2.090953284 2.08E-35 5.54E-34

CDC45 0.204142215 0.98382515 2.268827387 2.08E-35 5.54E-34

ACADSB 31.33014843 8.133489794 -1.945605205 2.12E-35 5.64E-34

LINC00472 4.818639603 1.329253511 -1.858009626 2.14E-35 5.68E-34

IGFBP3 50.72550458 562.2928074 3.470538377 2.16E-35 5.73E-34

PPM1H 9.595256125 2.545904381 -1.914143081 2.21E-35 5.88E-34

DACH1 6.507152597 1.307835817 -2.314844947 2.23E-35 5.93E-34

LNCTAM34A 0.366132159 1.55309642 2.084710995 2.27E-35 6.02E-34

MICE 0.150387182 0.87214647 2.535888843 2.27E-35 6.02E-34

P2RX2 0.158531583 0.013358513 -3.568938958 2.29E-35 6.05E-34

GLDCP1 0.22131459 0.039047373 -2.502801178 2.29E-35 6.05E-34

GPR182 0.806947868 0.156122974 -2.369792624 2.29E-35 6.05E-34

TRIM2 26.69296478 7.629951613 -1.806713741 2.29E-35 6.05E-34

PLAT 26.08385588 7.754012856 -1.750142121 2.32E-35 6.09E-34

GJC1 0.734173093 5.01368415 2.771678966 2.36E-35 6.19E-34

PLK2 5.418387389 25.39042822 2.228349279 2.44E-35 6.40E-34

THSD7A 10.1749424 3.184596181 -1.675838278 2.49E-35 6.51E-34

GPSM3 4.883909375 18.68207295 1.935546209 2.53E-35 6.61E-34

C1QB 23.21477589 179.5414103 2.951201374 2.69E-35 7.02E-34

APELA 0.754653473 0.080067494 -3.236525779 2.70E-35 7.04E-34

BX842568.2 0.59745245 0.110042019 -2.440769377 2.71E-35 7.05E-34

BTN3A2 4.651531708 18.77493027 2.013029783 2.74E-35 7.12E-34

POU5F1 1.138772681 12.5255037 3.459316929 2.74E-35 7.12E-34

BIN2 1.10576349 4.505740075 2.026721247 2.81E-35 7.30E-34

SLC12A5 0.02869143 0.151968432 2.405079885 2.81E-35 7.30E-34

PAPPA2 7.125206577 0.665734173 -3.419913712 2.84E-35 7.36E-34

LAT2 0.871756264 3.916974376 2.16774296 2.92E-35 7.54E-34

GMFG 6.790638792 24.41895476 1.846382248 2.94E-35 7.60E-34

AC016876.3 0.147796569 0.590297167 1.997828644 2.94E-35 7.60E-34

LILRB2 0.712980169 3.528278768 2.307030696 3.02E-35 7.78E-34

SCD 11.16170549 90.01779866 3.0116528 3.02E-35 7.78E-34

AC105020.1 0.103422968 1.258347583 3.604901961 3.08E-35 7.91E-34

AL121987.1 0.20628272 0.021319123 -3.274402952 3.13E-35 8.01E-34

CAB39L 9.514609514 3.04389965 -1.644223651 3.13E-35 8.01E-34

PRKAR1B-AS1 0.555321331 2.88380415 2.376578471 3.13E-35 8.01E-34

TRPV5 1.168906821 0.034900013 -5.065788551 3.13E-35 8.02E-34

LILRB3 0.191575925 1.056059909 2.462703506 3.19E-35 8.14E-34

MARCHF10 0.544944616 0.127717622 -2.093152013 3.22E-35 8.21E-34

SAP30-DT 0.493840268 3.709262762 2.909016086 3.21E-35 8.21E-34

GPAT3 31.91128811 5.607160768 -2.508724505 3.30E-35 8.42E-34

COL4A3 9.259925542 2.441788936 -1.923062091 3.33E-35 8.48E-34

FMNL1 1.03265516 4.96354021 2.265010912 3.33E-35 8.48E-34

GMPR 18.9570153 3.60522437 -2.394570883 3.39E-35 8.61E-34

AC079848.1 1.393802986 0.390118514 -1.83704228 3.51E-35 8.91E-34

OR2A4 0.011538265 1.428506527 6.951937514 3.52E-35 8.93E-34

SHROOM3 7.386548546 1.997940346 -1.886386897 3.57E-35 9.05E-34

AC091390.1 0.169001031 1.517106222 3.166218146 3.60E-35 9.10E-34

FASLG 0.139840728 1.632608074 3.545321997 3.64E-35 9.19E-34

AC079385.3 0.297191032 0.049740003 -2.578912091 3.66E-35 9.24E-34

GCOM1 0.184032681 0.061978029 -1.570133198 3.66E-35 9.24E-34

FOXJ1 2.582773893 0.28889677 -3.160295369 3.67E-35 9.24E-34

PRTG 0.597444692 0.151856139 -1.976099934 3.70E-35 9.32E-34

PATE1 0.023827809 0.003629037 -2.714987456 3.72E-35 9.34E-34

AL592546.2 0.840954661 0.119799743 -2.811403206 3.73E-35 9.37E-34

AC008760.2 0.19677516 6.251811183 4.989654192 3.73E-35 9.37E-34

AC022893.2 0.031464223 0.006220315 -2.338652851 3.80E-35 9.51E-34

ZNF728 0.516048889 0.097710968 -2.400915329 3.91E-35 9.78E-34

AL157373.2 2.742082392 0.618913586 -2.147462024 3.94E-35 9.83E-34

CCNA2 0.615271843 2.401520603 1.964652309 3.98E-35 9.91E-34

LSAMP 1.256063177 0.22611327 -2.473791466 4.16E-35 1.04E-33

AL499605.1 0.107381578 0.005933525 -4.177713317 4.26E-35 1.06E-33

ACKR2 0.926836596 0.190462646 -2.282806932 4.50E-35 1.12E-33

ENPP6 6.668230133 0.221967216 -4.908885381 4.54E-35 1.13E-33

SKA3 0.189742325 0.803104476 2.08154615 4.54E-35 1.13E-33

LINC00887 0.253411989 10.45881041 5.367090177 4.62E-35 1.14E-33

PHKA2-AS1 0.171816153 1.216439595 2.823727101 4.87E-35 1.20E-33

AC123788.1 0.044006095 0.00504621 -3.124431178 4.88E-35 1.21E-33

DEFB131E 0.009131719 0.276969319 4.922695937 4.91E-35 1.21E-33

ADCY6-DT 1.43587889 0.329297942 -2.12446867 4.95E-35 1.22E-33

RTCA-AS1 5.171181863 1.699710851 -1.605204702 4.96E-35 1.22E-33

AL645939.2 0.024869746 0.543143741 4.448870369 4.96E-35 1.22E-33

AC079760.2 0.11451777 4.718793375 5.364774613 4.96E-35 1.22E-33

AL023806.1 0.679632073 0.128522978 -2.402727619 5.00E-35 1.23E-33

NPM1P25 5.999087301 1.742020769 -1.783981202 5.05E-35 1.24E-33

CCL5 5.452020633 51.86301717 3.249843211 5.05E-35 1.24E-33

SLC7A14 0.074620497 0.010435995 -2.838003813 5.09E-35 1.25E-33

DNASE1L3 10.32917564 2.346363889 -2.138226441 5.14E-35 1.26E-33

CEL 3.982085128 0.537627863 -2.888844249 5.23E-35 1.28E-33

AL109946.1 0.010136973 0.898284903 6.469474247 5.25E-35 1.28E-33

S100A14 12.84185961 2.839144884 -2.177325754 5.27E-35 1.29E-33

CAV1 14.6949154 67.82712973 2.206545389 5.37E-35 1.31E-33

ANKRD18DP 0.00226623 0.045559803 4.329395268 5.46E-35 1.33E-33

AC108463.1 0.698628984 3.719427627 2.412482224 5.56E-35 1.35E-33

GZMH 0.977967646 7.902790277 3.01450348 5.56E-35 1.35E-33

VWA2 1.127260921 0.148274917 -2.926475015 5.71E-35 1.38E-33

ARHGDIB 40.47512806 127.8264013 1.659078291 5.71E-35 1.38E-33

E2F1 0.613877008 2.711799959 2.143229217 5.71E-35 1.38E-33

AP003469.2 0.071256052 0.730920074 3.358629201 5.73E-35 1.39E-33

ABCB1 22.41781958 6.986940544 -1.681913196 5.76E-35 1.39E-33

SYNE4 2.431315445 0.48135982 -2.336549458 5.86E-35 1.41E-33

SASH3 1.770754143 10.05557653 2.505559977 5.97E-35 1.44E-33

LINC00652 0.228496738 0.055164696 -2.050356389 6.18E-35 1.49E-33

HSPA7 0.288952251 4.601849173 3.993310684 6.18E-35 1.49E-33

AC093802.2 0.039491471 0.002213053 -4.15743135 6.28E-35 1.51E-33

APOBEC3D 0.729074669 2.993356202 2.037625483 6.35E-35 1.52E-33

JPH4 1.132185136 0.375809416 -1.591036768 6.41E-35 1.54E-33

AC144831.1 3.273022317 0.553122434 -2.564952677 6.58E-35 1.57E-33

PRF1 1.677410115 10.782119 2.684333371 6.58E-35 1.57E-33

SLC25A25 12.28048744 3.37535946 -1.863254771 6.69E-35 1.60E-33

TMC8 0.651497248 3.826700659 2.554270062 6.87E-35 1.64E-33

NEIL3 0.06360382 0.418741059 2.718873069 6.93E-35 1.65E-33

PALM 15.80622343 5.191854036 -1.606170978 7.06E-35 1.67E-33

RUNX3 0.905378113 5.939608304 2.713775459 7.06E-35 1.67E-33

SDHAP3 2.647056157 15.78444264 2.576042612 7.12E-35 1.68E-33

PATL2 0.094733664 0.707778679 2.901349218 7.12E-35 1.68E-33

AGR2 4.524751441 0.944386749 -2.260388838 7.35E-35 1.74E-33

LINC00461 0.211789435 0.021861231 -3.27618408 7.38E-35 1.74E-33

PDK1 1.321960119 5.058250405 1.935959804 7.51E-35 1.77E-33

DGCR10 0.155184998 1.988954659 3.679949391 7.53E-35 1.77E-33

DLG1-AS1 0.251982738 0.056230329 -2.163904526 7.54E-35 1.77E-33

AC090197.1 0.111106422 0.503296887 2.179467472 7.57E-35 1.78E-33

CNKSR1 2.830565743 0.468329354 -2.59549506 7.64E-35 1.79E-33

TP73 0.029289927 0.245782348 3.06890478 7.64E-35 1.79E-33

LDHA 90.01795639 283.8685387 1.656938245 7.71E-35 1.80E-33

RGS1 3.688435003 32.59565004 3.143598729 7.71E-35 1.80E-33

UBE2C 0.822440413 6.01320007 2.870149899 7.78E-35 1.82E-33

ACBD7 0.359549795 0.114927291 -1.645470167 7.84E-35 1.83E-33

AL139039.3 0.135433438 0.014614102 -3.212150849 7.85E-35 1.83E-33

AC005281.1 1.762453478 0.262359388 -2.747968855 8.05E-35 1.88E-33

PPP1R3G 0.450707474 2.008353376 2.15574986 8.05E-35 1.88E-33

KIF18B 0.106537277 0.703703389 2.723609142 8.05E-35 1.88E-33

LINC00462 0.339391906 15.43996781 5.507573775 8.14E-35 1.89E-33

ZNF503-AS2 2.694723569 0.838852464 -1.683648287 8.20E-35 1.91E-33

MYO1G 0.345630548 2.198040497 2.668915327 8.42E-35 1.95E-33

AP003555.1 0.027965087 0.437592764 3.967890182 8.79E-35 2.04E-33

AICDA 0.012507122 0.376703428 4.912607391 8.82E-35 2.04E-33

RUFY4 0.008875577 0.282116978 4.990308735 8.92E-35 2.06E-33

AL024508.1 0.603267806 0.176453586 -1.77350984 9.03E-35 2.09E-33

PFKFB2 8.036807147 2.444758198 -1.716930683 9.19E-35 2.12E-33

EPHA1 2.791812264 0.945351179 -1.562279665 9.19E-35 2.12E-33

KCNJ12 1.966168072 0.39195177 -2.326638607 9.35E-35 2.16E-33

AC127455.1 0.362458687 0.043461645 -3.060001868 9.45E-35 2.18E-33

ARHGAP25 1.824978474 6.30563266 1.78876168 9.52E-35 2.19E-33

GBP2 6.270769403 24.73639289 1.979920766 9.52E-35 2.19E-33

PCLAF 0.335500439 1.545367802 2.203563686 9.52E-35 2.19E-33

AQP7P2 0.103786206 0.012433941 -3.06125921 9.85E-35 2.26E-33

LST1 1.79740901 8.571814986 2.253681972 9.86E-35 2.26E-33

AC015911.7 0.162196999 1.552927104 3.259171074 9.95E-35 2.28E-33

AC005082.1 6.499592926 0.791732799 -3.037263841 1.00E-34 2.30E-33

LY86 1.905317428 10.66029259 2.484143758 1.00E-34 2.30E-33

LGI2 6.551814993 0.661253716 -3.308618789 1.01E-34 2.31E-33

NME8 0.041484689 0.295523026 2.832619658 1.01E-34 2.31E-33

COL23A1 2.064496081 61.90506148 4.906195787 1.04E-34 2.37E-33

AC091965.4 0.81290199 0.180198654 -2.173493186 1.07E-34 2.44E-33

AQP3 181.3012126 38.36542064 -2.240510097 1.11E-34 2.51E-33

AL133415.1 0.133377109 0.84456853 2.662703405 1.12E-34 2.53E-33

LINC00528 0.078201521 0.397998823 2.347495584 1.12E-34 2.54E-33

IRF6 16.37202504 5.311937999 -1.623922566 1.14E-34 2.57E-33

DEFB1 841.20163 207.3787319 -2.020183702 1.15E-34 2.59E-33

KCNH3 0.711739521 0.181763144 -1.969289648 1.17E-34 2.64E-33

GPM6B 3.772448464 0.875595194 -2.107165251 1.18E-34 2.66E-33

AURKB 0.239296783 1.492426949 2.64078741 1.21E-34 2.72E-33

BBC3 1.081136615 3.449776983 1.673954262 1.24E-34 2.79E-33

FSTL4 1.11280488 0.194508112 -2.516298421 1.27E-34 2.86E-33

GZMA 2.447645085 21.84271956 3.157686208 1.27E-34 2.86E-33

FKBP11 1.354539188 5.232706036 1.949755081 1.28E-34 2.88E-33

NEK6 7.426096165 30.81438683 2.052928181 1.28E-34 2.88E-33

SLC17A9 0.202426168 2.049075086 3.339505141 1.32E-34 2.95E-33

GOLGA8VP 0.077264523 0.680351597 3.138402456 1.32E-34 2.96E-33

NUSAP1 1.626359465 7.233556383 2.15305896 1.33E-34 2.97E-33

SH3BP2 4.034804486 13.43978336 1.735939211 1.37E-34 3.05E-33

DIRAS1 2.707172581 0.517510289 -2.387127409 1.41E-34 3.15E-33

FAM215A 0.541830385 0.052768803 -3.360084142 1.44E-34 3.22E-33

P2RY1 0.436379634 2.44693325 2.48731907 1.46E-34 3.25E-33

SLA2 0.277966546 2.135034307 2.941276083 1.46E-34 3.25E-33

TMCC1-AS1 0.216668486 0.745242156 1.782219952 1.48E-34 3.28E-33

FERMT3 2.426102363 10.57932314 2.124535 1.48E-34 3.28E-33

FUT11 4.624923431 13.74948659 1.571876363 1.52E-34 3.35E-33

CD247 0.498860564 3.068987214 2.621054103 1.52E-34 3.35E-33

AC108463.3 0.034094906 0.455254239 3.739044354 1.54E-34 3.40E-33

MIAT 0.033592374 0.734769957 4.451086975 1.54E-34 3.41E-33

HMGN1P17 1.090400898 0.139149509 -2.970150928 1.57E-34 3.47E-33

CSRNP3 1.294016257 0.431942902 -1.582943219 1.60E-34 3.52E-33

C1QC 25.38847476 173.9455879 2.776390454 1.61E-34 3.55E-33

AC000068.1 0.584852053 0.184171528 -1.667021678 1.63E-34 3.58E-33

ARRDC5 0.025069434 0.36263952 3.854534831 1.69E-34 3.71E-33

UMODL1 0.126600651 0.025685706 -2.301247193 1.69E-34 3.72E-33

MCM10 0.085175469 0.404863489 2.248925649 1.70E-34 3.73E-33

SLC9A3 57.94358244 5.313743508 -3.446848388 1.73E-34 3.78E-33

TREML1 0.059384478 0.469524638 2.983043061 1.73E-34 3.78E-33

KIF5A 0.445066445 0.103096773 -2.110021565 1.74E-34 3.81E-33

MELK 0.259265315 1.328792969 2.357615229 1.74E-34 3.81E-33

CES4A 0.604520013 7.942975039 3.715817464 1.76E-34 3.84E-33

FGD2 0.269128156 1.603339915 2.57471508 1.81E-34 3.93E-33

ANXA4 21.65618764 78.2592556 1.853482095 1.87E-34 4.07E-33

ARRDC2 4.970225778 20.02854001 2.010673965 1.89E-34 4.10E-33

HOXB6 16.81898631 3.883185867 -2.114778088 1.94E-34 4.21E-33

NCAPG 0.215689766 0.934882554 2.115827412 1.97E-34 4.28E-33

TGFBR3 15.92464507 5.356685893 -1.571848612 1.99E-34 4.31E-33

SNHG26 0.169398213 0.703647143 2.054433488 1.99E-34 4.31E-33

FCGR2C 0.161353472 1.876844382 3.540012511 1.99E-34 4.31E-33

AC005336.3 0.029164053 0.002907112 -3.326532862 2.00E-34 4.32E-33

EME1 0.118293792 0.502080546 2.085544463 2.01E-34 4.33E-33

CARD16 1.644555668 6.222953559 1.919901636 2.03E-34 4.37E-33

TBX21 0.179919735 1.026242273 2.511946014 2.06E-34 4.44E-33

CDC25C 0.070735089 0.448310259 2.663999554 2.16E-34 4.65E-33

FOXA3 1.737423934 0.228240284 -2.928324466 2.19E-34 4.71E-33

PITRM1-AS1 0.087117755 0.360992507 2.050930216 2.19E-34 4.71E-33

AC009779.4 0.272422906 1.129101781 2.051255621 2.19E-34 4.71E-33

TOX3 6.512128418 1.443172972 -2.173884925 2.21E-34 4.74E-33

TNFAIP8L2 1.337980286 6.368755729 2.25095468 2.21E-34 4.74E-33

PTTG1 0.751440964 3.737554144 2.314362809 2.23E-34 4.77E-33

AC023421.1 10.3351166 0.109043989 -6.566500613 2.25E-34 4.80E-33

AC007996.1 2.845346489 0.878342458 -1.695748897 2.25E-34 4.80E-33

MATK 0.298408072 1.447925052 2.278628459 2.25E-34 4.80E-33

AC011899.2 0.091457122 0.624373049 2.771240845 2.25E-34 4.80E-33

KIF4A 0.26490767 1.223594342 2.207563821 2.27E-34 4.84E-33

LINC02675 2.65848761 0.332068375 -3.001053505 2.29E-34 4.87E-33

UHRF1 0.175342857 0.999957169 2.511687642 2.35E-34 5.00E-33

DUSP5P1 0.033008682 0.561697978 4.088877161 2.35E-34 5.00E-33

AC148477.4 5.974009204 0.803677613 -2.89401066 2.37E-34 5.04E-33

CENPU 0.648318814 2.289110771 1.820011932 2.39E-34 5.08E-33

TNS4 1.063752366 0.205528647 -2.371750942 2.48E-34 5.25E-33

ALOX15B 0.078100228 1.511289479 4.274309451 2.52E-34 5.34E-33

AC108704.2 0.107932879 1.003006723 3.21612496 2.54E-34 5.38E-33

SHROOM2P1 0.019067493 0.002194718 -3.119007769 2.55E-34 5.38E-33

DCXR-DT 0.659760822 0.078579336 -3.069721221 2.57E-34 5.43E-33

RASSF2 2.203617176 10.21528677 2.212784186 2.59E-34 5.46E-33

PFKFB4 0.961428575 5.080213919 2.40163766 2.61E-34 5.51E-33

PRAM1 0.231288849 1.287315347 2.476597886 2.63E-34 5.55E-33

AC004921.1 0.137649601 0.70423988 2.355066504 2.66E-34 5.59E-33

CST7 2.115860032 18.83098504 3.15379237 2.68E-34 5.64E-33

C1orf162 2.351763361 11.95347687 2.345615505 2.70E-34 5.68E-33

AC092106.1 0.028573368 0.003442662 -3.053074596 2.74E-34 5.77E-33

P4HA1 16.37654519 53.63917231 1.711655938 2.77E-34 5.82E-33

PTP4A3 5.701081667 20.8782656 1.872694296 2.77E-34 5.82E-33

GINS2 0.527853749 1.929898306 1.870314663 2.85E-34 5.96E-33

NPSR1-AS1 0.221397411 0.024755133 -3.160838719 2.86E-34 5.98E-33

ADGRA1 0.114009011 0.019623113 -2.538522054 2.90E-34 6.06E-33

AL109615.3 0.62872963 4.433096658 2.81780316 2.98E-34 6.22E-33

NLRP3P1 0.004716156 0.603159632 6.998784646 3.07E-34 6.42E-33

B4GALNT2 6.037468302 0.450305995 -3.74496612 3.08E-34 6.42E-33

CBLC 4.100831594 0.988518325 -2.052576883 3.19E-34 6.64E-33

ATP2B3 0.183795848 0.05176835 -1.827961941 3.19E-34 6.64E-33

ITGB2 4.560106314 24.18347272 2.406882064 3.25E-34 6.75E-33

DNAJC5B 0.038939513 0.519577129 3.738031172 3.25E-34 6.75E-33

SNHG15 1.780567522 5.903480523 1.729228631 3.30E-34 6.86E-33

AMELY 0.083088657 0.005391562 -3.945876374 3.38E-34 7.01E-33

IGSF5 0.280319812 0.059824916 -2.228255347 3.40E-34 7.04E-33

AC005606.2 0.024246603 0.004083831 -2.569787529 3.45E-34 7.15E-33

AC113145.1 0.048555908 0.011348572 -2.097136041 3.50E-34 7.25E-33

PHF21B 0.236903305 0.051896333 -2.190593828 3.52E-34 7.27E-33

DCN 37.8382284 6.456298469 -2.551065363 3.60E-34 7.44E-33

AC087762.1 1.789442771 0.256268173 -2.803784182 3.66E-34 7.55E-33

E2F2 0.079670582 0.409862349 2.363020441 3.67E-34 7.56E-33

EFNA3 0.495254386 2.558876447 2.369268832 3.67E-34 7.56E-33

ZNF98 0.462720721 0.104169643 -2.151206792 3.69E-34 7.59E-33

LGALS1 59.06596569 252.1866685 2.09409303 3.70E-34 7.61E-33

AC025265.1 0.078608932 1.250925062 3.992158307 3.70E-34 7.61E-33

LINC02012 0.415988932 0.089509801 -2.216427584 3.75E-34 7.69E-33

LURAP1 3.753048014 1.096671007 -1.774931954 3.87E-34 7.92E-33

MS4A7 2.808058072 15.56794507 2.470933848 3.87E-34 7.92E-33

NCAPH 0.261502444 0.994792243 1.927570827 3.90E-34 7.99E-33

MYBL2 0.405437421 2.607001958 2.68484051 3.97E-34 8.12E-33

SMIM10L2B 3.039244736 1.042249146 -1.544012663 4.00E-34 8.19E-33

CAPN11 0.14195949 0.646903657 2.188071576 4.06E-34 8.29E-33

AC025431.1 0.206175978 0.052062252 -1.985566626 4.09E-34 8.35E-33

ZAP70 0.24077723 1.853038361 2.944121876 4.11E-34 8.38E-33

HSPB8 19.22729801 86.76589821 2.173972091 4.14E-34 8.44E-33

HJURP 0.175017236 1.042089992 2.573910959 4.14E-34 8.44E-33

AC025811.1 0.417126281 0.087348822 -2.25562406 4.16E-34 8.48E-33

VSIG1 0.073522565 1.1310414 3.943320824 4.20E-34 8.54E-33

HSPB7 14.64025027 1.421711648 -3.364239424 4.29E-34 8.71E-33

AL683807.1 0.02567042 0.591022164 4.52503342 4.31E-34 8.73E-33

ARNT2 16.22907947 4.725729053 -1.779972351 4.33E-34 8.77E-33

MTFP1 2.706267047 8.233979576 1.605285663 4.44E-34 8.99E-33

JAKMIP1 0.046943229 0.577018503 3.619628589 4.44E-34 8.99E-33

ATP2C2 0.589952918 0.152278652 -1.953886121 4.48E-34 9.07E-33

HOXB8 17.58115351 3.680588199 -2.256021482 4.52E-34 9.13E-33

SUCLG2-AS1 1.044462781 0.356145849 -1.552221002 4.52E-34 9.13E-33

FOXM1 0.435162837 2.359437653 2.438815788 4.56E-34 9.20E-33

ZNF488 0.819365013 0.075804794 -3.434145305 4.58E-34 9.23E-33

LINC02432 1.300241627 0.18074666 -2.846738854 4.60E-34 9.26E-33

PPARGC1A 14.43103276 4.334165749 -1.735348319 4.60E-34 9.26E-33

FREM2 5.036462653 1.644065175 -1.615143323 4.60E-34 9.26E-33

AC004816.1 1.608546747 0.515132742 -1.642741719 4.64E-34 9.33E-33

GTSE1 0.177278698 0.887581877 2.323861021 4.68E-34 9.40E-33

FAM111B 0.264237417 1.205421254 2.189630731 4.76E-34 9.55E-33

TNFSF14 0.114871977 1.55979764 3.763260072 4.76E-34 9.55E-33

FAM107A 17.70996376 6.201400776 -1.513895226 4.85E-34 9.71E-33

SALL3 0.65200688 0.14056505 -2.213649258 4.86E-34 9.73E-33

LCE2D 0.067431802 0.002326203 -4.857380419 5.04E-34 1.01E-32

SYT1 1.069950865 0.224506289 -2.25271678 5.06E-34 1.01E-32

NFAM1 0.668158324 2.910535405 2.123022665 5.15E-34 1.03E-32

ADA 1.034734726 4.739863256 2.195584485 5.15E-34 1.03E-32

EIF4EBP1 9.736554972 41.00997178 2.074491443 5.29E-34 1.05E-32

SIGLEC9 0.429428 2.155460318 2.327507835 5.48E-34 1.09E-32

BDH1 3.506545168 0.941491531 -1.897030289 5.52E-34 1.10E-32

C4orf47 0.914447017 5.236238429 2.517559304 5.52E-34 1.10E-32

CCND1 36.32078097 183.1363816 2.334051294 5.57E-34 1.11E-32

AC145098.1 0.201499502 1.140631816 2.500985002 5.77E-34 1.15E-32

SKA1 0.134348722 0.577037356 2.102682122 5.82E-34 1.15E-32

VAV1 0.70746699 3.586581249 2.341874574 5.87E-34 1.16E-32

RAD54L 0.174777705 0.998978966 2.514933138 5.97E-34 1.18E-32

BATF 0.477197401 3.853560308 3.01353388 6.08E-34 1.20E-32

KLK6 7.000276228 0.167178771 -5.38794829 6.17E-34 1.22E-32

PAG1 1.443285083 4.876323406 1.756437516 6.18E-34 1.22E-32

NDC80 0.315854479 1.294332188 2.034875996 6.24E-34 1.23E-32

HK3 0.300916402 1.835402138 2.608661545 6.24E-34 1.23E-32

FAM181B 0.465148054 0.097230943 -2.258202568 6.29E-34 1.24E-32

COL9A2 6.876993875 1.50120491 -2.195657147 6.29E-34 1.24E-32

CENPK 0.182479053 0.703768503 1.947370079 6.52E-34 1.28E-32

C13orf42 0.182422098 0.004952541 -5.202967692 6.64E-34 1.30E-32

DNMBP-AS1 0.726244099 0.244411314 -1.571143465 6.75E-34 1.32E-32

ANK2 17.19651207 4.063837527 -2.081201347 6.86E-34 1.34E-32

TNFSF9 0.555273832 6.020362831 3.438579124 6.92E-34 1.35E-32

AC026462.3 2.202735956 0.421615756 -2.385295881 6.99E-34 1.37E-32

ADH1B 20.46521623 3.096128932 -2.724636454 7.11E-34 1.39E-32

TFAP2A 2.712707357 0.655883323 -2.048222322 7.17E-34 1.40E-32

HCLS1 4.375098833 16.71255883 1.933545126 7.17E-34 1.40E-32

C5orf38 3.068163514 0.499474362 -2.61889284 7.31E-34 1.42E-32

INPP5D 1.973086658 6.462947246 1.711737895 7.42E-34 1.45E-32

IGSF10 0.34427253 0.05160863 -2.737866836 7.68E-34 1.49E-32

FAM163A 0.060743942 0.571592853 3.234175437 7.82E-34 1.52E-32

GBP5 0.402860415 4.777350086 3.567858643 7.89E-34 1.53E-32

ALDH6A1 74.57451519 9.606474867 -2.956603663 7.95E-34 1.54E-32

GAS5 14.87308425 48.29328388 1.699118716 7.95E-34 1.54E-32

AL031846.1 0.032092918 0.427136159 3.734369168 8.01E-34 1.55E-32

C1QA 30.67384269 188.6313927 2.620488976 8.09E-34 1.57E-32

OLFML2B 3.230107157 20.55591299 2.669899519 8.24E-34 1.59E-32

PSTPIP1 0.267624642 1.873409795 2.807383647 8.31E-34 1.61E-32

DEF6 1.186172283 5.79958666 2.289636516 8.38E-34 1.62E-32

FBXO2 12.82711423 2.0297757 -2.659804421 8.60E-34 1.66E-32

TPX2 0.917095694 4.347690937 2.245105203 8.60E-34 1.66E-32

SEPTIN1 0.907773419 3.300159965 1.862131806 8.90E-34 1.71E-32

MMP25 0.159609838 0.757231273 2.246184413 8.90E-34 1.71E-32

SH3GL2 6.929802973 0.996888801 -2.797309843 8.95E-34 1.72E-32

AL359317.2 0.237511063 0.041679885 -2.510571503 9.19E-34 1.77E-32

AC022733.1 0.297092976 0.021759239 -3.7712145 9.22E-34 1.77E-32

ACSL6 0.78189695 0.181689791 -2.10550112 9.22E-34 1.77E-32

GLOD5 3.269687926 0.62183194 -2.394556319 9.28E-34 1.78E-32

ANO5 2.884860404 0.697612012 -2.048004723 9.30E-34 1.78E-32

CNTN3 1.863694217 0.564183385 -1.723929086 9.38E-34 1.79E-32

CDO1 1.754360218 0.56376283 -1.637784737 9.38E-34 1.79E-32

BIRC5 0.401040877 2.170156096 2.435977619 9.38E-34 1.79E-32

AC092809.5 0.057033867 0.339361894 2.572933821 9.38E-34 1.79E-32

GDNF 0.462576742 0.132791763 -1.800527069 9.46E-34 1.81E-32

LTB4R2 0.168818613 0.802658086 2.249311586 9.46E-34 1.81E-32

CEP55 0.350289878 1.757562239 2.326954574 9.63E-34 1.84E-32

NBPF8 0.260077852 1.284331605 2.304002294 9.71E-34 1.85E-32

AL162724.2 0.207476886 1.892004637 3.188893097 9.77E-34 1.86E-32

KIAA1549L 0.611382906 0.144767673 -2.078336735 9.88E-34 1.88E-32

C16orf89 16.52573969 2.027019195 -3.027283193 1.01E-33 1.91E-32

ELMO1 1.445801311 5.114068108 1.82260207 1.01E-33 1.91E-32

TRIM9 0.273619809 2.002291953 2.871407761 1.05E-33 1.99E-32

AC122134.1 0.023159963 0.57588524 4.636076514 1.05E-33 1.99E-32

PTGER3 36.22309219 8.233594074 -2.137315479 1.06E-33 2.00E-32

AC243829.4 0.009532238 0.255826956 4.746209536 1.09E-33 2.07E-32

CDC6 0.273359045 1.075979973 1.976782209 1.10E-33 2.07E-32

DTL 0.312437717 1.368807148 2.131278668 1.10E-33 2.07E-32

CALB1 53.47279118 0.31468416 -7.408756613 1.11E-33 2.08E-32

CRACR2A 0.08711407 0.402706571 2.208751361 1.11E-33 2.08E-32

AC073115.2 0.025104265 2.518519716 6.648499744 1.11E-33 2.09E-32

SPC24 0.29440191 1.416040924 2.266004024 1.13E-33 2.11E-32

CCNE2 0.147261585 0.442864299 1.588483561 1.13E-33 2.13E-32

AC079015.1 0.069813338 0.732073011 3.390412942 1.14E-33 2.14E-32

ADAM18 0.032297591 0.55225607 4.095838916 1.14E-33 2.15E-32

LRRC37A6P 1.007007514 0.234723356 -2.101041138 1.16E-33 2.18E-32

PROX1-AS1 0.190016106 0.013245573 -3.842539534 1.17E-33 2.19E-32

PRSS53 0.054941561 0.64802855 3.560087575 1.19E-33 2.22E-32

AC135048.4 0.154308769 1.205756734 2.966046909 1.22E-33 2.27E-32

AC008415.1 0.03657233 0.006533528 -2.48481843 1.23E-33 2.28E-32

PDLIM1 27.92281167 87.90733683 1.654539359 1.24E-33 2.30E-32

NUF2 0.191138972 0.74719425 1.96686139 1.24E-33 2.30E-32

PARP4P3 0.184884605 0.029866906 -2.630005403 1.24E-33 2.31E-32

EOMES 0.180121103 2.13525873 3.567371772 1.25E-33 2.32E-32

VSNL1 0.529475108 0.143777375 -1.880726189 1.26E-33 2.34E-32

MYZAP 2.186091749 0.767622731 -1.509884612 1.30E-33 2.41E-32

APOC1P1 0.018793974 0.307021313 4.029996764 1.33E-33 2.46E-32

DLX1 0.029448873 0.269472099 3.19385156 1.34E-33 2.47E-32

ITGAL 1.221017113 7.198869878 2.559687022 1.35E-33 2.49E-32

PLAG1 0.776258156 0.245020357 -1.663634903 1.43E-33 2.64E-32

SFTA2 2.895263353 0.515823432 -2.488745366 1.45E-33 2.67E-32

DCLK3 0.020124175 0.199523588 3.309557753 1.47E-33 2.71E-32

MTNR1A 0.669944477 0.089751127 -2.900039573 1.47E-33 2.71E-32

AL109809.1 0.026992176 0.261042729 3.273672788 1.49E-33 2.74E-32

CCNB2 0.573388583 1.901121406 1.729265581 1.54E-33 2.82E-32

AMZ1 0.02425658 0.369250299 3.928151014 1.54E-33 2.82E-32

IGFBP2 25.08906847 7.10265177 -1.820629247 1.56E-33 2.86E-32

KISS1R 0.164233087 6.056842695 5.204749229 1.57E-33 2.88E-32

TCAF2 0.625867418 1.991993867 1.670284227 1.58E-33 2.88E-32

EBI3 0.982246128 4.88358894 2.31378529 1.62E-33 2.95E-32

APOL5 0.010578173 0.172650952 4.028695968 1.63E-33 2.98E-32

UNC13D 0.448942081 2.060304782 2.198256536 1.66E-33 3.03E-32

AP003071.4 1.116453966 0.169823319 -2.716817289 1.67E-33 3.05E-32

KCNA4 0.103192026 0.021309489 -2.275763578 1.70E-33 3.10E-32

DPP10 0.138597929 0.04124239 -1.748705858 1.72E-33 3.13E-32

PPP1R18 6.110836583 19.19803279 1.651516681 1.73E-33 3.15E-32

SLC11A1 0.304216049 1.604221745 2.398705405 1.83E-33 3.30E-32

PTP4A2P2 0.265044656 2.190796107 3.047147867 1.83E-33 3.30E-32

AC073257.2 0.125382719 1.172503457 3.225181755 1.84E-33 3.33E-32

CSPG4 2.881595844 19.60701756 2.766430192 1.87E-33 3.39E-32

AC105384.1 1.693074351 0.060408466 -4.808750773 1.89E-33 3.41E-32

RNF223 0.379922967 0.051825229 -2.873980432 1.89E-33 3.42E-32

GSTO2 2.900973017 0.828437673 -1.80807181 1.97E-33 3.55E-32

HS3ST6 0.425850615 0.035730731 -3.575110095 1.99E-33 3.57E-32

AC040160.1 0.11782695 0.513147147 2.122703027 1.99E-33 3.57E-32

PRR7-AS1 0.03829638 0.382321865 3.319507784 1.99E-33 3.57E-32

BTBD16 0.132070097 1.538103079 3.541776433 1.99E-33 3.57E-32

AP000781.2 0.016316239 0.142650603 3.128105428 2.01E-33 3.61E-32

AL049629.1 2.086014158 0.406784251 -2.358413219 2.06E-33 3.69E-32

CASP1 2.518213747 8.464012536 1.748941019 2.10E-33 3.74E-32

PTPRE 1.181018826 3.802451735 1.686897973 2.11E-33 3.77E-32

TPTEP1 1.90681709 0.39160884 -2.283681222 2.13E-33 3.80E-32

LINC02762 0.432109079 2.446632403 2.501329909 2.13E-33 3.80E-32

VWA8-AS1 0.131028592 0.026793018 -2.289952675 2.16E-33 3.85E-32

ASF1B 0.718953047 3.162062183 2.136896278 2.21E-33 3.93E-32

TTC36 3.104165213 0.427300864 -2.860881203 2.24E-33 3.99E-32

BMP1 2.151834175 7.837272629 1.864784779 2.28E-33 4.06E-32

E2F8 0.086575633 0.531694488 2.618564581 2.29E-33 4.07E-32

C3 22.99807194 235.7467393 3.357652995 2.34E-33 4.16E-32

AL031123.3 0.686247687 0.160048114 -2.100223702 2.40E-33 4.26E-32

CEP250-AS1 0.074205779 0.410289448 2.467038598 2.40E-33 4.26E-32

ZMYND15 0.558497018 1.872433833 1.745293256 2.43E-33 4.29E-32

THEMIS2 2.093788319 8.922891776 2.091395748 2.43E-33 4.29E-32

CORO1A 2.703767621 14.76266884 2.448910492 2.51E-33 4.43E-32

MPC1 84.37310833 27.62483053 -1.610817638 2.53E-33 4.46E-32

AC004847.1 0.11487841 0.917831515 2.998121655 2.53E-33 4.46E-32

RAB7B 0.426023451 1.749203007 2.037692982 2.58E-33 4.54E-32

AL109615.2 0.013303012 0.393285501 4.885752198 2.58E-33 4.54E-32

NECTIN4 2.119452682 0.550493021 -1.944895577 2.69E-33 4.73E-32

AC135977.1 0.514964235 0.156835511 -1.715219981 2.71E-33 4.75E-32

CALHM6 1.536161393 12.14379716 2.982817896 2.78E-33 4.88E-32

PFKP 28.33611743 110.7615584 1.966743257 2.81E-33 4.91E-32

TBC1D10C 0.398918212 2.37154956 2.571665124 2.81E-33 4.91E-32

GPC5-IT1 0.054111076 0.010658862 -2.343870482 2.84E-33 4.96E-32

PTPRN 0.164265237 1.186600894 2.852735669 2.86E-33 4.98E-32

RBFOX1 0.09872742 0.026733487 -1.884802803 2.92E-33 5.09E-32

CTSW 1.093540138 9.081308296 3.05389398 2.93E-33 5.11E-32

MST1L 5.617494215 0.833366217 -2.752904212 2.98E-33 5.18E-32

GAB3 0.56600116 1.789048151 1.660315303 3.06E-33 5.32E-32

EHD2 19.15319929 90.67564636 2.243129729 3.22E-33 5.58E-32

MUC12 0.008461517 0.1613938 4.253525069 3.22E-33 5.58E-32

MAB21L4 5.350334356 0.388980104 -3.781860784 3.23E-33 5.60E-32

LINC00475 0.058809384 0.796684891 3.759890921 3.25E-33 5.62E-32

ZNF683 0.115487873 1.209809755 3.388966927 3.29E-33 5.69E-32

CPHL1P 0.007774819 0.171896525 4.46658746 3.34E-33 5.77E-32

TBX3 6.350419642 1.790014118 -1.826880964 3.36E-33 5.80E-32

KIF6 0.310467042 0.108117119 -1.521845152 3.36E-33 5.80E-32

AL445985.1 0.348011542 0.071472845 -2.283668038 3.45E-33 5.94E-32

DIRAS3 4.383876575 1.240958533 -1.820752273 3.48E-33 5.99E-32

HCK 2.050127786 8.59168049 2.067226506 3.51E-33 6.04E-32

BTK 0.541992096 2.527741105 2.221504991 3.60E-33 6.20E-32

KIFC1 0.467072225 1.813281674 1.956885488 3.63E-33 6.25E-32

SMIM22 3.495290079 0.980954555 -1.833153985 3.69E-33 6.34E-32

LINC00343 0.023717629 0.004420815 -2.423575414 3.72E-33 6.39E-32

GPR19 0.049171793 0.227858184 2.212233335 3.74E-33 6.43E-32

AC006033.2 0.1086273 0.542931742 2.321384106 3.91E-33 6.70E-32

AC104590.1 0.232575246 0.052731548 -2.140959312 3.92E-33 6.72E-32

LINC02188 0.288385679 4.111824487 3.833707255 3.96E-33 6.78E-32

SERPINH1 17.48710889 50.73820463 1.53678068 3.99E-33 6.83E-32

BUB1 0.248782564 1.069230272 2.103615307 3.99E-33 6.83E-32

PLXNA4 1.030777932 0.183567644 -2.48934986 4.03E-33 6.88E-32

AC006449.5 0.54046313 0.182564402 -1.565790715 4.03E-33 6.88E-32

CENPA 0.131554968 0.704125775 2.420167428 4.13E-33 7.05E-32

JAK3 0.925142496 4.953011168 2.420558374 4.17E-33 7.11E-32

WAS 1.768378444 8.080171018 2.191958774 4.20E-33 7.16E-32

AC008105.3 0.051765831 0.825717928 3.995576987 4.20E-33 7.16E-32

PTCRA 0.030712418 0.231175395 2.912093842 4.22E-33 7.19E-32

CAVIN3 6.530876361 31.40651909 2.265715551 4.31E-33 7.34E-32

AC091057.1 0.079306576 0.297423205 1.907004817 4.39E-33 7.46E-32

LILRA6 0.102931937 0.594055662 2.528907434 4.39E-33 7.46E-32

SLC7A4 0.323193877 0.095937491 -1.75223325 4.67E-33 7.90E-32

CLDN10-AS1 1.514802259 0.280193245 -2.434635396 4.78E-33 8.08E-32

NRAD1 2.061569184 0.335522709 -2.619260557 4.86E-33 8.20E-32

AP005229.2 0.118296607 0.009870693 -3.58311346 4.93E-33 8.30E-32

IQGAP3 0.260562429 1.461545535 2.487793796 4.99E-33 8.40E-32

PYHIN1 0.172445116 1.405146101 3.026510971 5.03E-33 8.47E-32

ENAM 4.079121432 1.221067202 -1.740115854 5.21E-33 8.75E-32

ARL11 0.224860272 0.865995408 1.945330583 5.21E-33 8.75E-32

LINC01704 0.033101467 0.309539735 3.225157545 5.23E-33 8.78E-32

AC040977.1 2.882706806 8.47776831 1.556260429 5.30E-33 8.89E-32

AC068299.2 0.013518085 0.316513021 4.549302177 5.32E-33 8.92E-32

RPL12P30 0.522060525 0.133013942 -1.972639604 5.38E-33 9.02E-32

RAB24 2.272892013 7.24519473 1.672495324 5.39E-33 9.03E-32

UCN 0.219488887 1.32043169 2.588789867 5.39E-33 9.03E-32

LINC01615 0.062430013 0.827937884 3.729210855 5.52E-33 9.23E-32

KCNE3 5.540530222 25.48950462 2.201807383 5.53E-33 9.24E-32

APLN 6.064477011 33.99312623 2.486787907 5.53E-33 9.24E-32

LINGO2 0.317902487 0.059198408 -2.424954023 5.58E-33 9.31E-32

CXorf58 0.144561737 0.040935575 -1.82025868 5.61E-33 9.36E-32

AC009542.2 0.119693804 1.622238799 3.760565827 5.63E-33 9.39E-32

AL031710.1 10.23423708 1.697657815 -2.59178596 5.68E-33 9.42E-32

TSPAN33 77.48068181 25.92135175 -1.579695695 5.68E-33 9.42E-32

TPI1P2 1.226154872 0.423937875 -1.532216445 5.68E-33 9.42E-32

MIR155HG 0.252438202 2.736779781 3.438477195 5.68E-33 9.42E-32

KCNMB2 1.154601965 0.262183158 -2.138748666 5.87E-33 9.73E-32

DISC1 0.326601612 1.055304684 1.692055773 5.87E-33 9.73E-32

AC073218.1 0.106479487 3.861052261 5.180346648 5.96E-33 9.85E-32

LHFPL2 1.99220945 7.892943931 1.986194168 6.03E-33 9.96E-32

SMIM25 0.257030096 1.518804929 2.562927387 6.13E-33 1.01E-31

DPP6 1.305551346 0.131778975 -3.308467081 6.18E-33 1.02E-31

AC022509.1 0.049703376 1.30890708 4.718875024 6.21E-33 1.03E-31

AC011700.1 0.130914724 0.012533998 -3.384708837 6.31E-33 1.04E-31

PIP5K1B 3.304663321 1.041505637 -1.665832649 6.40E-33 1.05E-31

MICALL2 0.797501278 3.332508266 2.063049718 6.45E-33 1.06E-31

ADGRE1 0.169917055 1.136049951 2.741123696 6.51E-33 1.07E-31

MFAP3L 17.05089861 5.216904484 -1.708581849 6.56E-33 1.08E-31

PCDHA12 0.467588693 0.113315522 -2.04489455 6.78E-33 1.11E-31

DCXR 64.1071605 14.67242986 -2.127377698 6.79E-33 1.11E-31

ASPM 0.137410003 0.653812804 2.250390605 6.79E-33 1.11E-31

SLC7A13 7.676463792 0.273152616 -4.812662735 6.87E-33 1.13E-31

SERPINA4 3.617165533 0.69144386 -2.387175596 6.87E-33 1.13E-31

LINC02857 0.192425818 0.023539724 -3.031133095 6.93E-33 1.13E-31

ABTB2 9.307763861 2.96728645 -1.649290404 7.03E-33 1.15E-31

DEPP1 27.14535215 129.6864656 2.256250819 7.03E-33 1.15E-31

LRRN2 6.521923876 1.189875536 -2.454486931 7.09E-33 1.16E-31

TAC1 0.879882936 0.111907125 -2.975009697 7.19E-33 1.17E-31

LRRC19 17.56810768 4.554621854 -1.947555617 7.21E-33 1.18E-31

LINC00943 0.008333374 0.137138417 4.040588223 7.30E-33 1.19E-31

PRR15L 21.49308224 4.877756824 -2.139582648 7.34E-33 1.19E-31

Z93403.1 0.530354106 0.073921824 -2.842883673 7.53E-33 1.22E-31

CD37 1.638342547 7.275088653 2.150727799 7.53E-33 1.22E-31

GMIP 1.874372654 5.701016745 1.604811427 7.66E-33 1.24E-31

C5orf58 0.022704551 0.18236657 3.005787899 7.69E-33 1.25E-31

AC126177.5 0.280092763 0.06789276 -2.04457506 7.69E-33 1.25E-31

ABCB6 0.641922867 2.206010897 1.780968058 7.72E-33 1.25E-31

LOXL2 3.709400017 23.0460262 2.63526025 7.85E-33 1.27E-31

OLFML2A 3.795180365 25.06617713 2.72350163 7.85E-33 1.27E-31

MRGPRF 5.654163 1.223246113 -2.208598776 7.92E-33 1.28E-31

SAMD3 0.082423135 0.433972173 2.396481297 8.06E-33 1.30E-31

AC117457.1 0.073541146 0.014912568 -2.302022948 8.12E-33 1.31E-31

ETV7 0.654395974 4.278064482 2.708722451 8.20E-33 1.32E-31

NUAK2 29.82674314 6.831295251 -2.126375402 8.27E-33 1.33E-31

LINC00871 0.639447053 0.04669285 -3.775551344 8.52E-33 1.37E-31

ANXA2R 0.471497819 1.925064552 2.029583819 8.55E-33 1.38E-31

AC008659.1 0.00667799 0.22113118 5.049344785 8.62E-33 1.39E-31

NTNG1 1.948527505 0.09022643 -4.432690382 8.71E-33 1.40E-31

INTS6L 0.546731767 1.918682509 1.811210896 8.93E-33 1.44E-31

CRHBP 13.7814243 0.877572875 -3.973062252 9.08E-33 1.46E-31

FKBP10 7.593420875 51.42166518 2.759554451 9.08E-33 1.46E-31

SIRPA 15.50138138 45.14401342 1.542137899 9.24E-33 1.48E-31

CYTH4 1.108724722 4.447694386 2.004156447 9.32E-33 1.49E-31

SELPLG 3.126343449 13.11657302 2.068842653 9.32E-33 1.49E-31

SOST 8.345863233 0.077795032 -6.745239445 9.48E-33 1.52E-31

SLC29A2 7.048646268 1.796930508 -1.971811589 1.01E-32 1.61E-31

APCDD1L-DT 1.075267417 0.332487014 -1.693325601 1.02E-32 1.63E-31

RGS6 0.352658451 0.074795991 -2.237238759 1.04E-32 1.66E-31

HROB 0.259770076 0.757906064 1.544783801 1.04E-32 1.66E-31

ACAP1 0.646466929 2.365560149 1.871533371 1.08E-32 1.71E-31

IFITM3P6 0.159306454 0.91761334 2.526081651 1.10E-32 1.74E-31

CHST11 2.084835428 7.156856489 1.779392546 1.13E-32 1.80E-31

AC108463.2 0.294182831 1.337661305 2.184927916 1.13E-32 1.80E-31

LINC02685 0.004890102 0.336092614 6.102850342 1.17E-32 1.86E-31

C20orf197 0.032228768 0.217471963 2.754408457 1.19E-32 1.89E-31

BEST1 0.245153189 0.912996022 1.896925047 1.20E-32 1.91E-31

FCRL6 0.193703582 1.134865804 2.550599173 1.21E-32 1.92E-31

PLEKHG2 1.843376396 6.261068511 1.764058205 1.22E-32 1.93E-31

LZTS1 1.570720407 8.352466607 2.41077591 1.22E-32 1.93E-31

AC156455.1 0.231366171 1.954357258 3.078444376 1.22E-32 1.93E-31

MISP3 8.414979389 2.579467593 -1.705886417 1.23E-32 1.95E-31

PNMA8A 13.29497021 3.630071438 -1.872810699 1.24E-32 1.96E-31

AGTR2 0.166363553 0.028171206 -2.562046144 1.32E-32 2.07E-31

TBX15 0.210030784 1.786312855 3.088312072 1.32E-32 2.07E-31

LINC02526 0.05494887 1.075609227 4.29092041 1.33E-32 2.09E-31

BICDL1 29.6328196 7.271615195 -2.026848146 1.33E-32 2.09E-31

RAB37 0.712586286 2.73703049 1.941474882 1.38E-32 2.16E-31

LPAR5 0.543109325 2.480662166 2.191410734 1.38E-32 2.16E-31

AC138123.1 0.906416282 0.204915971 -2.145141348 1.41E-32 2.21E-31

ARHGAP45 2.681118772 7.69766429 1.521585622 1.41E-32 2.21E-31

VWF 18.45550564 116.5560549 2.658900782 1.43E-32 2.23E-31

AC126177.6 0.327537524 0.071442626 -2.196803178 1.43E-32 2.24E-31

TMC4 26.97228763 6.029732434 -2.161311997 1.48E-32 2.30E-31

NPAP1 0.090722671 0.015269734 -2.570788149 1.50E-32 2.33E-31

KCNJ13 3.301384003 0.639376054 -2.368334338 1.52E-32 2.36E-31

PLEKHO1 2.423417603 8.300898816 1.776224528 1.53E-32 2.38E-31

AL355102.4 2.675754908 0.422136817 -2.664163409 1.53E-32 2.38E-31

AC018553.1 0.626430707 3.968911411 2.663516523 1.57E-32 2.43E-31

CD244 0.212198227 1.08118021 2.3491225 1.61E-32 2.49E-31

SLC12A5-AS1 0.009083954 0.100209555 3.463555867 1.61E-32 2.49E-31

DDX12P 0.107296925 0.762302203 2.828754317 1.63E-32 2.53E-31

LINC01018 1.2876631 0.257171604 -2.323951921 1.66E-32 2.57E-31

CD84 0.537409615 3.845515907 2.839083121 1.66E-32 2.57E-31

MESTIT1 0.170063553 0.033420815 -2.347255172 1.67E-32 2.58E-31

AC116036.2 1.1691855 0.381791097 -1.614648476 1.69E-32 2.61E-31

STAT4 0.389246365 1.453216963 1.900494638 1.72E-32 2.65E-31

HOXB5 7.3413065 1.866719336 -1.975531803 1.75E-32 2.69E-31

FCGR1CP 0.101521983 0.786443502 2.953550974 1.79E-32 2.75E-31

PLK1 0.307266584 1.247110122 2.021026077 1.81E-32 2.78E-31

CD2 1.81832654 15.03387378 3.047533585 1.83E-32 2.80E-31

CCL4 1.353069597 8.732162588 2.690102944 1.84E-32 2.82E-31

TCEAL6 0.225808889 0.025859151 -3.126355452 1.84E-32 2.82E-31

TPD52L1 7.446355847 2.146230678 -1.794729415 1.87E-32 2.86E-31

PRSS8 44.46200105 13.2332334 -1.748407268 1.87E-32 2.86E-31

CDKN2C 2.615416444 7.829170889 1.581818853 1.87E-32 2.86E-31

PPP1R3C 7.713945653 29.83253044 1.951345467 1.92E-32 2.93E-31

HMOX1 24.87096883 128.4014111 2.368126444 1.97E-32 3.00E-31

ITPRIPL1 0.168101055 0.588772803 1.808382251 1.99E-32 3.03E-31

PDYN-AS1 0.152858108 0.039064072 -1.968278831 2.00E-32 3.04E-31

NCF4 1.735199821 7.074388123 2.027503563 2.00E-32 3.04E-31

AP001094.2 0.090901885 0.643657052 2.823910088 2.00E-32 3.04E-31

SPARC 157.9616897 569.4551487 1.850007512 2.04E-32 3.09E-31

NTN4 90.44181 28.77264223 -1.652292153 2.06E-32 3.11E-31

NCCRP1 1.738726015 0.225455437 -2.947116404 2.07E-32 3.14E-31

SLC15A3 2.414404156 7.561034736 1.646916488 2.07E-32 3.14E-31

CRTAM 0.140274249 1.197757133 3.094013307 2.14E-32 3.24E-31

ITGB6 23.15368819 3.738364266 -2.630762964 2.16E-32 3.27E-31

POLQ 0.048249848 0.216002948 2.162454705 2.22E-32 3.35E-31

TTYH3 11.75810811 46.1347723 1.972198588 2.26E-32 3.40E-31

AC010307.4 0.716867117 0.149844047 -2.25824395 2.29E-32 3.45E-31

TOP2A 1.030254579 3.844895731 1.899943598 2.30E-32 3.45E-31

HLA-H 17.05623982 57.10324799 1.743273179 2.33E-32 3.51E-31

MSC 2.398311154 19.57436037 3.028874416 2.35E-32 3.54E-31

FMN2 0.642036671 0.049321672 -3.702362099 2.38E-32 3.58E-31

SCIN 23.36804397 5.544212033 -2.07548264 2.39E-32 3.59E-31

LINC00944 0.07658033 1.148177627 3.906228156 2.46E-32 3.70E-31

HSD17B7 1.106739867 3.497367602 1.659953279 2.48E-32 3.71E-31

AC103563.2 1.603528419 0.289782957 -2.46820527 2.54E-32 3.80E-31

MPP2 1.067437387 0.274902997 -1.957156906 2.54E-32 3.80E-31

AC087242.1 0.059528293 0.019379112 -1.619073059 2.59E-32 3.86E-31

AC108174.1 0.383624707 0.053503785 -2.841982788 2.60E-32 3.88E-31

CYP17A1 13.60738757 3.803805891 -1.838874584 2.61E-32 3.88E-31

PPM1E 0.922726528 0.323920601 -1.51026291 2.61E-32 3.88E-31

LRRC25 1.648140349 8.083466858 2.294135071 2.61E-32 3.88E-31

IL21R 0.180143879 1.136034379 2.656784954 2.61E-32 3.88E-31

NR1I3 1.192470162 0.217708335 -2.453484618 2.63E-32 3.91E-31

BRCA2 0.130868212 0.439986619 1.74934494 2.63E-32 3.91E-31

AC093627.7 2.051749119 0.162971142 -3.654165902 2.65E-32 3.94E-31

CHSY3 0.327480898 1.513704245 2.20860069 2.65E-32 3.94E-31

TNFRSF18 0.155185162 1.163310576 2.906173788 2.65E-32 3.94E-31

FRG2 0.075811148 0.004661271 -4.023614655 2.67E-32 3.97E-31

CD160 0.097055096 0.368600621 1.925182628 2.67E-32 3.97E-31

AC010200.1 0.00739949 0.190759475 4.688185014 2.69E-32 3.99E-31

ARHGEF26-AS1 0.071877975 0.022396007 -1.68230822 2.71E-32 4.01E-31

MTCL1 0.662110686 3.213721341 2.279100521 2.72E-32 4.02E-31

TICRR 0.063202576 0.250010358 1.983932613 2.77E-32 4.08E-31

SEMA5B 2.082418883 27.6203172 3.729397684 2.77E-32 4.08E-31

HLA-G 1.676810196 24.47187207 3.867333171 2.77E-32 4.08E-31

TUBA3D 1.026352915 8.410392006 3.034646153 2.79E-32 4.11E-31

IFI16 7.022676042 21.35546167 1.604512297 2.81E-32 4.14E-31

RRM2 0.665280767 2.89982108 2.123928657 2.81E-32 4.14E-31

PIMREG 0.161342184 0.779358344 2.272163135 2.81E-32 4.14E-31

AL031123.2 0.85046066 0.229017389 -1.892787359 2.88E-32 4.24E-31

AC005682.1 0.192191314 1.129398828 2.55494 2.91E-32 4.27E-31

DLGAP5 0.248132319 0.993828229 2.001886866 2.98E-32 4.37E-31

LINC02678 0.085151695 0.766997604 3.171114923 3.05E-32 4.47E-31

ENPP3 3.092035128 78.62046653 4.66827631 3.06E-32 4.48E-31

AL121578.2 0.092253193 0.027422511 -1.750238143 3.08E-32 4.50E-31

FTH1P22 0.09501723 1.461906877 3.943518449 3.13E-32 4.58E-31

MOGAT2 0.562337691 0.029481677 -4.253546258 3.16E-32 4.61E-31

SLC1A3 0.5760844 3.952321389 2.77834817 3.19E-32 4.66E-31

CASP5 0.03871163 0.30118713 2.959821148 3.19E-32 4.66E-31

DLGAP2 0.202261921 0.034972972 -2.531912443 3.22E-32 4.69E-31

ALDH1A2 6.188338667 1.911341814 -1.694966347 3.22E-32 4.69E-31

HAPLN3 0.602839985 3.170850215 2.395022714 3.22E-32 4.69E-31

MYH14 9.389518944 2.638070607 -1.831568067 3.28E-32 4.77E-31

AC006357.1 0.060169733 0.008068305 -2.898700388 3.34E-32 4.86E-31

KIF14 0.084313681 0.312318041 1.88917725 3.36E-32 4.89E-31

TRIP13 0.459293692 1.571612808 1.774756957 3.39E-32 4.93E-31

EMP3 12.27501342 39.12582339 1.672396512 3.42E-32 4.96E-31

MUC3A 0.199117942 2.076072848 3.382161936 3.45E-32 5.00E-31

OIP5 0.289743232 0.861085187 1.571381008 3.47E-32 5.04E-31

LINC02417 1.58550095 0.1245831 -3.66975846 3.51E-32 5.09E-31

AC124861.1 0.277038319 0.034127334 -3.021085925 3.51E-32 5.09E-31

AL117335.1 0.307360112 2.085682196 2.762517492 3.56E-32 5.16E-31

LNX1 7.000729417 2.436167202 -1.522892093 3.59E-32 5.20E-31

AL049839.2 0.192372613 0.049010279 -1.972747148 3.60E-32 5.21E-31

AP005233.2 0.028870837 4.031178858 7.125445033 3.61E-32 5.22E-31

MS4A6A 3.065113829 14.92628491 2.283842572 3.62E-32 5.24E-31

GZMB 0.760945961 4.539312802 2.576607999 3.66E-32 5.28E-31

AC025580.3 0.051429403 0.798072769 3.955854993 3.67E-32 5.30E-31

HCG27 0.202245276 1.578450712 2.964331305 3.72E-32 5.36E-31

CDK18 9.204546861 48.65050373 2.402036138 3.75E-32 5.40E-31

MYBPH 0.749615097 0.079225133 -3.242119919 3.77E-32 5.43E-31

LMO7-AS1 3.659250473 0.681240102 -2.425312901 3.78E-32 5.44E-31

MALRD1 0.239121156 0.064699851 -1.885907474 3.79E-32 5.45E-31

DPP9-AS1 0.027380636 0.253833116 3.212652415 3.88E-32 5.57E-31

AC073115.1 0.0405357 2.249831888 5.794480338 3.97E-32 5.70E-31

AP000894.4 3.705652086 1.14408092 -1.69553834 4.01E-32 5.76E-31

SVOPL 0.540671419 0.113808786 -2.248140156 4.18E-32 5.99E-31

AC137932.2 0.071188117 0.368174783 2.370682473 4.18E-32 5.99E-31

LINC01020 2.262612016 0.028810844 -6.295233491 4.22E-32 6.04E-31

SGK2 13.74532426 4.641130054 -1.566392911 4.26E-32 6.09E-31

S1PR5 0.299206805 1.603826287 2.422302997 4.29E-32 6.13E-31

FBXO17 8.267283444 24.7774875 1.583544646 4.33E-32 6.17E-31

AC009133.1 0.80922559 2.517896361 1.637605053 4.33E-32 6.17E-31

APBB1IP 2.817833142 15.2176924 2.433091516 4.33E-32 6.17E-31

CNGA1 3.16827715 0.5471117 -2.533791229 4.37E-32 6.21E-31

LSP1 2.140109313 10.15848706 2.246929158 4.37E-32 6.21E-31

CXCL9 2.611677277 32.86933179 3.653693587 4.37E-32 6.21E-31

TACR3 0.168607374 0.01170598 -3.848350048 4.48E-32 6.37E-31

PCAT14 0.117672939 0.013060401 -3.171511466 4.52E-32 6.43E-31

SSTR5 1.622891918 0.109789717 -3.88575208 4.63E-32 6.57E-31

KIF20A 0.388391498 1.941276177 2.321421852 4.63E-32 6.57E-31

MYO3A 0.144442468 1.491503332 3.368200321 4.63E-32 6.57E-31

DEPDC1 0.125705224 0.487340381 1.95488516 4.67E-32 6.62E-31

CETN4P 0.88187175 0.241878598 -1.866285739 4.83E-32 6.84E-31

BIRC7 0.071508884 9.151525041 6.999745885 4.94E-32 6.99E-31

C11orf21 0.091988453 0.598313302 2.701376461 4.95E-32 7.02E-31

TNNI2 0.132532006 0.703935369 2.409102169 5.02E-32 7.10E-31

SOD3 50.24434181 17.101312 -1.554854127 5.04E-32 7.13E-31

PCED1B 1.170480503 3.836522289 1.712698234 5.08E-32 7.19E-31

ATG16L2 0.670101101 3.480230744 2.376732278 5.17E-32 7.31E-31

CKAP2L 0.160201086 0.623318241 1.960085006 5.26E-32 7.42E-31

AC007938.1 0.101852446 0.019259989 -2.402801848 5.26E-32 7.43E-31

HUNK 4.698769292 1.440625397 -1.705587691 5.34E-32 7.54E-31

PDIA5 5.542858264 18.6799957 1.752792101 5.34E-32 7.54E-31

AC011352.3 0.093314493 3.739439559 5.32457709 5.36E-32 7.55E-31

APOBEC3C 5.735219014 24.61184262 2.101432187 5.44E-32 7.66E-31

CLDN11 2.237101722 0.395328977 -2.500505246 5.48E-32 7.72E-31

RAB3B 0.634474758 0.141940666 -2.160274784 5.72E-32 8.04E-31

LINC01751 0.005015245 0.175787389 5.131367511 5.76E-32 8.09E-31

FCHO1 0.184007747 1.127399557 2.615160491 5.86E-32 8.23E-31

DNASE1 6.917358171 1.228918906 -2.492831441 5.91E-32 8.30E-31

AC078906.1 0.016378799 0.341561031 4.382241881 5.99E-32 8.41E-31

VAT1L 6.471405132 1.085896058 -2.575192982 6.01E-32 8.42E-31

MYBL1 0.341752438 1.169402929 1.774748578 6.01E-32 8.42E-31

KIF21B 0.185122881 0.835087992 2.173445003 6.06E-32 8.49E-31

MGAT4C 0.029615545 0.008383095 -1.820799688 6.12E-32 8.56E-31

RNF175 0.083770215 0.437738403 2.385559674 6.22E-32 8.69E-31

AC006064.3 0.165555254 1.109641934 2.744709517 6.22E-32 8.69E-31

XK 0.965144899 0.257089546 -1.908474607 6.38E-32 8.90E-31

AC018541.1 1.588500415 0.146668823 -3.437031325 6.49E-32 9.04E-31

CSTA 0.574392105 2.820911127 2.296053391 6.88E-32 9.57E-31

AC010442.1 48.5093381 8.981723355 -2.433198303 7.00E-32 9.72E-31

CDKL1 3.044271851 1.058873901 -1.523566404 7.00E-32 9.72E-31

ZNF469 0.10559336 0.450659405 2.093518389 7.00E-32 9.72E-31

LINC01275 0.056829079 0.354454687 2.640899957 7.05E-32 9.79E-31

HLA-DPB1 44.02344333 186.7294374 2.084605486 7.30E-32 1.01E-30

AC011899.3 0.041002606 0.317017231 2.950773755 7.39E-32 1.02E-30

NCKAP1L 1.048964601 5.351423428 2.350956693 7.42E-32 1.02E-30

LINC01358 0.244871587 3.127218933 3.674782937 7.41E-32 1.02E-30

MAP4K1 0.514946375 2.758239705 2.421253734 7.55E-32 1.04E-30

ADAMDEC1 0.134989204 2.665691681 4.303593988 7.61E-32 1.05E-30

BRIP1 0.119980233 0.433330819 1.852672104 7.61E-32 1.05E-30

RAD51 0.275551534 0.835361447 1.600078402 7.68E-32 1.06E-30

ACAA1 20.18380894 6.799000942 -1.56980378 7.74E-32 1.06E-30

FBLN5 41.056555 12.51781969 -1.713629277 7.81E-32 1.07E-30

SHISA3 28.26140581 6.635045083 -2.090655058 7.87E-32 1.08E-30

DLL4 3.828581707 20.8556227 2.445554437 7.87E-32 1.08E-30

CD8A 0.902661944 11.31597051 3.648030728 7.87E-32 1.08E-30

NCR1 0.050670735 0.327064827 2.690351947 8.14E-32 1.12E-30

AL731533.2 0.979157867 4.530346454 2.210007998 8.21E-32 1.12E-30

RSPO1 0.232633393 0.030163875 -2.947164507 8.24E-32 1.13E-30

TGFB1 14.84169029 43.80108268 1.561311124 8.28E-32 1.13E-30

GGTA1P 1.365545429 5.234507678 1.938576543 8.28E-32 1.13E-30

CDH8 0.015745156 0.180669227 3.520370847 8.42E-32 1.15E-30

IL16 0.589597089 2.210966514 1.906875869 8.63E-32 1.18E-30

PNMT 0.705595881 0.116488373 -2.598656181 8.69E-32 1.18E-30

ITLN2 0.119228146 0.025637024 -2.217424121 8.72E-32 1.19E-30

UBTFL10 0.201199462 0.006605976 -4.928710949 8.82E-32 1.20E-30

PPP1R1A 50.03813301 7.079538523 -2.821300735 8.85E-32 1.20E-30

AL008733.1 0.113651861 0.009052417 -3.650174522 9.05E-32 1.23E-30

AC105411.1 0.034116397 0.191165049 2.486281577 9.08E-32 1.23E-30

SIX1 0.08427676 0.452755343 2.425524915 9.31E-32 1.26E-30

CCR5 0.782307784 5.954670484 2.92821345 9.31E-32 1.26E-30

UGT8 24.84969878 7.249328525 -1.777309088 9.39E-32 1.27E-30

MMP2-AS1 0.020771671 0.620347168 4.900386635 9.41E-32 1.27E-30

PDGFRA 7.540041465 1.449602308 -2.3789153 9.55E-32 1.29E-30

ITGA5 7.631388542 29.35314983 1.943497838 9.55E-32 1.29E-30

CAPN12 0.563160472 5.200120577 3.206927095 9.63E-32 1.30E-30

HSD3B7 6.777910042 31.08186301 2.197160585 9.71E-32 1.31E-30

TRPA1 0.12684434 1.177720178 3.214865746 9.75E-32 1.32E-30

NLRC4 0.380052118 1.354477894 1.833467669 1.00E-31 1.35E-30

CD7 0.477295232 3.960605369 3.052767131 1.00E-31 1.35E-30

CD300C 0.445935792 2.184710382 2.292534136 1.01E-31 1.36E-30

AC091152.4 0.171667708 0.049532409 -1.793173995 1.03E-31 1.38E-30

CLEC12A 0.197688489 1.434497766 2.859244951 1.03E-31 1.38E-30

TRDC 0.536539903 2.540657403 2.243444473 1.04E-31 1.39E-30

KCNMA1-AS1 0.002736028 0.063132628 4.528230862 1.04E-31 1.39E-30

CXCL10 3.230415582 35.63265339 3.463408238 1.08E-31 1.45E-30

ABCC3 4.103354433 20.70616958 2.335185015 1.10E-31 1.48E-30

KRT7 35.15174872 10.83917463 -1.697341558 1.11E-31 1.49E-30

GACAT2 0.076221918 3.076454337 5.334918867 1.11E-31 1.49E-30

CYP39A1 3.964899986 1.254855272 -1.659763493 1.12E-31 1.50E-30

ARHGAP30 1.908123682 7.969489049 2.062332543 1.15E-31 1.53E-30

AL121974.1 0.509430146 0.109175322 -2.222237549 1.18E-31 1.57E-30

AC008555.2 0.034920106 0.195703983 2.486543276 1.19E-31 1.58E-30

OPN4 0.00336394 0.18188877 5.756760661 1.23E-31 1.64E-30

ANXA3 8.501242389 2.065835753 -2.04094814 1.24E-31 1.65E-30

METTL26 13.5204699 39.33778121 1.540770294 1.25E-31 1.66E-30

LINC01159 2.26319711 0.447639246 -2.337953807 1.28E-31 1.70E-30

MKI67 0.406896226 1.886047409 2.212633135 1.31E-31 1.74E-30

CHL1-AS1 0.14116662 0.03850972 -1.874104474 1.32E-31 1.75E-30

LINC02226 0.03544117 0.005783636 -2.615377476 1.33E-31 1.76E-30

ACKR3 8.768886819 42.47723586 2.276224274 1.34E-31 1.77E-30

NCF1 0.251276641 1.252014292 2.316902564 1.34E-31 1.77E-30

ZBP1 0.070055515 0.526008935 2.908516772 1.37E-31 1.81E-30

IL10RA 1.7733518 8.547340509 2.268996829 1.42E-31 1.87E-30

LDHD 27.38050605 5.644840111 -2.278144488 1.43E-31 1.88E-30

SCN3A 0.416593518 0.136774653 -1.606839497 1.45E-31 1.91E-30

MCAM 15.03714426 63.34914728 2.074794592 1.48E-31 1.94E-30

AFAP1L2 17.40745456 4.801922818 -1.858021137 1.51E-31 1.99E-30

RIPPLY1 1.584640428 0.223548539 -2.825495493 1.54E-31 2.02E-30

AC005041.3 0.172677064 0.583222112 1.755968947 1.55E-31 2.04E-30

TNFSF13B 1.005474456 5.701772058 2.503533935 1.57E-31 2.05E-30

NMB 4.496406361 26.26324022 2.54620059 1.59E-31 2.09E-30

ICOS 0.068278474 0.728396481 3.415221227 1.60E-31 2.09E-30

ADPGK-AS1 0.01908175 0.085412615 2.162255714 1.62E-31 2.12E-30

C21orf62 5.731995479 1.247830136 -2.199615915 1.63E-31 2.13E-30

AC009549.1 0.273816426 1.40451562 2.358791766 1.63E-31 2.13E-30

GNRH1 0.178043116 1.28296754 2.849186111 1.65E-31 2.15E-30

ST3GAL6-AS1 2.028861533 0.556074906 -1.867319269 1.75E-31 2.28E-30

AC020905.1 0.064139355 0.007437146 -3.10838884 1.77E-31 2.30E-30

AL137025.1 0.172195261 0.051446321 -1.742905634 1.81E-31 2.35E-30

CDKN3 0.414006472 1.510654293 1.867448318 1.85E-31 2.41E-30

CXCR6 0.308036263 2.060846095 2.742064663 1.85E-31 2.41E-30

PIF1 0.066551359 0.327408946 2.298553708 1.86E-31 2.41E-30

CD6 0.379998834 1.976623131 2.378970932 1.88E-31 2.44E-30

AC015540.1 0.037055725 0.004802734 -2.947768632 1.93E-31 2.50E-30

SNHG1 2.656819181 8.303979614 1.644102859 1.93E-31 2.50E-30

LINC00861 0.089858191 0.728956025 3.020109864 1.93E-31 2.50E-30

EBF2 0.318784504 2.134153921 2.743010823 1.95E-31 2.52E-30

AP002518.1 0.005338756 0.093865667 4.136021926 1.96E-31 2.53E-30

SLC6A3 0.593973177 42.62205684 6.165058623 1.96E-31 2.54E-30

AL022069.1 0.455974453 0.079161755 -2.52607749 1.99E-31 2.57E-30

AC004585.1 0.089846222 0.922131088 3.359442114 2.00E-31 2.59E-30

LINC01546 0.026377838 0.231946237 3.136392198 2.03E-31 2.63E-30

DNM1P47 0.009500397 0.067652815 2.83209028 2.04E-31 2.63E-30

MUC1 62.08031639 20.48334161 -1.599684817 2.05E-31 2.64E-30

INSRR 1.049377112 0.086549548 -3.59986314 2.06E-31 2.66E-30

OR51E1 0.423612902 2.478632567 2.548725986 2.08E-31 2.68E-30

HOGA1 21.11118324 5.717697668 -1.884501198 2.11E-31 2.72E-30

SNX20 0.342532793 1.902300351 2.473431029 2.11E-31 2.72E-30

SLAMF8 1.087885257 7.058921058 2.697921289 2.11E-31 2.72E-30

AC023154.1 1.457783421 0.036428063 -5.322582286 2.12E-31 2.72E-30

AC005831.1 0.239123886 1.071510317 2.163815589 2.13E-31 2.74E-30

DLGAP3 0.438954883 0.144681854 -1.601188669 2.17E-31 2.78E-30

DDIT4 32.32598861 145.0282952 2.165568002 2.24E-31 2.87E-30

MEIOC 0.193521362 0.063561036 -1.606278295 2.28E-31 2.92E-30

TSPYL5 12.62530863 4.062507463 -1.635876286 2.32E-31 2.97E-30

PALM3 15.46477183 4.164686495 -1.892705745 2.40E-31 3.06E-30

GPRIN1 0.307153246 1.369553214 2.156674787 2.44E-31 3.11E-30

NOD2 0.166210027 0.927548648 2.480415529 2.52E-31 3.21E-30

AC087468.1 0.115020676 2.031027506 4.142244648 2.54E-31 3.24E-30

HMGCS2 108.9225706 17.51671484 -2.6364988 2.58E-31 3.29E-30

C3orf52 2.357702238 0.598380986 -1.978245289 2.58E-31 3.29E-30

PLIN2 26.37278014 208.070087 2.979948003 2.63E-31 3.34E-30

AC040162.1 0.1188522 0.524543404 2.141893542 2.67E-31 3.39E-30

CASC22 0.071364109 0.016866571 -2.081031977 2.74E-31 3.48E-30

KCNH1 0.085635601 0.025140216 -1.768213636 2.78E-31 3.53E-30

ABCA1 3.708622333 11.2231736 1.597525424 2.81E-31 3.56E-30

HLA-J 1.269080317 6.961896228 2.455696934 2.81E-31 3.56E-30

HPCAL1 10.36381203 37.4564472 1.85365931 2.83E-31 3.59E-30

RNU6-796P 0.206706668 3.148393893 3.928959213 2.83E-31 3.59E-30

HSPA6 0.654532543 2.747032921 2.069337371 2.90E-31 3.67E-30

CLEC7A 0.744322863 3.934988063 2.402358804 2.90E-31 3.67E-30

CGREF1 0.912822861 7.771962178 3.089872051 2.93E-31 3.70E-30

SCD5 30.30366839 9.379611275 -1.69189241 2.98E-31 3.76E-30

SLC5A7 0.220128864 0.012111992 -4.183840216 3.04E-31 3.84E-30

SIGLEC1 0.553293826 3.309869657 2.580656675 3.08E-31 3.88E-30

LINC02224 0.054474178 0.007470917 -2.866215346 3.11E-31 3.92E-30

FGF1 26.41248142 1.479408148 -4.158127816 3.23E-31 4.07E-30

LINC01357 0.030276578 0.318187126 3.393601381 3.25E-31 4.09E-30

AL590764.1 0.153930179 0.753471698 2.291277215 3.26E-31 4.10E-30

KITLG 21.52851915 6.954415999 -1.630247813 3.34E-31 4.20E-30

AL590666.2 0.486509766 3.37347872 2.793696391 3.37E-31 4.23E-30

AC023024.1 0.244518768 1.823466286 2.898666419 3.37E-31 4.23E-30

SIRPB2 0.30079028 1.351912262 2.168171671 3.46E-31 4.33E-30

SLC44A4 29.69985932 8.055449093 -1.882419171 3.52E-31 4.40E-30

CXCR3 0.371623525 3.382527768 3.18618804 3.52E-31 4.40E-30

LBX2 0.120267261 0.506846629 2.075305285 3.60E-31 4.50E-30

FA2H 3.169834805 0.86258338 -1.877671832 3.63E-31 4.53E-30

LOX 5.912242657 63.54692831 3.426045002 3.63E-31 4.53E-30

BCL2A1 0.734244986 4.224732377 2.52452654 3.66E-31 4.57E-30

PPP4R4 0.211921715 0.057839301 -1.873409406 3.73E-31 4.64E-30

AP004247.2 0.013860291 0.213278595 3.943709724 3.75E-31 4.66E-30

AC099329.2 0.246749163 0.072722717 -1.762567174 3.75E-31 4.67E-30

ELOVL2 0.148109133 1.400430385 3.24113776 3.88E-31 4.82E-30

LONRF2 1.437745035 0.395287688 -1.862832931 3.98E-31 4.94E-30

ZNF37CP 0.181999683 2.785514859 3.935936167 4.04E-31 5.02E-30

PLCB2 0.808865414 3.622450668 2.162994463 4.12E-31 5.10E-30

SLC38A3 1.429770997 0.437433077 -1.708649871 4.21E-31 5.21E-30

AL096799.1 0.226582897 5.78237663 4.67355171 4.33E-31 5.35E-30

AC100860.1 0.251872551 0.018716951 -3.750276531 4.37E-31 5.40E-30

PLEKHN1 0.216677001 1.178643091 2.443508983 4.40E-31 5.43E-30

SH2D2A 0.393381143 2.600390207 2.724728417 4.40E-31 5.43E-30

AL512785.1 0.169599636 0.04018105 -2.077545915 4.59E-31 5.65E-30

NT5DC3 1.112540356 5.33735589 2.262267544 4.59E-31 5.65E-30

XKR4 0.085789157 0.02784934 -1.623152189 4.63E-31 5.69E-30

RGS10 6.044428903 18.56216488 1.61868704 4.70E-31 5.77E-30

AC011352.1 0.04359626 1.920661092 5.461254773 4.77E-31 5.85E-30

STK33 1.609813536 0.511475146 -1.654157548 4.82E-31 5.91E-30

PARP15 0.095127862 0.638959801 2.74778531 4.82E-31 5.91E-30

HPCA 0.124252004 0.842036195 2.760613134 4.86E-31 5.96E-30

MS4A4E 0.081544727 0.773823091 3.24634029 4.90E-31 6.00E-30

UCHL1 34.07244613 8.608046433 -1.98484776 4.98E-31 6.10E-30

TRNP1 10.33964227 2.56999227 -2.008350346 5.07E-31 6.19E-30

LINC00881 0.014347943 0.207719598 3.8557215 5.09E-31 6.22E-30

CADM2 0.072501317 0.018995735 -1.932331641 5.23E-31 6.39E-30

TRBC1 0.107523008 0.793897945 2.884308154 5.32E-31 6.49E-30

NKAIN1 0.087297619 0.902048153 3.369190245 5.32E-31 6.49E-30

AC022905.1 0.013860233 0.18273459 3.72072628 5.51E-31 6.72E-30

RHOBTB3 14.44933147 4.872251806 -1.568342144 5.55E-31 6.76E-30

ATRNL1 1.41614039 0.36887733 -1.940751262 5.60E-31 6.81E-30

HCAR1 1.33583617 0.413246465 -1.692668698 5.60E-31 6.81E-30

SLC39A4 7.309359653 2.503666145 -1.545702826 5.69E-31 6.92E-30

AC008079.1 0.015465565 0.077161358 2.318818993 5.78E-31 7.02E-30

FAM217A 0.171379088 0.036831657 -2.218172871 5.83E-31 7.08E-30

IGSF11 1.535052745 0.219585815 -2.805431463 6.03E-31 7.31E-30

FAR2P3 0.111863562 0.02845146 -1.975165562 6.06E-31 7.34E-30

SYTL5 0.89612689 0.287677011 -1.639253093 6.08E-31 7.36E-30

PNMA2 2.686043901 15.43642934 2.522784286 6.08E-31 7.36E-30

STOX1 1.882333121 0.567703606 -1.729312156 6.23E-31 7.53E-30

SMPDL3A 12.82003822 46.65686771 1.863688893 6.23E-31 7.53E-30

BATF3 0.34201229 1.501586129 2.134367154 6.23E-31 7.53E-30

AL133466.1 0.320063713 0.028249976 -3.502037595 6.24E-31 7.53E-30

AL606519.1 0.193411749 0.015402737 -3.650416829 6.36E-31 7.67E-30

OR7E90P 0.049165636 0.005254427 -3.226044889 6.44E-31 7.77E-30

LINC02747 0.151633392 11.75354171 6.276364246 6.46E-31 7.79E-30

AC004066.1 0.136998055 0.03124184 -2.132604104 6.64E-31 7.99E-30

TNFRSF9 0.093367998 1.623367798 4.119917946 6.71E-31 8.07E-30

TNK2-AS1 0.02102777 0.1098941 2.385746177 6.76E-31 8.13E-30

TMEM44 1.504340865 5.175391077 1.782536382 6.83E-31 8.20E-30

PLXND1 9.233672639 28.41906014 1.621882353 6.88E-31 8.27E-30

AL023653.1 0.087700191 0.540457459 2.623529174 6.89E-31 8.27E-30

FFAR4 0.06821887 0.476398208 2.803925232 6.91E-31 8.29E-30

TBX19 0.492261961 1.488782934 1.596635256 7.11E-31 8.53E-30

RASGRP4 0.268728011 0.926070755 1.784975716 7.29E-31 8.74E-30

NDUFB4P11 0.252445384 0.064782549 -1.962294153 7.45E-31 8.92E-30

ASB15 1.518333427 0.047306114 -5.004318171 7.72E-31 9.22E-30

MYMK 0.471131861 0.048811923 -3.270825413 7.80E-31 9.31E-30

RNF212B 2.278186778 0.136962508 -4.0560331 7.85E-31 9.37E-30

AC015911.3 0.100496825 0.821778501 3.031599658 7.92E-31 9.43E-30

PAQR4 0.73026084 2.427552069 1.733018466 7.92E-31 9.44E-30

CXCR2P1 0.083254558 2.038551653 4.613871437 8.02E-31 9.55E-30

AC027309.1 0.855457706 0.048682669 -4.135216349 8.10E-31 9.65E-30

ANKRD62P1-PARP4P3 0.043717686 0.008542192 -2.355538823 8.12E-31 9.66E-30

AL118508.1 0.400767311 0.064990506 -2.624463944 8.26E-31 9.81E-30

LIMD2 1.263923631 5.080647671 2.007103126 8.39E-31 9.97E-30

SLFN12L 0.085869018 0.590092278 2.780730987 8.39E-31 9.97E-30

CHRNA1 0.009914517 0.424061477 5.418587067 8.61E-31 1.02E-29

LINC01924 0.158240686 0.051799459 -1.611111637 8.76E-31 1.04E-29

ADTRP 3.606491113 1.116644583 -1.691425805 8.97E-31 1.06E-29

ADAMTS16 3.34860144 0.546467058 -2.615352236 9.12E-31 1.08E-29

NCF1C 0.360334308 1.750387695 2.280266577 9.19E-31 1.09E-29

AC023024.2 0.391276198 3.680516616 3.233649027 9.74E-31 1.15E-29

CASC11 0.0047544 0.030352407 2.674475853 9.84E-31 1.16E-29

BARX2 2.129813957 13.56773695 2.671380785 9.90E-31 1.17E-29

PSORS1C2 0.028610678 0.396213274 3.791651622 9.93E-31 1.17E-29

NCF1B 0.169723001 0.818902808 2.270510142 1.01E-30 1.19E-29

SFXN2 9.008744128 1.860068199 -2.275970481 1.03E-30 1.21E-29

PPP1R42 0.23661985 0.075453613 -1.648909222 1.06E-30 1.25E-29

LINC01480 0.101911092 0.678030913 2.734039975 1.08E-30 1.26E-29

CCL18 0.240432572 12.12416365 5.656109063 1.08E-30 1.27E-29

ALX1 1.673991251 0.188210374 -3.152873929 1.09E-30 1.27E-29

U62317.1 0.174245529 1.083324874 2.636272412 1.10E-30 1.29E-29

GPR27 3.575302901 1.011471444 -1.821609882 1.12E-30 1.31E-29

NPNT 36.50304207 9.270894031 -1.977236324 1.14E-30 1.33E-29

PYCARD 2.459117529 9.994204781 2.022951091 1.14E-30 1.33E-29

RNF157-AS1 1.457353044 0.327546108 -2.153580504 1.15E-30 1.34E-29

AC005498.2 0.264467026 0.092202816 -1.520205134 1.15E-30 1.34E-29

CYP2J2 1.437270426 52.21073534 5.182943038 1.17E-30 1.36E-29

AP000864.2 0.032410171 0.701472455 4.435867925 1.17E-30 1.37E-29

IL18BP 2.916749444 8.354901034 1.518261279 1.23E-30 1.43E-29

CXCL11 0.435641105 5.137675006 3.55990364 1.24E-30 1.44E-29

LINC00158 0.007937523 0.1749886 4.462428287 1.24E-30 1.45E-29

AC243962.1 0.028647656 0.321506163 3.488357402 1.26E-30 1.47E-29

GCNT4 3.427825563 1.129813953 -1.601208471 1.28E-30 1.49E-29

AC105227.1 0.124772914 0.009133469 -3.771997999 1.28E-30 1.49E-29

PRELID3A 0.239516872 0.742347295 1.631967 1.29E-30 1.50E-29

MILR1 0.7388053 2.976665446 2.010430962 1.31E-30 1.52E-29

CD74 419.7033403 1411.712739 1.750004703 1.32E-30 1.53E-29

UBASH3A 0.154910564 1.108511247 2.839115972 1.32E-30 1.53E-29

STRA8 0.277139073 2.941844627 3.408039022 1.32E-30 1.53E-29

CD33 0.283466928 1.113944592 1.974425142 1.34E-30 1.56E-29

RNU6-339P 0.278813563 4.039082535 3.856654975 1.36E-30 1.57E-29

HAPLN1 0.244811734 2.782122133 3.50644114 1.39E-30 1.61E-29

CD200 4.497830722 14.55637593 1.694349945 1.41E-30 1.63E-29

ITGAM 1.19621866 4.879095226 2.028132514 1.47E-30 1.70E-29

CCDC13-AS1 0.52312596 0.160503533 -1.704553314 1.49E-30 1.72E-29

OGDHL 64.02705629 16.169257 -1.985428296 1.51E-30 1.74E-29

PLCXD2 2.924746104 0.994981408 -1.555569917 1.51E-30 1.74E-29

AD000864.1 0.08821762 0.337801391 1.937036524 1.51E-30 1.74E-29

PRR7 0.341700545 1.745356908 2.352717628 1.51E-30 1.74E-29

SH3GL1P1 0.054448781 0.310310922 2.510742822 1.56E-30 1.79E-29

WNT8B 0.64252046 0.046007851 -3.803790431 1.58E-30 1.81E-29

ERCC6L 0.086382909 0.250520941 1.536113393 1.58E-30 1.82E-29

DOK7 1.273456439 0.235278725 -2.436306835 1.60E-30 1.84E-29

RASSF4 6.40596505 21.56521928 1.751218554 1.62E-30 1.86E-29

CD86 1.224751815 5.447647688 2.153143974 1.68E-30 1.92E-29

AC135050.3 0.160602104 1.482246647 3.206222835 1.68E-30 1.92E-29

PRR11 0.432956225 1.614095739 1.898433082 1.69E-30 1.93E-29

ANLN 0.522617155 2.123145237 2.022376677 1.69E-30 1.93E-29

C6orf223 1.300641026 15.03410088 3.530943847 1.71E-30 1.95E-29

KIAA0895L 1.434815147 6.152614144 2.100334637 1.72E-30 1.96E-29

AC008892.1 0.190136558 0.027517872 -2.78859516 1.75E-30 2.00E-29

AC138207.6 0.05887014 0.241793042 2.038164763 1.79E-30 2.04E-29

TSPAN32 0.154131487 0.642675757 2.059929439 1.82E-30 2.08E-29

E2F7 0.079399376 0.336965231 2.085400157 1.85E-30 2.11E-29

ADAMTS14 0.066169847 0.675236999 3.35114811 1.87E-30 2.13E-29

CTXND1 1.876877788 0.446013579 -2.073175174 1.91E-30 2.18E-29

CSF2RA 0.940421503 3.654180793 1.958168582 1.91E-30 2.18E-29

GFI1 0.164254187 0.947006789 2.527444623 1.98E-30 2.25E-29

PGGT1BP1 0.135476779 0.022289235 -2.603626555 1.98E-30 2.25E-29

AL031595.2 0.001643276 0.019333793 3.556478374 2.00E-30 2.27E-29

SCN7A 0.645395992 0.043240569 -3.899727192 2.11E-30 2.39E-29

TRBV28 1.116786232 8.664480758 2.955760234 2.11E-30 2.39E-29

EMILIN2 1.160915785 3.687760389 1.6674816 2.18E-30 2.46E-29

FCGR2A 2.956442069 10.95811033 1.890065121 2.18E-30 2.46E-29

FXYD5 8.884865472 29.48373252 1.730497338 2.20E-30 2.48E-29

TESPA1 0.195959615 1.094556215 2.481717786 2.25E-30 2.54E-29

HTR6 0.030121315 1.066320661 5.145712768 2.31E-30 2.60E-29

CLEC4A 1.019567549 3.370871741 1.725164375 2.33E-30 2.62E-29

PBK 0.378974348 1.304672361 1.78351545 2.33E-30 2.62E-29

CD4 6.587828292 23.21248573 1.817026166 2.33E-30 2.62E-29

MZT2A 3.393943589 9.956934727 1.552739087 2.35E-30 2.64E-29

GAL3ST1 6.995447208 53.04403877 2.922702432 2.39E-30 2.69E-29

HOXA7 3.380404974 1.194312178 -1.501016104 2.41E-30 2.71E-29

MLC1 0.053651164 0.253570775 2.240707108 2.41E-30 2.71E-29

AF186192.2 1.21758524 0.421162667 -1.531573313 2.55E-30 2.86E-29

AC005703.6 0.084068749 0.288222238 1.777540136 2.55E-30 2.86E-29

CENPF 0.358114201 1.24944129 1.802791479 2.55E-30 2.86E-29

TNIP3 0.033819218 0.436745959 3.690879135 2.59E-30 2.90E-29

ASPHD1 0.907101764 5.709440909 2.654013164 2.66E-30 2.97E-29

TLCD3B 0.006646536 0.236835249 5.155137319 2.68E-30 2.99E-29

TTK 0.203862099 0.599042881 1.555065698 2.70E-30 3.02E-29

FAXC 0.379651241 0.119333434 -1.669676426 2.77E-30 3.09E-29

PWRN1 0.279452267 0.051263218 -2.446605914 2.77E-30 3.09E-29

CRYAA2 0.335000242 0.023077444 -3.859606791 2.77E-30 3.09E-29

GATA2 9.248065569 3.141012732 -1.557921836 2.79E-30 3.11E-29

KRT36 0.005335592 0.072962119 3.773427376 2.89E-30 3.21E-29

PADI1 0.062343248 3.396826756 5.76781051 2.91E-30 3.23E-29

BCL2L10 2.550475111 0.771822101 -1.724425762 2.94E-30 3.27E-29

CYTOR 1.49133694 5.177977986 1.795782587 3.00E-30 3.34E-29

BTG2 193.3563436 44.83398722 -2.108597384 3.05E-30 3.39E-29

AL021328.1 0.173607002 2.369806387 3.770872154 3.07E-30 3.41E-29

PLEK 2.499327369 12.07705911 2.272657399 3.13E-30 3.47E-29

SLC30A2 8.328763157 0.800618674 -3.378915096 3.18E-30 3.53E-29

MAP3K7CL 1.753906256 7.416105473 2.080090123 3.23E-30 3.58E-29

Z99289.1 0.03301156 0.351164036 3.411101863 3.31E-30 3.66E-29

PPP1R36 1.478023525 0.259821455 -2.508076759 3.42E-30 3.79E-29

LINC01485 0.914876842 0.224060733 -2.029687706 3.48E-30 3.86E-29

AC005064.1 0.026594027 0.006299836 -2.0777161 3.54E-30 3.92E-29

C1QTNF7 1.878741599 0.636098711 -1.562444084 3.57E-30 3.94E-29

ARSDP1 0.086470075 0.014935076 -2.533496352 3.58E-30 3.96E-29

AP001636.3 0.054284778 0.236897369 2.125642559 3.60E-30 3.97E-29

LINC01649 0.067836731 0.013683335 -2.309646709 3.65E-30 4.03E-29

COX4I2 2.715824143 19.34008474 2.832132147 3.65E-30 4.03E-29

COL18A1-AS1 0.808831245 0.213232132 -1.923413873 3.71E-30 4.09E-29

AC093849.1 0.005487229 0.100213622 4.190857079 3.74E-30 4.12E-29

CD96 0.392553479 2.23494666 2.509279283 3.84E-30 4.22E-29

CENPI 0.125928687 0.385372228 1.61364563 3.97E-30 4.35E-29

AL359853.1 0.033341461 0.342614162 3.361195576 3.98E-30 4.37E-29

LGI4 0.924436335 21.86502865 4.563907463 4.00E-30 4.39E-29

YEATS2-AS1 0.080667792 0.454780835 2.495106785 4.10E-30 4.49E-29

LAMA5-AS1 0.102876616 0.812123599 2.98078422 4.16E-30 4.56E-29

ERC2 0.198771936 0.063590876 -1.64422238 4.20E-30 4.59E-29

SH3BP1 0.672830315 2.112300025 1.650500151 4.20E-30 4.59E-29

MYF6 0.068694421 0.014640319 -2.230245959 4.28E-30 4.67E-29

AC115284.2 0.628201907 0.192564751 -1.705884684 4.37E-30 4.77E-29

PHKG1 0.124915627 0.494667456 1.985505018 4.45E-30 4.84E-29

CATIP-AS2 1.148341592 0.378829991 -1.599929402 4.61E-30 5.02E-29

SLC24A4 0.034263751 0.119069286 1.797046324 4.75E-30 5.16E-29

CD53 9.384634139 32.94409817 1.811647627 4.78E-30 5.20E-29

AC015977.1 0.034084068 0.943823735 4.791348034 4.80E-30 5.22E-29

TINCR 0.542684103 0.112549678 -2.269550722 4.82E-30 5.24E-29

CD3D 1.618134958 12.11799203 2.904746818 4.82E-30 5.24E-29

ACSF2 25.56426811 2.721778388 -3.231507213 4.86E-30 5.28E-29

TMEM145 0.043538696 0.859505075 4.303136062 4.90E-30 5.32E-29

AKR7A2P1 0.035126332 0.336592543 3.260378383 4.92E-30 5.34E-29

MAMDC2 2.72457525 0.693518452 -1.974025165 4.98E-30 5.40E-29

HLA-DQB1 14.35841368 71.73125957 2.320705595 4.98E-30 5.40E-29

BNIP3 18.27417681 54.27520146 1.570486757 5.11E-30 5.53E-29

SEMA3G 18.17338861 6.023849073 -1.593069921 5.15E-30 5.57E-29

AC084880.3 0.135912057 1.608491711 3.564963148 5.16E-30 5.58E-29

S100Z 0.03762079 0.188931335 2.328259932 5.33E-30 5.76E-29

DCDC2 23.81228676 7.71032063 -1.626843413 5.36E-30 5.78E-29

PILRA 2.016723426 6.333978652 1.651098759 5.36E-30 5.78E-29

AC011239.2 0.771542661 0.070334181 -3.455448045 5.37E-30 5.79E-29

LINC01447 0.054389182 0.017820991 -1.609742151 5.44E-30 5.86E-29

AC009951.4 0.010020769 0.232969802 4.53907783 5.67E-30 6.10E-29

KLC3 1.091151011 0.262438015 -2.055802159 5.76E-30 6.20E-29

CLK4 1.822402832 5.295318708 1.538875624 5.82E-30 6.25E-29

MIR210 0.255814454 3.803167669 3.894031859 5.89E-30 6.33E-29

NIPA2P1 0.003460519 0.345481324 6.641475592 5.94E-30 6.37E-29

SOX30 0.301805273 0.077238923 -1.96621805 6.11E-30 6.54E-29

PPP1R13L 3.480957736 10.55501862 1.600372921 6.26E-30 6.70E-29

XRCC2 0.140246306 0.435195803 1.633701871 6.26E-30 6.70E-29

AC084759.3 1.596975883 0.237425552 -2.749795413 6.34E-30 6.77E-29

AL031429.2 7.9942565 2.014980741 -1.988197813 6.33E-30 6.77E-29

RIN1 0.472756317 1.888164688 1.997815964 6.36E-30 6.80E-29

IL11 1.442833319 0.134805611 -3.419952193 6.57E-30 7.01E-29

LINC01738 0.100749715 1.516500645 3.911898453 6.73E-30 7.18E-29

PTGFR 2.835108582 0.926759906 -1.613136453 6.90E-30 7.36E-29

IFNG 0.033548735 0.770130505 4.520772651 6.90E-30 7.36E-29

KLHL3 2.635180489 0.834973952 -1.658098681 6.96E-30 7.41E-29

EVPL 6.259346236 2.102499113 -1.573906789 6.96E-30 7.41E-29

SLC2A1 16.14210251 63.5368224 1.976764438 6.96E-30 7.41E-29

TCIM 121.0886589 26.42338456 -2.196176572 7.02E-30 7.46E-29

HES4 1.831551906 9.072631117 2.308454414 7.02E-30 7.46E-29

SLCO1C1 0.070529577 0.406953682 2.528564315 7.02E-30 7.46E-29

EXO1 0.161270118 0.525297646 1.703655979 7.07E-30 7.51E-29

SLAMF7 0.648377985 4.704244136 2.859055922 7.07E-30 7.51E-29

SAMD14 0.407015226 1.27276279 1.644808892 7.13E-30 7.57E-29

MIR122HG 0.016149912 0.129344199 3.001617128 7.14E-30 7.58E-29

GRAMD4 5.542686083 18.89700809 1.769500629 7.19E-30 7.62E-29

FAM193B 1.966080549 8.585823902 2.126634154 7.19E-30 7.62E-29

AC020633.1 0.064353709 0.009130463 -2.817263367 7.28E-30 7.72E-29

AC008750.1 0.018696014 0.310398634 4.05331959 7.45E-30 7.88E-29

AOAH 1.455888357 6.794139932 2.222391204 7.55E-30 7.98E-29

AL162274.1 0.059669646 0.455871669 2.933558634 7.55E-30 7.98E-29

AL121796.1 0.551355213 0.066351846 -3.054773565 7.59E-30 8.02E-29

CCBE1 1.732462516 0.489675237 -1.822926992 7.61E-30 8.03E-29

PTGDR 0.125898207 0.600976753 2.255051445 7.67E-30 8.09E-29

AC004221.1 0.09190154 0.026262295 -1.807096024 7.92E-30 8.35E-29

RGL4 0.096268601 0.375564126 1.963922034 7.93E-30 8.35E-29

SLC4A8 0.825652725 0.128299124 -2.686023779 7.99E-30 8.41E-29

CDCA5 0.471204965 1.399565671 1.570552537 7.99E-30 8.41E-29

AC139495.2 0.026152861 0.218351798 3.061613749 8.46E-30 8.88E-29

AL512288.1 0.10175104 0.023393693 -2.120851997 8.65E-30 9.08E-29

SRL 1.448605024 0.393006492 -1.882039235 8.67E-30 9.09E-29

DEGS2 6.733143993 0.794410569 -3.083323596 9.17E-30 9.61E-29

CCR5AS 0.255871983 1.128615107 2.141059473 9.83E-30 1.03E-28

SIRPG 0.206470924 2.189064102 3.406303666 1.01E-29 1.06E-28

ABCA13 0.231848626 0.020365569 -3.508979124 1.05E-29 1.10E-28

KIF18A 0.205855315 0.601444387 1.546800644 1.07E-29 1.12E-28

MMP11 0.922479864 3.658903457 1.987822025 1.11E-29 1.16E-28

NRG3 0.320035543 1.272436077 1.99128914 1.12E-29 1.17E-28

SRSF3P1 0.583085258 0.161696565 -1.850417816 1.15E-29 1.20E-28

AC147055.1 0.879903563 0.065471315 -3.748410552 1.18E-29 1.23E-28

LINC00314 0.056255088 0.009936029 -2.501242313 1.19E-29 1.24E-28

SIRPB1 0.17449623 1.080339326 2.630216747 1.20E-29 1.25E-28

SGCZ 0.222154237 0.015760152 -3.817208333 1.20E-29 1.25E-28

AC012645.3 0.047133073 0.428087156 3.183092906 1.21E-29 1.25E-28

DOCK2 0.635168029 3.143769683 2.307285326 1.22E-29 1.26E-28

STMN3 7.105869833 31.17910834 2.133496504 1.23E-29 1.27E-28

C20orf204 0.049870941 0.221980375 2.154160794 1.24E-29 1.28E-28

LINC01150 0.228776632 1.151939418 2.332053242 1.24E-29 1.28E-28

AC010378.1 0.020127396 1.957086024 6.603402839 1.24E-29 1.29E-28

AC099548.2 0.119738382 0.386389367 1.690169716 1.27E-29 1.31E-28

LINC01871 0.447713907 2.41537707 2.431599392 1.27E-29 1.32E-28

TMEM233 0.613281449 2.56744766 2.06571365 1.28E-29 1.32E-28

SIRPB3P 0.003348918 0.113653838 5.084807403 1.32E-29 1.36E-28

AL607028.1 0.488224595 0.119999406 -2.024517715 1.32E-29 1.36E-28

NEK2 0.248114188 0.848628824 1.774129445 1.32E-29 1.37E-28

AC016026.1 0.033276527 0.238546728 2.841695119 1.33E-29 1.38E-28

AL022069.2 0.448555332 0.097276708 -2.205119653 1.35E-29 1.39E-28

SLFN11 3.083823514 10.18932426 1.72426627 1.35E-29 1.40E-28

WDR72 25.78748292 7.343593324 -1.812112888 1.36E-29 1.41E-28

CDCA7L 1.556091649 5.368508037 1.786594171 1.38E-29 1.42E-28

AC012613.2 0.001842949 0.078987071 5.421528435 1.39E-29 1.43E-28

TENT5B 5.932483567 0.979166188 -2.599010554 1.41E-29 1.45E-28

FAM13A-AS1 0.216505404 1.241791942 2.519948536 1.42E-29 1.46E-28

TRGV10 0.161556216 1.184493175 2.874161723 1.45E-29 1.49E-28

CDCA3 0.23019797 0.736696491 1.678195258 1.47E-29 1.51E-28

AC022400.9 0.233741993 0.798530661 1.772430856 1.48E-29 1.52E-28

AL031595.1 0.003045572 0.042630923 3.807115077 1.49E-29 1.53E-28

PRMT8 0.08234332 0.022453481 -1.874712517 1.51E-29 1.55E-28

SIRPD 0.009206329 0.080327608 3.125197978 1.51E-29 1.55E-28

IRF7 3.246384853 10.86632612 1.742958307 1.53E-29 1.57E-28

NOS1 1.580688498 0.08064948 -4.292744047 1.54E-29 1.58E-28

CDH26 0.057993503 0.184008171 1.665806653 1.54E-29 1.58E-28

DOK2 1.319769275 4.914704685 1.896818994 1.54E-29 1.58E-28

LRRK1 0.686337219 2.153168658 1.649471832 1.55E-29 1.59E-28

CAPS 8.11307964 2.232825295 -1.861379277 1.58E-29 1.61E-28

ADAMTSL4 0.748160844 3.312091799 2.146322291 1.58E-29 1.61E-28

BIRC3 5.138881764 25.90594016 2.333756578 1.64E-29 1.68E-28

AC004817.3 0.008063515 0.223026129 4.78966014 1.65E-29 1.68E-28

RAPGEFL1 0.331838028 1.127817051 1.76498193 1.68E-29 1.71E-28

RBM22P12 0.052743307 0.001653337 -4.995535181 1.71E-29 1.74E-28

CDKN2B 1.077422874 3.489958453 1.695625263 1.72E-29 1.75E-28

CD27 0.729040837 7.389494292 3.341404101 1.74E-29 1.77E-28

PKHD1L1 0.136939298 0.026213869 -2.385134301 1.77E-29 1.80E-28

AC012593.2 1.673591173 16.69444367 3.318348965 1.78E-29 1.81E-28

MEFV 0.116192453 0.458298683 1.979771777 1.86E-29 1.88E-28

TOMM40P2 0.03204746 0.283348377 3.144294995 1.86E-29 1.88E-28

CD80 0.0603403 0.340053775 2.494569123 1.89E-29 1.92E-28

RTEL1-TNFRSF6B 0.077287971 0.443098514 2.519311685 1.95E-29 1.97E-28

CADM4 17.45104154 5.452549978 -1.67831015 1.98E-29 2.00E-28

HPGD 10.93876474 2.548996387 -2.101448597 1.99E-29 2.01E-28

AANAT 0.026894143 0.173324435 2.688111157 2.01E-29 2.03E-28

ITGB2-AS1 0.223036171 1.665270729 2.900407134 2.01E-29 2.03E-28

PROCR 9.636000347 28.80449262 1.579787495 2.03E-29 2.04E-28

TAGAP 0.799857706 3.178739016 1.990639299 2.03E-29 2.04E-28

FOXD2-AS1 0.512954235 1.517886158 1.565161571 2.09E-29 2.11E-28

ABLIM3 4.395845149 18.01005537 2.034590146 2.13E-29 2.14E-28

FAM230C 0.09866615 0.009017824 -3.45170394 2.18E-29 2.19E-28

DUSP4 0.711342711 2.673226358 1.909965309 2.20E-29 2.21E-28

S1PR4 0.727896708 2.586876839 1.829405723 2.23E-29 2.24E-28

RIMKLA 0.73349814 3.772760422 2.362755276 2.25E-29 2.26E-28

LINC02041 0.352964463 4.161978755 3.559674756 2.33E-29 2.34E-28

SLC47A2 7.855981212 0.541198923 -3.859560607 2.40E-29 2.41E-28

PPFIA4 0.369549845 3.517686723 3.250786133 2.44E-29 2.44E-28

ZNF83 3.586138097 10.22595921 1.511733226 2.46E-29 2.46E-28

AL713899.1 0.071985798 1.44729106 4.329498974 2.50E-29 2.50E-28

PLIN5 1.409475043 0.492997434 -1.515505891 2.50E-29 2.50E-28

AC015819.1 0.225566245 0.766739407 1.765185141 2.52E-29 2.52E-28

ATG9B 0.112389562 0.869842456 2.952246071 2.52E-29 2.52E-28

SEMA6A 2.831858922 10.95869285 1.952254424 2.56E-29 2.56E-28

TRDN 0.073391319 0.025552494 -1.522145311 2.59E-29 2.58E-28

AL139039.1 0.062585478 0.005722289 -3.451163675 2.59E-29 2.59E-28

AC010198.1 0.148573277 0.01948443 -2.930781011 2.65E-29 2.64E-28

STAB1 4.505997458 14.95877809 1.731073928 2.67E-29 2.66E-28

CYGB 3.984707111 16.16402176 2.020240608 2.69E-29 2.68E-28

RNA5SP18 0.147594589 1.75606293 3.572632808 2.70E-29 2.69E-28

SPN 0.684516422 3.083263994 2.171301362 2.71E-29 2.70E-28

GDF6 0.37890828 4.544392649 3.584166923 2.78E-29 2.76E-28

CDON 0.620468379 3.000772979 2.273904584 2.82E-29 2.80E-28

AC025271.1 0.404645464 0.103492844 -1.967127404 2.84E-29 2.82E-28

CD8B 0.386198107 4.244911035 3.45832132 2.84E-29 2.82E-28

HAMP 0.042262231 0.565979605 3.74330925 2.84E-29 2.82E-28

CTSS 10.84665618 41.53150929 1.936955946 2.94E-29 2.91E-28

LINC02521 0.030766358 0.227647061 2.887373252 2.97E-29 2.95E-28

PSORS1C3 1.595966915 15.21931104 3.253400401 2.98E-29 2.96E-28

CHGA 0.45881226 0.103209162 -2.152332893 2.99E-29 2.96E-28

LAT 0.025849011 0.275403271 3.413364728 3.03E-29 3.00E-28

AGR3 1.37164739 0.33103386 -2.050858957 3.20E-29 3.17E-28

RPL7P50 0.027957464 0.247761711 3.147647836 3.29E-29 3.25E-28

NAT2 0.900692763 0.270082497 -1.737634922 3.30E-29 3.26E-28

GABRP 2.220298322 0.482186992 -2.203088894 3.31E-29 3.27E-28

IYD 4.423878008 0.238376528 -4.213997512 3.37E-29 3.32E-28

SLC14A2 12.24156341 0.132961566 -6.524634734 3.45E-29 3.40E-28

LINC02609 0.187699391 0.898105391 2.258460783 3.48E-29 3.43E-28

RAC2 4.125681333 17.26656379 2.065276704 3.53E-29 3.48E-28

TLCD3A 3.705001542 11.03845636 1.574992389 3.59E-29 3.53E-28

AL445222.2 0.2085552 0.730726383 1.808902016 3.59E-29 3.53E-28

AL353801.1 5.664125478 1.296056012 -2.127725157 3.69E-29 3.63E-28

CTAGE9 0.020096938 0.368114611 4.195107404 3.71E-29 3.64E-28

AC083967.1 0.011282092 0.610006114 5.756717163 3.79E-29 3.71E-28

AC004865.2 0.122290623 0.491641994 2.007294365 3.81E-29 3.74E-28

TMEM45A 0.973257596 8.021134611 3.04291272 3.83E-29 3.75E-28

ADAMTS7P1 0.004995028 0.034948296 2.806657384 3.88E-29 3.80E-28

PGGHG 2.031994943 24.24549482 3.576747981 3.89E-29 3.81E-28

ARHGAP15 0.680641636 2.127802768 1.644397118 4.05E-29 3.96E-28

TPRG1 0.064556699 0.381203682 2.561923333 4.18E-29 4.09E-28

AC107396.1 0.074880825 0.007632358 -3.294395552 4.35E-29 4.25E-28

AL078604.2 0.034753459 0.355462745 3.354469882 4.46E-29 4.35E-28

AC007991.4 0.011748735 0.384423637 5.032119694 4.48E-29 4.37E-28

UBA52P6 0.114347293 0.480916806 2.072365132 4.51E-29 4.39E-28

AXL 8.205070653 24.92379701 1.60293621 4.57E-29 4.45E-28

DNAH11 0.108037348 2.824726231 4.708509015 4.60E-29 4.48E-28

AC016822.1 0.067473121 0.015166577 -2.15341736 4.63E-29 4.50E-28

AC087164.1 0.02430521 0.13722528 2.4972088 4.77E-29 4.63E-28

AC000067.1 0.051510998 0.495312068 3.265385386 4.78E-29 4.64E-28

CACNA2D4 0.299653483 0.982678717 1.713424666 4.87E-29 4.72E-28

TMIGD2 0.099493346 0.454141846 2.190471026 5.03E-29 4.87E-28

TRBC2 2.576459104 18.20364254 2.820765564 5.03E-29 4.87E-28

CDCA7 0.261865057 1.069732386 2.030354458 5.11E-29 4.94E-28

SPTSSB 0.843533951 0.071772863 -3.554935751 5.24E-29 5.06E-28

LAG3 0.321975235 3.78157404 3.553965234 5.32E-29 5.14E-28

TFR2 0.048947392 0.754157325 3.945561616 5.32E-29 5.14E-28

CLEC1B 0.008619285 0.064789805 2.910126767 5.33E-29 5.15E-28

AC084880.1 1.472826144 7.585566004 2.364669691 5.54E-29 5.34E-28

PADI2 14.23608738 2.525784916 -2.494748996 5.58E-29 5.37E-28

CYRIA 1.325616379 3.935572954 1.56991035 5.67E-29 5.46E-28

TIGIT 0.162190975 1.226441911 2.918713456 5.67E-29 5.46E-28

AC007728.2 0.057291831 0.301434979 2.395445492 5.75E-29 5.54E-28

AC004687.1 0.090107372 0.986754326 3.452973887 5.85E-29 5.63E-28

IFI44 5.393094931 16.26535588 1.592617055 5.90E-29 5.67E-28

AIDAP2 0.01694397 0.103166854 2.606135683 6.07E-29 5.83E-28

AC108704.1 0.200310682 0.912947227 2.188292107 6.19E-29 5.94E-28

AC015977.2 0.014170638 0.837433691 5.884998393 6.48E-29 6.21E-28

ANAPC1P4 0.170339962 0.701585016 2.042201004 6.50E-29 6.22E-28

P4HA3 0.305583323 2.089957349 2.773835782 6.55E-29 6.27E-28

AC128687.2 0.231486106 0.07044418 -1.716373171 6.58E-29 6.30E-28

BTBD19 0.959842457 3.711220214 1.951024075 6.71E-29 6.41E-28

LACTB2-AS1 0.04451216 0.18698045 2.07061602 6.71E-29 6.41E-28

NOC2LP1 0.310369837 0.088008789 -1.818268853 6.76E-29 6.45E-28

LAIR2 0.048505092 0.790285676 4.026166139 6.80E-29 6.49E-28

MARCHF4 0.054037708 0.36762737 2.7662058 6.87E-29 6.55E-28

ADAMTS7P3 0.009782047 0.054416217 2.475828426 7.09E-29 6.76E-28

LINC01270 0.061485005 0.224580755 1.868927804 7.15E-29 6.80E-28

NINJ2 0.398763239 1.284402884 1.687493488 7.26E-29 6.91E-28

MACORIS 0.235629934 0.933232814 1.985714208 7.29E-29 6.93E-28

Z94721.1 0.119882273 0.866926493 2.854291324 7.31E-29 6.94E-28

KLRK1 0.029883865 0.326691405 3.450489826 7.38E-29 7.01E-28

DIRAS2 0.909225106 7.364273278 3.017833739 7.44E-29 7.06E-28

AL133387.1 0.126228233 0.038417931 -1.716182908 7.48E-29 7.10E-28

ELDR 0.003973666 0.128014967 5.009698084 7.49E-29 7.10E-28

AL162727.1 0.152295201 0.039735914 -1.93835506 7.55E-29 7.15E-28

MYL4 0.171086951 0.51073126 1.577834637 7.56E-29 7.16E-28

PLD4 0.382278107 1.878126168 2.296599498 7.56E-29 7.16E-28

AC016397.2 0.018676896 0.089210347 2.255956345 7.56E-29 7.17E-28

SCARF1 2.366696394 7.057962935 1.576377208 7.62E-29 7.21E-28

FRG2C 2.011799035 0.098879493 -4.346671036 7.72E-29 7.31E-28

UPP2 9.156492904 0.480300644 -4.252785479 7.74E-29 7.32E-28

AC026336.1 0.073055192 0.006988731 -3.385884484 7.81E-29 7.38E-28

LINC02292 0.084176473 0.017905414 -2.233021214 7.85E-29 7.42E-28

AC079209.1 0.030613193 0.228008878 2.896864578 7.99E-29 7.55E-28

TRAM1L1 7.006953542 2.464245189 -1.507641521 8.06E-29 7.61E-28

CD3E 1.760655164 11.22003117 2.671892403 8.19E-29 7.73E-28

DLK2 0.198237225 1.013021194 2.353364553 8.73E-29 8.23E-28

SLAMF6 0.625194848 3.704744733 2.566996346 8.80E-29 8.29E-28

AC005785.1 0.121053028 0.610457021 2.334250555 9.01E-29 8.48E-28

MROCKI 0.417985647 2.241825967 2.423148979 9.01E-29 8.48E-28

AC148477.2 0.62095348 0.1488674 -2.060457337 9.06E-29 8.52E-28

CATSPER1 0.062505563 0.25256355 2.014589944 9.23E-29 8.68E-28

CHRDL1 7.544807333 2.171027519 -1.797106047 9.32E-29 8.76E-28

SLC10A6 0.140673225 0.908405767 2.690989102 9.38E-29 8.81E-28

AC016957.2 0.274332098 0.887658692 1.694081629 9.53E-29 8.94E-28

PCDHB10 0.623858614 2.063701843 1.725943539 9.68E-29 9.08E-28

RASSF5 1.457845893 4.860576768 1.737289295 9.68E-29 9.08E-28

LINC01312 0.018893219 0.004202115 -2.168681102 9.79E-29 9.17E-28

AL162171.1 2.443813018 0.76742304 -1.671039922 9.84E-29 9.21E-28

AC005034.5 0.749171785 0.227147565 -1.721666728 1.01E-28 9.42E-28

TMEM75 0.003842323 0.136767522 5.153602951 1.03E-28 9.61E-28

EVI2A 1.999889547 7.42991654 1.893425682 1.07E-28 9.93E-28

IKZF1 0.657617358 2.852471677 2.116892276 1.07E-28 9.93E-28

COL4A1 47.82685222 175.3824953 1.874612015 1.08E-28 1.01E-27

AC011462.1 0.010668796 0.073115951 2.776788758 1.08E-28 1.01E-27

NDNF 23.21243953 4.446979875 -2.384000371 1.09E-28 1.02E-27

GNA15 0.868479335 2.909155838 1.744037155 1.10E-28 1.02E-27

CR2 2.50528243 0.183293138 -3.77274857 1.14E-28 1.06E-27

LINC02814 0.184115915 0.027066322 -2.766043597 1.14E-28 1.06E-27

AL162725.2 0.839210423 0.02578768 -5.024278725 1.15E-28 1.06E-27

LINC02302 0.042472663 0.014875389 -1.513607179 1.15E-28 1.06E-27

USP30-AS1 0.394299421 2.072622248 2.394093699 1.15E-28 1.07E-27

CPA6 0.079085163 0.451020925 2.511715403 1.16E-28 1.07E-27

PTPN22 0.326500886 1.408671588 2.109176494 1.28E-28 1.18E-27

AC104393.1 0.057826104 0.018524069 -1.642319856 1.29E-28 1.19E-27

AC011337.1 0.034081547 0.179294767 2.395270657 1.29E-28 1.20E-27

AC079467.1 0.03272813 0.006990211 -2.227123283 1.34E-28 1.24E-27

CENPE 0.141142898 0.437663557 1.632665726 1.36E-28 1.26E-27

CBY2 0.127834824 0.032829459 -1.96121802 1.39E-28 1.28E-27

SLC30A8 0.720768195 0.078733198 -3.194491363 1.41E-28 1.30E-27

AC079612.1 0.010305511 0.003571513 -1.528808804 1.41E-28 1.30E-27

MDS2 0.031174077 0.150530425 2.271636362 1.42E-28 1.31E-27

MT1G 532.7081502 24.31696708 -4.453310148 1.42E-28 1.31E-27

INSC 0.198115473 0.058505053 -1.759708426 1.44E-28 1.32E-27

MT1H 93.20017893 4.531718349 -4.362202621 1.49E-28 1.37E-27

WDR62 0.108813111 0.315398072 1.535321439 1.52E-28 1.40E-27

KCNK9 0.046907578 1.113727643 4.569431666 1.55E-28 1.42E-27

CDKL2 4.504130528 1.504957873 -1.581525533 1.57E-28 1.44E-27

CD52 6.109696011 26.21698756 2.101329418 1.57E-28 1.44E-27

AC099568.2 1.935018079 0.578785479 -1.741246413 1.59E-28 1.45E-27

AC022144.1 1.099055503 5.979006996 2.443641654 1.59E-28 1.45E-27

AC026369.3 0.028990475 0.419224266 3.854071335 1.62E-28 1.48E-27

U62317.2 1.890257183 6.762733048 1.839023869 1.62E-28 1.48E-27

LINC01271 0.012861376 0.102703835 2.997373128 1.66E-28 1.52E-27

COL5A2 4.043147458 16.81573731 2.056261311 1.72E-28 1.57E-27

AL365181.3 0.45737711 2.678763996 2.550111412 1.72E-28 1.57E-27

PLOD2 13.39686915 44.16285257 1.72093748 1.73E-28 1.58E-27

NTRK2 7.674335042 1.867513918 -2.038922755 1.74E-28 1.59E-27

AC006960.3 1.009431838 0.043858089 -4.524556741 1.77E-28 1.61E-27

SEL1L3 6.965037833 22.26376404 1.676494429 1.79E-28 1.62E-27

CLSPN 0.143628893 0.449519532 1.646037806 1.80E-28 1.64E-27

IKZF3 0.331399566 2.030108258 2.614913046 1.80E-28 1.64E-27

KCNN1 0.048862429 0.965840504 4.304987469 1.80E-28 1.64E-27

LINC01428 0.190741621 2.727427881 3.837849459 1.82E-28 1.66E-27

DLX6 0.024513382 0.150392306 2.617089323 1.83E-28 1.66E-27

AL132657.1 0.280932633 0.978174418 1.799867516 1.83E-28 1.66E-27

LINC02073 0.018477512 0.175803975 3.25012529 1.86E-28 1.69E-27

EVI2B 3.330214731 13.03070022 1.968227502 1.87E-28 1.70E-27

AF131216.3 0.036487427 0.011599394 -1.653349933 1.89E-28 1.71E-27

LILRA2 0.253274353 0.957236218 1.918173988 1.90E-28 1.72E-27

S100A5 0.95986352 0.320581968 -1.582136007 1.95E-28 1.76E-27

HASPIN 0.085646454 0.28481299 1.733549527 1.96E-28 1.78E-27

FGR 1.999401647 5.741111935 1.521761869 1.98E-28 1.79E-27

C15orf54 0.010221749 0.076539845 2.904568962 1.99E-28 1.80E-27

TRPM5 0.096944333 0.022795453 -2.088410519 2.00E-28 1.81E-27

CTSV 2.577228031 0.372850582 -2.789150692 2.04E-28 1.85E-27

C3orf67 0.17337756 0.551913801 1.670525773 2.06E-28 1.86E-27

SEPTIN5 1.023569332 3.39916611 1.731572038 2.08E-28 1.87E-27

LINC00607 0.020602127 0.098244282 2.253580141 2.08E-28 1.87E-27

AP003108.5 0.138271355 0.027999767 -2.304015601 2.08E-28 1.88E-27

PCSK6 0.974560968 7.786660459 2.99818037 2.09E-28 1.89E-27

KPNA7 0.009504586 0.107683475 3.502029292 2.13E-28 1.92E-27

AC243960.1 0.190792211 0.905843615 2.247259733 2.19E-28 1.97E-27

AL662844.3 0.115057083 0.989206459 3.103921859 2.20E-28 1.98E-27

RHOH 0.232214088 1.114850157 2.263322408 2.23E-28 2.00E-27

GPR65 0.624472513 2.379821988 1.930143688 2.25E-28 2.02E-27

HLA-DQA1 8.914367764 42.15292427 2.241428333 2.25E-28 2.02E-27

GSG1L 0.040498175 0.013283382 -1.608234429 2.29E-28 2.06E-27

PLAC8L1 0.085234942 0.547450151 2.683210717 2.32E-28 2.08E-27

IL32 21.53573967 83.82719765 1.960685526 2.32E-28 2.08E-27

LINC01224 0.327547192 0.061737556 -2.407482495 2.36E-28 2.12E-27

AL162724.1 0.024456312 0.472565953 4.272236917 2.37E-28 2.12E-27

AC083902.1 0.126875166 0.02982883 -2.08863042 2.39E-28 2.14E-27

AL592295.1 0.076919283 0.256702541 1.738680362 2.41E-28 2.16E-27

AC007098.1 0.106151122 0.572507396 2.431174709 2.41E-28 2.16E-27

MND1 0.252720219 0.72236309 1.515183086 2.45E-28 2.19E-27

AJ003147.3 0.02833583 0.218285667 2.945518019 2.45E-28 2.19E-27

KCNE4 2.292917635 9.532058827 2.055603323 2.47E-28 2.21E-27

AC093908.1 0.106874047 0.431701506 2.014122566 2.51E-28 2.24E-27

WT1-AS 0.39421997 0.072296125 -2.447010634 2.56E-28 2.29E-27

KSR1 2.708142889 10.44603089 1.947579108 2.59E-28 2.31E-27

AC015911.8 0.118244872 0.948182238 3.003386754 2.69E-28 2.40E-27

GZMM 0.599017491 2.540450229 2.084414166 2.72E-28 2.42E-27

AC027559.1 0.523698049 1.824900066 1.801010328 2.74E-28 2.44E-27

KLHL2P1 0.183310527 0.735908388 2.00523654 2.74E-28 2.44E-27

KRT81 0.036959353 0.422861431 3.516173567 2.80E-28 2.49E-27

C5orf67 1.976399426 0.30011945 -2.719265817 2.85E-28 2.54E-27

LINGO3 0.035900347 0.210854578 2.554178654 2.96E-28 2.63E-27

B4GALNT1 0.19358754 2.863771179 3.886858222 2.96E-28 2.63E-27

AC010319.1 0.095278534 0.388062927 2.0260675 2.99E-28 2.65E-27

SLC16A10 1.837978289 0.648523139 -1.50288977 3.01E-28 2.67E-27

BX640514.2 0.309585969 1.326625909 2.099349613 3.01E-28 2.67E-27

PDE1B 0.777354383 2.45399052 1.658485321 3.03E-28 2.69E-27

ALKBH6 0.208363367 0.68840122 1.724148003 3.06E-28 2.71E-27

PYGO1 1.047223397 0.333787794 -1.64956613 3.08E-28 2.73E-27

AIM2 0.159185024 1.32448773 3.056657959 3.08E-28 2.73E-27

FPR3 3.044746508 14.24515296 2.226077087 3.11E-28 2.75E-27

SCIMP 0.48444316 1.939949194 2.001619561 3.13E-28 2.77E-27

PPEF1 0.044016187 0.286455638 2.702205654 3.13E-28 2.77E-27

AC090152.1 1.603297864 0.564576993 -1.505800233 3.26E-28 2.88E-27

SLC6A1 0.37253083 1.828547538 2.295266404 3.26E-28 2.88E-27

AC011313.1 0.281170306 2.621190686 3.220706163 3.28E-28 2.90E-27

AL031719.2 0.333552738 1.262468026 1.920260062 3.28E-28 2.90E-27

KIR2DL4 0.071827739 0.384075366 2.418776434 3.36E-28 2.97E-27

CORIN 0.38153444 0.129747899 -1.556102116 3.53E-28 3.11E-27

FABP5 1.390030232 5.110482824 1.878343339 3.53E-28 3.11E-27

LINC02236 0.024552503 0.224256705 3.191209129 3.67E-28 3.24E-27

SEZ6L2 4.18342761 20.48151179 2.291564832 3.70E-28 3.25E-27

AC026356.2 0.211542978 0.870792292 2.041377843 3.78E-28 3.33E-27

AP003071.3 0.156532093 0.026405986 -2.567521544 3.80E-28 3.34E-27

AL159163.1 0.091585242 0.427084014 2.221332853 3.96E-28 3.48E-27

SLC43A3 2.669088651 7.836844108 1.553925575 3.97E-28 3.48E-27

SPC25 0.338747939 1.2127816 1.840035695 4.00E-28 3.51E-27

CCL11 2.321754935 0.411467152 -2.496366534 4.10E-28 3.59E-27

SOCAR 0.067984681 0.342852448 2.334306212 4.16E-28 3.64E-27

UNC5B 5.123127689 17.84339698 1.800293543 4.23E-28 3.70E-27

DTHD1 0.025331771 0.25359307 3.323495464 4.26E-28 3.73E-27

MOXD1 14.11447025 2.802109149 -2.332589924 4.43E-28 3.87E-27

BRWD1-AS2 1.017552271 0.331309407 -1.618851835 4.43E-28 3.87E-27

HLA-DRB1 205.239071 640.0057864 1.640779549 4.50E-28 3.93E-27

CEACAM21 0.333127012 1.227125435 1.88113848 4.50E-28 3.93E-27

TMED10P2 1.248036803 0.290014011 -2.10546597 4.58E-28 3.99E-27

CYP1A1 0.93614846 0.110894701 -3.077546904 4.58E-28 4.00E-27

AC007490.1 1.750346536 0.577197223 -1.600504313 4.64E-28 4.04E-27

RGS5 58.33641944 358.7330972 2.620442112 4.64E-28 4.05E-27

GPR4 4.896542583 18.8594624 1.945453216 4.68E-28 4.08E-27

CARMIL2 0.089130701 0.515061262 2.530749673 4.72E-28 4.11E-27

OTOF 0.00837902 0.050407254 2.588777866 4.79E-28 4.16E-27

RAB33A 0.35543965 1.55095771 2.125482818 4.83E-28 4.20E-27

LINC02099 0.001818459 0.031559417 4.117282498 4.95E-28 4.30E-27

DYSF 6.811971028 19.42914683 1.512078347 4.99E-28 4.33E-27

C3AR1 3.346978871 12.57674676 1.909827438 5.02E-28 4.36E-27

LINC02310 0.008784105 0.256274742 4.866652233 5.06E-28 4.39E-27

NRP2 1.891662182 6.209033214 1.714714178 5.06E-28 4.39E-27

SLC9A9 1.806797042 6.365325386 1.816799808 5.19E-28 4.49E-27

ICAM3 0.250033743 1.014098726 2.020003403 5.31E-28 4.59E-27

LAMA4-AS1 0.015016673 0.076682076 2.352324176 5.39E-28 4.66E-27

AF131215.7 0.582491901 0.177725418 -1.71258796 5.42E-28 4.69E-27

UTS2R 0.545183141 0.031739207 -4.102402966 5.45E-28 4.71E-27

PLEKHG4 0.560673496 2.442771068 2.123285886 5.79E-28 4.99E-27

LINC01235 0.507236878 4.217569472 3.055680289 5.88E-28 5.07E-27

AFAP1L1 3.461961028 9.932583723 1.520579565 5.97E-28 5.15E-27

RTP2 0.006550877 0.258093904 5.300064099 5.99E-28 5.16E-27

AP000769.1 0.885670976 2.821652489 1.671697571 6.02E-28 5.18E-27

UNC5A 0.056414074 0.587547082 3.380577428 6.07E-28 5.22E-27

LINC00426 0.065963634 0.407551855 2.627240844 6.09E-28 5.24E-27

POU2F2 0.37479018 1.434952726 1.936848152 6.12E-28 5.26E-27

C3orf86 0.758859821 0.225647594 -1.749762014 6.16E-28 5.30E-27

KIR2DL3 0.015044934 0.087791208 2.544798646 6.21E-28 5.34E-27

HLA-DRA 343.1340806 1147.741158 1.741952989 6.26E-28 5.38E-27

AC104316.2 0.035198692 0.513354308 3.866361176 6.29E-28 5.41E-27

AL591926.7 0.498655644 0.131443856 -1.923597172 6.33E-28 5.44E-27

MEF2B 0.067171471 0.280599977 2.062594367 6.34E-28 5.44E-27

BNIP3P33 0.110170857 0.026821146 -2.038299852 6.38E-28 5.48E-27

CBLN3 1.152428208 3.602153913 1.644182947 6.46E-28 5.54E-27

NLGN1 0.863635864 3.606112446 2.061949324 6.46E-28 5.54E-27

LINC02446 0.0577345 0.658960558 3.512686529 6.62E-28 5.68E-27

MISP 5.671915125 1.779461104 -1.672395546 6.67E-28 5.71E-27

COL6A2 23.43663035 90.9595535 1.956460014 6.67E-28 5.71E-27

DCSTAMP 0.029995388 0.265781414 3.147427614 6.69E-28 5.73E-27

ANO4 0.154154371 2.038379574 3.724975027 6.75E-28 5.78E-27

CD3G 0.354834962 2.149054049 2.598481698 6.83E-28 5.84E-27

AC023794.4 1.396663994 0.46428762 -1.588894264 6.92E-28 5.92E-27

GAPLINC 0.085080507 0.369521722 2.118758632 7.04E-28 6.01E-27

AC108676.1 0.076567014 0.409667281 2.419657777 7.04E-28 6.01E-27

NKPD1 0.0085615 0.065996822 2.946461071 7.22E-28 6.16E-27

COLCA1 3.252070764 1.055254527 -1.623767633 7.27E-28 6.20E-27

KRT24 0.060968441 0.018393259 -1.728885534 7.44E-28 6.34E-27

MCOLN3 1.259686615 0.441798555 -1.511604258 7.80E-28 6.63E-27

AC011257.1 0.17487426 0.03551499 -2.299817951 7.85E-28 6.67E-27

AC116407.4 0.070262868 0.287690981 2.03368562 8.05E-28 6.84E-27

FANCA 0.205336984 0.652863696 1.66878832 8.37E-28 7.10E-27

NCF2 2.062473179 6.994414958 1.761828031 8.44E-28 7.15E-27

ADAM8 0.588298344 2.56805713 2.126057416 8.44E-28 7.15E-27

HMX3 0.046112717 0.008195967 -2.492178659 8.54E-28 7.24E-27

LOXL3 0.6874365 2.082144719 1.598771986 8.57E-28 7.25E-27

AC009159.2 0.089243674 0.732248935 3.036512374 8.60E-28 7.28E-27

PHLDA3 6.157462569 18.64748827 1.598573461 8.64E-28 7.30E-27

AC009119.1 0.103826994 0.343635301 1.726696674 8.64E-28 7.30E-27

KREMEN2 0.023089435 0.166809384 2.852895648 8.90E-28 7.52E-27

CHAC1 4.404742008 1.083240905 -2.023703397 8.91E-28 7.53E-27

SIGLEC7 0.3954582 1.735314715 2.133600216 8.91E-28 7.53E-27

CD180 0.324302843 1.435832085 2.146473465 9.05E-28 7.64E-27

SIT1 0.534095041 3.558756068 2.736204653 9.12E-28 7.70E-27

AC021078.1 0.830892818 2.949858335 1.82791138 9.41E-28 7.93E-27

MSR1 1.976159253 8.485425345 2.102287765 9.49E-28 7.99E-27

AL596442.2 0.150026935 1.039194333 2.792172025 9.52E-28 8.02E-27

Z98751.2 0.054203376 1.249112384 4.526376771 9.78E-28 8.23E-27

C19orf67 0.047955529 0.701440282 3.870551207 9.89E-28 8.32E-27

AL121832.1 0.020261633 0.007022355 -1.52872365 1.02E-27 8.53E-27

PLCXD3 2.719047508 0.783007491 -1.796003344 1.04E-27 8.75E-27

RPS24P17 4.402992474 0.255194408 -4.108815758 1.05E-27 8.82E-27

LTA 0.091767198 0.577242079 2.653126016 1.06E-27 8.91E-27

LINC02076 0.025477467 0.19778823 2.956662838 1.09E-27 9.11E-27

CNIH2 0.074387138 0.288520877 1.955550609 1.10E-27 9.23E-27

AC011411.1 0.037638989 0.337060441 3.16270752 1.12E-27 9.36E-27

AC103740.1 0.107899959 0.505373526 2.227655769 1.13E-27 9.43E-27

ZP2 0.059582248 0.00971281 -2.616921862 1.14E-27 9.55E-27

MANCR 0.011953172 0.206449322 4.110322251 1.14E-27 9.57E-27

AC022424.1 0.956468354 0.243550613 -1.97349562 1.16E-27 9.74E-27

SCG2 0.206756901 3.342029928 4.014717263 1.19E-27 9.95E-27

AC010273.3 0.79562018 0.142504719 -2.481070176 1.22E-27 1.02E-26

HLA-DPA1 33.19723619 121.3011518 1.869458208 1.23E-27 1.03E-26

AP007216.2 0.039121196 0.425048567 3.441605311 1.23E-27 1.03E-26

PCSK2 0.082859025 0.017615565 -2.233808071 1.24E-27 1.03E-26

PRSS35 4.475548983 0.971317928 -2.204049163 1.25E-27 1.04E-26

BCAS1 1.135993156 0.265547225 -2.096913782 1.25E-27 1.04E-26

AC004540.2 6.333620961 2.140098806 -1.565353125 1.30E-27 1.08E-26

LINC02285 0.100155606 0.365721441 1.868502036 1.37E-27 1.14E-26

LNCSRLR 0.26932953 1.394997038 2.372817735 1.37E-27 1.14E-26

MSH4 0.021121204 0.168699429 2.997691115 1.37E-27 1.14E-26

AADACL4 0.082334531 0.016537882 -2.315723165 1.41E-27 1.17E-26

DIPK2B 2.925778058 10.8789871 1.894651999 1.41E-27 1.17E-26

CTHRC1 1.958374904 16.23718885 3.051572999 1.44E-27 1.19E-26

ISM2 0.67842872 0.221270855 -1.616383808 1.46E-27 1.21E-26

CLEC3A 0.071104981 0.015132438 -2.232306153 1.46E-27 1.21E-26

AC093725.2 0.088434135 0.007730618 -3.51594776 1.48E-27 1.22E-26

CHTF18 0.475129856 1.394323007 1.553171044 1.48E-27 1.23E-26

SLC29A4 1.873210439 8.375827693 2.160718781 1.49E-27 1.24E-26

AL162419.1 0.155598446 0.045818612 -1.763821985 1.50E-27 1.24E-26

MMP16 0.077422438 0.560872813 2.856850009 1.52E-27 1.26E-26

SCGB3A2 0.016306443 0.698494462 5.420734615 1.53E-27 1.27E-26

AC004494.1 0.020400991 0.164121349 3.008051788 1.55E-27 1.28E-26

GPR141 0.126524831 0.660689293 2.384551415 1.56E-27 1.29E-26

HCST 2.506624654 9.894549697 1.98088807 1.59E-27 1.31E-26

MCUB 1.083694832 3.357785803 1.631551651 1.60E-27 1.32E-26

DUXAP8 0.036679161 0.250957731 2.774411845 1.60E-27 1.32E-26

RUNX1 1.330867568 4.475056271 1.749538805 1.61E-27 1.33E-26

P2RY8 2.062411426 7.683752404 1.89747887 1.68E-27 1.38E-26

HPCAL4 2.760841413 0.177628789 -3.958170691 1.71E-27 1.41E-26

SEMA6A-AS1 0.144724342 0.740373344 2.354945358 1.72E-27 1.42E-26

XCL2 0.332617371 1.964680643 2.562359401 1.73E-27 1.43E-26

GRB10 6.636349833 19.1403704 1.528156904 1.74E-27 1.44E-26

DTX2P1 0.214173535 0.671395063 1.648381706 1.76E-27 1.45E-26

PLSCR3 0.094858615 0.289108285 1.607759237 1.77E-27 1.46E-26

MLIP 0.069581502 0.312347902 2.166378109 1.86E-27 1.53E-26

CTLA4 0.114287353 0.907691279 2.98953593 1.86E-27 1.53E-26

AL031429.1 1.186410315 0.402810612 -1.558429451 1.86E-27 1.53E-26

USP2 21.11981061 5.947915357 -1.828140875 1.87E-27 1.54E-26

CD14 18.80520831 62.83544718 1.740446365 1.94E-27 1.60E-26

AC011586.2 0.089268126 0.024809082 -1.847276794 1.95E-27 1.60E-26

N4BP2L2-IT2 0.146033703 0.772945436 2.404065209 1.96E-27 1.61E-26

SCGB2A1 2.459267677 0.520661085 -2.239812285 1.97E-27 1.61E-26

ITPKA 0.09901447 1.438261707 3.860543031 2.01E-27 1.65E-26

PKNOX2 0.785185504 0.268871452 -1.546116957 2.07E-27 1.69E-26

AL136962.1 0.275713028 0.087878583 -1.649583922 2.18E-27 1.78E-26

CACNB4 0.473129447 0.156164726 -1.59916634 2.22E-27 1.81E-26

AC025171.4 0.167365533 0.980219953 2.550103063 2.23E-27 1.82E-26

FSCN1 9.285839792 27.24454454 1.552863078 2.27E-27 1.85E-26

AC011270.2 0.045599448 0.012108377 -1.91301084 2.28E-27 1.85E-26

HOXD13 0.026006922 0.258126209 3.311109087 2.32E-27 1.89E-26

LINC02159 0.279986728 0.063178183 -2.147860092 2.33E-27 1.90E-26

RPL7AP7 0.005833648 0.174641338 4.903852931 2.34E-27 1.90E-26

HES5 0.021555276 0.312271753 3.856689166 2.35E-27 1.91E-26

CLEC12B 0.005371979 0.075430587 3.81162412 2.35E-27 1.92E-26

PPP1R1C 0.276763931 0.084806221 -1.706413938 2.37E-27 1.93E-26

IL4I1 1.567318124 6.66819712 2.088998715 2.42E-27 1.97E-26

EIF4A1 0.425400621 1.931569211 2.182879328 2.49E-27 2.03E-26

B3GALT2 0.695039127 0.243777024 -1.51153204 2.57E-27 2.09E-26

ADAMTS10 0.720304188 3.318377704 2.203799909 2.63E-27 2.14E-26

CBLN2 0.563682111 0.065925184 -3.095980191 2.64E-27 2.14E-26

SLC9A5 0.11545852 0.402574641 1.801881665 2.65E-27 2.15E-26

AC084117.1 0.236447428 2.19738922 3.216199081 2.69E-27 2.18E-26

ARG2 37.12929571 9.539183227 -1.960620301 2.69E-27 2.18E-26

ALS2CL 4.751228111 1.461386593 -1.700962596 2.84E-27 2.30E-26

JAG2 2.464757322 7.855815598 1.672315458 2.89E-27 2.34E-26

UGT2A1 1.680503923 0.072063182 -4.543487739 2.89E-27 2.34E-26

SH3GL3 2.022772624 0.039532294 -5.677158668 2.90E-27 2.35E-26

ADGRE2 0.426565821 1.514130619 1.82764939 2.91E-27 2.35E-26

AL365181.2 0.130982067 1.030728596 2.9762233 2.91E-27 2.35E-26

NCMAP-DT 0.043702576 0.011280833 -1.953844752 2.95E-27 2.38E-26

ADGRB3 0.4072111 0.126998787 -1.680962169 3.03E-27 2.44E-26

POPDC2 1.385426447 4.176575524 1.591990407 3.03E-27 2.44E-26

FABP6-AS1 0.003299074 0.092563652 4.810312839 3.03E-27 2.45E-26

SLC14A1 13.07680767 1.642118798 -2.993379984 3.05E-27 2.46E-26

LINC01929 0.041636436 0.486620094 3.546877407 3.06E-27 2.47E-26

EGFR-AS1 0.162631198 5.553533335 5.093730003 3.10E-27 2.50E-26

SLA 1.537339118 5.434049576 1.821592287 3.15E-27 2.54E-26

AP005229.1 0.203256669 0.019925167 -3.350638947 3.16E-27 2.55E-26

TUBAL3 2.369958628 0.162240092 -3.868659597 3.17E-27 2.56E-26

AL356585.4 0.6124757 0.028723827 -4.414332733 3.22E-27 2.59E-26

FP325317.1 0.171195522 0.021263963 -3.009162573 3.22E-27 2.59E-26

AC027702.2 0.290416508 0.932654071 1.683218609 3.25E-27 2.61E-26

SLC39A14 15.33448104 51.30762502 1.742393903 3.30E-27 2.65E-26

AP005899.1 0.151139008 0.820445992 2.440532312 3.40E-27 2.73E-26

C22orf34 0.182433978 0.616539348 1.756818517 3.48E-27 2.79E-26

AC034238.2 0.004953148 0.067415543 3.766663648 3.60E-27 2.89E-26

SPATA18 3.055894821 10.28690348 1.75114198 3.70E-27 2.96E-26

AC069120.1 1.115246096 0.178333828 -2.6447098 3.72E-27 2.97E-26

GZMK 1.119425467 11.76101515 3.393182213 3.73E-27 2.98E-26

AC022432.1 0.039475432 0.639032419 4.01686216 3.74E-27 2.99E-26

UBTFL6 0.086737092 0.581771542 2.745731753 3.82E-27 3.05E-26

HLA-P 0.020674745 0.229237775 3.470903328 3.83E-27 3.06E-26

MRGPRF-AS1 0.06068753 0.018112871 -1.744384879 3.87E-27 3.09E-26

CDC20 0.978308635 3.118393183 1.672441262 4.00E-27 3.19E-26

ASIC2 0.447629276 0.124351111 -1.847885002 4.05E-27 3.23E-26

DLX2 0.01346866 0.079492544 2.561213198 4.07E-27 3.24E-26

AGAP2-AS1 1.581780747 5.362099825 1.761248438 4.16E-27 3.31E-26

TLR2 2.401574331 7.391895057 1.621963813 4.19E-27 3.33E-26

CHODL 1.624665236 0.511380237 -1.667674168 4.22E-27 3.36E-26

NPIPP1 0.558762067 2.295663486 2.03860519 4.42E-27 3.51E-26

SLFNL1 0.040011329 0.194727046 2.282972826 4.42E-27 3.51E-26

NFKBID 0.603899051 2.798481684 2.212264994 4.46E-27 3.54E-26

AC105105.2 0.003540735 0.123763534 5.127393554 4.52E-27 3.58E-26

LINC01146 0.037903737 0.20953499 2.466779202 4.67E-27 3.70E-26

IGDCC3 0.170563288 0.058611143 -1.541060282 4.69E-27 3.71E-26

EWSAT1 0.747417201 0.223222462 -1.743431561 4.74E-27 3.75E-26

PIEZO2 0.508548284 2.37200824 2.221652358 4.78E-27 3.78E-26

SNORD89 0.673311726 4.50720654 2.742887066 4.78E-27 3.78E-26

AC022509.2 0.845565649 6.118481481 2.855184968 4.78E-27 3.78E-26

COX7A2P1 4.159563642 0.941825515 -2.142900478 5.03E-27 3.97E-26

VN1R48P 0.031255656 0.008246313 -1.922296129 5.24E-27 4.13E-26

FAM153CP 0.028358289 1.114719651 5.296766622 5.24E-27 4.13E-26

AC020779.2 1.299145707 0.313116204 -2.05279317 5.32E-27 4.19E-26

KIR2DL1 0.023953729 0.128832876 2.4271786 5.44E-27 4.28E-26

HOXA11 2.285468482 0.805869011 -1.503872662 5.49E-27 4.32E-26

AC124852.1 0.341303133 0.095017822 -1.844783618 5.50E-27 4.33E-26

RDH8 0.811675895 0.110983873 -2.870553711 5.55E-27 4.36E-26

AL590428.1 0.08662498 0.375642895 2.116506799 5.60E-27 4.40E-26

NPHS2 70.15892911 0.552262563 -6.989128583 5.62E-27 4.41E-26

SIRPG-AS1 0.002872137 0.096412099 5.069017741 5.62E-27 4.42E-26

AC148477.3 0.335900098 0.096360366 -1.801520433 5.78E-27 4.54E-26

PMCH 0.011450176 0.323610897 4.820818461 5.80E-27 4.55E-26

SHBG 0.962381983 0.206551441 -2.220108506 5.84E-27 4.58E-26

CD48 1.921758003 7.55138923 1.974315405 5.84E-27 4.58E-26

AC119424.1 1.130563176 0.165090533 -2.775712311 6.22E-27 4.87E-26

MAPK8IP3 1.665459139 7.235707175 2.119214065 6.31E-27 4.93E-26

AKR1B1P4 0.031846619 0.003378438 -3.236711883 6.31E-27 4.94E-26

AL031665.1 0.015368267 0.134952211 3.134422251 6.34E-27 4.96E-26

LINC01684 0.017770514 0.096074547 2.434668878 6.45E-27 5.04E-26

MTMR11 4.119823125 13.10169104 1.669098728 6.55E-27 5.12E-26

LINC01747 0.014096781 0.066656021 2.241369404 6.64E-27 5.19E-26

AC069499.2 0.158084682 0.980618694 2.632994688 6.65E-27 5.20E-26

LINC00342 0.591508676 3.152218238 2.413896187 6.71E-27 5.23E-26

RTP4 3.341271958 11.26588039 1.753490741 6.81E-27 5.31E-26

WNT7B 3.936383163 0.245372546 -4.003824914 6.86E-27 5.35E-26

HAP1 0.253690968 0.079891511 -1.666958041 6.91E-27 5.38E-26

CFLAR-AS1 0.078687864 0.562187093 2.83683728 7.19E-27 5.59E-26

LINC00173 0.193862867 1.96901178 3.344363347 7.19E-27 5.59E-26

PAQR9-AS1 0.002874869 0.055419923 4.268836548 7.25E-27 5.63E-26

AC112777.1 0.029101341 0.147744014 2.343942164 7.34E-27 5.70E-26

TMPRSS9 0.023056947 0.159728755 2.792350678 7.36E-27 5.71E-26

AC093248.1 0.085411422 0.019207408 -2.152766177 7.39E-27 5.74E-26

SLC38A4 3.795252662 1.237788183 -1.61643148 7.64E-27 5.93E-26

ZNF806 0.031048099 0.005980397 -2.376191876 7.66E-27 5.94E-26

CLECL1 0.115265646 0.580856705 2.333219711 7.76E-27 6.02E-26

Z97192.3 0.009761804 0.104019511 3.413562628 7.94E-27 6.16E-26

ZP1 0.028479287 0.332307903 3.544535695 8.03E-27 6.22E-26

AC078864.1 1.264192232 9.301077437 2.879181993 8.13E-27 6.30E-26

LINC02280 0.004469069 0.039782911 3.154102552 8.14E-27 6.30E-26

LINC01863 0.198346986 0.064213109 -1.627086715 8.16E-27 6.32E-26

MS4A4A 2.608754557 10.09454606 1.952142915 8.38E-27 6.48E-26

LINC02709 0.156297453 0.51963389 1.733201256 8.78E-27 6.78E-26

CNTD2 3.80168081 0.137763559 -4.786371182 8.87E-27 6.84E-26

NCMAP 1.250661357 0.313875857 -1.994425235 9.12E-27 7.03E-26

SH2D1A 0.376502135 2.149816285 2.513483427 9.19E-27 7.09E-26

PTPRT 0.090126155 0.031277017 -1.526842903 9.29E-27 7.16E-26

TENM1 0.358796028 2.105451036 2.552893502 9.48E-27 7.30E-26

AL355990.2 0.088128151 0.018680205 -2.238092664 9.51E-27 7.32E-26

Z82246.1 0.239583535 0.065815529 -1.864028827 9.69E-27 7.46E-26

H2BC20P 0.604960483 1.839094015 1.60408242 9.70E-27 7.46E-26

LINC02084 0.147411539 0.832738078 2.49801334 9.70E-27 7.46E-26

IGSF9 0.34863367 0.114752717 -1.603183595 9.77E-27 7.51E-26

AL671277.2 0.466013775 1.742432827 1.902658535 9.77E-27 7.51E-26

LINC00240 0.186624332 0.698194921 1.903492763 1.00E-26 7.68E-26

HCG4P8 0.009524735 0.285907676 4.907726524 1.00E-26 7.70E-26

UBD 2.812574586 26.43713419 3.232602535 1.02E-26 7.85E-26

GPR62 0.253979704 0.085010537 -1.57899964 1.07E-26 8.17E-26

C5orf47 0.128537762 0.035592274 -1.852556244 1.08E-26 8.28E-26

AC006369.1 0.068966135 0.404364626 2.551696777 1.10E-26 8.41E-26

GAPT 0.476238973 2.054795962 2.109237551 1.11E-26 8.52E-26

LINC01857 0.149180115 1.176315933 2.979148435 1.11E-26 8.52E-26

SYN2 0.660790483 0.138703664 -2.252187015 1.15E-26 8.77E-26

TLR6 0.222823465 0.765516281 1.780531891 1.17E-26 8.91E-26

C9orf139 0.037343241 0.194996634 2.384530182 1.24E-26 9.46E-26

AL445471.1 0.010270689 0.141434656 3.783530779 1.25E-26 9.57E-26

ADAMTS20 0.001049433 0.044893387 5.418820869 1.28E-26 9.73E-26

OR2I1P 2.471635189 36.05138876 3.866517112 1.33E-26 1.01E-25

AC063976.2 0.038549317 0.265454173 2.783685615 1.39E-26 1.06E-25

CAMTA1-DT 1.149879181 0.364071883 -1.659187053 1.48E-26 1.12E-25

AC092683.1 0.013440879 0.092218746 2.778432511 1.48E-26 1.12E-25

RNASE2 0.376079281 2.019287882 2.424737872 1.50E-26 1.14E-25

SLC2A3 5.570609986 22.7955221 2.032843235 1.51E-26 1.15E-25

SIGLEC17P 0.100319781 0.349272618 1.799747441 1.52E-26 1.15E-25

AC004832.6 0.021249039 0.21442713 3.335017928 1.63E-26 1.23E-25

AC104667.2 1.149316144 0.400936751 -1.519329128 1.63E-26 1.23E-25

SH2D5 0.09833076 0.537879154 2.451567382 1.70E-26 1.28E-25

AC009084.1 0.064694354 2.012670838 4.959327615 1.71E-26 1.28E-25

AC015660.4 0.025210355 0.23617157 3.22774694 1.73E-26 1.30E-25

CPNE4 0.30061571 0.051433788 -2.547132102 1.77E-26 1.33E-25

FAM3D-AS1 0.813637887 0.147351838 -2.465121806 1.77E-26 1.33E-25

ZNF692 1.508794296 5.382348191 1.834839597 1.82E-26 1.36E-25

AL160286.1 0.299063323 0.090811007 -1.719511914 1.85E-26 1.39E-25

CXCL13 0.265823874 5.028927296 4.241708112 1.86E-26 1.39E-25

NPY2R 0.025548788 0.001998087 -3.676563446 1.90E-26 1.42E-25

RPA4 0.012372918 0.094859647 2.93860871 1.93E-26 1.45E-25

LCP1 9.375988889 33.71652484 1.84641308 1.98E-26 1.48E-25

LINC02701 0.491713883 0.155782615 -1.658284846 1.99E-26 1.49E-25

SCAT1 0.011204527 0.195631277 4.125983329 2.03E-26 1.52E-25

LINGO1 1.026240961 4.062510184 1.985001915 2.05E-26 1.53E-25

AC090337.1 0.008727053 0.132235523 3.921471386 2.12E-26 1.58E-25

IL19 0.0511435 0.008534322 -2.583202386 2.17E-26 1.62E-25

MYO1A 0.01971242 0.139412902 2.822187277 2.18E-26 1.63E-25

TTC24 0.011399278 0.161347788 3.823159424 2.20E-26 1.64E-25

LINC00676 0.050419321 0.014526373 -1.795302177 2.24E-26 1.67E-25

POU5F1P3 0.05484929 0.344005176 2.648885415 2.28E-26 1.70E-25

AC022532.1 0.064638127 0.310948939 2.266220401 2.43E-26 1.80E-25

NPIPB4 0.102648719 0.427041755 2.056661514 2.56E-26 1.90E-25

AC008385.1 0.30193715 0.021885089 -3.786228087 2.59E-26 1.93E-25

LINC01788 1.625362352 0.428770051 -1.922485337 2.63E-26 1.95E-25

AC093867.1 1.401101753 14.45199944 3.366635466 2.63E-26 1.95E-25

SLCO1A2 0.362725052 0.075019757 -2.273533891 2.66E-26 1.97E-25

HDAC10 0.607280494 1.769920006 1.543249221 2.83E-26 2.09E-25

LINC02613 0.291460966 0.099477052 -1.550867026 2.87E-26 2.12E-25

PKD2L1 0.03692825 0.350477956 3.246526889 2.87E-26 2.12E-25

EXOC3L1 1.931327681 5.710508641 1.564026292 2.94E-26 2.17E-25

ERFL 0.029979616 0.227607406 2.924493704 2.96E-26 2.19E-25

DTX2P1-UPK3BP1-PMS2P11 0.082123084 0.289376934 1.81709021 2.98E-26 2.20E-25

AP003717.1 0.018590934 0.61040162 5.037087644 3.04E-26 2.24E-25

MAGI2-AS3 5.701242833 1.921800221 -1.568818081 3.07E-26 2.27E-25

MNDA 2.405670243 8.574885096 1.83367844 3.07E-26 2.27E-25

AC105105.1 0.036781249 0.405513804 3.462708645 3.24E-26 2.38E-25

AVPR1B 0.132594746 1.831644103 3.788043694 3.26E-26 2.40E-25

HPSE2 0.909455705 0.169838211 -2.420842295 3.26E-26 2.40E-25

PMP2 0.144978829 0.027490539 -2.398835147 3.28E-26 2.41E-25

ITK 0.260597012 1.165764617 2.161384071 3.29E-26 2.42E-25

CD5 0.624199294 2.728489097 2.128023648 3.32E-26 2.44E-25

IFI30 0.192021464 0.970552 2.337537931 3.39E-26 2.49E-25

AC139769.3 0.574933064 0.136245659 -2.077183743 3.55E-26 2.61E-25

AC005515.1 0.016765179 0.509799721 4.926390772 3.59E-26 2.63E-25

AC239600.1 0.174565276 0.047403159 -1.880711507 3.63E-26 2.66E-25

TOX2 0.931117296 3.140214458 1.753828265 3.74E-26 2.74E-25

PCSK1N 22.2394353 7.159968765 -1.635094957 3.86E-26 2.82E-25

PDCD1 0.257014739 2.666049357 3.374780489 3.86E-26 2.82E-25

OR5BA1P 0.029969154 0.388261 3.695476527 4.04E-26 2.95E-25

LINC01948 1.777017847 0.613197798 -1.535033749 4.10E-26 2.99E-25

AGBL3 0.531623663 1.583935244 1.57503613 4.10E-26 2.99E-25

AQP7P4 0.033020641 0.00210588 -3.970872774 4.13E-26 3.01E-25

PLA2G5 0.049894417 0.344077579 2.785783578 4.29E-26 3.13E-25

AC120114.3 0.170486944 0.894307293 2.391109377 4.29E-26 3.13E-25

ABCA17P 0.073018171 0.397766159 2.4455931 4.36E-26 3.17E-25

PRKCQ 8.453836333 2.651547517 -1.67277158 4.39E-26 3.20E-25

NCR3 0.203123655 0.730691176 1.846903528 4.46E-26 3.24E-25

COL4A2 50.27486917 145.4251536 1.532367501 4.56E-26 3.31E-25

AC114757.1 0.027079654 0.302324918 3.480818679 4.61E-26 3.35E-25

LINC01033 0.017826231 0.168792637 3.243178356 4.65E-26 3.38E-25

FBP1 122.671715 25.50755822 -2.265805934 4.77E-26 3.46E-25

HNRNPA1P21 0.109964533 0.664512247 2.595257507 5.03E-26 3.64E-25

PCDH17 1.581885469 6.474528895 2.033130071 5.07E-26 3.67E-25

AL049634.1 0.006192444 0.055599392 3.166488377 5.16E-26 3.74E-25

ENOX1 2.132766885 0.588526596 -1.857546766 5.22E-26 3.78E-25

BCAN 0.156655338 0.616614545 1.976774987 5.38E-26 3.89E-25

TMEM255B 0.968235996 2.804660538 1.53439553 5.51E-26 3.98E-25

TIMP1 73.32822694 291.5273354 1.991190605 5.51E-26 3.98E-25

CTXN3 12.25457444 1.280067765 -3.259028294 5.53E-26 3.99E-25

OR52K3P 0.024937264 0.192767825 2.950489178 5.60E-26 4.04E-25

LINC02751 0.055488659 0.014899582 -1.896921086 5.61E-26 4.05E-25

CXorf65 0.032662397 0.371218888 3.506567554 5.73E-26 4.13E-25

AC022431.1 0.650883495 0.109665121 -2.569294576 5.75E-26 4.14E-25

AGER 0.735303564 3.212644635 2.127349524 5.81E-26 4.18E-25

AC073610.1 0.121997644 0.47124084 1.949611285 5.83E-26 4.20E-25

IFITM4P 0.111226415 0.712468944 2.679327677 5.84E-26 4.21E-25

HM13-IT1 0.27159886 1.359948026 2.324002186 6.03E-26 4.34E-25

GCM1 0.656977023 0.173973177 -1.916978024 6.21E-26 4.47E-25

PRRT2 0.211253089 1.18761652 2.491024728 6.31E-26 4.54E-25

AC078802.1 0.369983843 0.098802048 -1.904849421 6.43E-26 4.62E-25

AC087379.2 7.33464733 1.378487024 -2.411641912 6.60E-26 4.74E-25

HEY1 2.223286863 6.654564612 1.581650172 6.65E-26 4.77E-25

SHISAL2A 0.125346351 0.443073601 1.821626369 6.65E-26 4.77E-25

AC005616.1 1.28319189 0.023164062 -5.791704871 6.70E-26 4.80E-25

AC244035.1 0.071883294 0.658871218 3.196268071 6.72E-26 4.82E-25

AC127502.2 0.607823922 2.179395073 1.842202385 6.78E-26 4.86E-25

SCGB1C2 0.105515255 0.022033735 -2.259665597 6.79E-26 4.86E-25

MTTP 2.230482404 0.655611941 -1.766441731 7.28E-26 5.21E-25

GBP1 6.002254292 21.85772452 1.864566872 8.03E-26 5.73E-25

PLA2G4A 6.517388921 2.156617862 -1.595523526 8.22E-26 5.86E-25

IL27 0.030107474 0.16307528 2.437344538 8.25E-26 5.88E-25

AC015819.2 0.090652528 0.645324545 2.831605749 8.37E-26 5.96E-25

AC026992.2 1.618297154 0.567803464 -1.511012986 8.47E-26 6.03E-25

APOBR 1.117356768 3.537443977 1.662617393 8.53E-26 6.07E-25

AL138847.2 1.412018289 0.397092841 -1.830210517 8.66E-26 6.16E-25

ISG15 13.7994606 40.46061088 1.551906227 8.66E-26 6.16E-25

SLC5A3 38.45969168 10.00933542 -1.942001008 8.73E-26 6.21E-25

ATP2B2 0.16075763 1.697238912 3.40023054 9.06E-26 6.44E-25

AL353746.1 0.06530677 0.016073919 -2.022510827 9.08E-26 6.45E-25

AC004069.1 0.193290274 0.751973518 1.959912811 9.13E-26 6.48E-25

AC007278.1 0.020315359 0.165824187 3.029011662 9.28E-26 6.58E-25

PCDHGC5 0.0322822 0.144758182 2.164834084 9.30E-26 6.60E-25

RRN3P1 0.370900363 1.238297516 1.739254398 9.34E-26 6.62E-25

RNU2-19P 0.216529454 0.023748041 -3.188682849 9.49E-26 6.72E-25

AP001029.2 0.017650845 0.120197498 2.767597688 9.62E-26 6.81E-25

RN7SL124P 0.003968255 0.406173429 6.677447317 1.01E-25 7.12E-25

CRYGS 0.298278892 1.537256569 2.365624178 1.01E-25 7.17E-25

SPATA46 0.111556127 0.037011536 -1.591722829 1.02E-25 7.22E-25

MAPRE1P3 0.026633905 0.00589749 -2.175091024 1.02E-25 7.23E-25

PLVAP 92.60878361 358.9141602 1.954417903 1.04E-25 7.32E-25

AC051619.2 0.048252895 0.670219514 3.795946297 1.06E-25 7.51E-25

LINC01356 0.041418877 0.165936299 2.002269158 1.09E-25 7.71E-25

AC068492.1 0.00242462 0.165523294 6.093131794 1.12E-25 7.86E-25

OSMR 9.402279542 26.62434045 1.501663308 1.12E-25 7.88E-25

AC093496.1 3.372014013 0.366625623 -3.201231009 1.12E-25 7.91E-25

FAM238A 0.007124368 0.107449506 3.914753015 1.15E-25 8.11E-25

COL27A1 1.674292196 5.849796132 1.804835019 1.22E-25 8.59E-25

AC105094.2 0.450829207 0.058603398 -2.94352475 1.26E-25 8.83E-25

AC079466.1 0.079746909 6.708832791 6.394489381 1.30E-25 9.13E-25

LY9 0.087430479 0.379791438 2.118999175 1.31E-25 9.17E-25

LINC01678 0.040804632 0.213619981 2.388241759 1.36E-25 9.49E-25

LILRA4 0.119213843 0.687380617 2.527557403 1.37E-25 9.61E-25

CYP26B1 4.758004643 1.56253881 -1.606464656 1.39E-25 9.72E-25

AC069234.4 0.109205922 0.447202577 2.033877406 1.45E-25 1.01E-24

RASL10A 0.088502374 0.379535054 2.100445088 1.45E-25 1.02E-24

AC009093.2 0.032429517 0.14199955 2.130506918 1.46E-25 1.02E-24

ANG 5.349920968 17.26753471 1.690472639 1.47E-25 1.02E-24

AL360182.2 0.007235991 0.175288976 4.59840093 1.48E-25 1.03E-24

QRFPR 0.877919125 6.781851231 2.949519189 1.48E-25 1.03E-24

TRGC2 0.210222527 1.067557081 2.34432403 1.49E-25 1.04E-24

AC004080.5 0.084569552 0.017604192 -2.264219314 1.51E-25 1.05E-24

SLC7A11 0.114650648 0.388249872 1.759740939 1.52E-25 1.06E-24

TTC29 0.116461433 0.022083757 -2.39879473 1.52E-25 1.06E-24

SEMA6B 2.768146275 8.425802233 1.605893874 1.53E-25 1.07E-24

SMTNL1 0.051976402 0.407928257 2.972386766 1.53E-25 1.07E-24

AL731567.1 0.103841281 0.831823464 3.00189729 1.53E-25 1.07E-24

AC020907.4 0.134377623 1.094545189 3.025966698 1.54E-25 1.07E-24

AC090616.6 0.094940498 0.352983243 1.894504178 1.56E-25 1.08E-24

ADAM11 0.054528636 0.238834573 2.130925718 1.57E-25 1.09E-24

TMSB15B-AS1 0.050258143 0.193003687 1.941199146 1.59E-25 1.10E-24

MIR1270 0.414501792 4.529858341 3.450015692 1.62E-25 1.12E-24

CST11 0.042849146 0.006560476 -2.707394158 1.66E-25 1.15E-24

AC022101.1 0.049738169 0.017294177 -1.524067072 1.70E-25 1.18E-24

SHC3 0.59571092 0.209852883 -1.505234119 1.71E-25 1.19E-24

ASB10 0.024604958 0.002245417 -3.453893481 1.75E-25 1.22E-24

RNU2-52P 0.087830817 0.683435523 2.960006112 1.76E-25 1.22E-24

SLITRK3 0.03743456 0.005942445 -2.655242156 1.79E-25 1.24E-24

TRAF3IP3 0.35790943 1.356799832 1.922541437 1.83E-25 1.27E-24

NHLH1 0.024064524 0.102354738 2.088598104 1.83E-25 1.27E-24

OMG 0.037640447 1.127563107 4.904780599 1.83E-25 1.27E-24

EIF5AP3 0.028420873 0.33066148 3.540332227 1.84E-25 1.27E-24

MCHR1 0.087334375 4.565749915 5.708158423 1.85E-25 1.28E-24

PLAU 72.7867979 15.84399196 -2.199740923 1.85E-25 1.28E-24

AHSA2P 1.340136279 5.993290927 2.160968691 1.88E-25 1.29E-24

TNFSF8 0.475100429 1.746131819 1.877858061 1.89E-25 1.30E-24

AP003774.2 0.049876446 0.313073229 2.650069577 1.90E-25 1.31E-24

AL450322.2 0.006852714 0.138334252 4.335339153 2.02E-25 1.39E-24

ABAT 20.19248172 2.561271717 -2.978886016 2.05E-25 1.41E-24

PANO1 0.092043448 0.265765411 1.529766423 2.07E-25 1.42E-24

PKD1L1 0.047254573 0.177977386 1.913168075 2.07E-25 1.42E-24

CNTNAP1 0.773412464 2.399863273 1.633642295 2.10E-25 1.44E-24

ZDHHC20P1 0.100378211 0.420559926 2.066865243 2.12E-25 1.46E-24

AC004067.1 0.180212229 0.58833131 1.706931899 2.16E-25 1.48E-24

CCDC141 0.033631046 0.157404245 2.226608875 2.18E-25 1.49E-24

ORAOV1P1 0.417128785 2.602291587 2.641217848 2.21E-25 1.51E-24

AC124944.1 0.107699309 0.818837219 2.926567687 2.21E-25 1.52E-24

CD300LB 0.244598136 0.797307115 1.704722131 2.24E-25 1.54E-24

LINC01744 0.001988274 0.036021168 4.17925625 2.25E-25 1.54E-24

GJA1 17.40098624 60.51947384 1.798230369 2.30E-25 1.57E-24

AC040162.3 0.083759987 0.390618159 2.22142589 2.31E-25 1.58E-24

AC100854.1 0.359447124 1.105694727 1.621101658 2.35E-25 1.60E-24

ADAMTS2 1.123038863 4.229426687 1.913054262 2.35E-25 1.60E-24

GRIN2D 0.101210758 0.388634528 1.941051442 2.35E-25 1.60E-24

LINC00900 0.10549299 0.414216534 1.973238009 2.36E-25 1.61E-24

FBLN1 16.56879004 5.448985631 -1.60440866 2.38E-25 1.63E-24

JAML 0.704410566 2.284856423 1.697615058 2.38E-25 1.63E-24

AC004988.1 0.015603394 0.191669256 3.61868719 2.47E-25 1.68E-24

FAM177B 0.113337707 0.417375716 1.88071874 2.49E-25 1.70E-24

C16orf54 0.588778888 2.061638855 1.807993787 2.51E-25 1.71E-24

MMP9 0.839206088 13.7903335 4.038488393 2.51E-25 1.71E-24

ABHD11-AS1 0.579551177 3.120356187 2.428702754 2.61E-25 1.77E-24

ENTPD8 1.445470081 0.43740238 -1.724505773 2.62E-25 1.78E-24

HRH2 0.92316926 7.033842333 2.929645906 2.68E-25 1.83E-24

LINC01152 0.080504212 0.571609066 2.827892625 2.70E-25 1.84E-24

MEP1A 0.007248867 0.052831948 2.865583131 2.74E-25 1.86E-24

ANKLE1 0.041767373 0.176035978 2.075422001 2.91E-25 1.98E-24

AC139256.3 0.151196594 0.462560815 1.613217411 2.94E-25 1.99E-24

TAS2R2P 0.527392786 0.173025386 -1.607894113 2.94E-25 1.99E-24

DUXAP9 0.017039493 0.101480873 2.574253494 2.95E-25 2.00E-24

SNORD99 0.534826967 6.052408191 3.500365175 2.98E-25 2.02E-24

RN7SL668P 0.078379889 0.025754931 -1.605634857 3.02E-25 2.05E-24

ALOX5 2.980667235 12.6033022 2.08009456 3.05E-25 2.06E-24

GRAP2 0.204742758 0.68192207 1.735794455 3.09E-25 2.09E-24

XAF1 0.609519072 2.512464032 2.043359673 3.12E-25 2.11E-24

LRRC71 0.02224047 0.190980034 3.102162655 3.18E-25 2.15E-24

PDILT 0.148819723 0.021823229 -2.76962928 3.21E-25 2.17E-24

CDH23 0.107752377 0.573128409 2.411138717 3.21E-25 2.17E-24

PTH1R 73.50216802 8.079874382 -3.185382036 3.31E-25 2.23E-24

AP001351.1 0.070871625 0.25333622 1.837773328 3.33E-25 2.25E-24

CLEC5A 0.340400289 1.420781834 2.061380874 3.36E-25 2.26E-24

SORD2P 1.637897763 0.511294105 -1.679620008 3.43E-25 2.31E-24

LINC01541 0.020812675 0.004154262 -2.324798164 3.52E-25 2.37E-24

AC067945.2 0.046420783 0.295643446 2.671015532 3.57E-25 2.41E-24

PIK3CD-AS1 0.010928617 0.070712311 2.693850567 3.58E-25 2.41E-24

ADORA2BP1 0.774674054 0.08959517 -3.11209656 3.71E-25 2.49E-24

SH3PXD2B 1.633679643 4.718738435 1.530276098 3.75E-25 2.52E-24

MYLK3 0.957832387 0.061304478 -3.965708847 3.81E-25 2.56E-24

LINC02138 0.096006912 0.005064452 -4.244660305 3.84E-25 2.58E-24

AL132780.1 1.342400154 0.406507441 -1.723461121 3.87E-25 2.59E-24

AL450469.2 0.146768668 0.004592072 -4.998254866 3.91E-25 2.62E-24

LY96 4.158937982 13.22779512 1.669285529 3.92E-25 2.63E-24

TGFA 10.33891817 32.95195043 1.672278627 4.01E-25 2.69E-24

TRGV7 0.313894805 1.206958911 1.943023505 4.03E-25 2.70E-24

AL365475.1 0.013881199 0.118143347 3.089334339 4.06E-25 2.72E-24

TRGV2 0.052945686 0.356171188 2.749985772 4.09E-25 2.74E-24

CHRNA6 0.030612507 0.255630475 3.061866717 4.13E-25 2.76E-24

TRAV19 0.158428723 1.430488225 3.174601803 4.24E-25 2.84E-24

FOXN1 0.164405319 0.031413523 -2.387799326 4.27E-25 2.86E-24

GRPEL2-AS1 0.060939216 0.483846619 2.989106944 4.29E-25 2.87E-24

AC002091.1 0.2307892 0.802366979 1.79768652 4.34E-25 2.90E-24

AF111169.3 0.075761938 0.455529806 2.588000311 4.35E-25 2.90E-24

ALDOC 6.832748431 32.67437267 2.257621622 4.42E-25 2.95E-24

AL138930.1 0.007880179 0.097279808 3.62584012 4.43E-25 2.95E-24

HORMAD1 0.007232773 0.077209778 3.416162734 4.82E-25 3.21E-24

MTDHP3 1.905147479 0.553665066 -1.782817281 4.83E-25 3.22E-24

AC008957.3 0.017745285 0.259322862 3.869241773 4.83E-25 3.22E-24

AL121957.1 0.222320276 0.05146793 -2.110893862 4.84E-25 3.22E-24

LDHAP2 0.048608286 0.179174237 1.882089041 4.84E-25 3.22E-24

AL683842.1 0.043379087 0.432960587 3.319164122 4.89E-25 3.25E-24

APOL1 25.99648115 107.0530704 2.041937914 5.05E-25 3.36E-24

RBP3 0.015141174 0.002681823 -2.497191173 5.17E-25 3.44E-24

BTBD11 2.219668311 0.779528753 -1.509669965 5.20E-25 3.45E-24

MSC-AS1 1.220437258 3.699326263 1.599864417 5.20E-25 3.45E-24

ANO1 2.607134011 9.385392956 1.847952409 5.24E-25 3.48E-24

FAM9C 0.007104883 0.031395487 2.143674305 5.27E-25 3.49E-24

LINC02110 0.120013398 0.011851192 -3.340091347 5.28E-25 3.50E-24

AC090510.2 0.083297584 0.438557994 2.396421075 5.35E-25 3.55E-24

RERGL 5.761676472 1.992618295 -1.531823279 5.36E-25 3.55E-24

C10orf55 0.939172528 0.194072759 -2.274792578 5.44E-25 3.60E-24

ITGA4 1.272222754 4.459601974 1.809563658 5.48E-25 3.63E-24

LNX1-AS2 0.329619278 0.090882594 -1.858724698 5.57E-25 3.69E-24

GTF2IRD1P1 0.046302445 0.016243636 -1.511213759 5.61E-25 3.71E-24

KCNMA1 0.704355198 3.483006472 2.305958102 5.69E-25 3.76E-24

AC008875.1 0.045316393 0.307309455 2.761587203 5.88E-25 3.88E-24

PFN1P6 0.17861499 1.02713464 2.523700244 5.89E-25 3.89E-24

AC026412.3 0.159989171 0.501250074 1.647556292 6.40E-25 4.22E-24

SETP1 0.010923304 0.092501306 3.082064374 6.57E-25 4.33E-24

CREB5 1.370992689 3.91888113 1.515220935 6.59E-25 4.34E-24

AC254629.1 0.00621896 0.116597611 4.228720996 6.66E-25 4.38E-24

RXFP4 0.20715219 0.039171032 -2.402832021 6.68E-25 4.40E-24

DCLK1 0.495332021 2.182194352 2.139311805 6.89E-25 4.53E-24

GAPDHP63 0.15671585 0.459136792 1.550772946 6.94E-25 4.57E-24

ALPK2 2.429858385 9.829275626 2.016212866 6.94E-25 4.57E-24

PMFBP1 0.043101898 0.189445665 2.135960816 7.05E-25 4.63E-24

NGF 1.724507074 7.614480068 2.142561483 7.05E-25 4.63E-24

LINC01014 0.576123304 0.166149918 -1.793892037 7.05E-25 4.63E-24

PXDN 4.119729611 13.12571666 1.671774637 7.10E-25 4.66E-24

FLT1 11.79856785 45.57086422 1.94949998 7.10E-25 4.66E-24

CRIP1 0.368523833 1.357788786 1.881429245 7.15E-25 4.69E-24

MYH15 0.03583148 0.192661271 2.426767034 7.15E-25 4.69E-24

LMNTD2-AS1 0.309567291 4.084617159 3.721875914 7.15E-25 4.69E-24

C11orf72 0.066088295 0.022477817 -1.555892862 7.22E-25 4.73E-24

AP000525.1 0.029578894 0.315738456 3.416089953 7.40E-25 4.85E-24

LINC00437 0.032277366 0.006051693 -2.415112148 7.45E-25 4.88E-24

HLA-DQB2 2.422724475 17.12781795 2.821639118 7.47E-25 4.90E-24

AC012613.1 0.006202082 0.1573715 4.665277955 7.51E-25 4.92E-24

LINC01833 0.234033707 0.048207131 -2.27939785 7.57E-25 4.96E-24

GPR82 0.120444171 0.526660157 2.128507751 7.59E-25 4.97E-24

FAM72C 0.009679777 0.04974058 2.361377561 7.67E-25 5.02E-24

C11orf16 0.232792715 0.061445694 -1.921662086 7.80E-25 5.10E-24

AC243964.3 2.639445889 0.727702807 -1.858813809 7.81E-25 5.11E-24

PRR16 0.459714799 1.570134612 1.772077237 7.81E-25 5.11E-24

AC116366.2 0.244980328 1.022029135 2.060698511 7.87E-25 5.14E-24

RPS19P3 0.207425185 0.728064602 1.811475395 8.05E-25 5.26E-24

PLPPR5 0.019206032 1.661342626 6.434646341 8.05E-25 5.26E-24

AC015849.5 0.007267648 0.040622061 2.482702996 8.09E-25 5.28E-24

SPINK13 0.370466883 5.803573523 3.96952502 8.20E-25 5.35E-24

ZNF831 0.072928143 0.362103727 2.311855468 8.23E-25 5.37E-24

TRBJ2-3 0.189229992 1.843410977 3.284165078 8.53E-25 5.56E-24

FAM225A 0.021433487 0.088482101 2.045519034 8.69E-25 5.66E-24

AC023825.2 0.01586742 0.26004914 4.03464477 8.88E-25 5.78E-24

CDHR3 0.469942198 1.335962468 1.507324254 8.92E-25 5.81E-24

GPR18 0.146794618 0.693482736 2.240060894 9.12E-25 5.93E-24

GGACT 11.30962573 2.332852925 -2.277383927 9.19E-25 5.98E-24

LINC02328 0.16953704 0.553991658 1.708263746 9.33E-25 6.06E-24

NT5C3AP2 0.013586636 0.162024687 3.575953418 9.35E-25 6.08E-24

KIR3DL2 0.017168233 0.087998366 2.357735169 9.69E-25 6.29E-24

TH 0.054438489 0.012485069 -2.124423228 1.02E-24 6.63E-24

UBAP1L 0.154287469 0.735236811 2.252588108 1.05E-24 6.80E-24

GPC2 0.041112318 0.165825005 2.012018952 1.09E-24 7.05E-24

AC021491.4 0.481504822 0.061433829 -2.970445044 1.12E-24 7.23E-24

GABRE 0.390434599 3.042250423 2.961986102 1.14E-24 7.36E-24

CP 5.26941831 67.03234319 3.669141749 1.15E-24 7.42E-24

OPRD1 0.019345901 0.076695117 1.987106793 1.15E-24 7.47E-24

IQCF6 0.20945999 0.060833701 -1.783732015 1.16E-24 7.50E-24

SLC7A3 0.078397615 0.014160251 -2.46896294 1.16E-24 7.52E-24

TDRD6 0.080143275 0.30214995 1.914611342 1.20E-24 7.74E-24

AL035665.1 0.01413732 0.110161044 2.962033603 1.24E-24 7.98E-24

PLA1A 7.496634472 23.00606604 1.617699344 1.25E-24 8.07E-24

PMEPA1 8.418645319 25.88378275 1.620388466 1.25E-24 8.07E-24

AC137770.1 0.011964181 0.194714506 4.024566857 1.28E-24 8.21E-24

AC084876.1 0.0829491 0.572314339 2.786509521 1.30E-24 8.33E-24

KCNJ2 1.090567771 3.974674312 1.865757224 1.31E-24 8.41E-24

TLR7 0.653760812 2.853060323 2.125675444 1.31E-24 8.41E-24

AC009159.1 0.021385306 0.273619902 3.677481417 1.31E-24 8.45E-24

OR7E47P 0.535337151 3.257646792 2.605310508 1.34E-24 8.59E-24

AL513327.1 0.253826551 1.05298526 2.052570348 1.35E-24 8.65E-24

AC006272.1 0.036270779 0.25145554 2.793423683 1.37E-24 8.80E-24

AC016559.3 0.085199382 0.017456378 -2.287088713 1.38E-24 8.87E-24

HPD 114.4395138 3.977336003 -4.846639018 1.39E-24 8.89E-24

AC021188.1 0.113572811 0.398757986 1.811895913 1.40E-24 8.96E-24

AC105105.3 0.027002818 0.318164884 3.558592716 1.40E-24 9.00E-24

TMSB10P1 0.292893075 1.341456525 2.19535431 1.42E-24 9.11E-24

ZNF296 0.322364889 0.926108443 1.522486518 1.44E-24 9.21E-24

DDX47 0.147418555 0.473119744 1.682287248 1.44E-24 9.21E-24

EXOC3L4 0.918692661 4.06288031 2.144848655 1.48E-24 9.48E-24

AC027601.2 0.052105447 0.28295912 2.441087541 1.49E-24 9.54E-24

LINC02198 0.26627091 0.066258573 -2.006715794 1.51E-24 9.67E-24

NLRP12 0.058338486 0.196683869 1.753358794 1.52E-24 9.68E-24

AP002954.1 0.107101301 0.779962553 2.864428852 1.54E-24 9.81E-24

CSF1R 7.452381472 23.2148752 1.639276095 1.55E-24 9.89E-24

DHRS9 0.14599854 0.553026016 1.921393407 1.57E-24 1.00E-23

AC107021.1 0.222419802 1.197723969 2.428938314 1.58E-24 1.01E-23

RPL7L1P1 0.029380245 0.00294837 -3.316856971 1.58E-24 1.01E-23

ADAMTS15 6.290947799 1.576558163 -1.996498995 1.60E-24 1.02E-23

SH3PXD2A-AS1 0.0193395 0.101630364 2.393709105 1.60E-24 1.02E-23

TMEM262 0.099752466 0.298375424 1.580704306 1.61E-24 1.02E-23

AC010247.1 0.055769922 0.191990872 1.783478572 1.64E-24 1.05E-23

RBM46 0.009433514 0.122246925 3.695859049 1.68E-24 1.07E-23

PTPRC 2.958021914 10.97800215 1.891910882 1.70E-24 1.08E-23

ATF7-NPFF 0.146996536 0.752397199 2.355712318 1.70E-24 1.08E-23

LYZ 20.62648308 114.1078376 2.467828128 1.72E-24 1.09E-23

ADCY7 0.313740974 1.251110423 1.995563267 1.74E-24 1.10E-23

KIR3DX1 0.00607831 0.050713143 3.060617489 1.75E-24 1.11E-23

AL024497.2 0.118698618 0.032723303 -1.858912853 1.77E-24 1.12E-23

GLIS1 1.138620787 4.359423592 1.936850049 1.83E-24 1.16E-23

PA2G4P4 0.069492349 0.266114641 1.937121832 1.83E-24 1.16E-23

AL031714.1 0.188507858 0.798453436 2.08258361 1.83E-24 1.16E-23

ECRG4 9.485873918 2.445620058 -1.955580403 1.89E-24 1.19E-23

SORD 13.67269904 3.156407983 -2.114942466 1.93E-24 1.22E-23

ARHGAP33 0.533823811 2.370905437 2.15100256 1.93E-24 1.22E-23

SLC28A1 11.12268132 44.0771582 1.986526594 1.97E-24 1.25E-23

AC007342.3 0.113051661 0.030577786 -1.886426315 1.99E-24 1.25E-23

AC008013.2 0.02449986 0.282473929 3.527272289 2.00E-24 1.26E-23

LILRA1 0.314534836 0.921088995 1.550120743 2.00E-24 1.26E-23

FBXO39 0.028437845 0.143730192 2.33747911 2.01E-24 1.27E-23

AL512347.1 0.186633722 0.044979759 -2.052861832 2.03E-24 1.28E-23

AP001010.1 0.038194448 0.306582946 3.004842584 2.03E-24 1.28E-23

TRAT1 0.215105315 1.13876781 2.404358538 2.03E-24 1.28E-23

RNASE6 6.939145097 20.2870591 1.547729902 2.05E-24 1.29E-23

SEC16B 0.033312639 0.11602679 1.800316411 2.05E-24 1.29E-23

AL355493.1 0.13186075 0.036285871 -1.861535395 2.05E-24 1.29E-23

AC129507.4 1.895806248 0.539725507 -1.812513752 2.09E-24 1.31E-23

RN7SL832P 0.713108685 0.248929532 -1.518384579 2.11E-24 1.33E-23

AC008742.1 0.00810643 0.128129258 3.9823894 2.11E-24 1.33E-23

AC018816.1 0.359561465 1.112675037 1.629721988 2.15E-24 1.35E-23

AP005717.1 0.260708766 0.06808486 -1.937033166 2.21E-24 1.39E-23

AL161935.1 0.041822578 0.285633522 2.771811422 2.26E-24 1.42E-23

AC009093.1 0.025916049 0.148867003 2.522106317 2.26E-24 1.42E-23

AP003352.1 0.45892139 1.335152679 1.540685771 2.28E-24 1.43E-23

GARS1-DT 0.361001413 1.171895885 1.698768014 2.32E-24 1.45E-23

AC027419.2 0.067792712 0.009820298 -2.787291481 2.35E-24 1.48E-23

LINC01233 0.119532648 0.040548138 -1.559697134 2.36E-24 1.48E-23

FCRL3 0.075300144 0.560407206 2.895750971 2.37E-24 1.48E-23

AC099522.1 0.370856443 0.103850968 -1.836346166 2.38E-24 1.49E-23

NPIPB3 0.098969769 0.44901974 2.181719052 2.44E-24 1.53E-23

AC096541.1 0.132821227 0.50128275 1.916138863 2.46E-24 1.54E-23

RN7SL541P 0.043282614 0.596650087 3.785025556 2.47E-24 1.54E-23

ABCA12 0.177234322 0.977853194 2.463959876 2.47E-24 1.55E-23

SPRR2A 0.269145279 0.064052786 -2.071051885 2.51E-24 1.57E-23

AC009902.3 0.018278657 0.077296974 2.080251861 2.53E-24 1.58E-23

AC006026.3 2.111253449 0.707639774 -1.577012735 2.55E-24 1.59E-23

AC116407.2 0.225808339 0.84806892 1.909082748 2.59E-24 1.61E-23

PCAT18 0.161599435 0.049874813 -1.696038813 2.61E-24 1.63E-23

STARD9 0.373205973 1.107848869 1.569717104 2.62E-24 1.64E-23

TFAP2C 1.775898371 0.379051297 -2.228084016 2.65E-24 1.65E-23

FOXJ3 8.211005361 26.07583724 1.667082793 2.66E-24 1.66E-23

NRXN1 0.075913247 0.019795373 -1.939188403 2.68E-24 1.67E-23

NRBP2 3.968318847 11.25848348 1.504412656 2.68E-24 1.67E-23

ADAM19 0.823084474 2.493044472 1.598796211 2.68E-24 1.67E-23

PLGLA 0.288989649 0.008413659 -5.102140729 2.72E-24 1.69E-23

AC010247.2 0.075203435 0.244391859 1.700325756 2.77E-24 1.72E-23

ACAN 0.723247813 4.431471681 2.615223933 2.78E-24 1.73E-23

RNY3P16 0.206052521 1.402531039 2.76694868 2.79E-24 1.73E-23

LMNTD2 0.595623692 2.444870214 2.037284837 2.80E-24 1.74E-23

MT1HL1 0.229134497 0.054689724 -2.066853001 2.84E-24 1.77E-23

XPNPEP2 59.53000315 1.464139854 -5.345491699 2.88E-24 1.79E-23

CLMAT3 0.025207612 0.135804481 2.429599704 2.89E-24 1.79E-23

SLC6A8 25.86596617 81.88765431 1.662590881 2.95E-24 1.83E-23

SPTA1 0.012730472 0.065927635 2.372597419 3.02E-24 1.87E-23

ADAMTS19 0.283779219 0.015620244 -4.183280037 3.04E-24 1.88E-23

NACAP8 1.336140896 0.446838355 -1.580247216 3.05E-24 1.89E-23

YES1P1 0.033598984 0.260090488 2.952524128 3.06E-24 1.89E-23

COL8A1 3.147452988 12.4832125 1.987732519 3.06E-24 1.89E-23

CLIC5 22.71876015 1.727578613 -3.717060837 3.10E-24 1.92E-23

IL18RAP 0.202747075 0.690481198 1.767921031 3.10E-24 1.92E-23

LINC01687 0.180080195 0.059572242 -1.595927366 3.19E-24 1.97E-23

STARD4-AS1 0.414538776 1.478126912 1.83419118 3.24E-24 2.00E-23

LGALS12 0.207157898 2.365075111 3.513083275 3.24E-24 2.00E-23

AL135818.1 0.11022747 0.382355217 1.794429758 3.36E-24 2.07E-23

AC099850.4 0.640911625 2.18794834 1.771381333 3.39E-24 2.09E-23

TLR3 5.539796903 16.01348697 1.531382501 3.44E-24 2.12E-23

DLX4 0.047157219 0.2955714 2.647956119 3.46E-24 2.13E-23

AL807752.1 0.038033281 0.147705722 1.95739141 3.52E-24 2.17E-23

AC013724.1 0.017702657 0.18205377 3.362326774 3.54E-24 2.18E-23

KCNK7 0.086428862 0.280360638 1.69769875 3.64E-24 2.24E-23

POU2F3 0.385980503 0.087970593 -2.133434731 3.75E-24 2.31E-23

SMC1B 0.018411102 0.094258147 2.356041319 3.83E-24 2.35E-23

AC243732.1 0.063514948 0.265728701 2.064785979 3.86E-24 2.37E-23

MIR924HG 0.039702864 0.211046539 2.41024618 4.02E-24 2.46E-23

TM4SF19 0.02969557 0.285459178 3.264964797 4.11E-24 2.52E-23

PSAT1 44.95856672 7.969221281 -2.496085381 4.15E-24 2.54E-23

LINC02615 0.413451458 1.532030312 1.889654979 4.15E-24 2.54E-23

AC105105.4 0.004282557 0.116559359 4.766448542 4.26E-24 2.61E-23

KLK7 4.020145442 0.074967631 -5.744836073 4.42E-24 2.70E-23

TGFBI 31.25246186 219.1229792 2.809699041 4.43E-24 2.71E-23

OR51E2 0.253898779 1.216764734 2.260724882 4.56E-24 2.79E-23

AC010973.2 0.170749117 0.775083363 2.182473371 4.60E-24 2.81E-23

RNASE10 0.028264632 0.145127023 2.36024635 4.78E-24 2.91E-23

DDX43P3 0.011118173 0.380032373 5.095130724 4.79E-24 2.92E-23

DPRXP4 0.107680444 0.518838141 2.26852828 5.05E-24 3.07E-23

HSD17B3 0.084911115 0.735941346 3.115565469 5.09E-24 3.09E-23

HEATR9 0.007505469 0.08598201 3.518020652 5.13E-24 3.12E-23

RNASE3 0.035041144 0.190349086 2.441525863 5.26E-24 3.20E-23

BX284668.2 0.372799537 0.103543448 -1.848163797 5.33E-24 3.24E-23

CHMP1B2P 0.22576647 0.073318547 -1.622581147 5.59E-24 3.39E-23

AC008115.3 0.325154265 1.159394281 1.834175022 5.75E-24 3.49E-23

HLA-V 0.259283128 1.137347471 2.133072846 6.05E-24 3.67E-23

HLA-DQB1-AS1 0.995988793 4.980997234 2.322233196 6.05E-24 3.67E-23

TTC4P1 0.015392382 0.436816111 4.826737692 6.09E-24 3.69E-23

LY6E 33.87197125 108.7793846 1.683241314 6.10E-24 3.69E-23

VENTX 0.17365028 0.678147674 1.965414731 6.10E-24 3.69E-23

LRRC36 0.171985925 0.635806182 1.886296539 6.19E-24 3.74E-23

AC018695.6 0.032841499 0.60090292 4.193540036 6.27E-24 3.79E-23

AC080112.4 0.108886245 0.452154788 2.05399502 6.28E-24 3.79E-23

AC104966.1 0.067775142 0.703967032 3.376679731 6.35E-24 3.84E-23

AC022509.3 0.602419953 1.8686019 1.63311778 6.37E-24 3.85E-23

TMEM229A 0.026563244 0.007010727 -1.921795274 6.46E-24 3.90E-23

SPEM2 0.001861683 0.036323768 4.286234508 6.50E-24 3.92E-23

AC005920.3 0.089734367 0.020549668 -2.126545553 6.52E-24 3.93E-23

EPS8L1 4.630310065 1.510631278 -1.615957242 6.65E-24 4.00E-23

GAPDHP2 0.075626262 0.228777572 1.596986417 6.65E-24 4.00E-23

RGCC 28.49081399 86.71599104 1.605801221 6.65E-24 4.00E-23

AP001605.1 0.004943581 0.057784826 3.547062242 6.72E-24 4.04E-23

AC011472.2 0.118748808 0.401684633 1.758150244 6.77E-24 4.07E-23

CARD11 0.949591299 4.033692469 2.086722476 6.80E-24 4.09E-23

EMB 1.590882528 4.852979378 1.60904342 6.90E-24 4.14E-23

FOXD3 0.038037446 0.008016997 -2.246286513 6.90E-24 4.14E-23

TMEM92-AS1 0.063029065 0.48206275 2.935131793 7.00E-24 4.20E-23

AC091185.1 0.171909205 0.846735722 2.30026496 7.20E-24 4.32E-23

AC019257.1 0.414670791 2.352645847 2.504245828 7.20E-24 4.32E-23

SHISA6 0.834881871 0.265613266 -1.652244879 7.41E-24 4.45E-23

TEN1-CDK3 0.108263607 0.451365744 2.059748567 7.41E-24 4.45E-23

AC010519.1 0.056486838 0.413089523 2.87046782 7.44E-24 4.46E-23

OASL 1.068942564 3.167356973 1.567095137 7.52E-24 4.51E-23

WIF1 0.177173203 0.045889812 -1.948914628 7.76E-24 4.65E-23

AC007336.2 0.006158945 0.208470624 5.08101715 7.90E-24 4.73E-23

CXorf21 0.672212811 2.154950442 1.680664749 7.91E-24 4.73E-23

ASAP1-IT2 0.025077325 0.20773717 3.050303988 8.05E-24 4.81E-23

HHLA2 2.198480475 19.55388748 3.152876831 8.08E-24 4.83E-23

AL022322.1 0.196393332 1.376384687 2.809065894 8.38E-24 5.00E-23

POLR2F 0.014665 0.048278265 1.718996772 8.50E-24 5.07E-23

FCGR2B 0.396042087 1.586283661 2.001925122 8.50E-24 5.07E-23

AC060234.2 0.016992768 0.106796734 2.651874786 8.68E-24 5.17E-23

C2orf92 0.109701749 0.474382284 2.11246361 8.69E-24 5.18E-23

TRPM2-AS 0.039165696 0.192135404 2.294460891 8.75E-24 5.21E-23

AL355870.1 0.0852538 0.019482594 -2.129578389 8.93E-24 5.32E-23

LINC01758 0.528690713 0.045040343 -3.553134276 9.59E-24 5.71E-23

AC136601.1 0.004255947 0.169712434 5.317468147 9.75E-24 5.80E-23

AC018638.5 2.744867347 8.85276631 1.68939191 9.82E-24 5.84E-23

NOTUM 0.303009139 0.086973727 -1.800709753 9.94E-24 5.91E-23

AL160411.2 0.042688889 0.013438283 -1.66751176 1.01E-23 5.98E-23

AL121574.1 0.604693601 0.187214729 -1.691510371 1.01E-23 6.02E-23

HMGN2P47 0.155827437 1.330675826 3.094137966 1.03E-23 6.10E-23

PDGFD 6.480145444 24.03746681 1.891186766 1.07E-23 6.35E-23

ACRV1 0.01781674 0.068534895 1.943605362 1.08E-23 6.43E-23

KLHL31 0.098753783 0.327206487 1.72829343 1.09E-23 6.44E-23

AC092535.5 0.684499673 4.749027837 2.794510456 1.10E-23 6.53E-23

TAS2R5 0.171041228 0.676939957 1.984683757 1.11E-23 6.58E-23

LINC01783 0.33221432 0.070395373 -2.238561758 1.13E-23 6.68E-23

AC079385.2 0.100677387 0.022010787 -2.193457071 1.17E-23 6.90E-23

TXLNB 0.101656461 0.372347149 1.872946406 1.17E-23 6.91E-23

SPDYC 0.117018193 0.028315366 -2.047075777 1.18E-23 6.99E-23

RTL4 0.378157429 0.106653276 -1.826058678 1.18E-23 7.00E-23

TRBV6-5 0.180384492 1.651042643 3.194230164 1.19E-23 7.04E-23

AC011383.1 0.038250756 0.406493653 3.409672654 1.20E-23 7.11E-23

CCDC73 0.030912856 0.105359783 1.769045433 1.22E-23 7.21E-23

HPX 0.048201714 0.775773465 4.008479067 1.25E-23 7.41E-23

CDH19 0.182708721 0.042084363 -2.1181893 1.26E-23 7.43E-23

RPL7AP64 0.078852287 0.382058297 2.276568281 1.26E-23 7.43E-23

AC004080.4 0.257317257 0.064676775 -1.992228555 1.29E-23 7.61E-23

ENHO 0.703398515 0.232181354 -1.59909017 1.33E-23 7.83E-23

AL353719.1 0.294770051 0.086774126 -1.764253112 1.34E-23 7.87E-23

AGRP 0.526783585 0.15123876 -1.800382464 1.34E-23 7.91E-23

LINC01706 0.001690021 0.085149667 5.654887835 1.39E-23 8.18E-23

DRD4 0.160799212 0.651045903 2.01749893 1.40E-23 8.22E-23

LINC00488 0.527088041 0.150949499 -1.80397799 1.44E-23 8.48E-23

GABRA4 0.054580457 0.008072308 -2.757331398 1.53E-23 8.98E-23

LINC02100 0.063519158 0.873416948 3.781406821 1.55E-23 9.06E-23

TRBV7-9 0.379669498 2.827487652 2.896704718 1.59E-23 9.29E-23

CACNG8 0.02523689 0.117275582 2.216296617 1.62E-23 9.50E-23

EN1 0.021873766 0.180476198 3.044535023 1.63E-23 9.54E-23

ARL6IP4 0.273682172 0.854984085 1.643396106 1.66E-23 9.70E-23

AC136475.10 0.280333681 1.361817251 2.280316123 1.68E-23 9.84E-23

PDCD6IPP1 0.254122191 0.08710219 -1.544741457 1.72E-23 1.00E-22

TTLL11-IT1 0.118020165 0.035186553 -1.745937287 1.73E-23 1.01E-22

KIF1C-AS1 0.128572671 0.534126832 2.05459834 1.76E-23 1.03E-22

AC073130.2 0.091287256 0.344391647 1.915564774 1.81E-23 1.06E-22

CD248 8.726147347 29.91070497 1.777245174 1.82E-23 1.06E-22

LPIN3 3.327830181 9.995920641 1.586757633 1.88E-23 1.09E-22

TAS2R20 0.0960179 0.400112361 2.059029916 1.90E-23 1.11E-22

LINC02642 0.021493399 0.150624775 2.808993549 1.92E-23 1.12E-22

AL358334.3 0.012758182 0.163671053 3.681304553 1.99E-23 1.15E-22

KIAA0319 0.051204146 0.378006195 2.884077335 1.99E-23 1.15E-22

AL009179.1 0.022768137 0.113230748 2.314177611 2.01E-23 1.17E-22

TSHR 0.046037775 0.251863624 2.451752769 2.01E-23 1.17E-22

AC132872.4 0.068951152 0.350134175 2.344261325 2.03E-23 1.18E-22

DERL3 0.658600557 2.965654707 2.170874997 2.06E-23 1.20E-22

AC005387.2 0.076183328 0.48807851 2.679566005 2.08E-23 1.21E-22

AC079340.2 0.602395475 0.031522067 -4.256276883 2.10E-23 1.22E-22

COL1A1 16.98836258 114.9473076 2.758353964 2.10E-23 1.22E-22

STRA6 2.406388614 0.153884146 -3.966953138 2.12E-23 1.23E-22

C10orf99 0.340605909 20.61363852 5.919351906 2.12E-23 1.23E-22

KRT32 0.005769785 0.07702279 3.738695826 2.14E-23 1.24E-22

MATN1 0.024744817 0.078655699 1.668424937 2.21E-23 1.28E-22

AC007991.2 0.043205717 1.357644529 4.973739767 2.21E-23 1.28E-22

XCL1 0.196233068 1.000537621 2.350135332 2.24E-23 1.30E-22

CTBP1-AS 0.113337408 0.457503607 2.013159007 2.28E-23 1.32E-22

AL450311.1 0.039974938 0.496412706 3.634372335 2.31E-23 1.33E-22

NLRP10 0.020343245 0.006485601 -1.649237534 2.38E-23 1.38E-22

EGLN3-AS1 0.010421138 0.280675426 4.751317974 2.40E-23 1.39E-22

AC004862.1 0.018461933 0.003610916 -2.354116646 2.45E-23 1.41E-22

DPT 10.11693416 2.111837764 -2.260201248 2.48E-23 1.43E-22

AL161908.1 0.087331412 0.024564567 -1.829921851 2.50E-23 1.44E-22

LMX1B 1.775058821 0.10747216 -4.045831939 2.51E-23 1.45E-22

IL2RG 3.815911381 13.90997645 1.866020405 2.53E-23 1.46E-22

TLR8 0.398471467 1.794289803 2.170864597 2.55E-23 1.47E-22

CD36 3.561980571 16.4489814 2.207246696 2.55E-23 1.47E-22

AC003070.1 0.283998638 1.390484124 2.291631357 2.55E-23 1.47E-22

LILRP2 0.003749909 0.048267712 3.686130821 2.56E-23 1.47E-22

AC020917.4 0.249400595 1.092883681 2.131603048 2.57E-23 1.48E-22

GLIPR1 1.906767725 5.421736673 1.507625934 2.59E-23 1.49E-22

PCDHB9 0.37329261 1.120707173 1.586030515 2.59E-23 1.49E-22

PLXNC1 1.1945283 3.515213769 1.557171392 2.64E-23 1.52E-22

ADAM20P1 0.027850614 0.141913187 2.34922763 2.64E-23 1.52E-22

AC111000.5 0.102159456 0.651808054 2.673624429 2.65E-23 1.52E-22

ARAP1-AS2 0.034522458 0.259971451 2.912746129 2.66E-23 1.53E-22

AC011498.7 0.145778768 0.595105995 2.02936604 2.74E-23 1.57E-22

AC016405.3 0.288723805 1.655008079 2.519076294 2.74E-23 1.57E-22

P2RX3 0.011591731 0.089828083 2.954070503 2.75E-23 1.57E-22

C1orf87 0.09050804 0.013631441 -2.731107934 2.80E-23 1.60E-22

VDR 10.8537089 3.359952172 -1.691675519 2.80E-23 1.60E-22

PRKCQ-AS1 2.779532613 0.917097311 -1.599695582 2.80E-23 1.60E-22

PRR18 0.115085945 0.035709352 -1.688337781 2.81E-23 1.61E-22

CRYAA 0.203056779 0.030006346 -2.758543634 2.83E-23 1.62E-22

H2AC13 0.129501403 1.287822075 3.313893652 2.88E-23 1.64E-22

ANGPTL3 11.02284145 3.314820816 -1.733493375 2.96E-23 1.69E-22

NPIPB5 0.066270527 0.511438065 2.948120246 3.01E-23 1.72E-22

MYC 12.39717535 35.53526388 1.519239967 3.09E-23 1.76E-22

OR10Q1 0.001752835 0.111613804 5.99268157 3.13E-23 1.78E-22

BX539320.1 0.200132513 0.698220653 1.802727466 3.18E-23 1.81E-22

AC079341.1 0.001237096 0.056233848 5.506409577 3.19E-23 1.81E-22

DPEP1 84.10286769 4.305901897 -4.287767638 3.23E-23 1.84E-22

MSH5 0.160756603 0.771716197 2.263192391 3.27E-23 1.86E-22

DMP1 0.005290149 0.113648965 4.42513249 3.31E-23 1.88E-22

AC079313.1 0.050808629 0.403234257 2.988472755 3.32E-23 1.89E-22

AC010280.1 0.094024702 0.020718877 -2.182094008 3.41E-23 1.94E-22

CHST15 5.040470694 16.10054749 1.675479379 3.49E-23 1.98E-22

AC009533.1 0.258111078 1.297927696 2.330146049 3.49E-23 1.98E-22

AC099568.1 0.018430652 0.168238281 3.190327009 3.59E-23 2.04E-22

AL139352.1 0.037076113 0.204496561 2.463514655 3.59E-23 2.04E-22

ANKK1 0.062672516 0.292614868 2.22309826 3.62E-23 2.05E-22

AL390760.1 0.477635591 0.076930782 -2.634277458 3.64E-23 2.06E-22

DEFB127 0.053798308 0.018603025 -1.532023581 3.69E-23 2.09E-22

PAQR9 0.007253609 0.08971684 3.628607926 3.70E-23 2.09E-22

C5orf64-AS1 0.092498313 0.027080053 -1.772196473 3.75E-23 2.12E-22

NT5C1A 0.516031631 0.029656896 -4.121020002 3.76E-23 2.13E-22

RDM1 0.048193351 0.195816958 2.02259969 3.76E-23 2.13E-22

AP006545.1 0.0319304 0.147569388 2.208390953 3.81E-23 2.15E-22

AL356652.1 0.030681877 0.219996928 2.842024726 3.82E-23 2.16E-22

LSMEM1 0.239030119 0.881811679 1.883278166 3.83E-23 2.16E-22

GBX2 0.005580884 0.053542097 3.262108152 3.94E-23 2.23E-22

AL392089.1 0.291313706 0.062832279 -2.212995758 3.96E-23 2.24E-22

AC138207.1 0.030829903 0.194120522 2.654550365 3.96E-23 2.24E-22

IGFLR1 0.362848168 1.149624678 1.663725047 4.00E-23 2.26E-22

AL591501.1 0.050717138 0.014319651 -1.824476996 4.02E-23 2.27E-22

RGS18 0.687485483 2.176571798 1.662656455 4.05E-23 2.29E-22

CCDC84 0.964298764 3.305318058 1.777237 4.14E-23 2.33E-22

AC243829.2 0.004642846 0.136299913 4.875631372 4.17E-23 2.35E-22

LCAT 1.370049468 4.805940794 1.81059089 4.20E-23 2.36E-22

PSD2 0.051766686 0.282810553 2.449740076 4.20E-23 2.36E-22

OBI1-AS1 0.007575735 0.027974505 1.884654844 4.26E-23 2.40E-22

NOTCH4 4.496523375 13.7228948 1.609702972 4.29E-23 2.41E-22

DLGAP1 0.44741331 0.133852322 -1.740966004 4.32E-23 2.43E-22

VWCE 0.467453959 3.153475373 2.754046485 4.32E-23 2.43E-22

PPIAP78 0.141495508 0.03798933 -1.897090085 4.37E-23 2.46E-22

KLRA1P 0.310575245 1.112915896 1.841329826 4.38E-23 2.46E-22

ELL2P1 0.04325205 0.13198181 1.609498681 4.50E-23 2.53E-22

TRAC 4.743952097 20.71046861 2.12619885 4.54E-23 2.55E-22

TOMM20P2 0.141794125 0.675107046 2.251318518 4.55E-23 2.56E-22

P2RY13 1.04058252 3.482637241 1.742788828 4.61E-23 2.59E-22

TLK2P2 0.019142912 0.13277853 2.794139636 4.63E-23 2.60E-22

TOMM40P1 0.015540303 0.1446604 3.218583485 4.77E-23 2.67E-22

TNKS2-AS1 0.964664955 0.316663156 -1.607078936 5.05E-23 2.83E-22

C1orf61 0.003378604 0.023616695 2.805307998 5.11E-23 2.86E-22

LINC01843 0.734497359 2.935287205 1.998672463 5.23E-23 2.93E-22

TRBV9 0.190927867 1.639053492 3.10176335 5.33E-23 2.98E-22

AC091887.1 0.039846947 0.239376263 2.586739002 5.46E-23 3.05E-22

AP001636.2 0.01518488 0.112812212 2.893215878 5.57E-23 3.11E-22

ZBTB32 0.074897747 0.271962501 1.860413514 5.70E-23 3.18E-22

IL9R 0.034792876 0.152810758 2.134882284 5.73E-23 3.20E-22

MIR320E 0.214871935 1.545851342 2.846852624 5.78E-23 3.22E-22

KCNV2 0.013951766 0.081424724 2.545019222 5.80E-23 3.23E-22

AC110015.1 0.044357726 0.27691196 2.642170049 5.82E-23 3.24E-22

AC090164.2 0.014076668 0.261995536 4.218164453 5.84E-23 3.25E-22

TDGF1P3 0.199155348 0.033740612 -2.561336165 5.85E-23 3.26E-22

AC011481.2 0.173586929 0.497653643 1.519483684 5.88E-23 3.27E-22

C9orf153 0.035970928 0.133036979 1.886924043 5.90E-23 3.29E-22

C16orf74 0.501583044 3.99209426 2.9925853 6.07E-23 3.38E-22

AC015743.1 0.006645737 0.117642071 4.145831148 6.10E-23 3.39E-22

AC126564.1 0.191084928 0.03388127 -2.495654127 6.23E-23 3.46E-22

CDH18 0.129648228 0.030017628 -2.110720608 6.24E-23 3.47E-22

RN7SL834P 0.239019111 1.24867322 2.385198092 6.38E-23 3.54E-22

LINC01991 0.128539162 0.005491379 -4.548895689 6.39E-23 3.54E-22

ADAMTS4 2.251445369 8.919761138 1.986153605 6.43E-23 3.56E-22

MAFA 0.197988124 0.046057353 -2.103910506 6.53E-23 3.62E-22

AL358075.2 0.238604115 0.083359726 -1.517196475 6.62E-23 3.67E-22

AC012435.2 0.01926713 0.114622137 2.5726721 6.66E-23 3.69E-22

AC109460.3 0.038774118 0.19753044 2.348909106 6.75E-23 3.74E-22

LINC01934 0.028407072 0.209886415 2.885286748 6.79E-23 3.76E-22

LINC02473 0.008233233 0.18804417 4.513468775 6.81E-23 3.77E-22

KLRB1 1.471052933 4.314373181 1.552301811 7.10E-23 3.92E-22

AL355482.2 0.033043123 0.007061857 -2.226230527 7.19E-23 3.97E-22

LINC01638 0.060197012 1.138491507 4.241287833 7.30E-23 4.03E-22

AC012615.6 0.180508467 0.822616013 2.188152643 7.62E-23 4.19E-22

AC114284.1 0.059152957 0.226879308 1.939402838 7.63E-23 4.20E-22

LINC01163 0.002357929 0.038163479 4.016600522 7.64E-23 4.21E-22

CPNE5 0.734589456 2.583561551 1.814351162 7.72E-23 4.25E-22

AC124944.2 0.175001827 1.050264643 2.58531101 7.89E-23 4.34E-22

IFT74-AS1 0.164566632 0.048271641 -1.769424078 8.16E-23 4.48E-22

GUCA2B 0.057989515 3.516992214 5.922406262 8.30E-23 4.55E-22

WDFY4 0.376538008 1.32030667 1.810005663 8.41E-23 4.61E-22

AP000577.1 0.197600098 1.249658111 2.660877881 8.54E-23 4.68E-22

AC004832.5 0.212312167 0.727070708 1.775908626 9.21E-23 5.05E-22

BNIPL 0.829635176 0.283248702 -1.55040772 9.41E-23 5.15E-22

LCN1 0.004005765 0.39145799 6.610636044 9.49E-23 5.19E-22

AL450322.1 0.008485966 0.177871181 4.389610033 9.85E-23 5.39E-22

ARHGAP27P1-BPTFP1-KPNA2P3 0.31731414 1.367695025 2.107762852 9.89E-23 5.40E-22

FGFBP2 0.560145814 2.112985156 1.915408297 1.00E-22 5.48E-22

RAG2 0.125957667 0.009126786 -3.786688158 1.01E-22 5.49E-22

ZNF33CP 0.003411242 0.054260062 3.991521736 1.02E-22 5.57E-22

AC068473.4 0.280388492 0.041327947 -2.762237521 1.03E-22 5.64E-22

ASGR1 0.176208928 0.524056897 1.572436434 1.07E-22 5.82E-22

KIRREL3-AS1 0.067917175 0.008409924 -3.013611754 1.09E-22 5.93E-22

CD200R1 0.227560597 1.144087666 2.329874925 1.09E-22 5.94E-22

AC009090.4 0.03120789 0.202180095 2.695658263 1.10E-22 5.98E-22

PLA2G4C-AS1 0.027952661 0.168287209 2.589868006 1.11E-22 6.05E-22

AL031275.1 0.91051505 0.235134916 -1.953194075 1.13E-22 6.16E-22

CARD9 0.263058502 0.747432322 1.506559276 1.14E-22 6.19E-22

MUC12-AS1 0.233053782 1.497607159 2.683924406 1.19E-22 6.44E-22

SLC17A2 0.179394519 1.838151896 3.35704827 1.19E-22 6.48E-22

AC093583.1 7.450630268 2.086020688 -1.836609006 1.21E-22 6.57E-22

AL122125.1 0.05372958 0.398730479 2.891625425 1.22E-22 6.62E-22

C3orf70 0.725644515 2.121469403 1.547729004 1.22E-22 6.62E-22

TRBV19 0.319424665 2.130600632 2.73771257 1.23E-22 6.65E-22

CTSLP6 0.035059602 0.005767021 -2.603911475 1.23E-22 6.67E-22

PRSS44P 0.111539594 0.03875992 -1.524918447 1.26E-22 6.83E-22

SVEP1 3.704366904 1.228410829 -1.592433864 1.30E-22 7.04E-22

MARCHF1 0.712775113 2.191787105 1.620588802 1.33E-22 7.18E-22

AC132812.1 1.430879772 5.73534493 2.002977797 1.44E-22 7.74E-22

AC009185.1 0.869134477 0.265513657 -1.710793345 1.49E-22 8.00E-22

AL499616.1 0.009269536 0.072647339 2.970340876 1.49E-22 8.02E-22

AC011498.6 0.140205536 0.650710007 2.214471425 1.51E-22 8.11E-22

LINC01614 0.107100236 0.735019609 2.778821081 1.51E-22 8.15E-22

SLFNL1-AS1 0.079033619 0.361954744 2.195270948 1.53E-22 8.23E-22

CDH6 7.953147674 27.66590364 1.798511183 1.55E-22 8.34E-22

THEMIS 0.240537162 1.168348983 2.280139563 1.55E-22 8.34E-22

HLA-DPA3 0.023220086 0.31058697 3.741552063 1.57E-22 8.45E-22

IL1RL1 8.227516492 2.075214184 -1.987196768 1.62E-22 8.69E-22

AC136632.1 1.624702597 5.77391277 1.829373656 1.63E-22 8.75E-22

AP002812.5 0.068616694 0.360178985 2.39208249 1.68E-22 9.04E-22

AC116914.2 0.236940207 1.374503062 2.536315183 1.69E-22 9.07E-22

CDK3 0.06523874 0.357515974 2.454206888 1.70E-22 9.11E-22

LRRC75B 4.068962664 11.58559773 1.509599531 1.71E-22 9.17E-22

SLC16A6 0.550368194 2.03508022 1.886616661 1.75E-22 9.36E-22

GAPDHP70 0.053006785 0.180589934 1.768468536 1.77E-22 9.48E-22

RPL22P19 0.024782544 0.30236259 3.608883429 1.79E-22 9.56E-22

AL117382.3 0.182445902 0.019238457 -3.245403722 1.80E-22 9.66E-22

AC005840.2 0.190084825 0.793156273 2.060961783 1.82E-22 9.75E-22

LINC02528 0.014541152 0.282896719 4.28206202 1.82E-22 9.76E-22

LINC01874 6.198979607 0.912968108 -2.763394389 1.83E-22 9.82E-22

SMG1P7 0.11726164 0.488570014 2.058834181 1.85E-22 9.88E-22

AC005821.1 0.092375383 0.028108721 -1.716490605 1.87E-22 1.00E-21

AL031009.1 0.048721938 0.211836501 2.120307767 1.93E-22 1.03E-21

CCR10 0.194860055 0.623353335 1.677611777 1.94E-22 1.04E-21

AC136604.2 0.075976137 0.364630913 2.262818607 1.94E-22 1.04E-21

RPS28P5 0.199805525 0.709832488 1.828882132 1.96E-22 1.04E-21

KIR3DL1 0.02344261 0.108910368 2.215936171 1.97E-22 1.05E-21

STEAP1B 0.063848706 0.297272238 2.219055453 2.01E-22 1.07E-21

PLAC8 0.304165923 1.103140788 1.858686492 2.02E-22 1.08E-21

AC128707.1 0.354299595 0.047489151 -2.89929994 2.07E-22 1.10E-21

PLCG1-AS1 0.094879833 0.335665901 1.822852613 2.19E-22 1.17E-21

AC021146.12 0.643607603 0.184672742 -1.801210432 2.27E-22 1.21E-21

KCNE1 0.222098208 0.068252066 -1.702253118 2.33E-22 1.24E-21

AC106799.1 0.218272604 0.068965215 -1.662190277 2.44E-22 1.30E-21

AC021443.1 0.714956115 3.163806818 2.145734916 2.55E-22 1.35E-21

AL031733.2 0.019916068 0.13384023 2.748507111 2.56E-22 1.36E-21

SMPD5 0.083065513 0.236910553 1.512020932 2.63E-22 1.39E-21

AC087379.1 0.097847693 0.025521702 -1.938813301 2.69E-22 1.43E-21

LINC02406 0.106066092 0.028265009 -1.907874445 2.71E-22 1.43E-21

LINC00926 0.143547305 0.576711203 2.006322805 2.77E-22 1.47E-21

RGS13 0.02239441 0.083573101 1.899899978 2.79E-22 1.47E-21

CPA1 0.211671597 0.031660353 -2.741078455 2.85E-22 1.51E-21

CYP2D8P 0.034565439 0.249633869 2.852411511 2.87E-22 1.52E-21

GLYATL1P1 0.030605593 0.126917194 2.052020327 2.99E-22 1.58E-21

ADD2 0.053915945 0.35032464 2.699908555 2.99E-22 1.58E-21

SLCO2B1 3.712007903 11.62793469 1.647323186 3.01E-22 1.59E-21

AC009159.4 0.012473685 0.101212713 3.020430886 3.02E-22 1.59E-21

KCNH4 0.040225 0.126611913 1.654248816 3.03E-22 1.60E-21

LRRC66 0.203381476 0.811747103 1.996842043 3.03E-22 1.60E-21

AC004923.4 0.157851053 0.649031048 2.039723608 3.03E-22 1.60E-21

AC068299.1 0.179007379 1.91669832 3.420532318 3.04E-22 1.60E-21

CYP21A2 0.115831797 0.658639805 2.507458363 3.12E-22 1.64E-21

UGT1A3 0.008093478 0.12170626 3.910499757 3.13E-22 1.65E-21

CYP51A1P2 0.061836636 0.237276212 1.940033724 3.17E-22 1.67E-21

LAMC2 9.558824882 3.190328548 -1.583128267 3.19E-22 1.68E-21

E2F3P1 0.016749896 0.154437875 3.204802563 3.25E-22 1.71E-21

CCR8 0.036456592 0.258426905 2.825504683 3.29E-22 1.73E-21

SUCNR1 17.03261574 4.083018258 -2.060592086 3.30E-22 1.73E-21

AL162430.2 0.083907732 0.253796347 1.596795646 3.30E-22 1.73E-21

AL157871.6 0.195362242 0.06079279 -1.684179531 3.35E-22 1.76E-21

KDM2B-DT 0.091010783 0.32453466 1.834263173 3.36E-22 1.76E-21

LENG8-AS1 1.052020947 3.302748556 1.650503708 3.37E-22 1.77E-21

NRARP 2.5812195 8.156627695 1.659919973 3.44E-22 1.80E-21

C3orf22 0.004764819 0.040318889 3.080962564 3.61E-22 1.89E-21

AL137073.1 0.031586004 0.436606874 3.788977487 3.63E-22 1.90E-21

AC092376.2 0.173537563 0.496127797 1.515463819 3.64E-22 1.90E-21

IL5RA 0.139697618 0.047803512 -1.547118911 3.68E-22 1.93E-21

FTLP15 0.033773816 0.646487475 4.258645338 3.82E-22 2.00E-21

CLEC12A-AS1 0.003970516 0.032215639 3.020362939 3.83E-22 2.00E-21

FYB1 2.609953703 8.63751124 1.726591467 3.92E-22 2.05E-21

C19orf84 0.040405157 0.156530863 1.953835783 3.94E-22 2.05E-21

KCNT1 0.006269 0.033542945 2.419702071 3.94E-22 2.06E-21

SLC27A6 0.034964929 0.00798464 -2.130609286 3.98E-22 2.07E-21

CNGA3 0.041258642 0.013890012 -1.570648513 3.98E-22 2.08E-21

PAQR6 0.238329471 1.478289197 2.632899262 4.06E-22 2.12E-21

AC008163.1 0.009639511 0.192192964 4.317451683 4.20E-22 2.19E-21

AL158834.2 0.063918798 0.280866533 2.135572532 4.32E-22 2.25E-21

ZC3H12D 0.172030263 0.535988113 1.63953862 4.32E-22 2.25E-21

AL592114.3 0.831015308 0.287067181 -1.53348665 4.35E-22 2.26E-21

GATA5 0.860232943 0.201383656 -2.094780781 4.35E-22 2.26E-21

NPIPB13 0.006329718 0.056324871 3.153558913 4.37E-22 2.27E-21

ZNF33BP1 0.015633192 0.161760395 3.371174152 4.54E-22 2.35E-21

LINC02664 0.011879925 0.10754798 3.178382756 4.55E-22 2.36E-21

DLX6-AS1 0.012291302 0.047942366 1.963663409 4.60E-22 2.39E-21

AKAP4 0.035115256 0.007057201 -2.314929928 4.70E-22 2.43E-21

DAND5 0.023520241 0.196057432 3.059301589 4.70E-22 2.44E-21

SCN4B 1.765081297 6.988504366 1.985249099 4.80E-22 2.48E-21

F2RL3 1.866981751 9.903045003 2.407164369 4.80E-22 2.48E-21

MIR670HG 0.024846309 0.007434586 -1.740707345 4.85E-22 2.51E-21

FAM72D 0.02115616 0.074304628 1.812374304 5.01E-22 2.59E-21

AC008870.2 0.14045925 0.60869582 2.11556982 5.25E-22 2.71E-21

CHST13 1.531017755 6.216933418 2.021712114 5.29E-22 2.73E-21

AL359397.1 1.086088829 0.342991112 -1.662899008 5.40E-22 2.78E-21

AC073130.1 0.005885483 0.045787857 2.95973237 5.42E-22 2.79E-21

AC004253.1 0.148081443 0.831291728 2.488963995 5.64E-22 2.91E-21

CKAP2LP1 0.002242024 0.045642889 4.347516235 5.82E-22 3.00E-21

AC068790.5 0.149478973 0.657821977 2.137754652 5.98E-22 3.08E-21

NPIPA1 0.169593602 0.57378574 1.758430366 6.12E-22 3.14E-21

AC027373.1 0.042779997 0.209999219 2.295375676 6.25E-22 3.21E-21

LY6H 0.117695956 1.230300631 3.385874235 6.33E-22 3.25E-21

AC093520.2 0.007361274 0.251314751 5.09339615 6.36E-22 3.26E-21

ATP5MC1P5 0.068176079 0.013014745 -2.389118601 6.37E-22 3.27E-21

APBB3 1.563731728 4.423714624 1.500265293 6.37E-22 3.27E-21

TRBJ2-1 0.223418438 1.614231899 2.853027696 6.50E-22 3.34E-21

BNIP3P1 0.110459855 0.321717745 1.542273375 6.74E-22 3.45E-21

AC141557.2 0.034163917 0.358754161 3.392450264 6.74E-22 3.45E-21

FN1 32.84256128 113.2576303 1.785969702 6.78E-22 3.47E-21

SNORD104 1.413838704 8.331461407 2.558952037 7.17E-22 3.67E-21

AC092667.1 0.042215338 0.264775802 2.648932107 7.30E-22 3.73E-21

TIFAB 0.025143763 0.141667383 2.494235148 7.31E-22 3.74E-21

CARD17 0.023547889 0.194493077 3.046049136 7.32E-22 3.74E-21

KRTAP5-10 0.023261024 0.153066646 2.718173434 7.33E-22 3.75E-21

LINC01736 0.061913901 0.420851079 2.764974551 7.35E-22 3.75E-21

AF241728.1 0.215968328 0.056896583 -1.92440584 7.66E-22 3.91E-21

LRRC39 0.199670994 0.867277457 2.118868844 7.73E-22 3.95E-21

AP001893.3 0.012141949 0.129646589 3.416512355 7.85E-22 4.00E-21

LINC01732 0.003715158 0.081156316 4.449207769 8.13E-22 4.14E-21

VCAM1 29.0877245 103.6318576 1.832985227 8.23E-22 4.19E-21

AC091390.4 0.351979311 1.401581811 1.993493422 8.23E-22 4.19E-21

HMGN1P13 0.034014298 0.633976739 4.220216704 8.30E-22 4.23E-21

AC009061.1 0.181685307 0.060543797 -1.585390683 8.43E-22 4.29E-21

AC137932.3 0.086061301 0.38460449 2.159939047 8.46E-22 4.30E-21

LY6G5B 0.177001851 0.967895761 2.451087234 8.46E-22 4.30E-21

TRBV2 0.182627847 1.268133092 2.7957275 8.58E-22 4.37E-21

AC079584.2 0.008976606 0.116171966 3.693948131 8.69E-22 4.42E-21

PRC1-AS1 0.026583376 0.115714817 2.121977363 8.73E-22 4.44E-21

SEMA4A 9.113818778 3.051167014 -1.578694532 9.06E-22 4.60E-21

RHEX 4.348334465 17.28271285 1.990794874 9.06E-22 4.60E-21

AL121987.2 0.068106159 0.234777903 1.785439457 9.19E-22 4.66E-21

PCDHGC4 0.028101463 0.105953651 1.914716164 9.19E-22 4.66E-21

ISX 0.059164528 0.010020919 -2.561717653 9.23E-22 4.68E-21

GPA33 0.034885763 0.117138353 1.747503209 9.25E-22 4.69E-21

STX1B 0.202938824 0.712610634 1.812069123 9.25E-22 4.69E-21

AL133406.2 0.080592015 0.300502303 1.898667235 9.32E-22 4.72E-21

ACTG1P3 0.111938717 0.320383975 1.517092878 9.44E-22 4.78E-21

CCL4L2 1.021498395 6.129015295 2.584968368 9.51E-22 4.81E-21

EFCAB3 0.003116055 0.045447665 3.866413563 9.52E-22 4.82E-21

CR936218.1 0.259280711 1.093931576 2.076935713 9.57E-22 4.84E-21

AL590762.1 0.059879243 0.408805494 2.771286705 9.71E-22 4.91E-21

AC013553.3 0.125077088 0.492833906 1.978283979 9.77E-22 4.94E-21

PRG2 0.007741326 0.063593605 3.038229018 9.85E-22 4.98E-21

AL589743.1 0.011218543 0.063542217 2.501830152 9.87E-22 4.99E-21

WEE2 0.157457096 0.053873356 -1.547314939 9.97E-22 5.03E-21

EGILA 0.040205158 0.207802301 2.369759116 1.00E-21 5.06E-21

LINC02633 0.121459474 0.013878897 -3.129510231 1.00E-21 5.06E-21

TRBV4-1 0.123017573 1.354294019 3.460604661 1.01E-21 5.09E-21

ETNK2 20.55196175 6.529046657 -1.654331854 1.01E-21 5.10E-21

AC245884.8 0.222197161 1.305749995 2.554966411 1.03E-21 5.21E-21

AC092325.1 1.648129599 0.056590993 -4.864113425 1.03E-21 5.21E-21

AC087482.1 3.536526086 14.45827188 2.031490317 1.04E-21 5.26E-21

LINC02036 0.082439276 0.476345565 2.530604813 1.06E-21 5.31E-21

ATP11A 8.018090472 23.31906001 1.540179033 1.06E-21 5.34E-21

AL512770.1 0.086839521 0.374746255 2.109490393 1.06E-21 5.34E-21

AC044781.1 0.009184703 0.226240156 4.622478132 1.06E-21 5.35E-21

FAM242F 0.046498768 0.014127809 -1.718654746 1.09E-21 5.48E-21

AL450313.1 0.469125864 0.006756481 -6.1175593 1.11E-21 5.56E-21

RAB44 0.019063597 0.063946278 1.746040012 1.11E-21 5.56E-21

TSPOAP1-AS1 0.08763831 0.261557493 1.577494536 1.14E-21 5.71E-21

AC079331.2 0.022072828 0.157466132 2.834698198 1.14E-21 5.71E-21

U52111.1 0.035438985 0.178923446 2.335933246 1.15E-21 5.76E-21

AC090004.1 0.075303686 0.308365584 2.033849373 1.16E-21 5.83E-21

CYP3A5 1.208858242 5.412248331 2.162582961 1.17E-21 5.87E-21

KCNIP2 0.178899345 0.613359249 1.777584213 1.18E-21 5.91E-21

TRAV21 0.168999613 1.258429278 2.896532294 1.19E-21 5.96E-21

AL645939.5 0.05451231 0.512490354 3.232870882 1.20E-21 6.00E-21

CRYM-AS1 0.040342502 0.148884109 1.88381731 1.21E-21 6.07E-21

LIM2 0.006308094 0.066605654 3.40036849 1.21E-21 6.07E-21

CHIT1 0.077507872 2.431162175 4.971159477 1.23E-21 6.15E-21

IBSP 0.021935604 0.827301317 5.237066521 1.23E-21 6.15E-21

FUT7 0.054989345 0.192083204 1.80450738 1.27E-21 6.35E-21

AC104070.1 0.041801953 0.761102258 4.186448036 1.30E-21 6.51E-21

IGLON5 0.448483657 2.641338327 2.55814179 1.32E-21 6.61E-21

SERPINB9P1 0.63220218 2.841475856 2.168182542 1.36E-21 6.80E-21

LINC02641 0.011331097 0.097892487 3.110910586 1.37E-21 6.87E-21

AC132872.3 0.365955634 2.019289163 2.464106857 1.40E-21 6.98E-21

AC007750.1 0.007208222 0.046339931 2.684540513 1.43E-21 7.15E-21

AC009090.5 0.015064752 0.096036274 2.672402485 1.45E-21 7.23E-21

KCTD19 0.014784044 0.084905949 2.521824716 1.45E-21 7.24E-21

LINC00664 0.022162288 0.139424946 2.653309961 1.45E-21 7.24E-21

AC136601.2 0.00190124 0.213582761 6.811710843 1.46E-21 7.28E-21

RPL32P1 0.102028774 0.723311807 2.825641621 1.46E-21 7.29E-21

TRAV4 0.155301446 1.013204457 2.705782163 1.48E-21 7.37E-21

TRBV4-2 0.117070998 1.417207799 3.59759569 1.48E-21 7.37E-21

BPHL 10.42599381 3.653792286 -1.512718382 1.49E-21 7.41E-21

LINC00707 0.160311446 1.100786842 2.779585792 1.53E-21 7.61E-21

AL731571.1 0.207636123 0.638208813 1.619971076 1.56E-21 7.76E-21

AC133961.1 0.01253894 0.086777616 2.790907513 1.56E-21 7.79E-21

NKAIN3-IT1 0.089689619 0.026139562 -1.778706041 1.57E-21 7.81E-21

AC009237.8 2.240744128 0.703462564 -1.67143236 1.64E-21 8.17E-21

FAM43B 1.165653957 0.373494866 -1.641979246 1.72E-21 8.53E-21

CDH13 3.096009276 9.338292442 1.592748975 1.73E-21 8.58E-21

AL353801.2 0.327162795 0.103045972 -1.666720588 1.77E-21 8.78E-21

KLK5 0.563448881 0.050996355 -3.465818683 1.77E-21 8.79E-21

AC087222.1 0.103054198 0.659191437 2.677294231 1.79E-21 8.90E-21

SLC25A47 0.297222548 0.057592607 -2.367588043 1.81E-21 8.96E-21

PI16 2.674226382 0.563014786 -2.247876883 1.82E-21 9.03E-21

C1RL-AS1 1.378691882 4.238105339 1.620119375 1.84E-21 9.11E-21

AC010729.2 0.000142987 0.05600825 8.613613525 1.84E-21 9.12E-21

RNU6-956P 0.137739364 0.037599665 -1.873149202 1.90E-21 9.40E-21

AL158163.1 0.162386515 0.537603478 1.727110637 1.97E-21 9.73E-21

KIF19 0.048058923 0.172149479 1.840785589 1.98E-21 9.79E-21

AC079322.1 0.100086147 0.429485207 2.101366135 2.01E-21 9.92E-21

TCAF2P1 0.02418008 0.082405771 1.768926336 2.09E-21 1.03E-20

C7orf61 0.071046797 0.265264539 1.90059032 2.11E-21 1.04E-20

AL139246.2 0.044376466 0.36833398 3.053147798 2.11E-21 1.04E-20

AC105020.5 0.289827802 1.055407806 1.864532659 2.15E-21 1.06E-20

MYO15B 3.354788324 10.8815231 1.697586862 2.18E-21 1.07E-20

AL606537.1 0.025678066 0.00538629 -2.253172804 2.19E-21 1.08E-20

TNFRSF14-AS1 0.585105108 1.91843143 1.713159481 2.19E-21 1.08E-20

LAX1 0.220904992 0.937900384 2.086008679 2.19E-21 1.08E-20

AC093520.1 0.004167721 0.059155691 3.827186427 2.21E-21 1.09E-20

GPR84 0.148641791 0.633879364 2.092368515 2.24E-21 1.10E-20

BX293995.1 0.001821509 0.287108271 7.300317268 2.26E-21 1.11E-20

CIITA 1.230969225 3.498989709 1.507143727 2.29E-21 1.12E-20

AC125494.1 0.026716775 0.106080234 1.989338089 2.32E-21 1.14E-20

MIR223HG 0.246558054 0.945240897 1.938754671 2.37E-21 1.16E-20

ZNF804B 0.314120073 0.049109129 -2.677252984 2.41E-21 1.18E-20

PLAUR 1.890214832 5.432327378 1.523020213 2.41E-21 1.19E-20

CD5L 0.038950162 1.215745921 4.964068607 2.45E-21 1.20E-20

NANOS1 1.425184593 0.462028822 -1.625094035 2.46E-21 1.21E-20

NEU4 0.574721458 0.186410772 -1.624377687 2.46E-21 1.21E-20

AC093690.1 0.061226114 0.261658656 2.095466971 2.48E-21 1.22E-20

ARID3C 0.034184743 0.169419516 2.309175595 2.49E-21 1.22E-20

TEX11 0.167128398 1.922829313 3.524201906 2.49E-21 1.22E-20

TUBA3E 0.265976945 1.287764595 2.275495789 2.54E-21 1.25E-20

GABRG3 0.059358044 0.010731771 -2.467555376 2.57E-21 1.26E-20

LINC01141 0.015422602 0.08360436 2.438532015 2.58E-21 1.26E-20

DCST2 0.063241944 0.263138779 2.056870242 2.58E-21 1.27E-20

AC007879.3 0.034327104 0.142751294 2.05608375 2.59E-21 1.27E-20

FBXO43 0.042628835 0.145577564 1.771886482 2.60E-21 1.27E-20

IL9RP3 0.01837799 0.095818329 2.382322657 2.61E-21 1.28E-20

ADAMTSL4-AS2 0.133109945 0.801461282 2.590014468 2.64E-21 1.29E-20

GPR55 0.058124977 0.210811517 1.858723544 2.64E-21 1.29E-20

RUBCNL 0.258648188 0.74376568 1.523857094 2.66E-21 1.30E-20

AC125603.4 0.004853458 0.072076279 3.892439648 2.72E-21 1.33E-20

MYCN 1.312991738 0.371646926 -1.820853259 2.79E-21 1.36E-20

AC012593.1 0.007397281 0.100663479 3.766401434 2.82E-21 1.38E-20

HLA-L 0.832115805 2.433738542 1.548317961 2.84E-21 1.39E-20

LINC01176 0.548287228 2.119976966 1.951044818 2.84E-21 1.39E-20

TRBV5-1 0.240400225 1.300121923 2.43513677 2.86E-21 1.39E-20

AC099066.2 0.012649188 0.120875922 3.256410193 2.86E-21 1.40E-20

CASP1P2 0.011449801 0.119858372 3.387936196 2.87E-21 1.40E-20

AC093281.2 0.004856362 0.236493717 5.605782093 2.88E-21 1.40E-20

DIO1 26.5740953 1.008427804 -4.71984087 2.88E-21 1.40E-20

AL158166.1 0.192879231 0.553920296 1.521980595 2.90E-21 1.41E-20

PROC 5.261353549 1.422607443 -1.886896381 2.92E-21 1.42E-20

TRAV41 0.09234061 0.563372793 2.609052729 2.93E-21 1.43E-20

DNA2 0.293911138 0.835864797 1.50788957 2.94E-21 1.43E-20

AC010205.1 0.468295785 0.142325324 -1.718227674 2.98E-21 1.45E-20

SLC16A8 0.098107919 0.426491983 2.120077126 2.98E-21 1.45E-20

GPR174 0.23297248 1.358328198 2.543600652 3.02E-21 1.47E-20

MIR34AHG 0.087719431 0.276289166 1.655210638 3.29E-21 1.59E-20

ALG13-AS1 0.229273251 1.059664213 2.20846722 3.30E-21 1.60E-20

AC022730.2 0.099320888 0.016602828 -2.580668166 3.31E-21 1.60E-20

RNU6-1161P 1.101683818 0.039846027 -4.789130552 3.32E-21 1.61E-20

AC110995.1 0.194480309 0.650764654 1.742511803 3.35E-21 1.62E-20

AC073610.2 0.0367806 0.12881654 1.808300926 3.36E-21 1.63E-20

AC016027.2 0.008834968 0.115736442 3.711474407 3.43E-21 1.66E-20

KIR2DS4 0.02859278 0.208915468 2.869196535 3.48E-21 1.68E-20

PEPD 84.34766029 23.9005466 -1.819304437 3.68E-21 1.78E-20

Z99289.2 0.011043182 0.118115126 3.418965911 3.69E-21 1.78E-20

WTAPP1 0.104547297 0.032246999 -1.696918963 3.79E-21 1.83E-20

AL450306.1 0.032118003 0.173409744 2.432730868 3.81E-21 1.84E-20

AC112491.1 0.359300245 1.373930144 1.93504683 3.83E-21 1.85E-20

LRRC17 1.137970015 3.561319569 1.645949355 3.91E-21 1.89E-20

AC111170.4 0.033387993 0.214307164 2.682278811 3.95E-21 1.90E-20

LMTK3 1.428098524 0.498654714 -1.51798242 3.99E-21 1.92E-20

AL096865.1 0.068664485 0.399413322 2.540246448 4.09E-21 1.97E-20

MIR3142HG 0.115701526 0.552081466 2.254473278 4.13E-21 1.99E-20

RNU2-11P 0.159922159 1.404027851 3.134129794 4.15E-21 2.00E-20

HS6ST1P1 1.514442026 0.269082608 -2.4926653 4.18E-21 2.01E-20

OR9Q1 0.006241072 0.066016901 3.402969679 4.18E-21 2.01E-20

CLEC4M 0.034087065 0.008633246 -1.981249417 4.29E-21 2.06E-20

LINC00544 0.003957738 0.046647255 3.559044242 4.33E-21 2.08E-20

LHX2 0.008705954 0.252054896 4.855591839 4.41E-21 2.12E-20

AL645929.1 2.079993101 6.34009256 1.607925159 4.48E-21 2.15E-20

PILRB 0.318904555 1.759513662 2.463980105 4.48E-21 2.15E-20

ADSS1 2.430510576 13.95030413 2.520965258 4.48E-21 2.15E-20

AL445430.1 0.047063142 0.006326286 -2.895166998 4.50E-21 2.16E-20

H2AC17 0.086024004 0.588016089 2.773044447 4.55E-21 2.18E-20

AC243829.1 0.027803995 0.295618653 3.410373207 4.57E-21 2.19E-20

AC078850.2 0.835273908 0.283142977 -1.560718628 4.58E-21 2.19E-20

RTP5 0.009853407 0.084855323 3.106310624 4.62E-21 2.21E-20

KC877373.1 0.12917878 0.656768932 2.346016782 4.68E-21 2.24E-20

PROZ 4.27465719 0.243415444 -4.134316116 4.72E-21 2.26E-20

GAPDHP61 0.05022381 0.210613256 2.068152875 4.79E-21 2.29E-20

SP140 0.248055484 0.866994532 1.805360042 4.89E-21 2.34E-20

HLA-DPB2 0.304300758 1.970213627 2.694782234 4.89E-21 2.34E-20

DPEP3 0.021403368 0.106191369 2.31075676 4.93E-21 2.36E-20

SLC47A1P2 0.097415891 0.655742694 2.750900796 5.16E-21 2.46E-20

ATP2A1 0.081020136 0.436369754 2.429198701 5.20E-21 2.48E-20

CEACAM4 0.149628548 0.537953748 1.846096683 5.22E-21 2.49E-20

MRPS6 34.24203228 10.80997837 -1.663404692 5.23E-21 2.49E-20

FBLN7 0.327401933 1.513740814 2.208983461 5.27E-21 2.51E-20

AC068790.3 0.146629954 0.547016098 1.899403435 5.38E-21 2.56E-20

AL353596.1 0.326822931 0.097749404 -1.741349404 5.51E-21 2.62E-20

SPARCL1 54.94232603 168.6541579 1.618077991 5.60E-21 2.66E-20

H3C10 0.456986592 2.772094947 2.600752931 5.64E-21 2.68E-20

AC007834.1 0.081529208 0.02288101 -1.833166265 5.73E-21 2.72E-20

RPS15AP24 0.121618251 0.382536158 1.653236378 5.75E-21 2.73E-20

TRBV29-1 0.375992816 2.167340172 2.527148604 5.79E-21 2.75E-20

LINC02481 0.106715727 0.303743815 1.509082229 5.79E-21 2.75E-20

AL592431.1 0.028590686 0.405737472 3.826929395 5.93E-21 2.81E-20

PCDHGB8P 0.024755542 0.107328761 2.11621326 6.03E-21 2.86E-20

AC010463.2 0.027957635 0.460038065 4.040439016 6.05E-21 2.87E-20

BX322650.1 0.028353828 0.192806207 2.765536066 6.06E-21 2.88E-20

LINC02487 0.073158506 0.242901513 1.731273964 6.11E-21 2.90E-20

AC131097.2 0.295919689 0.09059168 -1.707755234 6.22E-21 2.95E-20

AC005332.1 0.054997057 0.306993464 2.480781619 6.54E-21 3.09E-20

DPY19L2P2 0.567186582 1.911702269 1.752962546 6.54E-21 3.09E-20

CSPG4P13 0.05881242 0.367797036 2.644717081 6.58E-21 3.11E-20

TRAV17 0.140349866 0.945829806 2.752552917 6.60E-21 3.12E-20

BCL11B 0.334573893 1.048570882 1.648027608 6.76E-21 3.20E-20

KLF2P3 0.011258991 0.002648345 -2.087914245 6.92E-21 3.27E-20

DLEU7 0.087213831 0.292946653 1.748009122 6.95E-21 3.28E-20

NLRP2 1.264827701 0.395416881 -1.677494501 7.00E-21 3.30E-20

C4orf50 0.005899494 0.035301506 2.581066641 7.07E-21 3.34E-20

KCNH1-IT1 0.078173177 0.019691134 -1.989127484 7.14E-21 3.37E-20

LINC02541 0.043188738 0.267704167 2.631912537 7.20E-21 3.39E-20

AC093827.3 0.255533635 0.730213015 1.514804185 7.48E-21 3.53E-20

PLA2G4D 0.005294935 0.115613662 4.448555212 7.56E-21 3.56E-20

TRAV26-1 0.078861069 0.468924188 2.571969524 7.88E-21 3.71E-20

GP6 0.066037686 0.314418408 2.251324202 7.95E-21 3.74E-20

RN7SL209P 0.041212979 0.298990371 2.858928373 8.14E-21 3.83E-20

AP001046.1 0.088206378 0.023778706 -1.891212747 8.17E-21 3.84E-20

ZIC3 0.0150609 0.003177085 -2.245032538 8.35E-21 3.92E-20

LINC02362 0.032315737 0.251135043 2.958154555 8.55E-21 4.02E-20

BGLAP 0.12450433 0.631912616 2.343529155 8.76E-21 4.11E-20

AC011510.1 0.049217096 0.413731336 3.071462784 8.77E-21 4.11E-20

MAP3K20-AS1 0.023402912 0.094145907 2.008210346 8.82E-21 4.13E-20

RARRES2 42.75623274 127.4521886 1.5757495 9.09E-21 4.26E-20

AC245100.4 0.338447143 1.109618612 1.713061447 9.09E-21 4.26E-20

ETNPPL 1.252024841 0.215209912 -2.540446754 9.20E-21 4.30E-20

SLC2A7 0.017332147 0.0952898 2.458871394 9.24E-21 4.32E-20

NPIPB11 0.065136228 0.337381343 2.37284812 9.34E-21 4.37E-20

SLC13A3 72.00659971 2.805283593 -4.681910613 9.40E-21 4.40E-20

TERT 0.004934442 0.117466173 4.573214667 9.60E-21 4.49E-20

SLC17A4 0.937143787 12.5323201 3.741239295 9.89E-21 4.62E-20

AF064858.1 0.218331442 1.656994642 2.923977124 1.01E-20 4.71E-20

AC025171.5 0.151054002 0.450048807 1.575017061 1.02E-20 4.76E-20

TRGV4 0.064596539 0.307302827 2.25013227 1.03E-20 4.79E-20

LINC01355 0.246016616 1.126133675 2.194550428 1.08E-20 5.01E-20

SNORD63B 0.991436574 5.304064265 2.41950587 1.09E-20 5.07E-20

NRIR 0.057481411 0.321269818 2.48261807 1.20E-20 5.60E-20

GUSBP2 0.137838934 0.424303461 1.622112997 1.21E-20 5.60E-20

PPM1K-DT 0.269934942 0.053963756 -2.322549061 1.21E-20 5.61E-20

GP9 0.031071872 0.153921106 2.308510009 1.22E-20 5.65E-20

AC016773.2 0.107833654 0.677946824 2.652364619 1.23E-20 5.69E-20

LIX1 5.394239722 1.579757224 -1.771716773 1.23E-20 5.71E-20

LRIT3 0.620074711 0.140502306 -2.141848244 1.24E-20 5.75E-20

FFAR2 0.07535404 0.264411352 1.81102734 1.24E-20 5.76E-20

CTSL3P 0.408633627 3.27304904 3.001755385 1.24E-20 5.77E-20

RRN3P2 0.173627052 0.530757282 1.61206052 1.28E-20 5.94E-20

ARHGAP26-AS1 0.013249832 0.180762131 3.770046505 1.34E-20 6.19E-20

AC002091.2 0.206906069 0.703430353 1.765431625 1.35E-20 6.25E-20

LINC00514 0.020054705 0.07656581 1.932759593 1.35E-20 6.25E-20

NAIPP1 0.046442275 0.154493345 1.734034146 1.36E-20 6.27E-20

MALL 0.49131175 1.589124379 1.6935214 1.39E-20 6.42E-20

MIMT1 0.028374186 0.008671052 -1.71029999 1.39E-20 6.45E-20

DLX3 0.45062237 0.095611132 -2.236668431 1.42E-20 6.58E-20

AL365361.1 0.314556736 1.027701595 1.708029262 1.42E-20 6.59E-20

EPHA3 0.854871614 2.921112756 1.772738373 1.42E-20 6.59E-20

AL158151.4 0.035789896 0.307705279 3.10392494 1.48E-20 6.82E-20

AL162741.1 0.046637788 0.230315141 2.304037977 1.56E-20 7.22E-20

LINC01460 0.146067375 0.043045464 -1.762700865 1.60E-20 7.36E-20

KLRC1 0.061161691 0.233621995 1.933475905 1.68E-20 7.76E-20

AC009704.2 0.150205429 0.940764571 2.646896768 1.69E-20 7.77E-20

AC148476.1 0.211363621 1.766309352 3.062939044 1.70E-20 7.82E-20

CYBB 5.948485569 21.40530795 1.847374268 1.71E-20 7.85E-20

Z94721.2 0.020794434 0.08887277 2.095544023 1.71E-20 7.85E-20

AC117383.1 0.061232478 0.364532804 2.573679677 1.73E-20 7.98E-20

Z97056.1 0.0709241 0.689246686 3.280672579 1.78E-20 8.17E-20

SCNN1D 0.205090881 1.118036754 2.446632362 1.79E-20 8.22E-20

AC009133.3 0.024588266 0.122404377 2.315613253 1.79E-20 8.22E-20

TRGV9 0.020194769 0.067425076 1.739303621 1.84E-20 8.46E-20

ENO1-AS1 0.535005874 0.155833734 -1.779547159 1.85E-20 8.52E-20

AC005837.3 0.321768687 1.596520054 2.310834832 1.86E-20 8.54E-20

AL118522.1 0.404388807 0.113843229 -1.828694579 1.87E-20 8.60E-20

ANKRD10-IT1 2.534112981 7.590593428 1.582731832 1.90E-20 8.71E-20

ATP5PBP2 0.025960773 0.008041997 -1.690707668 1.91E-20 8.75E-20

BCO1 1.194116639 3.607608036 1.595098836 1.91E-20 8.76E-20

GGT8P 0.301965661 1.791896263 2.569030716 1.93E-20 8.83E-20

GYG2P1 0.735405855 0.059061156 -3.638259166 1.93E-20 8.83E-20

MYCNOS 0.105583843 0.032453662 -1.70193589 1.94E-20 8.88E-20

AC063977.6 0.012726927 0.074557574 2.550470752 1.94E-20 8.89E-20

AC097658.2 0.16121303 0.480004161 1.574078555 1.94E-20 8.90E-20

AC021146.3 0.300824394 0.077609399 -1.954618272 1.96E-20 8.97E-20

ELAVL4 0.01377857 0.051622974 1.905587047 2.00E-20 9.15E-20

HLA-DRB6 11.64693354 37.90984239 1.702622294 2.02E-20 9.21E-20

AL450384.2 0.302826407 1.05262366 1.797426808 2.02E-20 9.21E-20

AC103719.1 0.011957237 0.117605241 3.297996357 2.02E-20 9.21E-20

AC073655.2 0.13464933 0.510042223 1.921409629 2.04E-20 9.33E-20

IGSF21 0.346311228 1.041709338 1.588811719 2.06E-20 9.39E-20

GPR171 0.414092141 1.356570128 1.711939902 2.10E-20 9.57E-20

LINC01055 9.400381382 0.112579448 -6.383703907 2.10E-20 9.58E-20

HLA-DOB 0.636122774 2.937564404 2.20724334 2.20E-20 1.00E-19

TPSAB1 1.572556854 5.045338535 1.681838898 2.21E-20 1.01E-19

CYP21A1P 0.265406118 1.358733308 2.355988781 2.23E-20 1.01E-19

HNF4A-AS1 2.914970828 0.144329283 -4.336045503 2.24E-20 1.02E-19

H2BP2 0.005920178 0.038582845 2.704246963 2.27E-20 1.03E-19

AC025857.2 0.766397057 2.93842087 1.938877123 2.30E-20 1.05E-19

C2-AS1 0.008887091 0.168224418 4.242532021 2.30E-20 1.05E-19

IGFALS 0.57918144 0.182622971 -1.665147129 2.33E-20 1.06E-19

GPR34 2.830308182 9.108638451 1.686276266 2.33E-20 1.06E-19

CCR6 0.020535865 0.123027039 2.582757822 2.34E-20 1.06E-19

AC246787.1 1.136922089 3.321617631 1.546752614 2.35E-20 1.07E-19

LINC00298 0.005132372 0.049962174 3.283138601 2.35E-20 1.07E-19

HEYL 7.11452725 22.30896922 1.648784056 2.43E-20 1.10E-19

AC005776.2 0.069657548 0.330542771 2.246485364 2.49E-20 1.13E-19

AC026316.3 0.074597071 0.012901981 -2.531526386 2.51E-20 1.14E-19

AL589182.2 0.009445158 0.098907257 3.388429475 2.54E-20 1.15E-19

CFAP77 0.092112133 0.028532111 -1.690804715 2.55E-20 1.15E-19

AC025178.2 0.083500441 0.343700725 2.04129718 2.56E-20 1.16E-19

AC010323.2 0.021082458 0.148460907 2.815968097 2.60E-20 1.18E-19

AC002059.1 0.089059067 0.529891689 2.572863097 2.61E-20 1.18E-19

AC013565.3 0.100185354 0.033767392 -1.568968972 2.64E-20 1.19E-19

SNORA5C 0.569443038 2.315415503 2.02364767 2.73E-20 1.24E-19

C19orf33 8.927035264 47.67551722 2.41699556 2.81E-20 1.27E-19

AC068196.1 0.017532434 0.167226048 3.253701367 2.91E-20 1.31E-19

FAM83E 0.533412917 0.102362912 -2.381559673 2.96E-20 1.34E-19

BTNL9 2.535815522 11.89983962 2.23042043 2.96E-20 1.34E-19

AP006623.1 0.196932677 0.93836278 2.252443273 3.04E-20 1.37E-19

TLR10 0.142757554 0.527868606 1.886611782 3.06E-20 1.38E-19

OBP2B 0.003735112 0.047486279 3.66828742 3.08E-20 1.39E-19

ALDOB 1633.284657 63.12515953 -4.693417314 3.15E-20 1.42E-19

WDR97 0.034583582 0.195018633 2.495452777 3.15E-20 1.42E-19

AL031708.1 0.010981942 0.056314507 2.358373442 3.15E-20 1.42E-19

RNF14P4 0.026730193 0.152843226 2.515510467 3.16E-20 1.42E-19

DCST1 0.0178158 0.103609814 2.539931459 3.16E-20 1.42E-19

LINC02605 0.074267851 0.244609852 1.719672775 3.17E-20 1.42E-19

APOLD1 9.551160456 38.6446188 2.016519601 3.21E-20 1.44E-19

AL603839.4 0.02649574 0.114455302 2.110951962 3.41E-20 1.53E-19

OR52N4 0.069167246 0.420757039 2.60482649 3.42E-20 1.53E-19

AC008764.8 0.129767693 0.510344489 1.975540159 3.45E-20 1.55E-19

AC090971.4 0.083883455 0.311712105 1.893756 3.46E-20 1.55E-19

CHRNA4 0.349798217 0.041219517 -3.085123438 3.50E-20 1.57E-19

AL033543.1 0.001853124 0.035925467 4.276975694 3.56E-20 1.59E-19

LINC01031 0.1324026 0.028830928 -2.199242265 3.59E-20 1.61E-19

RPE65 0.006168873 0.041521298 2.750772697 3.59E-20 1.61E-19

CLVS2 0.005982607 0.070357926 3.555866784 3.59E-20 1.61E-19

DGAT2L7P 0.010046357 0.147492972 3.875901808 3.64E-20 1.63E-19

AP001636.1 0.01723994 0.109741132 2.670277667 3.70E-20 1.66E-19

AC015849.3 0.466717133 1.71093629 1.874165705 3.75E-20 1.68E-19

CSAG1 0.039104925 0.276996601 2.824446068 3.81E-20 1.70E-19

AC010886.1 0.004293861 0.116381428 4.76044141 3.85E-20 1.72E-19

AL158824.2 0.116785795 0.03826199 -1.609880984 3.86E-20 1.72E-19

AC093915.1 0.040442217 0.512529181 3.663700165 3.90E-20 1.74E-19

LINC00565 0.02752078 0.119319989 2.116242484 3.93E-20 1.75E-19

ALK 0.017966837 0.064570417 1.84553688 3.96E-20 1.77E-19

PCDH15 0.110663492 0.007688812 -3.8472748 4.00E-20 1.78E-19

ZNF80 0.013279371 0.084607457 2.671598051 4.02E-20 1.79E-19

AC005253.1 0.241345153 0.82706465 1.776902254 4.04E-20 1.80E-19

RAPGEF4-AS1 0.001704631 0.021920468 3.684747496 4.05E-20 1.81E-19

LINC02195 0.027041274 0.297716564 3.460704474 4.15E-20 1.85E-19

DNASE2B 0.037669388 0.283850006 2.913664269 4.36E-20 1.94E-19

FHL1 17.83232988 57.64560407 1.692715385 4.44E-20 1.97E-19

TPT1P5 0.19946955 0.909946695 2.189613506 4.45E-20 1.98E-19

MT1F 47.98363982 10.68370914 -2.167129993 4.46E-20 1.99E-19

AC092941.1 0.081182683 0.006521113 -3.637981933 4.56E-20 2.03E-19

KCNN4 0.325663654 0.927194318 1.50948901 4.65E-20 2.06E-19

AC087672.2 0.038628444 0.140935501 1.867299578 4.75E-20 2.11E-19

MRO 6.735794965 0.91192082 -2.884867756 4.83E-20 2.15E-19

RPL18P10 0.034062344 0.28931865 3.086409715 4.85E-20 2.15E-19

AC090589.3 0.409017473 1.357323426 1.730530151 4.99E-20 2.22E-19

PPDPFL 0.685562165 4.780564735 2.801821659 5.24E-20 2.32E-19

TCL6 1.450816965 0.236554236 -2.616622618 5.37E-20 2.38E-19

MC1R 0.264908213 0.799431296 1.59348148 5.41E-20 2.39E-19

HTR7 0.115943217 0.400627176 1.788841871 5.48E-20 2.42E-19

AC024361.3 0.045829807 0.356237808 2.958482514 5.51E-20 2.44E-19

AC008467.1 0.029966757 0.127001916 2.083415379 5.53E-20 2.45E-19

UTS2 0.030193772 0.21105439 2.805291952 5.53E-20 2.45E-19

MUC16 0.005035521 0.035033404 2.798518127 5.81E-20 2.57E-19

AC000120.1 0.315899972 1.658531715 2.392366884 5.90E-20 2.60E-19

JPH2 0.9676099 3.040657713 1.651885986 5.93E-20 2.62E-19

AC096642.1 0.071196627 0.310014922 2.122456852 6.13E-20 2.70E-19

C11orf88 0.020716586 0.064205842 1.631918311 6.26E-20 2.76E-19

CARD14 0.069239688 0.427836773 2.627389363 6.29E-20 2.77E-19

FAM151A 57.19443873 3.926809124 -3.864445497 6.41E-20 2.82E-19

AC027288.3 1.896463498 0.285271068 -2.73290626 6.41E-20 2.82E-19

LINC02694 0.003099137 0.029463854 3.249007519 6.41E-20 2.82E-19

TRDV1 0.137387798 0.746249974 2.441405095 6.49E-20 2.86E-19

MUC5B 0.008756929 0.049196725 2.49006536 6.55E-20 2.88E-19

FSTL3 11.03094181 36.62563896 1.731297957 6.63E-20 2.91E-19

TRBV6-1 0.15718123 0.85341478 2.440818147 6.70E-20 2.95E-19

KLRC2 0.016971242 0.095282756 2.489123024 6.83E-20 3.00E-19

HMGB3P32 0.037206387 0.228376937 2.617794766 6.99E-20 3.07E-19

AC233300.1 0.088627751 0.438261927 2.305962946 7.07E-20 3.10E-19

AL157935.3 0.091951719 0.416358534 2.178877951 7.17E-20 3.15E-19

CCNL2 3.985122583 14.25868958 1.839145392 7.27E-20 3.19E-19

PREX2 1.238045343 5.347232305 2.110728201 7.27E-20 3.19E-19

FAM230I 0.357568262 0.044881139 -2.994037502 7.27E-20 3.19E-19

AL096701.1 0.073557564 0.380780096 2.372012458 7.35E-20 3.22E-19

CHKB 0.561143344 1.851868177 1.722540145 7.36E-20 3.23E-19

TRAV12-1 0.194002807 1.310076449 2.755501573 7.45E-20 3.26E-19

AC008735.2 0.60931559 3.237210055 2.409489424 7.61E-20 3.33E-19

U62317.4 0.056263419 0.352408609 2.646980041 7.80E-20 3.42E-19

SCGN 1.127248955 30.33762812 4.750230218 7.96E-20 3.49E-19

BTNL10 0.045314035 0.131525545 1.537313168 8.08E-20 3.54E-19

MCIDAS 0.14356578 0.040765072 -1.816306435 8.17E-20 3.57E-19

AC023906.5 0.126820873 0.476635068 1.910092889 8.17E-20 3.58E-19

SIGLEC18P 0.006388393 0.071678578 3.488017154 8.26E-20 3.61E-19

LINC02097 0.022044811 0.116114037 2.397031374 8.40E-20 3.67E-19

LINC02061 6.409707789 0.701613794 -3.19150957 8.42E-20 3.68E-19

AC008764.6 0.158822782 0.479355328 1.593677599 8.50E-20 3.71E-19

C8orf17 0.001566228 0.04306069 4.781005184 8.61E-20 3.76E-19

C20orf203 0.006560768 0.055879818 3.09039078 8.63E-20 3.76E-19

AL158163.2 0.13578472 0.388466221 1.516468012 8.84E-20 3.86E-19

MTND4P9 0.027108826 0.124328549 2.197323072 8.90E-20 3.88E-19

AL023653.2 0.162942959 0.516977177 1.665733583 8.95E-20 3.90E-19

CRYZL2P-SEC16B 0.100537364 0.337087303 1.745390514 9.08E-20 3.96E-19

LINC02703 0.009056442 0.252962234 4.803833867 9.08E-20 3.96E-19

LINC01589 1.196982837 0.192172886 -2.63892576 9.18E-20 4.00E-19

AC116407.3 0.035846712 0.154074133 2.103711988 9.24E-20 4.02E-19

HCG4B 0.435287967 1.790990774 2.040715863 9.38E-20 4.08E-19

ASIC3 0.110392172 0.45563984 2.045256023 9.38E-20 4.08E-19

STAG3L5P 0.671310943 2.993066756 2.156571391 9.82E-20 4.27E-19

ST13P20 0.061497757 0.018405922 -1.740363778 9.94E-20 4.32E-19

CCR7 0.523290493 1.614489439 1.625394051 1.00E-19 4.35E-19

PLP1 0.269390244 0.06255718 -2.106450217 1.00E-19 4.35E-19

AC109446.3 0.03300969 0.175920929 2.413965644 1.01E-19 4.40E-19

C4B 3.488472213 11.63988239 1.738409234 1.01E-19 4.41E-19

KCNJ9 0.108510814 0.033654769 -1.68895597 1.02E-19 4.42E-19

NPIPB12 0.021018755 0.093244234 2.149337275 1.04E-19 4.52E-19

CDKN2A-DT 0.001114762 0.036504991 5.033286695 1.05E-19 4.54E-19

ABCA8 2.512803188 0.663691116 -1.92071381 1.07E-19 4.64E-19

KCNQ5 0.039050476 0.135086869 1.790475396 1.08E-19 4.66E-19

LGR5 0.756553736 0.121060404 -2.643715487 1.08E-19 4.68E-19

AC096531.2 0.476646588 0.147280328 -1.694355228 1.09E-19 4.72E-19

AC145207.9 0.166395745 0.952999761 2.51785731 1.10E-19 4.75E-19

TRBJ2-7 0.354102063 2.373454182 2.744751048 1.10E-19 4.76E-19

TRBV5-6 0.114617708 0.736621355 2.684093268 1.11E-19 4.80E-19

ANKRD36 0.146064745 0.519656781 1.830951077 1.11E-19 4.81E-19

AC007613.2 0.05404938 0.380339302 2.814937043 1.12E-19 4.85E-19

LRRC37A15P 0.077409195 0.265325007 1.777183799 1.12E-19 4.86E-19

AC092162.3 0.270544144 0.864878032 1.676632681 1.13E-19 4.90E-19

LINC01126 0.137911907 0.52035866 1.91575933 1.16E-19 5.00E-19

TRAV2 0.119579648 0.705460227 2.56059288 1.16E-19 5.02E-19

HAUS7 0.456368492 1.733783817 1.925652927 1.16E-19 5.03E-19

PTPRQ 0.181420444 0.012713683 -3.834883094 1.18E-19 5.10E-19

PDE2A-AS2 0.112589153 0.353161506 1.649260257 1.19E-19 5.12E-19

TNFRSF25 0.642991257 2.448286157 1.928901165 1.19E-19 5.12E-19

AC133637.1 0.000415076 0.052873874 6.993036054 1.19E-19 5.13E-19

SLC5A2 8.089921186 0.329811965 -4.616410003 1.19E-19 5.15E-19

AC073941.1 0.121248687 0.042271485 -1.520212419 1.20E-19 5.19E-19

WT1 5.661493042 0.999446581 -2.501981205 1.21E-19 5.21E-19

AL590787.1 0.181477333 0.062840751 -1.530017032 1.21E-19 5.23E-19

AC093829.1 0.034547801 0.005017176 -2.783646564 1.22E-19 5.23E-19

SPIC 0.011538868 0.175653602 3.928159568 1.25E-19 5.38E-19

AC005899.2 0.026454198 0.197372407 2.89935174 1.27E-19 5.47E-19

TSPAN16 0.00855377 0.068562155 3.002780122 1.31E-19 5.65E-19

LINC02705 0.017466007 0.165250538 3.242033221 1.32E-19 5.66E-19

ARSH 0.040509803 0.005321176 -2.928454167 1.36E-19 5.82E-19

RETN 0.080380331 0.512194119 2.671776271 1.36E-19 5.85E-19

Z98257.1 0.009198857 0.225992518 4.618676608 1.38E-19 5.93E-19

AC008750.2 0.005628509 0.050616562 3.168784737 1.38E-19 5.93E-19

HK2P1 0.003454311 0.031523259 3.189946688 1.40E-19 5.99E-19

AL161668.3 0.03729688 0.216062204 2.534319857 1.47E-19 6.30E-19

AC007881.3 0.398255527 0.104959464 -1.923862123 1.48E-19 6.32E-19

SPHKAP 0.0306111 0.010703707 -1.515944346 1.50E-19 6.42E-19

AC008569.2 0.056332537 0.251062942 2.156008736 1.51E-19 6.45E-19

CHRND 0.004821205 0.055963291 3.537015208 1.52E-19 6.48E-19

AC012066.1 0.033962885 0.232640698 2.776072594 1.55E-19 6.62E-19

AQP7P1 1.063368198 0.213133942 -2.318808954 1.57E-19 6.73E-19

H1-0 182.7320093 48.2787236 -1.920269935 1.58E-19 6.76E-19

AL161452.1 0.058053673 0.246043353 2.083453281 1.59E-19 6.79E-19

AC005104.1 0.290904015 1.230954214 2.081161986 1.63E-19 6.95E-19

TRAV8-6 0.15330211 0.827524867 2.43242511 1.64E-19 6.99E-19

SRSF9P1 0.130479737 0.380115444 1.542611862 1.66E-19 7.10E-19

DDX39B 3.716356333 11.56706923 1.638062629 1.67E-19 7.10E-19

NRBF2P5 0.066555866 0.300421489 2.174350283 1.72E-19 7.31E-19

SNORD14A 0.824523808 3.752927079 2.186383201 1.72E-19 7.33E-19

NYAP1 0.590700408 0.093840923 -2.654137492 1.72E-19 7.33E-19

ARMC12 0.089948121 0.371689272 2.046932003 1.72E-19 7.33E-19

LINC00460 0.030224813 0.994363976 5.03996869 1.74E-19 7.41E-19

WNT4 0.811476534 0.215655529 -1.911820673 1.74E-19 7.42E-19

IL17REL 0.007216656 0.048230172 2.740533618 1.75E-19 7.45E-19

SLC2A14 0.028615941 0.151614152 2.405513481 1.76E-19 7.49E-19

AC051619.1 0.005578915 0.122231714 4.453490196 1.82E-19 7.74E-19

AC107081.2 0.067362323 0.309622893 2.200498356 1.85E-19 7.84E-19

AL109811.1 0.020496836 0.098513069 2.264913941 1.85E-19 7.86E-19

PTPN7 0.664340028 2.307957304 1.796622786 1.91E-19 8.10E-19

LINC02828 0.029520076 0.135212466 2.195459814 1.98E-19 8.39E-19

AL109659.2 0.08474623 0.3250274 1.939340252 1.99E-19 8.45E-19

AC073487.1 0.133786008 0.604039306 2.174715197 2.05E-19 8.71E-19

ERMN 0.035008035 0.149159665 2.091099479 2.06E-19 8.75E-19

TAS2R6P 0.06481485 0.280608522 2.114162522 2.10E-19 8.89E-19

CYP27B1 5.799186388 0.492092382 -3.558849421 2.10E-19 8.91E-19

PLA2G2D 0.10591699 1.17059791 3.466239676 2.11E-19 8.95E-19

SNAP25 0.645310559 2.606078364 2.013814929 2.12E-19 8.96E-19

SIGLEC22P 0.064944098 0.249275523 1.940470895 2.13E-19 9.00E-19

CEP164P1 0.013599324 0.067166395 2.304204644 2.13E-19 9.02E-19

AC130469.1 0.057492006 0.584919001 3.346803578 2.14E-19 9.07E-19

SIGLEC14 0.703643475 2.200836907 1.645135711 2.17E-19 9.18E-19

EME2 0.541334508 2.03330192 1.90923219 2.23E-19 9.42E-19

AC026458.2 0.144867045 0.048848797 -1.568334492 2.26E-19 9.53E-19

AL031846.2 0.165472565 0.609876724 1.88192562 2.26E-19 9.54E-19

RPL7L1P9 0.023536097 0.308625371 3.712909682 2.28E-19 9.61E-19

SAPCD1 0.072301114 0.596161888 3.043614375 2.29E-19 9.65E-19

PTCHD3 0.077880716 0.020581227 -1.919937135 2.29E-19 9.67E-19

C4A 3.652500701 12.22123267 1.742433353 2.33E-19 9.84E-19

SLC22A8 36.29032022 0.563868794 -6.008081474 2.37E-19 1.00E-18

RPS20P4 0.042325676 0.271253861 2.680038667 2.38E-19 1.00E-18

AL031717.1 0.122615147 0.591304273 2.269763488 2.41E-19 1.01E-18

BMP6 14.4672776 3.283360442 -2.139548426 2.46E-19 1.03E-18

AC083867.2 0.063297674 0.021705219 -1.544110533 2.51E-19 1.06E-18

C2 1.981194317 8.994835323 2.182726481 2.57E-19 1.08E-18

LINC01780 0.850135904 0.244153942 -1.799902419 2.65E-19 1.11E-18

IRF9 0.563746139 1.906369183 1.757709983 2.67E-19 1.12E-18

TRBV3-1 0.200870173 1.049109697 2.384830274 2.68E-19 1.12E-18

AP000897.2 0.070933636 0.489897744 2.787938848 2.70E-19 1.13E-18

AC005041.2 0.247789167 0.032275623 -2.94059626 2.73E-19 1.15E-18

AC004832.4 0.091147509 0.419442037 2.202196315 2.73E-19 1.15E-18

CREB3L3 1.220201108 8.002731616 2.713373581 2.74E-19 1.15E-18

AC100830.1 0.04234937 0.165222175 1.963994928 2.76E-19 1.16E-18

WNT10B 0.036286564 0.166401282 2.197159203 2.78E-19 1.16E-18

SCARNA6 0.146265926 18.81642477 7.007255006 2.80E-19 1.17E-18

SNORD94 0.613890572 9.947254898 4.018245028 2.81E-19 1.18E-18

RNU1-47P 0.086700914 0.819308348 3.240287401 2.82E-19 1.18E-18

AXDND1 0.177767955 0.047048361 -1.917778914 2.89E-19 1.21E-18

TRAV29DV5 0.162362257 1.008920985 2.635524988 2.90E-19 1.21E-18

AL161719.1 0.753366417 0.191812089 -1.973658076 2.93E-19 1.23E-18

MIR4477B 0.507853347 1.751291947 1.785935751 2.97E-19 1.24E-18

BX284613.2 0.174220522 0.739341759 2.08532683 2.97E-19 1.24E-18

EIPR1-IT1 0.339329813 0.068835787 -2.301457483 3.04E-19 1.27E-18

LYZL1 0.006315682 0.145010296 4.521072932 3.04E-19 1.27E-18

AC073850.1 0.062646858 0.642528592 3.358446603 3.04E-19 1.27E-18

AL137247.1 0.257971799 1.41548377 2.456009943 3.09E-19 1.29E-18

AC006064.1 0.039086055 0.223274369 2.514091754 3.13E-19 1.30E-18

TRAV12-3 0.169048066 1.006449563 2.573769456 3.13E-19 1.31E-18

C1orf147 0.016416033 0.09407719 2.518739448 3.20E-19 1.33E-18

HS3ST5 0.222191209 0.023705017 -3.228537385 3.21E-19 1.34E-18

RIMBP3C 0.001295976 0.008648561 2.738420828 3.26E-19 1.36E-18

ITGB1-DT 0.074538423 0.298452208 2.001443732 3.27E-19 1.36E-18

BMX 0.376925734 1.104408092 1.550921162 3.29E-19 1.37E-18

CHCHD2P7 0.430581815 0.122205491 -1.816978277 3.31E-19 1.38E-18

P2RY10 0.409902844 1.636432905 1.997200545 3.36E-19 1.40E-18

LINC01767 0.04520809 0.235749603 2.382602472 3.41E-19 1.42E-18

CADM3-AS1 0.036551332 0.170315218 2.22021147 3.41E-19 1.42E-18

AP000695.3 0.030750333 0.147380344 2.26087019 3.43E-19 1.43E-18

AP003357.1 1.600164907 0.543142429 -1.55881812 3.43E-19 1.43E-18

TMPRSS12 0.048641985 0.015212987 -1.676898671 3.48E-19 1.44E-18

AC026471.3 0.916604604 0.190983233 -2.262853549 3.50E-19 1.46E-18

KLRC4-KLRK1 0.022598509 0.115319575 2.351337934 3.53E-19 1.47E-18

MIR140 0.80691875 5.594120875 2.793416109 3.60E-19 1.49E-18

AC093788.1 0.138315563 0.584969991 2.080399126 3.62E-19 1.50E-18

TRBV6-6 0.108635302 0.675095226 2.635598024 3.66E-19 1.52E-18

TRAV13-1 0.276330233 1.49609232 2.436733886 3.73E-19 1.55E-18

PCK1 149.2913056 22.33713424 -2.740614137 3.76E-19 1.56E-18

MIR6503 0.071031215 0.835919587 3.556839093 3.86E-19 1.60E-18

TTN 0.043473493 0.167684732 1.947543425 3.86E-19 1.60E-18

AC092143.3 0.028850592 0.217073421 2.911510266 3.91E-19 1.62E-18

ZDHHC19 0.023928049 0.092610415 1.952471691 4.02E-19 1.66E-18

AC011603.3 0.040794481 0.138627557 1.764768189 4.04E-19 1.67E-18

AC010761.1 0.345711257 1.065560814 1.623973447 4.06E-19 1.68E-18

AC013287.1 0.123850598 0.043491422 -1.509798056 4.07E-19 1.68E-18

TRAV14DV4 0.124019138 0.70335574 2.503691783 4.07E-19 1.68E-18

Z99916.2 0.047990124 0.279260643 2.540802803 4.08E-19 1.69E-18

MASP1 0.967176061 4.030233341 2.059012928 4.09E-19 1.69E-18

SLC6A1-AS1 0.062080255 0.38010067 2.614175168 4.09E-19 1.69E-18

AC009102.2 0.075994744 0.019478444 -1.964021238 4.15E-19 1.71E-18

CCDC39-AS1 0.10230375 0.024644772 -2.053505487 4.16E-19 1.72E-18

TRAV16 0.12231083 0.661238388 2.43461833 4.26E-19 1.76E-18

AC011933.1 0.069031232 0.27919662 2.015960346 4.27E-19 1.76E-18

ADCY10 0.037129373 0.143673253 1.952158643 4.28E-19 1.77E-18

TRBJ2-2P 0.265883929 1.528393628 2.523147664 4.37E-19 1.80E-18

AC087284.1 0.018044317 0.253644979 3.813194183 4.37E-19 1.80E-18

AC004951.3 0.095118798 0.339192163 1.834300455 4.42E-19 1.82E-18

RN7SL172P 0.042553106 0.329834557 2.954406201 4.48E-19 1.85E-18

RP1 0.120831667 0.040365843 -1.581791679 4.50E-19 1.85E-18

SNORA77 0.117443957 0.703215212 2.581993792 4.51E-19 1.86E-18

AC079340.1 0.067909879 0.008590559 -2.982797585 4.56E-19 1.88E-18

CGB7 0.008057242 0.043756381 2.441135394 4.58E-19 1.88E-18

AL359399.1 0.040462567 0.400934604 3.308707183 4.61E-19 1.90E-18

DAAM2-AS1 0.100026162 0.680252753 2.765693495 4.65E-19 1.91E-18

AC023818.1 0.112477131 0.38616023 1.779567891 4.71E-19 1.94E-18

SNORD6 0.775640914 2.907899137 1.906516418 4.72E-19 1.94E-18

AC147067.2 0.238913819 1.252577816 2.390338023 4.78E-19 1.96E-18

LINC02015 0.355904689 2.235593755 2.651095206 4.83E-19 1.99E-18

AC006128.1 0.341817386 1.677452632 2.294974343 4.86E-19 2.00E-18

TRAV13-2 0.121282795 0.650105494 2.422298941 4.92E-19 2.02E-18

AC026461.4 0.129329745 0.038198771 -1.759456013 4.92E-19 2.02E-18

AC027607.1 0.160421123 0.489209562 1.608588486 5.02E-19 2.06E-18

VNN2 1.094486039 3.860287269 1.81845466 5.05E-19 2.07E-18

H2BC13 0.044490352 0.352915892 2.987759994 5.08E-19 2.08E-18

AL662899.1 0.466685543 1.92935934 2.047599186 5.11E-19 2.10E-18

HNRNPA1P27 0.045625098 0.228187521 2.322320347 5.17E-19 2.12E-18

SKINT1L 0.036387577 0.170860375 2.231299944 5.24E-19 2.15E-18

SHOX2 0.026889015 0.138010835 2.359692729 5.25E-19 2.15E-18

AC018755.4 0.460884291 1.392503397 1.595204346 5.28E-19 2.16E-18

SLC5A4 0.295514144 1.068480623 1.854261656 5.39E-19 2.21E-18

AC011466.3 0.018302057 0.110233328 2.590482769 5.43E-19 2.22E-18

TRAV12-2 0.218301273 1.35452236 2.633391757 5.45E-19 2.23E-18

TRBV11-2 0.139708356 0.863098204 2.627106406 5.48E-19 2.24E-18

RSL24D1P11 0.022401611 0.162160728 2.855750075 5.49E-19 2.25E-18

TRAV3 0.116137831 0.748555373 2.688271044 5.58E-19 2.28E-18

LRRC77P 0.024716884 0.141600091 2.518253409 5.61E-19 2.30E-18

AC007639.1 0.065388545 0.503103809 2.943746282 5.68E-19 2.32E-18

ETV7-AS1 0.129546329 0.455585185 1.814252696 5.74E-19 2.35E-18

ALDH4A1 68.44591693 16.63498608 -2.040743823 5.93E-19 2.42E-18

HOXC13 0.03539602 0.131091017 1.888909754 6.01E-19 2.45E-18

ZFPM2-AS1 0.396056096 1.701299423 2.102860385 6.01E-19 2.45E-18

SATL1 0.040239473 0.011553868 -1.800235528 6.09E-19 2.49E-18

LINC00160 0.012243723 0.238290277 4.282605866 6.18E-19 2.52E-18

AC007016.1 0.059794261 0.393515102 2.71834008 6.23E-19 2.54E-18

SPHK1 0.60873218 1.930841201 1.665349977 6.24E-19 2.54E-18

AC138512.1 0.162518894 0.035738553 -2.185054332 6.25E-19 2.55E-18

TRBV24-1 0.099810743 0.523539388 2.391031074 6.28E-19 2.56E-18

BX255925.1 0.260464784 0.788156688 1.597394143 6.32E-19 2.58E-18

AC018450.1 0.106235756 0.367007395 1.78853972 6.44E-19 2.62E-18

AC116366.1 0.57729071 2.630437463 2.187932838 6.44E-19 2.62E-18

UBE2Q1-AS1 0.096730846 0.431441603 2.157117384 6.45E-19 2.63E-18

KCNK10 0.694313116 0.074084144 -3.228349723 6.57E-19 2.67E-18

CCDC194 0.018068788 0.165304333 3.193552894 6.58E-19 2.68E-18

AL021407.3 0.041406063 0.224180169 2.436744734 6.74E-19 2.74E-18

PERM1 0.265677277 2.254864083 3.085293726 6.74E-19 2.74E-18

AC079793.1 0.013316394 0.176229455 3.726179718 6.77E-19 2.75E-18

CALB2 0.097860397 0.377642218 1.94822301 6.93E-19 2.81E-18

RGSL1 0.000767219 0.008690566 3.501739324 7.12E-19 2.89E-18

AC079922.1 0.9746661 3.070877361 1.655670926 7.14E-19 2.90E-18

AL353795.2 0.135352237 0.043167783 -1.648691815 7.15E-19 2.90E-18

TMPRSS5 0.059097502 0.218376724 1.885650037 7.18E-19 2.91E-18

LGR6 0.632048805 0.210063396 -1.589211175 7.28E-19 2.95E-18

USP32P3 0.04580543 0.199383625 2.121956384 7.37E-19 2.99E-18

PRAL 0.017405835 0.081539274 2.227924055 7.47E-19 3.03E-18

OBP2A 0.005416306 0.116089209 4.421780823 7.55E-19 3.06E-18

AP000593.3 0.046869507 0.214859842 2.196674352 7.63E-19 3.09E-18

LINC01426 0.747948715 4.168572739 2.478542252 7.71E-19 3.12E-18

CDHR1 0.211467849 3.991554242 4.238440375 7.71E-19 3.12E-18

ANKRD23 0.076018372 0.238487592 1.64949417 7.76E-19 3.14E-18

ZP3 0.67016821 1.945932893 1.537866801 7.81E-19 3.16E-18

AL391840.1 0.465619772 0.13487627 -1.787515774 7.98E-19 3.22E-18

C9orf152 0.185779835 0.055791135 -1.735486108 8.01E-19 3.23E-18

SCART1 0.070071317 0.347844668 2.311547278 8.01E-19 3.23E-18

AC006148.2 0.015891718 0.322356575 4.342310366 8.02E-19 3.24E-18

AC021744.1 1.716990777 15.52936538 3.177044679 8.18E-19 3.30E-18

RGS20 0.049943709 0.262410345 2.393449724 8.21E-19 3.32E-18

AL590560.2 0.514288684 0.037311963 -3.784868246 8.50E-19 3.43E-18

CRYBB2 0.091891097 0.315006788 1.77738592 8.84E-19 3.56E-18

SLC9A3-AS1 1.743309179 14.67071627 3.073038947 8.87E-19 3.57E-18

AC092135.3 0.029572338 0.157227044 2.410529176 8.90E-19 3.59E-18

CTAGE6 0.002542347 0.022862315 3.168738757 8.91E-19 3.59E-18

AP005131.4 0.007155844 0.126828194 4.147609688 9.09E-19 3.66E-18

GSG1L2 0.004601747 7.758228835 10.71932983 9.38E-19 3.77E-18

AC055855.2 0.133914978 0.511256362 1.932729559 9.50E-19 3.82E-18

AC097713.1 0.002792501 0.100949428 5.17593107 9.57E-19 3.84E-18

VCAN-AS1 0.021682237 0.186450321 3.104205794 9.65E-19 3.87E-18

NGFR 2.313789089 8.467775684 1.871725689 9.76E-19 3.91E-18

AP001992.1 0.188532858 0.812645846 2.107810774 9.76E-19 3.91E-18

IL2RA 0.334480075 1.620337851 2.276302479 9.76E-19 3.91E-18

PRDM7 0.037542211 0.009543549 -1.975915774 9.90E-19 3.97E-18

LINC01702 2.552884988 0.345894001 -2.883726646 9.95E-19 3.99E-18

ANTXRLP1 0.012845287 0.085247669 2.730421238 1.04E-18 4.16E-18

TRIM46 0.131881852 0.508832042 1.947943472 1.05E-18 4.22E-18

LINC01786 0.196841294 1.039864447 2.401290667 1.06E-18 4.24E-18

AC103724.4 0.057850022 0.25321157 2.129953906 1.07E-18 4.27E-18

AC073346.2 0.007925256 0.044552925 2.490990652 1.07E-18 4.28E-18

AC006329.1 0.194328304 0.045543713 -2.093172224 1.07E-18 4.30E-18

TMEM132C 0.320381827 0.082270925 -1.96133774 1.08E-18 4.32E-18

STAG3L5P-PVRIG2P-PILRB 0.25937769 0.989454774 1.931579369 1.11E-18 4.46E-18

AC073575.4 0.140452414 0.399035432 1.506435432 1.12E-18 4.48E-18

COL21A1 0.430954178 2.071087579 2.264782176 1.12E-18 4.48E-18

DPY19L2P1 0.01299374 0.150532961 3.534190747 1.13E-18 4.51E-18

CD163 4.77661625 17.76826151 1.89524165 1.13E-18 4.51E-18

AC074286.1 0.131842682 0.49110517 1.897214516 1.14E-18 4.54E-18

TRAV5 0.092755032 0.600441044 2.694525143 1.14E-18 4.56E-18

WNT11 1.353755155 0.465916315 -1.538824076 1.14E-18 4.57E-18

AC005165.1 0.750683948 0.045267406 -4.051661094 1.15E-18 4.61E-18

MUC17 0.000286631 0.177844166 9.277200799 1.16E-18 4.62E-18

CCL20 3.115283676 17.81231427 2.515439526 1.17E-18 4.65E-18

KCNK3 3.464236917 14.98458001 2.112869145 1.20E-18 4.77E-18

AC002558.1 0.021212787 0.136455604 2.685425575 1.21E-18 4.82E-18

SEC14L3 0.001775193 0.050958978 4.843288641 1.22E-18 4.87E-18

AC025171.3 0.089514831 0.295542209 1.723165548 1.28E-18 5.09E-18

TRBV12-4 0.138878832 0.845183387 2.60543769 1.30E-18 5.19E-18

PDE6B-AS1 0.009347143 0.091023467 3.283641094 1.34E-18 5.32E-18

AC008735.1 0.061577444 0.371671466 2.593554034 1.34E-18 5.33E-18

MIR6728 0.049133807 0.5571913 3.503384803 1.34E-18 5.33E-18

AC091849.2 0.138060852 0.622669615 2.173162591 1.40E-18 5.56E-18

KRT72 0.003466177 0.050724608 3.871268586 1.41E-18 5.58E-18

AC009902.2 0.053535656 0.168289844 1.652376131 1.42E-18 5.64E-18

TRBV18 0.158751505 0.818320916 2.365896461 1.42E-18 5.65E-18

SNAP25-AS1 0.041873276 0.156352164 1.900697477 1.42E-18 5.65E-18

SLPI 166.7490311 48.27262438 -1.788401211 1.44E-18 5.70E-18

AC009948.2 0.064688952 0.183632221 1.505227986 1.48E-18 5.88E-18

AC016769.3 0.031979412 0.604939504 4.24157554 1.48E-18 5.88E-18

CEBPA 1.50495391 4.269424801 1.504322411 1.49E-18 5.91E-18

LRAT 0.136894504 0.655736692 2.2600521 1.50E-18 5.95E-18

AC068790.2 0.1267637 0.46818075 1.884923939 1.51E-18 5.98E-18

AC011290.2 0.191914794 0.68217528 1.829676549 1.51E-18 5.98E-18

GSC 0.058884432 0.218356727 1.890728811 1.53E-18 6.05E-18

PTOV1-AS2 0.642868247 2.771483232 2.10806328 1.53E-18 6.06E-18

FXYD7 0.035496919 0.118390974 1.737793397 1.54E-18 6.10E-18

OR7E102P 0.248677417 0.059820287 -2.05556875 1.56E-18 6.17E-18

UCA1 0.52940671 0.113915645 -2.216410587 1.59E-18 6.30E-18

MIR1249 0.134043078 0.735103214 2.455250105 1.61E-18 6.36E-18

SEMA6A-AS2 0.140508804 0.43234198 1.621512397 1.63E-18 6.45E-18

AL023881.1 0.177253684 0.536215737 1.596997948 1.68E-18 6.61E-18

SLC12A9-AS1 0.246444155 0.724152224 1.555032235 1.68E-18 6.63E-18

HAVCR2 6.370026603 23.52073779 1.884562012 1.68E-18 6.63E-18

AL139351.1 0.088745039 0.63526481 2.839619728 1.69E-18 6.67E-18

CRLF2 0.018149847 0.108204443 2.575730428 1.72E-18 6.77E-18

MIR7848 0.052277588 0.719554979 3.782840452 1.72E-18 6.78E-18

AL021707.6 0.457953759 1.920309812 2.06806525 1.75E-18 6.88E-18

VNN3 0.017112872 0.104361309 2.608433176 1.76E-18 6.94E-18

AC093162.3 0.473383946 1.429102835 1.594027046 1.83E-18 7.19E-18

AC005387.1 0.085842017 0.450948958 2.393208269 1.84E-18 7.22E-18

PLA2R1 10.68592537 1.316313958 -3.021136309 1.84E-18 7.23E-18

AC011815.3 0.325129649 1.117828123 1.781611348 1.91E-18 7.50E-18

TNXA 0.069539539 0.531437018 2.933993321 1.92E-18 7.56E-18

LINC01621 0.149132211 0.034447678 -2.114113237 1.95E-18 7.68E-18

IGFL2 0.008924422 0.189955892 4.411761959 1.97E-18 7.74E-18

AP000692.1 0.192446729 0.716232717 1.895969272 2.00E-18 7.84E-18

AL513282.1 0.158483506 0.928888107 2.551172117 2.01E-18 7.90E-18

LINC01700 0.004395725 0.057940299 3.720394119 2.02E-18 7.93E-18

AC002553.1 0.484196907 1.375797734 1.506602616 2.07E-18 8.13E-18

AC012236.1 0.062926022 0.43360171 2.784641791 2.08E-18 8.17E-18

AL360181.2 0.624783126 2.160390777 1.7898649 2.09E-18 8.18E-18

PLG 29.10647671 3.352723678 -3.117934724 2.10E-18 8.24E-18

AC244157.2 0.007734469 0.142313618 4.201627601 2.13E-18 8.33E-18

AP003465.3 0.005740092 0.112474083 4.292375059 2.13E-18 8.33E-18

AP000445.1 0.554602745 2.255348534 2.023823737 2.15E-18 8.41E-18

ABCB4 0.195752971 0.864833787 2.14338868 2.19E-18 8.58E-18

DNMT3L-AS1 0.086228166 0.017238403 -2.322533089 2.20E-18 8.63E-18

CDH4 0.253601562 4.342345418 4.097838956 2.22E-18 8.69E-18

GPR150 0.034626422 0.157508631 2.185485666 2.25E-18 8.80E-18

SCARNA12 0.111366675 2.97258363 4.738327904 2.28E-18 8.91E-18

RPL36AP40 0.175250801 0.040153623 -2.125818953 2.29E-18 8.95E-18

AC005912.1 6.574553694 19.68909981 1.582432285 2.29E-18 8.96E-18

HIGD1B 2.076005817 6.529603948 1.653185002 2.31E-18 9.01E-18

AC104076.1 0.597276423 0.189041662 -1.659694559 2.31E-18 9.04E-18

CD40LG 0.375650774 1.065211627 1.503676102 2.32E-18 9.07E-18

AL136295.2 0.186265177 0.643394003 1.788344508 2.34E-18 9.12E-18

RN7SKP275 0.133887075 0.74990386 2.485688951 2.35E-18 9.19E-18

COL5A1 3.042497317 12.15200668 1.997866672 2.37E-18 9.23E-18

DMRTC1B 0.013245339 0.003712757 -1.834922168 2.37E-18 9.25E-18

AL357514.1 0 0.162253456 Inf 2.44E-18 9.51E-18

SLC5A11 1.551706779 0.198199633 -2.968829765 2.44E-18 9.52E-18

AL031595.3 0.015930212 0.080632875 2.339602684 2.44E-18 9.52E-18

AL121985.1 0.004579094 0.027608519 2.591979409 2.44E-18 9.52E-18

AC011462.4 0.384346072 1.666939175 2.116723632 2.50E-18 9.76E-18

TREH 4.600384207 0.972181992 -2.242456039 2.55E-18 9.94E-18

AP002812.1 0.076203802 0.397663656 2.383613828 2.56E-18 9.96E-18

RRAD 11.97482158 42.68068958 1.833579325 2.57E-18 1.00E-17

TBX5 0.001234323 0.240517073 7.606275203 2.62E-18 1.02E-17

CLRN3 12.60649473 36.52594955 1.534754593 2.63E-18 1.02E-17

AGBL2 0.187472221 0.540246643 1.526941362 2.65E-18 1.03E-17

AC092296.2 0.060690347 0.190474228 1.650056826 2.67E-18 1.04E-17

FOXF2 0.115490241 0.373069307 1.691672721 2.67E-18 1.04E-17

AC023813.3 0.048855156 0.186013586 1.928825271 2.67E-18 1.04E-17

AC005306.1 0.060826949 0.360572816 2.567508089 2.68E-18 1.04E-17

CCDC188 0.138070449 0.473324341 1.777424537 2.68E-18 1.04E-17

PRSS37 0.013774434 0.10543793 2.936329056 2.74E-18 1.07E-17

AC092353.1 0.096792377 0.026391574 -1.874816029 2.74E-18 1.07E-17

AC104031.1 1.315596706 6.273398718 2.253529958 2.77E-18 1.07E-17

AC026362.1 0.029691597 0.098044223 1.723377951 2.80E-18 1.09E-17

PFN1P2 0.085580625 0.299502024 1.807209641 2.82E-18 1.09E-17

WNT1 0.01070319 0.062131061 2.537273837 2.89E-18 1.12E-17

HMGB1P3 0.034837253 0.16420285 2.236776393 2.92E-18 1.13E-17

FAM186A 0.024720263 0.087445015 1.822682068 2.95E-18 1.14E-17

AL357874.1 0.067580107 0.228662368 1.758548421 2.96E-18 1.15E-17

FBXO41 0.301115941 1.192639593 1.985767144 3.00E-18 1.16E-17

SLFN14 0.006462419 0.041372799 2.678536309 3.03E-18 1.17E-17

FABP1 26.40938491 1.126210216 -4.551502653 3.06E-18 1.18E-17

AC114316.1 0.029381612 0.339201182 3.529155736 3.10E-18 1.20E-17

C1QL1 2.116853707 26.62247187 3.652651055 3.12E-18 1.21E-17

LINC02390 0.012688221 0.099747442 2.974790015 3.12E-18 1.21E-17

AL356364.1 1.430962038 0.107294211 -3.73734125 3.18E-18 1.23E-17

IMPDH1P10 0.013093505 0.067369601 2.363246451 3.33E-18 1.29E-17

PPL 14.98075406 5.063256254 -1.564972837 3.38E-18 1.30E-17

TRBV15 0.047038241 0.521969299 3.472058939 3.47E-18 1.34E-17

KIAA0408 0.006198697 0.028815892 2.216827816 3.51E-18 1.35E-17

AZU1 0.024325141 0.106283378 2.127395916 3.51E-18 1.35E-17

AC007347.1 0.069902336 0.446118619 2.674014787 3.59E-18 1.38E-17

NODAL 0.042646224 0.132608872 1.636687379 3.60E-18 1.39E-17

KL 53.4211564 18.70037734 -1.514343825 3.60E-18 1.39E-17

AL021407.1 0.029023622 0.230397698 2.98882682 3.62E-18 1.39E-17

PDE6C 0.050592255 0.175264644 1.79254655 3.69E-18 1.42E-17

AC093063.1 0.006020191 0.445473202 6.209385683 3.70E-18 1.42E-17

AC140125.2 0.01481989 0.111503681 2.91148469 3.72E-18 1.43E-17

RNY4P10 0.552204486 2.401061084 2.120397593 3.72E-18 1.43E-17

AC079946.1 0.31823724 0.067510277 -2.236923624 3.73E-18 1.43E-17

AC005332.2 0.068232873 0.203580474 1.577060317 3.79E-18 1.45E-17

HNRNPA1P17 0.004649368 0.105173199 4.499588621 3.94E-18 1.51E-17

CEBPE 0.029887527 0.085686515 1.519524641 3.97E-18 1.52E-17

AP003100.1 0.002882561 0.113065644 5.293665616 3.99E-18 1.53E-17

VWA3B 0.087120348 0.019405799 -2.166521868 4.03E-18 1.55E-17

AL645608.7 0.422838843 1.821948996 2.107302755 4.11E-18 1.58E-17

TRAV9-2 0.147797347 0.720451599 2.285281142 4.12E-18 1.58E-17

AC018737.2 0.006922734 0.047683882 2.784087794 4.21E-18 1.61E-17

LGALS9C 0.006382312 0.021259748 1.735973404 4.21E-18 1.61E-17

LINC02421 0.009824952 0.041975094 2.095011259 4.22E-18 1.62E-17

KIF4B 0.004553018 0.024364666 2.419895478 4.23E-18 1.62E-17

RNU6-226P 0.02020744 0.437624079 4.436733633 4.31E-18 1.65E-17

HECW2 1.392989002 3.988837624 1.517784529 4.34E-18 1.66E-17

AC007406.2 2.072276318 8.050849708 1.957924672 4.38E-18 1.68E-17

AC234782.2 0.015179452 0.096300345 2.66542124 4.44E-18 1.70E-17

AL049776.1 0.143580238 0.408791446 1.50950781 4.48E-18 1.71E-17

AC010531.5 0.043424322 0.244724432 2.49458291 4.56E-18 1.74E-17

AGAP5 0.032156782 0.111755999 1.797157315 4.64E-18 1.77E-17

AP000873.4 0.13111748 0.506492122 1.949679793 4.73E-18 1.80E-17

TRPM6 1.109150587 0.148155525 -2.904270921 4.77E-18 1.82E-17

EDN1 9.967852014 34.23102498 1.779949937 4.86E-18 1.85E-17

AC005899.9 0.012678261 0.072525942 2.51614023 4.88E-18 1.86E-17

PDCL3P5 0.114453043 0.350743695 1.615661349 4.89E-18 1.86E-17

AC064805.1 0.033248421 0.106677134 1.681893252 4.90E-18 1.87E-17

DNAJB13 0.087978912 1.182662321 3.748736642 4.92E-18 1.88E-17

AC006262.2 0.009043203 0.193929848 4.422557264 4.95E-18 1.89E-17

AP000344.1 1.008316956 0.21050915 -2.259994364 4.99E-18 1.90E-17

AC011479.3 0.052126083 0.2685893 2.365324482 5.12E-18 1.95E-17

AC090001.1 0.193384971 0.035806239 -2.433192767 5.12E-18 1.95E-17

CIB4 0.204928942 1.183853107 2.530294427 5.19E-18 1.98E-17

CSNK1G2-AS1 0.011943984 0.076410037 2.677478014 5.21E-18 1.98E-17

SERPINE3 0.049503086 0.141108368 1.511213169 5.24E-18 1.99E-17

GBP1P1 0.7993546 2.322642102 1.538859324 5.24E-18 1.99E-17

AC004034.1 0.060675346 0.286162741 2.237653508 5.28E-18 2.01E-17

EML4-AS1 0.031132087 0.125405501 2.01012644 5.39E-18 2.05E-17

AC007362.1 0.007462974 0.093055816 3.640273857 5.40E-18 2.05E-17

TRGV3 0.136668263 0.466451619 1.771049186 5.45E-18 2.07E-17

AL354836.1 1.377484261 4.828557104 1.809556304 5.64E-18 2.14E-17

LINC01827 0.016488492 0.15559669 3.238280001 5.72E-18 2.17E-17

AC136475.3 0.856646009 13.48917833 3.976959498 5.75E-18 2.18E-17

GPR143P 0.169413932 0.026974571 -2.650880583 5.77E-18 2.19E-17

AC005757.1 0.004077011 0.104003602 4.672977942 5.78E-18 2.19E-17

CDSN 0.004506629 0.037250694 3.047146616 5.83E-18 2.21E-17

AC011455.2 0.005009292 0.037650095 2.90997489 5.87E-18 2.22E-17

CACNA1F 0.062106309 0.302419122 2.283737639 5.93E-18 2.25E-17

RPL36AP43 0.239485396 0.856996862 1.839352237 5.97E-18 2.26E-17

LINC01501 0.003366316 0.040611057 3.592630262 6.01E-18 2.28E-17

GSDMB 0.363560933 1.345897775 1.888299752 6.04E-18 2.29E-17

CACNA1C-AS1 0.03225456 0.113371502 1.813483003 6.17E-18 2.34E-17

AC021146.1 0.048532821 0.70549143 3.861595921 6.25E-18 2.36E-17

TRBV5-4 0.135794445 0.662669958 2.286866049 6.29E-18 2.38E-17

AC244093.5 0.034704589 0.15740405 2.181274307 6.34E-18 2.40E-17

SPDEF 0.069527114 0.433682778 2.640992545 6.35E-18 2.40E-17

VCAN 7.009032463 25.42183894 1.858781183 6.47E-18 2.44E-17

AC073621.2 0.033264117 0.13904001 2.06346145 6.51E-18 2.46E-17

BX640515.1 0.000649559 0.069450437 6.740380257 6.55E-18 2.47E-17

NTN5 0.043152295 0.163295741 1.919977966 6.55E-18 2.47E-17

FIBCD1 0.045066528 0.926502771 4.361667066 6.71E-18 2.53E-17

AC018410.1 0.078971944 0.347141635 2.136112301 6.72E-18 2.53E-17

TSGA10IP 0.013566488 0.059569521 2.134527109 6.76E-18 2.55E-17

AC010894.5 0.009949443 0.066127737 2.732567792 6.77E-18 2.56E-17

CYP4B1 0.883420445 0.298382399 -1.565937787 6.80E-18 2.56E-17

AC067852.3 0.167249728 0.609204143 1.864921886 6.88E-18 2.59E-17

SLC5A5 0.008798098 0.072965847 3.051957731 6.89E-18 2.59E-17

AC007566.1 0.641806218 2.09545086 1.707051018 6.93E-18 2.61E-17

AC104564.3 0.177312788 0.780916822 2.138872305 7.16E-18 2.70E-17

AC109361.1 0.010524556 0.04380736 2.057413915 7.20E-18 2.71E-17

INHBE 0.114432542 1.186352414 3.373963352 7.23E-18 2.72E-17

AP000763.3 0.034723134 0.245492232 2.821708286 7.35E-18 2.76E-17

LINC01730 0.055357133 0.26712062 2.270650227 7.35E-18 2.77E-17

AL606534.1 0.058091993 0.225937647 1.959513438 7.38E-18 2.77E-17

NKX6-2 0.035264526 0.004481649 -2.976116042 7.41E-18 2.79E-17

CD38 0.36920732 1.385397938 1.907797369 7.46E-18 2.80E-17

AL033397.2 0.065993166 0.291547895 2.143344378 7.60E-18 2.85E-17

CCNYL2 0.083645897 0.620798665 2.891758779 7.70E-18 2.89E-17

PRSS30P 0.057992751 0.170374046 1.554761105 7.75E-18 2.91E-17

AC092653.1 0.136184582 0.387262806 1.507749571 7.96E-18 2.99E-17

AC025766.1 0.122195837 0.545379563 2.158065508 8.07E-18 3.03E-17

LINC02345 0.031340214 0.113553431 1.857284372 8.19E-18 3.07E-17

AC079336.5 0.031068549 0.135043201 2.119894222 8.39E-18 3.14E-17

AC120042.1 0.118092733 0.020160961 -2.550283843 8.51E-18 3.19E-17

CTRC 0.010162027 0.049785035 2.292523942 8.57E-18 3.21E-17

AC006557.1 0.017230378 0.084897384 2.300765768 8.65E-18 3.24E-17

FGF12-AS2 0.024005337 0.138150258 2.52481116 8.70E-18 3.25E-17

LINC02604 0.866312332 2.634192653 1.604401702 8.71E-18 3.26E-17

CCL3 1.57382809 5.668468198 1.848680963 8.77E-18 3.28E-17

AC105021.1 0.066250209 0.020115768 -1.719598187 8.82E-18 3.30E-17

DNMT3L 0.177691297 0.025208815 -2.817372816 8.98E-18 3.36E-17

AC079380.1 0.052990525 0.233624699 2.140386476 8.99E-18 3.36E-17

GPS2 0.650231104 1.854846437 1.512275276 9.15E-18 3.42E-17

CA15P1 0.039478989 0.185978683 2.235980325 9.21E-18 3.44E-17

AL162430.3 0.068273418 0.307635553 2.171826349 9.24E-18 3.45E-17

KLRC4 0.032420423 0.172061396 2.407948644 9.61E-18 3.58E-17

AC099552.1 0.175357848 0.009456634 -4.212831452 9.83E-18 3.66E-17

AC012676.5 0.246254406 0.869672326 1.820322391 9.92E-18 3.69E-17

AL359378.1 0.033469392 0.011382818 -1.555984568 1.01E-17 3.77E-17

PRND 0.17529766 1.657114571 3.240794707 1.02E-17 3.78E-17

AC084783.1 0.004785108 0.082532238 4.108334227 1.02E-17 3.79E-17

CENPUP2 0.010247205 0.097024877 3.24312428 1.03E-17 3.83E-17

HDC 0.093007408 0.349956859 1.911759548 1.06E-17 3.92E-17

LINC01928 0.663338219 0.058846115 -3.494725583 1.09E-17 4.04E-17

AP000812.1 0.081465681 0.28189464 1.790891727 1.10E-17 4.08E-17

AC004836.1 0.1572689 0.663780891 2.077473693 1.10E-17 4.08E-17

RNA5SP498 0.109179076 1.070315845 3.29326829 1.10E-17 4.10E-17

AC021945.1 0.014354455 0.068757177 2.260011775 1.11E-17 4.12E-17

HSPA8P4 0.024996855 0.083239598 1.735523376 1.14E-17 4.24E-17

AC009120.2 0.521049923 1.716232632 1.719751609 1.15E-17 4.27E-17

SLC36A2 16.97225293 0.714666882 -4.569763338 1.16E-17 4.29E-17

MCEMP1 0.074701727 0.334087127 2.161010899 1.16E-17 4.29E-17

SIGLEC12 0.06434534 0.395776131 2.620777027 1.16E-17 4.30E-17

LINC00996 0.069649995 0.226331137 1.700239918 1.16E-17 4.32E-17

SLAMF1 0.249723053 0.722332122 1.53233332 1.17E-17 4.34E-17

AC008982.2 0.147223165 0.604220638 2.037070767 1.18E-17 4.36E-17

AC100830.2 0.236398046 0.99661949 2.075824676 1.22E-17 4.52E-17

Z97200.1 0.014978465 0.173613535 3.534917774 1.22E-17 4.53E-17

AQP8 0.011493006 0.077771778 2.758490582 1.24E-17 4.59E-17

AC092813.2 3.771409921 0.115020279 -5.035143815 1.24E-17 4.59E-17

LINC00174 0.635956639 1.856050674 1.545235792 1.25E-17 4.61E-17

ACTG1P17 0.078803524 0.232229241 1.559217585 1.26E-17 4.67E-17

LINC00410 0.075631852 0.00935892 -3.01458002 1.29E-17 4.76E-17

AL590004.3 0.045680603 0.341215207 2.901028337 1.32E-17 4.87E-17

AC018648.1 0.059203504 0.25742098 2.12037518 1.33E-17 4.92E-17

RPL12P10 0.056432751 0.444107923 2.976305725 1.34E-17 4.94E-17

RPL4P3 0.066248092 0.364626201 2.460467421 1.35E-17 5.00E-17

AP003484.1 0.016846087 0.290150652 4.106316712 1.37E-17 5.06E-17

NARF-IT1 0.09580311 0.423671537 2.144801808 1.37E-17 5.07E-17

FGF20 0.031710593 0.140403702 2.146544222 1.38E-17 5.08E-17

P2RX5 0.052175432 0.199626917 1.935863715 1.38E-17 5.10E-17

AL731556.1 0.015529527 0.102690587 2.725218109 1.39E-17 5.13E-17

DNHD1 0.214978321 0.669063306 1.637951544 1.39E-17 5.13E-17

SPDYE5 0.062982417 0.212024265 1.751208351 1.39E-17 5.13E-17

RN7SL535P 0.060859746 0.313521606 2.365004656 1.45E-17 5.36E-17

AC138409.2 0.083065689 0.266853049 1.683720914 1.46E-17 5.39E-17

AC004148.1 0.510152588 1.863830991 1.869270313 1.47E-17 5.42E-17

AC138409.1 0.01342604 0.107998485 3.007905274 1.50E-17 5.50E-17

AL136380.1 0.1362943 0.803176303 2.558991476 1.53E-17 5.64E-17

TRAV27 0.080017135 0.826501118 3.368635894 1.54E-17 5.68E-17

SPDYA 0.075396623 0.249306077 1.725346245 1.64E-17 6.04E-17

DDN 8.607555135 0.110987796 -6.277130564 1.65E-17 6.05E-17

CX3CR1 2.027509138 6.70631854 1.725812594 1.66E-17 6.11E-17

AC012645.4 0.058530354 0.338718391 2.532829414 1.68E-17 6.18E-17

AP003096.1 0.059404579 0.194981782 1.714693274 1.68E-17 6.18E-17

LINC00051 0.119957001 0.015833112 -2.92150059 1.70E-17 6.24E-17

AC006272.2 0.156066015 0.563393263 1.851985905 1.72E-17 6.32E-17

AC027796.4 0.224249392 1.066627439 2.249880365 1.74E-17 6.37E-17

H2BW3P 0.025784802 0.122504962 2.248247342 1.76E-17 6.44E-17

AL161669.1 0.218977187 1.05455292 2.267779015 1.76E-17 6.44E-17

AC084026.2 0.02925416 0.180751413 2.62729324 1.76E-17 6.46E-17

PADI3 0.125578809 1.037830567 3.046905992 1.82E-17 6.68E-17

AC005532.2 0.05299806 0.448296202 3.080440823 1.83E-17 6.71E-17

MIR324 0.104733558 0.788007903 2.911486331 1.85E-17 6.78E-17

SNORA71B 0.209083912 0.889761927 2.089337311 1.96E-17 7.17E-17

AC006160.1 0.112001637 0.393050196 1.811193751 1.99E-17 7.26E-17

AC012213.3 0.060651819 0.213201401 1.813594105 1.99E-17 7.27E-17

AL357078.2 0.043064464 0.264383725 2.618063582 2.08E-17 7.59E-17

LILRB5 0.319792113 1.086771141 1.764841898 2.09E-17 7.62E-17

AC034236.3 0.014365977 0.122883354 3.09656146 2.12E-17 7.74E-17

AC093732.2 0.005670026 0.120906144 4.41438848 2.16E-17 7.87E-17

AC093797.1 0.130756709 0.436611166 1.739464049 2.20E-17 8.04E-17

CST6 1.902074173 0.670825225 -1.503564663 2.22E-17 8.09E-17

H4C11 0.050470749 0.244289287 2.275071192 2.22E-17 8.09E-17

LINC01659 0.765055267 0.16044609 -2.253475342 2.26E-17 8.23E-17

LINC01359 0.064567435 0.292444003 2.179281787 2.26E-17 8.23E-17

LINC00896 0.010682984 0.101190566 3.243688187 2.27E-17 8.28E-17

AC138969.1 0.020431601 0.067002867 1.71342055 2.27E-17 8.28E-17

AC099684.1 0.257866906 0.039017949 -2.72441679 2.27E-17 8.29E-17

AC008267.3 0.293571544 0.947909182 1.691036707 2.29E-17 8.33E-17

AC063965.2 0.111036948 0.705899427 2.668422829 2.30E-17 8.39E-17

RN7SKP70 0.440695764 1.528459802 1.794223674 2.31E-17 8.42E-17

AC004890.3 0.049373396 0.258951777 2.390877674 2.34E-17 8.51E-17

AC025580.2 0.016518958 0.048521076 1.554488828 2.36E-17 8.57E-17

AC104964.4 0.191484267 0.818304624 2.095412145 2.36E-17 8.58E-17

LINC01513 0.038108275 0.174722374 2.196888146 2.39E-17 8.70E-17

ARL4C 8.339481043 24.57319224 1.559055772 2.40E-17 8.73E-17

AL022323.3 0.010352248 0.08093825 2.966877564 2.40E-17 8.73E-17

LDHAP4 1.612061846 4.581599986 1.506944411 2.42E-17 8.78E-17

FAIM2 0.630381083 0.202041075 -1.641575621 2.46E-17 8.94E-17

MIR25 0.760879156 3.101571785 2.027260271 2.47E-17 8.99E-17

CCDC116 0.030575547 0.099638966 1.704331731 2.54E-17 9.21E-17

AL662884.1 0.034127644 0.174125957 2.351118559 2.54E-17 9.23E-17

AL133371.3 0.025706536 0.192975654 2.908211733 2.55E-17 9.25E-17

AC008264.2 0.463694801 0.072262361 -2.681859244 2.57E-17 9.32E-17

AC113189.4 0.005869167 0.020204795 1.783470018 2.60E-17 9.43E-17

AC007787.2 0.026279433 0.148668977 2.500097579 2.61E-17 9.47E-17

AL035446.1 0.139378666 0.699667281 2.32765928 2.69E-17 9.76E-17

AL645939.3 0.050749796 0.332269615 2.710880437 2.69E-17 9.76E-17

SLC26A10 0.019480796 0.139510446 2.840248628 2.72E-17 9.84E-17

AC138028.4 0.342256766 1.258070924 1.878062287 2.75E-17 9.95E-17

KHDRBS2 0.244053756 0.067649435 -1.851049167 2.88E-17 1.04E-16

TPSB2 1.704389531 5.54143091 1.701003463 2.88E-17 1.04E-16

AC009095.1 0.06693367 0.223331941 1.73838558 2.95E-17 1.07E-16

AC008897.1 0.291317218 0.872613272 1.582751442 2.95E-17 1.07E-16

AL022328.2 0.640800372 2.063638931 1.687243678 2.97E-17 1.07E-16

KIAA1324 0.127750022 0.454819651 1.831971046 3.03E-17 1.09E-16

AC104837.2 0.02900484 0.237613179 3.0342493 3.06E-17 1.11E-16

MAMDC4 0.855645818 2.995246688 1.807589185 3.06E-17 1.11E-16

C3orf85 1.672702609 0.173523796 -3.268975544 3.10E-17 1.12E-16

P2RY12 0.478947898 1.637549988 1.773598319 3.12E-17 1.13E-16

PICSAR 0.019409076 0.158732758 3.031796533 3.13E-17 1.13E-16

AP002770.2 0.048160339 0.188738266 1.97046951 3.14E-17 1.13E-16

AC007497.1 0.072972847 0.271837423 1.897312429 3.19E-17 1.15E-16

AC007598.3 0.020382645 0.158883218 2.962553571 3.20E-17 1.15E-16

AC010261.1 0.041590909 0.332178171 2.997617159 3.21E-17 1.16E-16

AC127024.4 0.250425028 1.055792991 2.075876332 3.21E-17 1.16E-16

AP001029.1 0.054674511 0.544214524 3.315235132 3.22E-17 1.16E-16

AC092162.2 0.035567866 0.137079404 1.946365483 3.22E-17 1.16E-16

AC066613.1 0.195761845 0.597887761 1.610775071 3.26E-17 1.17E-16

AL096711.2 0.013031717 0.040411983 1.632755994 3.32E-17 1.19E-16

BTG1P1 0.058069398 0.228322871 1.975225389 3.35E-17 1.21E-16

LHX8 0.003195502 0.185485289 5.859118247 3.36E-17 1.21E-16

RN7SL659P 0.014656278 0.159025304 3.439665629 3.37E-17 1.21E-16

IGF2BP3 0.04052223 0.327299929 3.013827801 3.38E-17 1.21E-16

NLGN1-AS1 0.003573108 0.297364789 6.378910362 3.51E-17 1.26E-16

LINC02516 0.012968994 0.06779141 2.386035922 3.52E-17 1.27E-16

AC122710.2 0.011648667 0.108998156 3.226066939 3.56E-17 1.28E-16

ATP5MC1P4 0.079867404 0.33561703 2.071137203 3.57E-17 1.28E-16

AC009974.1 0.149144972 0.553979095 1.893116198 3.59E-17 1.29E-16

AC016949.1 0.090029061 0.343306084 1.931032742 3.61E-17 1.30E-16

ASIC4 0.006416118 0.032080412 2.321920175 3.62E-17 1.30E-16

AP000919.2 0.01634306 0.188297578 3.526264444 3.65E-17 1.31E-16

SNORA71A 0.204155151 2.271975998 3.476209723 3.65E-17 1.31E-16

TREM1 0.2352162 1.03368845 2.135742099 3.77E-17 1.35E-16

AC006378.1 0.064806349 0.224302831 1.791240758 3.92E-17 1.40E-16

AC026403.1 1.777309651 6.045366115 1.766134657 3.95E-17 1.42E-16

NAPSB 2.800682515 9.098204151 1.699803358 4.08E-17 1.46E-16

FPR1 1.842032599 5.525870812 1.584903241 4.13E-17 1.48E-16

TRPC2 0.063280854 0.912948132 3.850691929 4.18E-17 1.49E-16

AC000078.1 0.103484055 0.400117095 1.951013777 4.23E-17 1.51E-16

CYP4F2 12.57277723 0.40139887 -4.969122998 4.28E-17 1.53E-16

AC008667.1 0.047834265 0.261689384 2.451739078 4.44E-17 1.58E-16

SPRR2D 0.055232481 0.017905478 -1.625115859 4.46E-17 1.59E-16

LINC01779 0.054022308 0.017547632 -1.622278955 4.51E-17 1.61E-16

RN7SL81P 0.124909661 0.595919181 2.254231616 4.58E-17 1.63E-16

AC063977.2 0.028485689 0.158383499 2.475112841 4.62E-17 1.65E-16

RN7SL113P 0.050338546 0.307341317 2.610106278 4.69E-17 1.67E-16

AL109806.1 0.020184212 0.091467309 2.18002891 4.71E-17 1.68E-16

AC008957.1 0.08807267 0.327478725 1.894634876 4.74E-17 1.69E-16

AC092821.2 0.017385539 0.056171128 1.691941011 4.76E-17 1.69E-16

TRBV7-6 0.097191294 0.779281389 3.003245367 4.79E-17 1.71E-16

LINC01433 0.012945448 0.086313071 2.737134131 4.94E-17 1.76E-16

SLC2A5 5.262929739 18.04480087 1.777645183 4.95E-17 1.76E-16

SNORD60 0.165953001 1.068878575 2.687251344 4.97E-17 1.77E-16

EEF1A1P31 0.01310151 0.099061737 2.918594781 5.02E-17 1.79E-16

GCNA 0.131661904 0.398376327 1.59729395 5.04E-17 1.79E-16

AC000120.2 0.060980182 0.313825537 2.363550393 5.11E-17 1.82E-16

SNORA71C 0.189753187 1.043018488 2.45856871 5.12E-17 1.82E-16

GMNC 1.494253624 0.132791998 -3.492184927 5.15E-17 1.83E-16

AMZ2P2 0.003532044 0.06799637 4.266882391 5.19E-17 1.84E-16

DIRC3 0.148503618 0.480643752 1.694469902 5.26E-17 1.87E-16

AL031963.2 0.03848798 0.172313736 2.162557866 5.34E-17 1.90E-16

AL021707.7 0.123280223 0.576657021 2.225772127 5.35E-17 1.90E-16

AC012181.1 0.453947644 1.343302473 1.565186374 5.35E-17 1.90E-16

PCDHGB9P 0.045224241 0.14049514 1.635352022 5.42E-17 1.92E-16

AC004623.1 0.036374501 0.148261004 2.027139831 5.48E-17 1.94E-16

AC139100.2 0.276792594 0.989477556 1.83786164 5.48E-17 1.94E-16

PITX3 0.06749454 0.021034872 -1.681987752 5.49E-17 1.95E-16

AMH 0.060116315 0.39390047 2.712002652 5.55E-17 1.96E-16

AL512378.1 0.002573306 0.042284515 4.038434641 5.64E-17 2.00E-16

PRLR 5.089004043 1.480109057 -1.781679857 5.65E-17 2.00E-16

AP005131.6 0.035820858 0.188753659 2.397632823 5.69E-17 2.01E-16

MIR548AN 0.081081994 0.693958818 3.09739657 5.78E-17 2.04E-16

ASCL5 0.031496327 0.117904523 1.904363559 5.79E-17 2.05E-16

SPON2 4.512449071 14.75412186 1.709135502 5.89E-17 2.08E-16

AL354751.2 0.011080791 0.284266973 4.681113772 5.95E-17 2.10E-16

AC010487.1 0.181014186 0.798013912 2.140311137 5.98E-17 2.11E-16

AC079684.2 0.171040217 0.581492446 1.765424861 6.02E-17 2.13E-16

TRBV12-3 0.085789612 0.472627531 2.461828806 6.04E-17 2.13E-16

VWFP1 0.13309167 0.854219307 2.682186226 6.04E-17 2.13E-16

AC087163.3 0.077597036 0.280426138 1.853547371 6.20E-17 2.19E-16

AC005332.7 0.130386864 0.394477518 1.597144549 6.28E-17 2.21E-16

TRBV27 0.081946518 0.658507945 3.006446299 6.31E-17 2.23E-16

AC239859.5 0.662986087 2.607382667 1.975551833 6.32E-17 2.23E-16

AL589993.1 0.0173223 0.066981908 1.951140964 6.38E-17 2.25E-16

PRSS51 0.113934046 0.459849657 2.012963347 6.53E-17 2.30E-16

AFAP1-AS1 0.028427279 0.416487594 3.872925585 6.57E-17 2.31E-16

U47924.3 0.101494313 0.437019335 2.106298217 6.76E-17 2.38E-16

AL139349.1 0.288177374 1.652000911 2.519185511 6.87E-17 2.42E-16

GOLGA8A 0.516654217 3.861288915 2.901811555 6.98E-17 2.45E-16

LINC01234 0.038619992 0.504905611 3.708593934 6.98E-17 2.45E-16

IGBP1-AS1 0.096276568 0.377840418 1.97252042 7.08E-17 2.49E-16

AC104806.2 0.021918952 0.112480847 2.359428658 7.08E-17 2.49E-16

AC138956.1 0.2669516 0.903600354 1.759106639 7.10E-17 2.50E-16

AC092681.2 0.026505402 0.088935513 1.746473213 7.15E-17 2.51E-16

AC138956.2 0.50321061 1.650622201 1.713775705 7.23E-17 2.54E-16

UGT1A1 0.019376902 0.12012843 2.6321678 7.31E-17 2.57E-16

AC131888.1 0.050553968 0.147572274 1.545525468 7.36E-17 2.58E-16

AL353588.1 0.020099624 0.073531031 1.871184704 7.58E-17 2.66E-16

MIR6797 0.123608046 0.75477466 2.610273334 7.58E-17 2.66E-16

AL135999.1 0.211246046 0.893068678 2.079846786 7.64E-17 2.68E-16

AP005131.2 0.084327603 0.345269043 2.033644142 7.64E-17 2.68E-16

AC073548.1 0.229846181 0.907966817 1.981970875 7.68E-17 2.69E-16

AL450326.2 0.02820349 0.123675364 2.132612565 7.69E-17 2.70E-16

CELF2-AS1 0.025955065 0.163214382 2.652680207 7.91E-17 2.77E-16

IRF4 0.192470378 0.781507344 2.021623002 8.01E-17 2.81E-16

AC073370.1 0.019708884 0.107973705 2.45376199 8.05E-17 2.82E-16

AC036108.2 0.026727964 0.155694642 2.542297431 8.05E-17 2.82E-16

C10orf105 0.012303183 0.035964653 1.547548123 8.17E-17 2.86E-16

AP001625.3 0.043532106 0.012045313 -1.853607924 8.20E-17 2.87E-16

ERVK9-11 0.70537211 2.369300617 1.748004822 8.26E-17 2.89E-16

STX16-NPEPL1 0.07910886 0.336039759 2.086720745 8.26E-17 2.89E-16

AC119751.6 0.015862134 0.325389335 4.358508181 8.34E-17 2.92E-16

AL450998.3 0.095521077 0.332924604 1.801304484 8.38E-17 2.93E-16

RPL37P1 0.019256936 0.229082903 3.572419703 8.39E-17 2.93E-16

TRBV14 0.083606474 0.464931186 2.475330631 8.50E-17 2.97E-16

CA6 0.002099154 0.017620536 3.069378094 8.53E-17 2.98E-16

AC020658.5 0.136235651 0.421622932 1.629849046 8.67E-17 3.03E-16

AGAP6 0.564174238 1.745870308 1.629733699 8.87E-17 3.10E-16

RPL4P1 0.034111139 0.132256398 1.955022669 9.01E-17 3.14E-16

AL162457.1 0.004533185 0.04168865 3.201057858 9.09E-17 3.17E-16

TH2LCRR 0.05910671 0.250700743 2.08457245 9.11E-17 3.18E-16

MIR6819 0.131684028 1.098284751 3.060099875 9.24E-17 3.22E-16

AC141557.1 0.212571125 0.972681117 2.194021273 9.27E-17 3.23E-16

AC090282.1 0.002641293 0.036436651 3.786074319 9.50E-17 3.31E-16

LINC00893 0.168802925 0.800961054 2.246392189 9.65E-17 3.36E-16

AC087627.1 0.018249593 0.107840816 2.562967144 9.76E-17 3.40E-16

LENG8 7.999182653 24.47658098 1.613477549 1.01E-16 3.52E-16

AC073869.1 0.052973365 0.240861785 2.184866461 1.02E-16 3.57E-16

PCSK6-AS1 0.002299507 0.132464729 5.848139731 1.03E-16 3.57E-16

LINC02363 0.030120822 0.166439991 2.466169064 1.03E-16 3.59E-16

NPIPB14P 0.057734765 0.22349632 1.952738882 1.04E-16 3.63E-16

AL139041.1 0.042387273 0.207514468 2.291508859 1.05E-16 3.67E-16

ZKSCAN2-DT 0.240281043 0.857670623 1.835700873 1.06E-16 3.67E-16

AL008727.1 0.005055629 0.024065302 2.250992156 1.06E-16 3.67E-16

AC092436.3 0.009227091 0.138819655 3.911192157 1.06E-16 3.69E-16

C3orf35 0.059995698 0.195945757 1.707523365 1.06E-16 3.69E-16

LINC02119 0.012274494 0.002747763 -2.159333921 1.07E-16 3.72E-16

VSIG4 5.905771228 18.19610974 1.623432663 1.08E-16 3.75E-16

LINC02577 0.007771186 0.485712515 5.965824067 1.09E-16 3.78E-16

AC044849.1 0.096537564 0.312691086 1.69557576 1.10E-16 3.83E-16

AL365209.1 0.007334432 0.041182107 2.489260591 1.12E-16 3.88E-16

LINC02154 0.023894145 0.33760518 3.820607981 1.13E-16 3.91E-16

LINC02804 0.069057336 0.306794199 2.151404628 1.14E-16 3.96E-16

AL109955.1 0.036888204 0.115124813 1.641967347 1.17E-16 4.04E-16

AL359182.1 0.148687713 0.472457208 1.667898235 1.18E-16 4.09E-16

DHFRP2 0.005700251 0.122572152 4.42646203 1.18E-16 4.09E-16

AL355488.1 0.557591263 1.825068095 1.710670434 1.18E-16 4.10E-16

TPM1-AS 0.146790096 0.472097363 1.68532979 1.26E-16 4.34E-16

ANKRD33 0.005335347 0.051374523 3.267399044 1.26E-16 4.36E-16

TRAV1-2 0.166123721 0.76676314 2.206522893 1.26E-16 4.37E-16

LINC02765 0.053242168 0.169648863 1.671910542 1.27E-16 4.38E-16

SNORA75 0.166460722 1.0910353 2.71244408 1.27E-16 4.39E-16

LINC02802 0.382981501 1.086455469 1.504282432 1.28E-16 4.42E-16

RPL39P38 0.237425852 0.798983069 1.750687901 1.29E-16 4.44E-16

PAIP1P1 0.099654756 0.353393898 1.82626657 1.30E-16 4.48E-16

RUSC1-AS1 0.482382394 1.460941908 1.598649654 1.31E-16 4.52E-16

CAMK2A 0.37374992 0.062435568 -2.581633241 1.32E-16 4.57E-16

ZNF826P 3.213913482 9.879239535 1.620068901 1.32E-16 4.57E-16

TRAV25 0.071733493 0.478806804 2.738724876 1.34E-16 4.62E-16

GOLGA8M 0.063319622 0.265518763 2.068089267 1.36E-16 4.68E-16

AC004000.1 0.040751675 0.331218312 3.022851188 1.36E-16 4.68E-16

CYP2D7 0.061098965 0.365046052 2.578858628 1.39E-16 4.80E-16

MIR23C 0.026604671 0.886837322 5.058918027 1.41E-16 4.87E-16

AC018682.1 0.076464423 0.295166162 1.948666777 1.41E-16 4.87E-16

AC245884.10 0.030319894 0.239226194 2.980038757 1.42E-16 4.90E-16

KIAA1210 0.106991545 0.023802215 -2.168329035 1.42E-16 4.91E-16

LDHAP3 0.205788991 0.725188683 1.817190604 1.44E-16 4.94E-16

AP000844.2 0.026764018 0.587040146 4.455092555 1.45E-16 4.98E-16

AC091564.1 0.049896461 0.246097576 2.302221041 1.45E-16 5.00E-16

AC122719.1 0.022557583 0.325929803 3.852876856 1.48E-16 5.10E-16

YBX2P1 0.003588169 0.057851504 4.011034593 1.48E-16 5.10E-16

AC005722.3 0.44241656 0.094099545 -2.233145733 1.49E-16 5.13E-16

AC009495.2 0.078292324 0.441091062 2.494133757 1.51E-16 5.20E-16

AC009119.2 0.062631439 0.314896319 2.329917963 1.53E-16 5.25E-16

AC098828.1 0.370248663 0.085603873 -2.112746555 1.57E-16 5.40E-16

RPL35AP32 0.068402578 0.395546606 2.531725079 1.58E-16 5.44E-16

AP001363.1 0.087840368 0.533056 2.601331108 1.60E-16 5.48E-16

AC099786.2 0.001092304 0.11248565 6.686223109 1.60E-16 5.48E-16

LINC02067 0.004515601 0.042616222 3.238412668 1.60E-16 5.50E-16

AC011375.1 0.053255723 0.361258459 2.762022902 1.61E-16 5.51E-16

AC009120.1 0.730694297 2.358117531 1.690295773 1.61E-16 5.53E-16

OTOG 0.008597775 0.041974571 2.287480264 1.63E-16 5.58E-16

RN7SL608P 0.362101949 1.33118521 1.878243463 1.63E-16 5.59E-16

CHST6 0.374786808 0.055354491 -2.75929789 1.67E-16 5.73E-16

CKM 0.902833927 0.129967094 -2.796314232 1.68E-16 5.75E-16

AQP9 0.912599834 4.128871543 2.177693238 1.69E-16 5.80E-16

RNA5SP383 0.359829795 1.768077902 2.296795285 1.70E-16 5.83E-16

KRT18P5 0.044830705 0.144000684 1.683516562 1.72E-16 5.89E-16

AC008033.3 0.134335614 0.963922257 2.843074965 1.73E-16 5.93E-16

AC005154.4 0.110775601 0.419301689 1.920348488 1.74E-16 5.96E-16

AL136295.5 0.03769532 0.127014686 1.752538002 1.76E-16 6.03E-16

SNORA73B 0.64666156 98.86331895 7.256280675 1.77E-16 6.05E-16

SNHG25 0.347610737 1.310574896 1.914655252 1.77E-16 6.05E-16

COL4A2-AS1 0.140709301 0.553167068 1.974997574 1.78E-16 6.10E-16

AL033384.2 0.172763993 0.514625301 1.574719823 1.79E-16 6.13E-16

TRAV8-4 0.152096451 0.656695569 2.110238227 1.80E-16 6.16E-16

AC007216.3 0.062909036 0.293995291 2.22445388 1.83E-16 6.26E-16

TIMD4 1.461899504 4.944896481 1.75809618 1.85E-16 6.31E-16

AL022322.2 0.005208356 0.029827026 2.517720225 1.85E-16 6.31E-16

AC107959.3 0.290349772 0.978538707 1.752837017 1.88E-16 6.43E-16

TRAV8-3 0.223816011 0.979909809 2.130335726 1.91E-16 6.51E-16

ST6GALNAC4P1 0.168335711 0.505284119 1.585753577 1.91E-16 6.52E-16

AC073636.1 0.079442661 0.280968039 1.822420181 1.92E-16 6.57E-16

ALOX12B 0.090868373 0.354813187 1.965209486 1.93E-16 6.58E-16

AC008649.1 0.003235652 0.094502135 4.868218466 1.95E-16 6.66E-16

LINC01474 0.130085024 0.514798811 1.984553843 1.96E-16 6.69E-16

AC009053.1 0.29931351 1.177778028 1.976338361 1.97E-16 6.72E-16

AL021707.8 0.398045527 1.502743392 1.916593322 1.98E-16 6.75E-16

IL26 0.007047254 0.044584936 2.661423149 1.99E-16 6.79E-16

AC015813.1 0.572767729 1.860718595 1.699837771 2.02E-16 6.88E-16

AC024592.2 0.4734371 0.109491846 -2.112349331 2.02E-16 6.89E-16

AC007728.3 0.014404428 0.108800188 2.917096761 2.03E-16 6.91E-16

AL357500.2 0.577709978 2.089434476 1.8546952 2.04E-16 6.95E-16

B3GNT4 0.467202585 1.644622184 1.815636032 2.06E-16 7.00E-16

AC087276.2 0.195804961 0.833166315 2.089187193 2.06E-16 7.00E-16

AC019080.1 5.948519454 0.532585864 -3.481444594 2.09E-16 7.12E-16

LINC00845 0.576643845 0.06956692 -3.05120719 2.13E-16 7.24E-16

AL353804.2 0.036084652 0.296991746 3.04096559 2.15E-16 7.31E-16

CARD11-AS1 0.00388704 0.078608661 4.337944575 2.18E-16 7.40E-16

PABPC1L 0.912772401 4.341193613 2.249764691 2.18E-16 7.42E-16

IL21R-AS1 0.009237262 0.049855589 2.432218102 2.31E-16 7.85E-16

LINC02657 0.079285061 0.308751319 1.961324344 2.32E-16 7.89E-16

AC103858.1 0.03452358 0.188175942 2.446428204 2.32E-16 7.89E-16

AC090907.2 0.048422237 0.16084224 1.731904685 2.33E-16 7.91E-16

CERS3-AS1 0.046829572 0.261087029 2.479039023 2.38E-16 8.07E-16

AC004263.1 0.03976167 0.266426599 2.744287858 2.39E-16 8.10E-16

AC108749.1 1.098309368 0.105363746 -3.381834034 2.39E-16 8.11E-16

HSPA8P15 0.078928468 0.29046177 1.879730643 2.41E-16 8.17E-16

ITM2BP1 0.035137487 0.255483193 2.862145483 2.41E-16 8.19E-16

AC103810.3 0.566058615 2.473615909 2.127598146 2.42E-16 8.20E-16

RN7SKP296 0.019227778 0.174186835 3.179371648 2.46E-16 8.35E-16

CEACAM3 0.026589309 0.092717151 1.801989921 2.52E-16 8.54E-16

ANKRD36C 0.100477279 0.357829696 1.832403812 2.54E-16 8.62E-16

AL008582.1 0.135247671 0.577301808 2.093721998 2.57E-16 8.69E-16

AL096701.3 0.087898713 0.305176833 1.795731505 2.57E-16 8.71E-16

RPL21P13 0.027472491 0.326355104 3.570382976 2.58E-16 8.72E-16

RAET1E 0.890929207 0.255746995 -1.800593513 2.61E-16 8.81E-16

AC022784.1 0.253861588 0.844935468 1.734799047 2.61E-16 8.81E-16

AC103739.2 0.049491817 0.18634405 1.912706837 2.61E-16 8.84E-16

BHLHA15 0.221811852 1.214245792 2.452652126 2.64E-16 8.91E-16

AC020978.3 0.139303573 0.588476248 2.078751925 2.66E-16 8.98E-16

TRAV36DV7 0.061838876 0.289964705 2.22929131 2.69E-16 9.09E-16

AC009226.1 0.042834798 0.12345161 1.527090447 2.69E-16 9.10E-16

SNX29P1 0.019572544 0.094673669 2.274131951 2.79E-16 9.41E-16

AC012368.1 0.659458854 1.997190781 1.5986176 2.81E-16 9.49E-16

AC105235.1 0.027810552 0.185326648 2.736366035 2.82E-16 9.51E-16

AC245884.3 0.576801096 2.221839945 1.945609082 2.85E-16 9.60E-16

HAVCR1 6.007565563 27.91057405 2.215959404 2.90E-16 9.77E-16

GPR35 0.293800333 1.599365655 2.444591878 2.90E-16 9.77E-16

APOOP1 0.006107487 0.147851721 4.597428386 2.91E-16 9.83E-16

ASMT 0.014891983 0.063562388 2.093637436 2.93E-16 9.87E-16

TMEM92 0.430332932 2.821656251 2.713017087 2.97E-16 1.00E-15

RNU1-67P 0.046360858 0.885557967 4.255607573 3.00E-16 1.01E-15

DIAPH2-AS1 0.016023942 0.105336356 2.716702482 3.01E-16 1.01E-15

AC008781.2 0.107344848 0.414423765 1.948853791 3.07E-16 1.03E-15

AC034243.1 1.247529015 0.437180451 -1.512772574 3.07E-16 1.03E-15

AC022558.3 0.118206197 0.383756613 1.698885936 3.16E-16 1.06E-15

LINC01664 0.013363964 0.055939466 2.065518498 3.17E-16 1.07E-15

PTPRVP 0.012656549 0.067875203 2.423000549 3.18E-16 1.07E-15

PNPLA7 0.564264138 1.717209593 1.605623572 3.18E-16 1.07E-15

RPL37P6 0.39315398 1.227342303 1.642371303 3.21E-16 1.08E-15

TRBV23-1 0.018425888 0.311711993 4.080407585 3.26E-16 1.10E-15

AC005006.1 0.066921196 0.373100215 2.479028058 3.27E-16 1.10E-15

SMIM9 0.005073795 0.055602996 3.454025379 3.27E-16 1.10E-15

MIR7111 0.133812167 0.815199782 2.606944369 3.38E-16 1.13E-15

AC079907.1 0.148504083 0.461424425 1.635591781 3.38E-16 1.14E-15

TRAV24 0.068604758 0.681325397 3.311963443 3.45E-16 1.16E-15

SLX1A-SULT1A3 0.003107762 0.017709093 2.51054254 3.46E-16 1.16E-15

CR559946.2 0.029140515 0.181276599 2.637094434 3.47E-16 1.16E-15

AC012157.2 0.030718889 0.156169496 2.345914728 3.58E-16 1.20E-15

MCCD1P1 0.03412002 0.210459923 2.624855138 3.62E-16 1.21E-15

CALML3 3.394824496 0.073922323 -5.521183095 3.63E-16 1.22E-15

AL356752.1 0.014838017 0.168970669 3.5094026 3.66E-16 1.23E-15

AD000671.2 0.012627414 0.045623 1.853202085 3.67E-16 1.23E-15

EGFLAM 0.925767089 2.621092148 1.501446894 3.73E-16 1.25E-15

SNORD123 0.150153979 1.047172675 2.801984744 3.74E-16 1.25E-15

AC120498.9 0.013123121 0.063476326 2.27410776 3.74E-16 1.25E-15

DCLRE1CP1 0.013550927 0.132754796 3.292300569 3.74E-16 1.25E-15

HAL 0.041285662 0.203634116 2.302266539 3.75E-16 1.26E-15

AC083841.1 0.457585922 0.096964292 -2.238517209 3.82E-16 1.28E-15

KLRC3 0.010073941 0.051366509 2.350199869 3.89E-16 1.30E-15

AC092941.2 0.023328803 0.003355178 -2.797651051 3.91E-16 1.31E-15

NFASC 5.810665203 1.877161087 -1.630150873 3.93E-16 1.31E-15

AL606807.1 0.020175771 0.090497878 2.165260142 3.97E-16 1.33E-15

AC006566.1 0.174338961 0.510535798 1.550117109 3.98E-16 1.33E-15

SCARA5 0.903456468 0.242767964 -1.895877037 3.98E-16 1.33E-15

TBC1D3B 0.007193465 0.03589618 2.319071531 4.00E-16 1.34E-15

RN7SL364P 0.057771776 0.525579516 3.18547231 4.03E-16 1.34E-15

AL022323.2 0.002499327 0.03420028 3.774396619 4.03E-16 1.35E-15

AC005220.1 0.107242481 0.027007217 -1.989459603 4.07E-16 1.36E-15

ERICH6B 0.030938902 0.104977091 1.762580612 4.08E-16 1.36E-15

AL391416.1 0.037002338 0.11417486 1.625556701 4.15E-16 1.39E-15

AF127936.1 0.023081282 0.093103674 2.012114761 4.23E-16 1.41E-15

LIMS1-AS1 0.113409345 0.549407112 2.276336056 4.25E-16 1.42E-15

ZFHX2-AS1 0.067244723 0.255484777 1.925744366 4.35E-16 1.45E-15

AC073655.1 0.032821129 0.163186112 2.313821515 4.37E-16 1.45E-15

AC004854.1 0.025692648 0.214872023 3.064050153 4.42E-16 1.47E-15

AL359314.1 0.016203699 0.339825844 4.390400501 4.42E-16 1.47E-15

AC034199.1 0.009654693 0.039766437 2.042248938 4.45E-16 1.48E-15

AGAP11 0.039721464 0.187049463 2.235429119 4.46E-16 1.48E-15

AP001160.1 0.194224708 0.655605872 1.755102036 4.58E-16 1.52E-15

LINC01389 0.156305425 0.522615172 1.741381159 4.61E-16 1.53E-15

AC020978.7 0.055780963 0.163940545 1.555327959 4.66E-16 1.55E-15

COL1A2 25.30513908 83.16353519 1.716520683 4.66E-16 1.55E-15

MICG 0.008858072 0.216341165 4.610171631 4.68E-16 1.56E-15

RN7SKP173 0.052262833 0.307925944 2.55872618 4.76E-16 1.58E-15

AC018695.4 0.120264572 0.430601555 1.840141825 4.80E-16 1.59E-15

AC087286.4 0.018593857 0.180103071 3.275924845 5.02E-16 1.67E-15

POU5F2 0.003976049 0.082640695 4.377445118 5.05E-16 1.67E-15

AC007496.3 0.008162589 0.137496118 4.074220181 5.10E-16 1.69E-15

AP000997.4 0.004026812 0.046522868 3.530230007 5.12E-16 1.70E-15

AC016383.1 0.024626195 0.005163553 -2.25375763 5.26E-16 1.74E-15

MIR647 1.437939718 6.738405274 2.228404005 5.30E-16 1.76E-15

AC090607.2 0.271769295 0.834130505 1.617890655 5.30E-16 1.76E-15

UBOX5-AS1 0.050898551 0.183001928 1.846162346 5.40E-16 1.79E-15

GOLGA5P1 0.001930854 0.05037243 4.705323541 5.47E-16 1.81E-15

MTND5P28 0.012447597 0.055497104 2.156545254 5.63E-16 1.86E-15

AC009022.1 0.252384983 0.796080532 1.657288313 5.66E-16 1.87E-15

AL451069.2 0.152063032 0.03929819 -1.952134699 5.91E-16 1.95E-15

AC104564.5 0.04828209 0.214476504 2.151259585 5.96E-16 1.96E-15

AL157871.2 0.09581481 0.533825767 2.478048377 5.99E-16 1.98E-15

RPL23AP3 0.046540148 0.169472583 1.864504177 6.04E-16 1.99E-15

AL022334.1 0.002573987 0.029623766 3.524678589 6.08E-16 2.00E-15

LGSN 0.39184147 0.100517586 -1.962822153 6.11E-16 2.01E-15

AC005912.2 0.008788907 0.080809088 3.200761881 6.11E-16 2.01E-15

CCER2 0.072077471 0.453540799 2.653612032 6.15E-16 2.03E-15

AC244197.3 0.107981339 0.42859881 1.988845829 6.17E-16 2.03E-15

SNORD46 0.221810124 1.18370783 2.415915911 6.21E-16 2.05E-15

ERICH4 5.257066422 0.457508877 -3.522386322 6.41E-16 2.11E-15

AC244035.3 0.137401314 2.377059863 4.112710531 6.44E-16 2.12E-15

AC011939.1 0.027203366 0.159322446 2.550092468 6.44E-16 2.12E-15

AC116003.3 0.048784186 0.014354561 -1.764904348 6.54E-16 2.15E-15

CNN2P3 0.067165084 0.011833237 -2.504866632 6.54E-16 2.15E-15

HOXC12 0.012537769 0.066281061 2.402316045 6.55E-16 2.15E-15

TRGV5P 0.013232089 0.102441927 2.952693574 6.57E-16 2.16E-15

LGALS9B 0.00544303 0.022440798 2.043642058 6.58E-16 2.16E-15

AC008737.1 0.105066975 0.532859648 2.342446315 6.62E-16 2.18E-15

AC015660.2 0.303284273 1.177231154 1.956655033 6.67E-16 2.19E-15

AC012307.1 0.163240197 0.034989944 -2.221984082 6.69E-16 2.20E-15

RPL23AP64 0.478601727 1.823033579 1.929443628 6.70E-16 2.20E-15

AC133065.2 0.300273807 0.0921073 -1.704891226 6.80E-16 2.23E-15

TMEM179 0.080882284 0.877063473 3.438785605 6.81E-16 2.24E-15

SLC47A1P1 0.083782365 0.379165453 2.178109016 6.91E-16 2.27E-15

AL596087.2 0.005598164 0.15428045 4.784457581 6.91E-16 2.27E-15

RNU1-14P 2.082746217 11.01866014 2.403389841 6.93E-16 2.27E-15

LINC02515 0.008102108 0.170613961 4.396294522 6.98E-16 2.29E-15

AC138649.4 0.079433599 0.024182415 -1.715791042 7.00E-16 2.30E-15

RPL38P5 0.205348121 0.892044764 2.119044365 7.02E-16 2.30E-15

AC109460.2 0.216084534 0.790538593 1.871240079 7.14E-16 2.34E-15

KIF18BP1 0.66172161 0.0253517 -4.706070024 7.15E-16 2.34E-15

AC027243.1 0.106094994 0.480964159 2.180572809 7.22E-16 2.37E-15

LINC02384 0.864156558 4.964735538 2.522352258 7.27E-16 2.38E-15

AC019294.2 0.01657527 0.050874026 1.617896909 7.29E-16 2.39E-15

AL022316.1 0.202243716 0.585042507 1.532446573 7.35E-16 2.41E-15

AC098614.2 0.604291846 2.253037619 1.898554023 7.35E-16 2.41E-15

AC000036.1 0.006511802 0.042452354 2.704715853 7.43E-16 2.43E-15

AC026412.2 0.047412244 0.192628968 2.022493097 7.63E-16 2.50E-15

FLT1P1 0.007454305 0.039353578 2.400349057 7.66E-16 2.51E-15

AC011933.2 0.002893742 0.073967796 4.675889321 7.72E-16 2.52E-15

ATP11A-AS1 0.003430414 0.108343025 4.981079607 7.75E-16 2.53E-15

AC079907.2 0.006682635 0.065579682 3.294759832 7.76E-16 2.54E-15

AC100803.4 0.062379176 0.517254557 3.051738052 7.77E-16 2.54E-15

AC113208.4 0.023286458 0.073481267 1.657885308 7.85E-16 2.57E-15

AC126365.1 0.001654191 0.016340451 3.304249855 7.97E-16 2.60E-15

ELOVL2-AS1 0.002208066 0.032348845 3.872858982 8.14E-16 2.66E-15

MIR378B 0.013860365 0.435407652 4.97332958 8.16E-16 2.66E-15

MTDHP1 0.030056957 0.114781159 1.933115003 8.20E-16 2.68E-15

AC103691.1 0.388933929 1.332946557 1.777021939 8.26E-16 2.69E-15

GLIS3-AS1 0.33275128 0.10566147 -1.654994824 8.35E-16 2.72E-15

CLIC3 0.534747398 1.644326152 1.620567022 8.36E-16 2.72E-15

AL353795.3 0.010121685 0.072163372 2.833817251 8.48E-16 2.77E-15

TRBV5-5 0.044206428 0.312789636 2.822864647 8.54E-16 2.78E-15

SNORA31 0.548961778 2.763544343 2.331742154 8.60E-16 2.80E-15

CHI3L2 0.442306416 2.096467856 2.244842634 8.60E-16 2.80E-15

MGAM2 0.043343866 0.201556645 2.217285618 8.65E-16 2.82E-15

APOC2 0.088198234 0.442778635 2.327763932 8.96E-16 2.91E-15

GTSF1L 0.008801439 0.124904764 3.826945276 9.01E-16 2.93E-15

CDC42P4 0.013945773 0.085554852 2.617021797 9.02E-16 2.93E-15

RERG-IT1 0.231745942 1.159428306 2.322797634 9.08E-16 2.95E-15

TRBV30 0.110564428 0.62400682 2.496674495 9.14E-16 2.97E-15

NPHS1 13.98247244 0.237648489 -5.878646447 9.17E-16 2.98E-15

CYP4F60P 0.073044896 0.728426155 3.317927346 9.19E-16 2.99E-15

AC135983.4 0.037265432 0.142180287 1.931811579 9.26E-16 3.01E-15

TRAV6 0.068594009 0.376544184 2.456664669 9.46E-16 3.07E-15

GPR22 0.203040608 0.063734372 -1.671624769 9.50E-16 3.09E-15

AC015909.1 0.009029503 0.058533388 2.696541371 9.61E-16 3.12E-15

AC010267.1 0.002054986 0.147716567 6.167559087 9.77E-16 3.17E-15

AC104758.1 0.103819454 0.330996036 1.672737134 9.82E-16 3.19E-15

RPSAP20 0.015224858 0.075190248 2.304116749 9.87E-16 3.20E-15

AC025434.1 0.005495189 0.12370722 4.492616895 9.88E-16 3.21E-15

AL121772.1 0.038744181 0.16557326 2.095418148 1.01E-15 3.28E-15

AC019206.1 0.030126716 0.224953746 2.900513069 1.02E-15 3.30E-15

AC007496.2 0.014476965 0.19036635 3.716947414 1.02E-15 3.32E-15

MIR570 2.855735589 10.53021831 1.882601034 1.02E-15 3.32E-15

AC111186.1 0.294090657 0.083288785 -1.820066801 1.03E-15 3.34E-15

TRBV10-3 0.188714398 0.79042779 2.066429173 1.05E-15 3.42E-15

IL2 0.010778047 0.061840571 2.52045787 1.10E-15 3.56E-15

PKD1L2 0.016491436 0.174416682 3.402749134 1.10E-15 3.57E-15

AC015849.1 0.021489523 0.126740727 2.560174831 1.13E-15 3.67E-15

LINC00111 0.082573595 0.025875367 -1.674101209 1.14E-15 3.67E-15

AC019117.3 1.232244084 23.47148678 4.251549272 1.15E-15 3.73E-15

AC008870.4 0.03880417 0.166308088 2.099574738 1.16E-15 3.74E-15

KRT40 0.152034346 0.016667326 -3.189302677 1.17E-15 3.77E-15

FIRRE 0.035576001 0.172943355 2.281323314 1.17E-15 3.78E-15

AC234782.3 0.01537211 0.124542476 3.018250769 1.17E-15 3.79E-15

FOXS1 1.948901399 5.715997555 1.552344198 1.21E-15 3.91E-15

H3P25 0.047632456 0.414771367 3.122299462 1.21E-15 3.91E-15

BNIP3P19 0.005808539 0.093903624 4.014933526 1.23E-15 3.97E-15

FABP5P7 0.053096545 0.195739834 1.882247502 1.23E-15 3.97E-15

SNORD12B 0.260109582 1.217535684 2.226772605 1.24E-15 3.99E-15

BEST4 0.215099418 1.350160675 2.650055576 1.25E-15 4.04E-15

SLC22A7 11.08029853 1.266998231 -3.128510336 1.26E-15 4.06E-15

AC092542.1 0.172227987 0.657488916 1.932646974 1.28E-15 4.12E-15

AC132938.6 0.093328151 0.379528953 2.023825734 1.30E-15 4.20E-15

AC027601.3 0.223624711 0.666671988 1.575897493 1.32E-15 4.25E-15

LINC00632 0.012957762 0.081767923 2.657718383 1.35E-15 4.36E-15

AC099684.2 1.919925497 0.485309291 -1.984073941 1.38E-15 4.43E-15

CYTL1 0.620975596 1.974365518 1.668780626 1.38E-15 4.44E-15

SLC22A16 0.018373599 0.091639619 2.318337184 1.38E-15 4.45E-15

AL158071.4 0.089309709 0.305014679 1.771989745 1.43E-15 4.59E-15

CCL28 2.527795814 7.263620979 1.522808991 1.43E-15 4.59E-15

LINC02803 0.058947871 0.24972618 2.082835473 1.45E-15 4.64E-15

AC012181.2 0.51326245 1.485844042 1.533514072 1.45E-15 4.65E-15

AL135844.1 0.310782456 1.484519437 2.256019016 1.47E-15 4.72E-15

PFN3 0.166305973 0.035731614 -2.218566992 1.49E-15 4.78E-15

RNU6-850P 0.522957382 2.393153533 2.194147673 1.51E-15 4.84E-15

PRELID2P1 0.009045189 0.1325491 3.873232394 1.53E-15 4.90E-15

AC104964.1 0.137810995 0.504949755 1.873448843 1.54E-15 4.94E-15

CNPY1 0.001598436 0.03911181 4.612871778 1.59E-15 5.07E-15

AC005154.2 0.228774068 0.724188804 1.662442346 1.59E-15 5.08E-15

AL136528.1 0.040002573 0.366775542 3.196732726 1.62E-15 5.18E-15

AL157392.4 0.134044345 0.56613771 2.078442663 1.63E-15 5.20E-15

AF067845.1 0.060378785 0.011909536 -2.341924546 1.65E-15 5.26E-15

C15orf48 1.413460164 4.141319402 1.550859252 1.65E-15 5.27E-15

HMHB1 0.027173724 0.196337655 2.853052678 1.67E-15 5.34E-15

RN7SL19P 0.025150109 0.17333284 2.784908451 1.68E-15 5.35E-15

Z99916.1 0.043563042 0.191263806 2.1343873 1.68E-15 5.35E-15

SERPINE1 26.71965024 121.9353135 2.190142975 1.69E-15 5.39E-15

SH3GL1P2 0.045409892 0.173368643 1.932764473 1.71E-15 5.46E-15

AL390729.1 0.012988309 0.212610207 4.032925373 1.71E-15 5.46E-15

AC090164.3 0.016996844 0.051675788 1.6042216 1.72E-15 5.48E-15

BCL11A 0.160436965 0.475625296 1.567818863 1.73E-15 5.52E-15

AC010618.2 0.09451756 0.433380676 2.196980538 1.73E-15 5.53E-15

AC007272.2 0.030998435 0.246937252 2.993877211 1.75E-15 5.59E-15

ENPP7P4 0.054145798 0.221583111 2.032926627 1.78E-15 5.67E-15

AP003392.2 0.848771507 3.005051687 1.823941675 1.78E-15 5.67E-15

AC104534.1 0.252252502 0.950528673 1.913861567 1.81E-15 5.77E-15

AC020763.4 0.035518957 0.11621389 1.710121402 1.82E-15 5.80E-15

Z84480.1 0.063204588 0.237496424 1.909804602 1.84E-15 5.85E-15

NTNG2 0.131784685 0.440321129 1.740373358 1.85E-15 5.90E-15

AC099542.1 0.02296663 0.107552312 2.227427453 1.87E-15 5.94E-15

VSX1 0.015353039 0.143341574 3.222860967 1.92E-15 6.09E-15

AC068790.7 0.110991305 0.381369551 1.780742999 1.92E-15 6.10E-15

SETP17 0.035828151 0.110956968 1.630834779 1.93E-15 6.12E-15

CDH9 0.730981499 0.17186065 -2.088595639 1.93E-15 6.13E-15

AC010457.1 0.082443346 0.452768206 2.457297691 1.94E-15 6.17E-15

IL22RA1 0.865452788 2.586410077 1.579424009 1.95E-15 6.20E-15

ZG16 0.03241781 0.011054307 -1.552178004 1.99E-15 6.32E-15

A2MP1 0.037902871 0.121406702 1.67946902 2.00E-15 6.34E-15

LINC00603 0.049585117 0.013315379 -1.896813694 2.02E-15 6.41E-15

AC129926.1 0.078316205 0.00978408 -3.000802777 2.03E-15 6.44E-15

S100A8 3.659235781 10.48543314 1.518772175 2.03E-15 6.45E-15

RNU6-247P 0.449619025 0.067000012 -2.746469814 2.03E-15 6.45E-15

MYOSLID 0.086680165 0.292671303 1.755507491 2.04E-15 6.47E-15

AC007272.1 0.021619173 0.159546172 2.883590739 2.05E-15 6.49E-15

AP003043.1 0.017329147 0.131634666 2.925266909 2.05E-15 6.49E-15

AL031587.1 0.007306227 0.097143978 3.732926071 2.06E-15 6.53E-15

AL138831.3 0.143119701 0.536279925 1.905763974 2.07E-15 6.55E-15

AP005264.5 0.001527775 0.021949947 3.844713398 2.09E-15 6.61E-15

MIR126 0.357106199 2.802709243 2.972397003 2.10E-15 6.66E-15

BLOC1S5-TXNDC5 0.729780425 0.198290467 -1.879847132 2.11E-15 6.70E-15

AL645939.4 0.314076355 0.920294123 1.550979679 2.13E-15 6.75E-15

RPL11P3 0.186554939 0.555282818 1.573622203 2.14E-15 6.79E-15

AL109809.2 0.006353783 0.076375203 3.587416666 2.16E-15 6.84E-15

TRIM73 0.011793412 0.049113925 2.058151017 2.17E-15 6.87E-15

SLC28A2 0.761291573 0.075849001 -3.327247026 2.18E-15 6.88E-15

EMP2P1 0.030365937 0.211530158 2.800337583 2.18E-15 6.90E-15

ASMTL-AS1 0.500563539 3.275899338 2.710265911 2.21E-15 7.00E-15

AC134669.1 0.02379892 0.079905288 1.74739487 2.25E-15 7.10E-15

CPNE6 1.886353055 0.022183501 -6.409968829 2.26E-15 7.13E-15

AC098934.1 1.56642688 0.336224979 -2.219978613 2.29E-15 7.24E-15

BPI 0.437693637 0.085133916 -2.362115514 2.30E-15 7.28E-15

ASS1 292.4275089 46.38766543 -2.656265887 2.32E-15 7.32E-15

FAM209A 0.023796621 0.096686371 2.022555844 2.38E-15 7.50E-15

RNU6-312P 0.263787836 1.222036765 2.211837746 2.39E-15 7.54E-15

AC006435.2 0.201304351 0.937788223 2.219883808 2.41E-15 7.61E-15

HAO1 0.400816922 0.102745325 -1.963870664 2.43E-15 7.66E-15

ENKUR 0.193718139 0.558118736 1.526613034 2.44E-15 7.69E-15

RN7SL481P 0.11636316 0.494376254 2.086975073 2.44E-15 7.70E-15

REELD1 0.114162678 0.6019447 2.398539873 2.47E-15 7.78E-15

LINC01483 0.005129308 0.022235378 2.116020717 2.49E-15 7.85E-15

PTX4 0.011800043 0.059022582 2.3224749 2.52E-15 7.95E-15

ATP10B 0.146464073 0.033622693 -2.123039618 2.53E-15 7.97E-15

AC048382.2 0.179138951 0.644482009 1.847061019 2.58E-15 8.12E-15

AP002812.3 0.037167593 0.191661375 2.366442463 2.60E-15 8.18E-15

AC114402.1 0.085288147 0.416867369 2.28917128 2.62E-15 8.26E-15

AC010976.1 0.072017193 0.241902787 1.748014117 2.63E-15 8.27E-15

AC005899.7 0.329949503 1.153752238 1.806016296 2.64E-15 8.32E-15

AL138693.1 0.318990567 0.982916411 1.623554972 2.65E-15 8.34E-15

TTC16 0.058585024 0.192953596 1.71965012 2.67E-15 8.41E-15

AC024451.4 0.020810697 0.109915871 2.401002514 2.68E-15 8.44E-15

AC111000.2 2.205802839 9.93726236 2.171544611 2.70E-15 8.49E-15

AL157791.2 0.123332945 0.450409971 1.868680537 2.73E-15 8.58E-15

HNRNPA1P59 0.083392509 0.256022707 1.618282072 2.73E-15 8.59E-15

GAPDHP71 0.035674068 0.11771638 1.722367423 2.75E-15 8.64E-15

Z69666.1 0.033658556 0.182249163 2.436867003 2.77E-15 8.69E-15

AC135279.2 0.053482805 0.232248698 2.118523477 2.77E-15 8.71E-15

AC007953.1 0.425381453 2.477238388 2.54190367 2.78E-15 8.72E-15

ZBTB12BP 0.054784234 0.215272927 1.974334208 2.79E-15 8.77E-15

CATSPERG 0.168024106 0.528706904 1.653799934 2.81E-15 8.83E-15

AL160286.3 0.175950959 0.017758101 -3.308624119 2.82E-15 8.87E-15

NPPA 0.035653505 0.151546055 2.087640463 2.83E-15 8.87E-15

AC010261.2 0.041846596 0.226296068 2.435029355 2.85E-15 8.95E-15

LINC00894 0.117141069 0.545007525 2.218029179 2.86E-15 8.98E-15

LINC01255 0.023751126 0.004977231 -2.254580646 2.86E-15 8.98E-15

AC012485.1 0.011066125 0.051551717 2.219870415 2.92E-15 9.17E-15

MSS51 0.217666512 0.622219774 1.515304779 2.93E-15 9.18E-15

TRAV35 0.067022355 0.318884614 2.250320207 3.01E-15 9.43E-15

AC136489.1 0.01510228 0.067188672 2.15345168 3.01E-15 9.44E-15

LINC02367 0.018618498 0.071598241 1.943187489 3.06E-15 9.57E-15

C3orf67-AS1 0.000924042 0.026299606 4.830939335 3.08E-15 9.65E-15

AL136452.1 0.003861481 0.067772619 4.133476503 3.13E-15 9.80E-15

AL589743.5 0.00666615 0.103980821 3.963319853 3.15E-15 9.86E-15

AC010642.1 0.012647915 0.061116432 2.272660735 3.20E-15 9.99E-15

AL450384.1 0.081069558 0.349933559 2.109848855 3.21E-15 1.00E-14

RNU6-1011P 0.126873626 0.511155543 2.010370162 3.24E-15 1.01E-14

AL353681.1 0.014457483 0.090470683 2.645633903 3.30E-15 1.03E-14

AC017048.3 0.341540392 1.200181064 1.813123959 3.32E-15 1.04E-14

FOXH1 0.014238421 0.056038465 1.976628311 3.32E-15 1.04E-14

AP4B1-AS1 0.070945853 0.245309291 1.789811615 3.36E-15 1.05E-14

LINC02244 0.258086935 0.961574116 1.897540949 3.37E-15 1.05E-14

AL139385.1 0.411065233 0.129716393 -1.664006548 3.38E-15 1.05E-14

RAET1L 0.230924435 0.049748724 -2.214689419 3.38E-15 1.05E-14

GAPDHP68 0.019613244 0.074939725 1.933902522 3.40E-15 1.06E-14

RPL9P2 0.045856471 0.189878344 2.049878128 3.41E-15 1.06E-14

AC018644.1 0.064440033 0.281097144 2.125039654 3.43E-15 1.07E-14

AC117500.2 0.03526777 0.221652003 2.65187415 3.44E-15 1.07E-14

KCNK17 0.065368599 0.594725957 3.185555357 3.56E-15 1.11E-14

AC013452.3 0.014274932 0.081340798 2.510495271 3.58E-15 1.11E-14

BMP3 0.353946778 0.084489759 -2.06668405 3.59E-15 1.12E-14

AC010894.3 0.013239253 0.064342423 2.280948503 3.59E-15 1.12E-14

AC087289.1 0.039580718 0.149026292 1.912697189 3.68E-15 1.15E-14

TRAV26-2 0.057277341 0.388472446 2.761775838 3.69E-15 1.15E-14

AC092301.1 0.06719418 0.241983787 1.848502211 3.75E-15 1.17E-14

GAPDHP37 0.004203862 0.07368311 4.131546753 3.78E-15 1.17E-14

AP005131.1 0.014693054 0.128013672 3.123091704 3.82E-15 1.19E-14

A3GALT2 0.021433803 0.109776208 2.356605664 3.91E-15 1.21E-14

MRLN 0.631245909 0.0324992 -4.279726004 3.91E-15 1.21E-14

AL133352.1 0.018037572 0.06576487 1.86631195 3.92E-15 1.22E-14

AL356273.4 0.056525316 0.165160078 1.54689596 3.93E-15 1.22E-14

TRAV23DV6 0.112269687 0.428993963 1.933988895 3.94E-15 1.22E-14

AL358115.1 0.038182069 0.140490531 1.879505715 3.95E-15 1.22E-14

AC117503.4 0.127779634 0.402620444 1.655762513 3.97E-15 1.23E-14

ZAN 0.003512768 0.047161977 3.746943882 4.02E-15 1.25E-14

RPL10P6 3.010269059 10.44209538 1.794446894 4.05E-15 1.26E-14

LINC01529 0.012129416 0.109539807 3.174873261 4.07E-15 1.26E-14

AC004461.3 0.057300405 0.202983877 1.824747909 4.14E-15 1.28E-14

LINC01891 0.005773617 0.069119786 3.581551448 4.14E-15 1.28E-14

U73169.1 0.058882449 0.285203073 2.276079934 4.15E-15 1.28E-14

LINC01789 0.237053931 2.248996411 3.245994138 4.17E-15 1.29E-14

AC005899.8 0.089888873 0.373835774 2.056190189 4.24E-15 1.31E-14

AL049714.1 0.041768133 0.202621991 2.278316202 4.31E-15 1.33E-14

AC103591.3 0.275541812 0.999781431 1.859341475 4.34E-15 1.34E-14

AC245052.4 0.090430671 0.261874188 1.533989788 4.43E-15 1.37E-14

AC020917.1 0.028515752 0.093273766 1.709712292 4.44E-15 1.37E-14

AL513218.1 0.142617511 0.595938404 2.063012096 4.51E-15 1.39E-14

TRGV5 0.128248552 0.435064862 1.762287971 4.56E-15 1.41E-14

SPATA21 0.005337945 0.044315312 3.053448833 4.56E-15 1.41E-14

AP001341.1 0.001870058 0.081068376 5.437984598 4.57E-15 1.41E-14

AC087752.2 0.043375357 0.247698112 2.513635335 4.62E-15 1.43E-14

NSUN5P1 0.627663686 2.109712935 1.748983059 4.72E-15 1.46E-14

AC096669.1 0.008181739 0.085176453 3.379975291 4.79E-15 1.48E-14

AL080317.1 0.18743386 0.589897072 1.65408165 4.83E-15 1.49E-14

AL157791.1 0.031944475 0.123558795 1.951559395 4.93E-15 1.52E-14

AC008050.1 0.076283648 0.30441559 1.996596508 4.99E-15 1.54E-14

AL031709.1 0.140392904 0.540365565 1.944465719 5.01E-15 1.54E-14

EBF3 0.35716588 1.150825616 1.688003066 5.02E-15 1.55E-14

IMPDH1P5 0.039731059 0.180377448 2.182679808 5.06E-15 1.56E-14

AL731733.1 0.03407874 0.108556864 1.671507056 5.06E-15 1.56E-14

OR9N1P 0.003254017 0.097479068 4.904798914 5.10E-15 1.57E-14

AC091959.2 0.022174546 0.284801162 3.682978552 5.14E-15 1.58E-14

AC112722.1 0.090853519 0.328100664 1.852524204 5.18E-15 1.59E-14

MIR6753 0.047639953 0.394986168 3.051558239 5.20E-15 1.60E-14

LINC02273 0.08570194 0.279799367 1.70699293 5.22E-15 1.61E-14

PGBD5 0.704718219 2.431164767 1.786529254 5.22E-15 1.61E-14

AL356750.1 0.022464623 0.085110385 1.921680298 5.24E-15 1.61E-14

M1AP 0.922131493 0.271860063 -1.762108257 5.25E-15 1.62E-14

AC093010.1 0.003620557 0.019152381 2.403239999 5.29E-15 1.63E-14

ATP5MFP4 0.045101258 0.321704044 2.834494488 5.30E-15 1.63E-14

AP001180.4 0.002771323 0.047724017 4.106068615 5.31E-15 1.63E-14

AC087257.2 0.045238422 0.230682613 2.35028876 5.37E-15 1.65E-14

AC127024.8 0.022962914 0.156637169 2.770048983 5.37E-15 1.65E-14

AC105339.2 0.038800143 0.144082515 1.892761401 5.46E-15 1.68E-14

TNNT1 0.231251107 1.199889323 2.375369161 5.48E-15 1.68E-14

ACBD3-AS1 0.139406209 0.420900858 1.594185632 5.49E-15 1.68E-14

RPL21P136 0.027152176 0.258807825 3.252741518 5.50E-15 1.69E-14

AP001180.1 0.003520794 0.025540248 2.858799795 5.56E-15 1.71E-14

AL121782.1 0.072199328 0.25322588 1.810367554 5.62E-15 1.72E-14

RN7SKP150 0.130307209 0.532791252 2.031653492 5.65E-15 1.73E-14

DSCR9 0.027287923 0.081018167 1.569982838 5.68E-15 1.74E-14

AP001107.6 0.017164557 0.109399804 2.672105665 5.69E-15 1.75E-14

AC068051.1 0.054396318 0.348146862 2.67811512 5.73E-15 1.76E-14

TBC1D26 0.001035676 0.010919614 3.398276706 5.75E-15 1.76E-14

AL160287.1 0.007797066 0.068092635 3.12649554 5.80E-15 1.78E-14

LINC01611 0.004500129 0.12442183 4.78912959 5.82E-15 1.78E-14

AC133644.1 0.168096263 1.568148095 3.221702259 5.92E-15 1.81E-14

LRP1-AS 0.011091308 0.062849094 2.502462375 5.92E-15 1.81E-14

Z98885.3 0.039882354 0.155290183 1.961144166 6.00E-15 1.84E-14

TRBV13 0.06753949 0.642841843 3.250660639 6.13E-15 1.87E-14

VSIG8 1.052724628 0.156703924 -2.748014891 6.15E-15 1.88E-14

RPS2P24 0.005556722 0.045892746 3.045960059 6.16E-15 1.88E-14

AC007878.1 0.124091059 0.587720868 2.243731957 6.22E-15 1.90E-14

AC010327.3 0.021630369 0.180655865 3.062113901 6.25E-15 1.91E-14

AC012651.1 0.152596748 0.542478263 1.829841114 6.26E-15 1.91E-14

FAM172BP 0.015432348 0.061539103 1.995545848 6.31E-15 1.93E-14

AL121594.1 0.012149506 0.049514842 2.026963403 6.34E-15 1.93E-14

NPM1P46 0.044273026 0.137372115 1.633589291 6.36E-15 1.94E-14

GPR25 0.017796742 0.079476503 2.158915245 6.47E-15 1.97E-14

AL161773.1 0.042797663 0.209367261 2.290431929 6.66E-15 2.03E-14

RPS17P2 0.05006045 0.176400455 1.817111123 6.70E-15 2.04E-14

AC010327.5 0.027839093 0.101039765 1.85973907 6.73E-15 2.05E-14

MTND4P26 0.010379091 0.048016331 2.209845081 6.78E-15 2.07E-14

TMLHE-AS1 0.018296734 0.056654692 1.630609266 6.79E-15 2.07E-14

AC010907.1 0.017098118 0.005103292 -1.744337399 6.80E-15 2.07E-14

AP002907.1 0.159508453 0.54597421 1.775199927 6.88E-15 2.10E-14

AC008700.1 0.005684533 0.093920801 4.046330849 6.89E-15 2.10E-14

RPL5P11 0.017791142 0.112803921 2.664586236 7.08E-15 2.16E-14

AC023301.1 0.023596795 0.159648552 2.758236639 7.29E-15 2.22E-14

HMGB2P1 0.037993522 0.189643294 2.319463004 7.31E-15 2.22E-14

AC008663.3 0.030584735 0.197723939 2.692603889 7.42E-15 2.25E-14

FBXL16 4.41547229 13.12623379 1.571813369 7.45E-15 2.26E-14

AC026333.4 0.077933936 0.257530006 1.724416956 7.47E-15 2.27E-14

SULT1A3 0.005643854 0.030740021 2.445365603 7.53E-15 2.29E-14

CREG2 0.122122549 0.482505367 1.982215384 7.57E-15 2.30E-14

AC005342.2 0.017221678 0.11860115 2.783820342 7.60E-15 2.31E-14

LINC02677 0.230216358 0.677928585 1.558142954 7.62E-15 2.31E-14

AC022784.5 0.237053066 0.78364534 1.72499082 7.66E-15 2.33E-14

LINC00589 0.004049243 0.027580169 2.767907173 7.68E-15 2.33E-14

TRIML2 0.008952889 0.059695433 2.737195429 7.88E-15 2.39E-14

AC092611.1 0.03568021 0.105781009 1.567884637 7.96E-15 2.41E-14

AL133330.1 0.043470985 0.241123775 2.471649203 8.02E-15 2.43E-14

SSXP10 0.008406803 0.20444881 4.604038508 8.05E-15 2.44E-14

PLXNB3 0.12341833 0.500386446 2.019486028 8.29E-15 2.51E-14

INHBA-AS1 0.031580925 0.246114993 2.962207218 8.31E-15 2.52E-14

AC092119.2 0.153225191 0.659499182 2.10571737 8.33E-15 2.52E-14

TRAV10 0.048122876 0.335127569 2.799915593 8.40E-15 2.54E-14

AC146507.2 0.035940537 0.206470043 2.522248603 8.52E-15 2.58E-14

AC099786.1 0.002351742 0.208959184 6.47334785 8.53E-15 2.58E-14

AL354751.1 0.010728969 0.133159982 3.633577226 8.64E-15 2.61E-14

AC005324.3 0.002401857 0.014250586 2.568798968 8.77E-15 2.65E-14

AC074366.1 0.00363451 0.058855576 4.017346225 8.77E-15 2.65E-14

AL645924.2 0.044946693 0.289508133 2.687316993 8.87E-15 2.68E-14

HTR1F 0.217808437 0.67250603 1.626487367 8.96E-15 2.71E-14

TPO 0.004699489 0.018535719 1.979732136 9.09E-15 2.74E-14

LINC00705 0.001890445 0.03642279 4.268043557 9.10E-15 2.75E-14

OSM 0.525026197 1.919826635 1.870514724 9.11E-15 2.75E-14

AL034399.2 0 0.12724402 Inf 9.27E-15 2.80E-14

RNU4-62P 0.346408164 2.113860407 2.609335271 9.51E-15 2.87E-14

SYNGR4 0.028194065 0.13262929 2.23393599 9.62E-15 2.90E-14

ISL2 0.010006858 0.064144337 2.680332841 9.62E-15 2.90E-14

THEM7P 0.006744615 0.039208203 2.539347571 9.68E-15 2.92E-14

MUC6 0.618639668 0.101126121 -2.612943654 9.77E-15 2.94E-14

LINC00222 0.01293364 0.047595108 1.87968497 9.82E-15 2.96E-14

AC114760.2 0.0396378 0.24355636 2.619306869 9.84E-15 2.96E-14

MIR4420 0.037886468 0.526033294 3.795399559 9.99E-15 3.01E-14

AC005740.3 0.06267081 0.284944327 2.184814534 1.00E-14 3.02E-14

C18orf15 0.002241139 0.081274313 5.18049526 1.01E-14 3.04E-14

AC114730.3 0.105112435 0.500254576 2.250729112 1.02E-14 3.06E-14

RN7SKP16 0.403985548 1.262591649 1.644012525 1.05E-14 3.17E-14

AC005785.2 0.184874462 0.757598718 2.03488794 1.06E-14 3.19E-14

DHDDS-AS1 0.13790796 0.404823175 1.553586154 1.08E-14 3.24E-14

TLL1 0.834454313 2.536952502 1.604191541 1.12E-14 3.36E-14

AC006441.4 1.586704021 0.082427006 -4.26677214 1.17E-14 3.50E-14

AC012213.2 0.010199527 0.04336584 2.08805686 1.17E-14 3.50E-14

HSPD1P6 0.040245493 0.11653179 1.533824428 1.17E-14 3.51E-14

LINC01311 0.167618835 0.481194399 1.521435576 1.17E-14 3.51E-14

AC004584.1 0.038649855 0.208054316 2.428425298 1.18E-14 3.55E-14

AC087884.1 0.113810315 0.971543567 3.093647372 1.21E-14 3.62E-14

LRRC37A9P 0.004870188 0.020562447 2.077962765 1.22E-14 3.65E-14

OR7E94P 0.052353562 0.157124806 1.58555135 1.22E-14 3.66E-14

RN7SL67P 0.042388856 0.426426292 3.330539458 1.23E-14 3.68E-14

MRTFA-AS1 0.015995722 0.133465395 3.060707758 1.24E-14 3.71E-14

PZP 0.044686897 0.146526019 1.71323311 1.25E-14 3.73E-14

AC024145.1 0.14571822 0.535562285 1.877873091 1.25E-14 3.75E-14

FSCN3 0.01481064 0.04954952 1.742237131 1.26E-14 3.78E-14

RPL10P3 0.341681747 1.018850855 1.576217793 1.27E-14 3.79E-14

MIR499A 0.045923037 0.403001614 3.133495651 1.28E-14 3.82E-14

AC008592.1 0.010721082 0.049321737 2.201773144 1.28E-14 3.83E-14

AL117382.2 0.348501802 0.057386073 -2.602393561 1.29E-14 3.85E-14

XXYLT1-AS1 0.204889618 0.06682127 -1.616467567 1.31E-14 3.91E-14

AL078459.1 0.081824285 0.282804933 1.789206287 1.31E-14 3.91E-14

POLR2CP1 0.008352786 0.059980206 2.844157102 1.33E-14 3.96E-14

AP001029.3 0.036523498 0.190810351 2.385242592 1.33E-14 3.97E-14

SNORC 0.209656909 0.686175812 1.710547899 1.34E-14 4.00E-14

LINC02561 0.039582635 0.1497836 1.919940118 1.34E-14 4.00E-14

LY6E-DT 0.24588886 1.076474862 2.130236351 1.38E-14 4.11E-14

BVES-AS1 0.141736004 0.029941455 -2.242990042 1.42E-14 4.24E-14

AC105910.1 0.009541414 0.271886951 4.832660035 1.42E-14 4.24E-14

TRBV10-2 0.041812886 0.420734824 3.330891707 1.44E-14 4.28E-14

AC010530.1 0.078999766 0.286894834 1.860601703 1.46E-14 4.33E-14

AC133561.1 0.005626685 0.028937897 2.362602969 1.46E-14 4.35E-14

AL139123.1 0.082770263 0.360858653 2.124249398 1.48E-14 4.41E-14

AC012186.2 0.147331077 0.460884319 1.645342912 1.54E-14 4.59E-14

MIR548AA2 0.197358683 1.553989385 2.977084748 1.55E-14 4.62E-14

FGF10-AS1 0.012710203 0.002726543 -2.220842508 1.56E-14 4.64E-14

AC016769.1 0.011416574 0.139770717 3.613860504 1.58E-14 4.69E-14

LINC01886 0.307132188 1.617644497 2.396962965 1.58E-14 4.69E-14

AC016722.2 0.087689023 0.25058467 1.514830002 1.58E-14 4.70E-14

AFM 13.44497647 0.557824857 -4.591111197 1.59E-14 4.73E-14

AL132657.2 0.006387816 0.137314395 4.426016373 1.62E-14 4.80E-14

VN2R9P 0.004189532 0.046988442 3.487444962 1.62E-14 4.80E-14

AL449212.1 0.465918425 1.321462386 1.503986073 1.64E-14 4.85E-14

AC079384.1 0.015391495 0.201908179 3.713494054 1.64E-14 4.86E-14

AC005954.1 0.041962832 0.211144406 2.331046069 1.65E-14 4.90E-14

CEACAM22P 0.008534008 0.057544988 2.753394853 1.68E-14 4.98E-14

TREX2 0.333426857 1.252323939 1.909165572 1.69E-14 5.01E-14

CNTNAP4 0.026410639 0.006688646 -1.981333081 1.72E-14 5.08E-14

AC112255.1 0.002981233 0.017518675 2.554912859 1.74E-14 5.16E-14

AC120498.8 0.135769697 0.047355953 -1.519543813 1.75E-14 5.17E-14

AL118505.1 0.271120793 0.071757862 -1.917726958 1.76E-14 5.20E-14

AC134407.3 0.045093886 0.149360531 1.727795205 1.76E-14 5.20E-14

LRIT2 0.00286906 0.053369229 4.217358075 1.76E-14 5.20E-14

AL049634.2 0.001850433 0.022507872 3.604494788 1.81E-14 5.33E-14

LINC01508 1.247707283 3.608484867 1.532113692 1.81E-14 5.34E-14

AL353662.2 0.056528918 0.198774176 1.814069361 1.81E-14 5.35E-14

RPL31P52 0.073388158 0.3043666 2.052190851 1.82E-14 5.38E-14

AL359852.1 0.022901837 0.083684722 1.869500899 1.85E-14 5.47E-14

GTF3C2-AS1 0.031340005 0.12649022 2.01294851 1.86E-14 5.48E-14

TBC1D27P 0.01223008 0.037575103 1.619343249 1.86E-14 5.49E-14

AC009533.3 0.012850851 0.104072967 3.017659623 1.87E-14 5.51E-14

AL353801.3 0.088063481 0.401032331 2.187102776 1.87E-14 5.51E-14

AP000439.2 0.002875944 0.080879926 4.813674145 1.88E-14 5.56E-14

LILRP1 0.005230333 0.029883496 2.514374313 1.90E-14 5.61E-14

PSMD10P1 0.096606309 0.473264538 2.292457517 1.91E-14 5.62E-14

FSIP2-AS1 0.074798642 0.282715429 1.918266634 1.93E-14 5.68E-14

SELE 3.96314371 1.125186636 -1.81648096 1.93E-14 5.69E-14

AL117350.1 0.023830157 0.103835583 2.123440568 1.98E-14 5.83E-14

SAP25 0.026029942 0.136610009 2.391819187 2.01E-14 5.92E-14

AC026150.3 0.005143164 0.038312999 2.897105955 2.01E-14 5.92E-14

MS4A15 0.002915787 0.032223656 3.466162825 2.01E-14 5.92E-14

CNR1 0.120202125 0.394957521 1.716235091 2.02E-14 5.94E-14

ACY1 8.587370308 1.968624897 -2.12502816 2.08E-14 6.10E-14

AC005324.5 0.044987565 0.184904577 2.039182755 2.09E-14 6.15E-14

AL117328.1 0.143187946 0.422381336 1.560636036 2.10E-14 6.18E-14

DOC2GP 0.068241907 0.271246848 1.990876504 2.11E-14 6.20E-14

AC005776.1 0.035039633 0.333274108 3.249649648 2.13E-14 6.25E-14

AL157912.1 0.003747485 0.06016421 4.00491092 2.17E-14 6.36E-14

AC048383.1 0.000378654 0.057823626 7.254636053 2.18E-14 6.39E-14

GBP6 0.026712693 0.093125278 1.801647421 2.18E-14 6.40E-14

CAMK2N2 0.102393013 0.374546558 1.871027793 2.18E-14 6.40E-14

CCDC168 0.004787509 0.015728753 1.716057313 2.20E-14 6.47E-14

GPR12 0.031455255 0.003007287 -3.386766569 2.22E-14 6.51E-14

AC007342.6 0.041899612 0.145838108 1.799358957 2.23E-14 6.54E-14

AC012368.2 0.029730459 0.118203938 1.991264466 2.24E-14 6.57E-14

AL008723.2 0.046784342 0.287412023 2.619022758 2.29E-14 6.72E-14

AL159169.2 0.141663383 0.4179915 1.561006702 2.31E-14 6.75E-14

AMY2B 0.407151139 1.425070527 1.807396977 2.31E-14 6.75E-14

AC008154.1 0.061800597 0.015702159 -1.976657819 2.32E-14 6.80E-14

AP003392.1 0.517678371 1.759650235 1.765160746 2.38E-14 6.97E-14

AC011611.3 0.03887101 0.221581098 2.511068308 2.43E-14 7.12E-14

ASS1P8 0.025412822 0.005771049 -2.138651058 2.43E-14 7.12E-14

AC011297.1 2.367640214 0.196664734 -3.589639682 2.49E-14 7.28E-14

LINC02809 0.005793526 0.037793911 2.705640189 2.56E-14 7.49E-14

SNHG22 0.226258552 0.790850408 1.805432506 2.57E-14 7.52E-14

AC104365.3 0.015279216 0.12366283 3.016769491 2.59E-14 7.56E-14

NETO1 0.024227083 0.144877896 2.580144867 2.60E-14 7.59E-14

AL137856.1 0.008258402 0.059157839 2.840634884 2.60E-14 7.60E-14

AC068722.2 0.076708431 0.27382581 1.835801388 2.60E-14 7.60E-14

ZDHHC20-IT1 0.097635697 0.286261883 1.551854961 2.66E-14 7.76E-14

AC099518.2 0.034389362 0.099387616 1.531103736 2.66E-14 7.78E-14

AC005324.4 0.000647856 0.008362738 3.690229866 2.68E-14 7.81E-14

BCL2L1-AS1 0.074774261 0.503164811 2.750417376 2.70E-14 7.88E-14

TRBJ2-2 0.152181082 0.851993732 2.485053793 2.71E-14 7.90E-14

HLA-U 0.988192049 3.252757761 1.718800032 2.72E-14 7.93E-14

AL136982.6 0.023478698 0.109410706 2.220329568 2.76E-14 8.04E-14

AL355578.1 0.000775003 0.059477681 6.262003629 2.76E-14 8.04E-14

GK-AS1 0.18814117 0.639210314 1.764475109 2.78E-14 8.10E-14

AL021707.1 0.103823628 0.347080997 1.741137579 2.82E-14 8.22E-14

KRT8P39 0.057729853 0.16442511 1.510041186 2.82E-14 8.23E-14

SLC22A13 4.29512116 0.62710593 -2.775917762 2.87E-14 8.36E-14

H2BC17 0.051967681 0.356193988 2.776976586 2.87E-14 8.36E-14

LINC02863 0.013996643 0.115511022 3.044877789 2.88E-14 8.39E-14

SNORD124 0.107708433 0.583803879 2.438352584 2.88E-14 8.39E-14

TBL1Y 0.746666982 0.077791497 -3.262780557 2.90E-14 8.45E-14

AL049552.1 0.211944165 0.704279811 1.732464478 2.94E-14 8.55E-14

STYK1 0.529848755 0.154276335 -1.78006382 2.94E-14 8.55E-14

AP003721.3 0.159227334 0.028679896 -2.472976311 2.94E-14 8.56E-14

CDH10 0.083973174 0.028445285 -1.56173896 2.96E-14 8.60E-14

AC006330.1 0.057501172 0.205656263 1.838571736 2.98E-14 8.66E-14

RNU6-953P 0.018421721 0.49044612 4.734614911 2.99E-14 8.71E-14

MIR590 0.547023436 2.027765338 1.890216158 3.00E-14 8.71E-14

LINC01705 0.019412782 0.453374623 4.545624877 3.00E-14 8.72E-14

AL022238.3 0.099236905 0.379808316 1.936322852 3.04E-14 8.85E-14

ARGFXP2 0.022509121 0.132546678 2.557918897 3.05E-14 8.85E-14

AC092375.2 0.07211339 0.279618851 1.955122556 3.10E-14 9.01E-14

TAS2R19 0.077389849 0.258648084 1.740774264 3.12E-14 9.06E-14

AC008894.2 0.009721265 0.035866185 1.883408297 3.17E-14 9.19E-14

GAPDHP62 0.033882094 0.099702704 1.557109597 3.19E-14 9.25E-14

RPS3AP38 0.08204843 0.315895997 1.944902027 3.24E-14 9.39E-14

CDRT4 0.07250026 0.212584795 1.551980338 3.26E-14 9.46E-14

EPYC 0.006790116 0.218079226 5.005272369 3.28E-14 9.53E-14

SCN10A 0.00050246 0.008938502 4.152951601 3.29E-14 9.53E-14

AL365356.1 0.014903581 0.13410891 3.169674165 3.31E-14 9.58E-14

TMEM269 0.021510906 0.079422435 1.884478334 3.32E-14 9.63E-14

SCDP1 0.008019792 0.043241942 2.430794575 3.33E-14 9.66E-14

SLC34A1 38.70892885 1.961465431 -4.302662571 3.35E-14 9.71E-14

NXF5 0.019254262 0.08199615 2.090378381 3.38E-14 9.78E-14

AC130462.3 0.021521258 0.100235194 2.219554806 3.40E-14 9.83E-14

LINC02102 0.014419259 0.047451063 1.71844338 3.47E-14 1.00E-13

AC116552.1 0.065841347 0.324134164 2.299525334 3.53E-14 1.02E-13

AL117379.1 0.36918492 1.101465494 1.57700877 3.60E-14 1.04E-13

NCLP1 0.024335931 0.120303779 2.305522113 3.66E-14 1.06E-13

AC005274.1 0.049091158 0.254390025 2.373507011 3.66E-14 1.06E-13

IFIT1P1 0.003807936 0.024619014 2.692691847 3.69E-14 1.07E-13

RPL30P7 0.100184982 0.879925891 3.134715758 3.72E-14 1.08E-13

AP006621.2 0.441335212 1.854161364 2.070820041 3.74E-14 1.08E-13

SNORA66 0.432772066 1.605951953 1.891749444 3.75E-14 1.08E-13

TRPC7-AS2 0.096911204 0.016208384 -2.579923199 3.76E-14 1.09E-13

AP005131.5 0.01885711 0.277348897 3.878521507 3.78E-14 1.09E-13

CD1A 0.039832605 0.278854276 2.807489666 3.80E-14 1.10E-13

AC010325.1 0.016446861 0.089665815 2.446745842 3.80E-14 1.10E-13

RNU7-49P 0.559304349 2.073553924 1.890400113 3.80E-14 1.10E-13

AC011374.1 0.02957517 0.106335201 1.846160904 3.82E-14 1.10E-13

AC138470.1 0.095035199 0.269020731 1.501183493 3.82E-14 1.10E-13

AL358335.2 0.03608265 0.011227697 -1.684243222 3.84E-14 1.11E-13

AC104619.1 0.065868503 0.199751611 1.600546478 3.84E-14 1.11E-13

RNA5SP82 0.455530874 1.507539396 1.726574963 3.85E-14 1.11E-13

LINC00906 0.010717003 0.124430138 3.537362568 3.86E-14 1.11E-13

AC009090.1 0.253848084 0.813872623 1.680837652 3.89E-14 1.12E-13

CCN5 0.139120158 1.440516521 3.372182828 3.89E-14 1.12E-13

GTF2IP23 0.657663858 1.902858313 1.532745849 3.95E-14 1.14E-13

AP001020.3 0.033135758 0.127083223 1.939312744 3.97E-14 1.14E-13

TMC1 0.286988936 0.077673639 -1.885498156 3.97E-14 1.14E-13

CLEC18A 0.509953973 2.317215841 2.183953488 3.99E-14 1.15E-13

AC092139.3 0.006274563 0.082459373 3.716096499 4.03E-14 1.16E-13

SNORD93 0.077320328 0.630326737 3.027180201 4.08E-14 1.17E-13

RN7SL68P 0.02531729 0.157134415 2.633804287 4.11E-14 1.18E-13

LINC02227 0.00321477 0.025530503 2.989434637 4.12E-14 1.18E-13

AL049780.1 0.141855743 0.570798577 2.008557176 4.22E-14 1.21E-13

AC211433.1 0.022371733 0.150325806 2.748343741 4.23E-14 1.21E-13

DBIL5P2 0.027011496 0.084942191 1.65290779 4.23E-14 1.22E-13

AC092384.2 0.014684593 0.077118095 2.392766142 4.26E-14 1.22E-13

TBC1D3L 0.051281753 0.239612384 2.224184994 4.32E-14 1.24E-13

AL136234.1 0.209327326 0.056476965 -1.890026189 4.39E-14 1.26E-13

KRTAP17-1 0.083863237 0.017961126 -2.223160715 4.45E-14 1.28E-13

CACNA1I 0.010661362 0.036445466 1.773347547 4.50E-14 1.29E-13

ADAMTSL4-AS1 0.060322694 0.208730429 1.790868161 4.55E-14 1.30E-13

AC015982.1 0.174249153 0.714763043 2.036313397 4.63E-14 1.33E-13

AC138649.2 0.131129049 0.407555681 1.636009858 4.74E-14 1.36E-13

AC079313.2 0.020930698 0.0815219 1.961567244 4.79E-14 1.37E-13

TRGVA 0.019954278 0.111537995 2.482765254 4.80E-14 1.37E-13

BCL2L15 1.031817271 0.265193145 -1.960072109 4.81E-14 1.38E-13

DYNLL1P4 0.028849015 0.14898848 2.368606798 4.91E-14 1.40E-13

AL358933.1 0.099825952 0.031880941 -1.646720728 4.92E-14 1.41E-13

ASIC5 0.041914285 0.012131886 -1.788638139 4.95E-14 1.42E-13

SNORD62B 0.619861683 1.891764494 1.609714267 4.96E-14 1.42E-13

AL360182.1 0.007722757 0.12095338 3.969191234 5.01E-14 1.43E-13

AC023490.6 0.16679534 0.04675542 -1.834873467 5.04E-14 1.44E-13

MRPL37P1 0.021631085 0.141272723 2.707305029 5.05E-14 1.44E-13

NCBP2-AS1 0.067544097 0.234796234 1.797507666 5.07E-14 1.45E-13

LINC01281 0.003675191 0.048736263 3.729104438 5.44E-14 1.55E-13

AL513190.1 0.110530533 0.331572858 1.584880959 5.47E-14 1.56E-13

MUC20-OT1 0.875010683 2.629490607 1.587410807 5.48E-14 1.56E-13

AC090589.1 0.219372157 0.815535509 1.894367269 5.51E-14 1.57E-13

AL139811.1 0.027866148 0.098438479 1.820708783 5.56E-14 1.58E-13

BPIFB9P 0.022300595 0.105316894 2.239582799 5.59E-14 1.59E-13

HSPE1P18 0.054941676 0.315441312 2.521398793 5.70E-14 1.62E-13

CCDC78 0.077735483 0.538048185 2.791090193 5.72E-14 1.63E-13

AL583722.3 0.005942254 0.05556687 3.225142779 5.81E-14 1.65E-13

AL731563.2 0.020923057 0.263556299 3.654945635 5.90E-14 1.68E-13

RNU6-1160P 0.047059413 0.469974637 3.320027688 5.91E-14 1.68E-13

PVRIG 0.057095614 0.182208147 1.674135649 5.99E-14 1.70E-13

AGAP13P 0.131014963 0.571561446 2.125177015 6.01E-14 1.71E-13

SOX21-AS1 0.004601358 0.030358897 2.721987764 6.03E-14 1.71E-13

RN7SL127P 0.00988481 0.189176431 4.25837529 6.16E-14 1.75E-13

LINC02555 0.063150592 0.362904438 2.522721526 6.19E-14 1.76E-13

AC141002.1 0.183337668 0.541174402 1.561590372 6.20E-14 1.76E-13

RPL31P61 0.083182249 0.384420382 2.208337225 6.21E-14 1.77E-13

AC114939.1 0.127972185 0.439929126 1.781440849 6.48E-14 1.84E-13

SNORD63 0.362167521 1.54759488 2.095298784 6.49E-14 1.84E-13

AC025917.1 0.206882977 0.615589517 1.573153723 6.52E-14 1.85E-13

LINC00384 0.001323545 0.008206987 2.632445585 6.58E-14 1.87E-13

AC087311.1 0.056293396 0.01018832 -2.466049475 6.62E-14 1.88E-13

LPA 0.165093796 0.039139077 -2.076604282 6.62E-14 1.88E-13

HLA-DQA2 7.124018247 39.21800228 2.460752932 6.66E-14 1.89E-13

ROCR 0.014826187 0.095704241 2.690435258 6.73E-14 1.91E-13

AL136361.1 0.060091489 0.010432086 -2.526132956 6.77E-14 1.92E-13

MIR6719 0.041702235 0.409065868 3.294136564 6.78E-14 1.92E-13

RN7SL663P 0.082667577 0.335876811 2.022538689 6.79E-14 1.93E-13

AC069549.1 0.082428281 0.460471051 2.48189915 6.82E-14 1.93E-13

RPS7P3 0.073770041 0.239179528 1.696986958 6.83E-14 1.93E-13

AC087239.1 0.123115105 0.428898151 1.800627322 6.87E-14 1.95E-13

RN7SL558P 0.047308392 0.364864947 2.947194538 6.92E-14 1.96E-13

AC127070.4 0.060436082 0.244573509 2.016786108 7.07E-14 2.00E-13

AC089999.1 0.608504551 0.173298864 -1.812005858 7.23E-14 2.05E-13

AC092127.1 0.003324055 0.062743026 4.238438976 7.24E-14 2.05E-13

KRT18P57 0.007473638 0.100534411 3.74973486 7.30E-14 2.06E-13

LINC02172 0.473096934 0.016114632 -4.875692648 7.32E-14 2.07E-13

AC138466.5 0.021917948 0.12347203 2.493999594 7.34E-14 2.08E-13

AC012020.1 0.038837868 0.121526868 1.645739398 7.37E-14 2.08E-13

CALML3-AS1 0.201764567 0.045670678 -2.143332726 7.38E-14 2.09E-13

EIF4EP1 0.108532114 0.417804919 1.944707483 7.53E-14 2.13E-13

LINC00303 0.006465473 0.042495818 2.716492958 7.60E-14 2.15E-13

AC010761.3 0.182288929 0.653050153 1.840966849 7.63E-14 2.15E-13

H3P37 0.015468171 0.075394192 2.285150791 7.75E-14 2.19E-13

AC006065.4 0.001439881 0.045450931 4.980288517 7.81E-14 2.20E-13

SERPINA1 106.3144874 333.446596 1.649117516 7.83E-14 2.21E-13

CELA1 0.010233955 0.052934466 2.370843611 7.93E-14 2.24E-13

TRAV1-1 0.037999211 0.228868729 2.590478984 7.94E-14 2.24E-13

AL136982.1 0.027992719 0.100319838 1.841483399 8.11E-14 2.29E-13

ZNF812P 0.265407519 0.801803052 1.595038667 8.18E-14 2.30E-13

ATP1A3 0.105380447 0.529069591 2.327850293 8.22E-14 2.32E-13

RNU7-40P 0.233198582 1.551485173 2.734018989 8.23E-14 2.32E-13

LINC01237 0.068311596 0.196610994 1.525141605 8.27E-14 2.33E-13

AC007216.1 0.049884767 0.431083861 3.111297319 8.27E-14 2.33E-13

LHFPL5 0.003867964 0.043237373 3.48263256 8.34E-14 2.35E-13

AL512652.1 0.070394588 0.255079651 1.85741139 8.37E-14 2.35E-13

AC068888.2 0.066524203 0.282294995 2.085252315 8.44E-14 2.37E-13

TRBV7-4 0.033955945 0.282215242 3.055059823 8.58E-14 2.41E-13

RNU6-88P 0.200936085 1.121783265 2.48098539 8.67E-14 2.44E-13

AP001458.1 0.07706929 0.522679203 2.761697745 8.78E-14 2.47E-13

AC004678.1 0.028036024 0.101294003 1.853195086 8.83E-14 2.48E-13

AC007601.1 0.023922577 0.120116455 2.327989069 8.87E-14 2.49E-13

AL355388.1 0.092308192 0.315543922 1.77331024 8.94E-14 2.51E-13

TRIM74 0.020995791 0.096518711 2.200708485 9.14E-14 2.56E-13

TRAV20 0.092756593 0.475867866 2.359039294 9.24E-14 2.59E-13

AC005519.1 0.195832194 0.735491067 1.909089863 9.31E-14 2.61E-13

AL158166.2 0.059617772 0.17211889 1.529591083 9.46E-14 2.65E-13

AL354709.2 0.009936942 0.178165991 4.164276274 9.61E-14 2.69E-13

AC005614.1 0.01626509 0.005364412 -1.600286977 9.73E-14 2.72E-13

AC069281.1 0.098453782 0.030241223 -1.702930159 9.77E-14 2.74E-13

LINC01583 0.001352655 0.059527008 5.459678645 9.94E-14 2.78E-13

VIP 0.100782698 0.450943659 2.161699212 9.95E-14 2.78E-13

AL645608.8 0.059979217 0.216026617 1.848674493 9.98E-14 2.79E-13

SLC16A6P1 0.054498167 0.204975735 1.911173527 1.01E-13 2.82E-13

AC020907.5 0.010342682 0.108884961 3.396122438 1.02E-13 2.84E-13

TAS2R4 0.10803253 0.315483386 1.54609824 1.02E-13 2.86E-13

LYPLAL1-AS1 0.512683828 0.083274155 -2.622128676 1.03E-13 2.89E-13

AC006262.1 0.006589519 0.078617104 3.576598157 1.04E-13 2.89E-13

SNORD117 0.180261794 0.927368069 2.363048396 1.05E-13 2.92E-13

AC098934.2 0.692046812 0.115446574 -2.583644269 1.05E-13 2.93E-13

KIRREL2 0.380836298 0.073965964 -2.364237527 1.05E-13 2.94E-13

NRXN2-AS1 0.052704874 0.205531753 1.963353005 1.05E-13 2.94E-13

YJEFN3 0.272044113 1.205023804 2.147149135 1.07E-13 2.99E-13

AC010533.1 0.007399321 0.050679989 2.775951348 1.08E-13 3.02E-13

CDC37P2 0.021007766 0.108650521 2.370700449 1.09E-13 3.05E-13

RNU4ATAC18P 0.471305239 1.631738128 1.791675918 1.10E-13 3.07E-13

MIR509-3 0.045156225 0.472379984 3.386951048 1.10E-13 3.08E-13

AC008393.2 0.414007529 1.655987024 1.999962458 1.11E-13 3.11E-13

AC111182.1 0.021486029 0.09840963 2.195400638 1.12E-13 3.12E-13

LYG2 0.048534186 0.199871155 2.041997081 1.13E-13 3.14E-13

MOV10L1 0.041804696 0.122496704 1.551006004 1.13E-13 3.15E-13

NPHP3-ACAD11 0.003291221 0.01108029 1.75130087 1.13E-13 3.16E-13

JMJD7-PLA2G4B 0.175024358 0.531440104 1.602351382 1.14E-13 3.18E-13

AL355075.2 0.189104686 0.720110146 1.929032477 1.15E-13 3.20E-13

PPIAP45 0.039650163 0.468260028 3.561911182 1.17E-13 3.25E-13

AC234063.1 0.094960925 0.631985648 2.734485904 1.17E-13 3.26E-13

AC007496.1 0.012437263 0.23401342 4.233850386 1.17E-13 3.27E-13

HSD17B3-AS1 0.021357228 0.103845353 2.281640325 1.20E-13 3.33E-13

AL009181.2 0.017919739 0.064409103 1.845715015 1.21E-13 3.38E-13

AC002066.1 0.043866007 0.136264743 1.635237044 1.22E-13 3.40E-13

CSAD 1.590289622 4.768674093 1.584298656 1.24E-13 3.46E-13

AL031658.1 0.081078745 0.291021863 1.843731877 1.25E-13 3.48E-13

TPBGL 0.118207265 0.58470706 2.306395304 1.26E-13 3.51E-13

AC020913.3 0.074073049 0.61797341 3.060524138 1.27E-13 3.52E-13

SENP3-EIF4A1 0.007320989 0.02553759 1.802511832 1.31E-13 3.64E-13

AL109976.1 0.754934557 0.184213344 -2.034974012 1.31E-13 3.64E-13

MIR509-2 0.020975217 0.358844878 4.09660271 1.32E-13 3.65E-13

AC080097.1 1.085882704 0.156923679 -2.790733303 1.34E-13 3.71E-13

CD163L1 0.458637876 1.333879941 1.540201412 1.34E-13 3.72E-13

AC008667.3 0.015408311 0.102510633 2.733992892 1.34E-13 3.72E-13

RNVU1-3 0.159660863 0.746680368 2.225480087 1.34E-13 3.72E-13

AC087741.1 0.311494034 1.167836086 1.906561358 1.35E-13 3.74E-13

AC243772.3 0.00768823 0.044195459 2.523174707 1.36E-13 3.76E-13

AC097382.1 0.015314615 0.173449334 3.501533324 1.37E-13 3.80E-13

LINC00165 0.013443698 0.062780207 2.223379758 1.38E-13 3.82E-13

AC069503.2 0.027040715 0.101363582 1.906334215 1.41E-13 3.90E-13

AC012313.10 0.044571489 0.153519824 1.784231895 1.41E-13 3.91E-13

LINC00161 0.00986138 0.062969739 2.674797207 1.44E-13 3.97E-13

AL672207.1 0.969931827 0.310124963 -1.645033692 1.44E-13 3.98E-13

AC008556.1 1.932685154 0.639892982 -1.594704085 1.44E-13 3.98E-13

LINC00313 0.003037873 0.029040736 3.256944609 1.44E-13 3.99E-13

AC092338.1 0.111578252 0.428301333 1.940570306 1.49E-13 4.12E-13

TRAV8-1 0.078945269 0.38536212 2.287290054 1.50E-13 4.14E-13

LINC01832 0.006800184 0.080316576 3.562052073 1.52E-13 4.18E-13

AC008750.5 0.081667123 0.277560328 1.764974069 1.53E-13 4.22E-13

LINC02499 1.062363146 0.093727066 -3.50266747 1.53E-13 4.24E-13

SCARNA8 0.054421215 0.543763665 3.320738681 1.54E-13 4.26E-13

AC087854.2 0.060331085 0.225822624 1.904216597 1.55E-13 4.27E-13

AC097713.2 0.013356605 0.121602268 3.186544872 1.55E-13 4.28E-13

NCF4-AS1 0.0008617 0.019480769 4.498720557 1.55E-13 4.28E-13

AC073912.3 0.050980679 0.277327436 2.443567861 1.59E-13 4.38E-13

KLHDC8A 0.262609357 0.836617986 1.671650694 1.59E-13 4.39E-13

AC091516.1 0.039302992 0.148210947 1.914940951 1.61E-13 4.43E-13

AC138932.6 0.194844928 0.569217787 1.546654366 1.64E-13 4.53E-13

AC007383.4 0.121167056 0.628173621 2.374165866 1.67E-13 4.59E-13

SMCO2 0.034392028 0.115258409 1.744725933 1.67E-13 4.60E-13

GAPDHP39 0.008610256 0.075521245 3.132754533 1.68E-13 4.63E-13

EGFL8 0.203743336 0.776781758 1.930756449 1.69E-13 4.65E-13

TBX4 0.012065409 0.043765947 1.858931938 1.69E-13 4.66E-13

GCNT7 0.041419845 0.149935762 1.855950471 1.70E-13 4.67E-13

AL109741.2 0.069847908 0.268675661 1.943576814 1.70E-13 4.68E-13

ENTHD1 0.007733233 0.035585324 2.202138733 1.75E-13 4.81E-13

FAAHP1 0.164335821 0.69019275 2.070352338 1.75E-13 4.82E-13

AC134407.2 0.016395324 0.073413183 2.162754699 1.76E-13 4.85E-13

STRC 0.002808395 0.020009028 2.832833483 1.77E-13 4.86E-13

AC131212.2 0.124790647 0.437859981 1.810959789 1.82E-13 4.99E-13

STEAP3-AS1 0.029062661 0.12343569 2.086520878 1.85E-13 5.07E-13

MIR3941 0.10197068 0.785412746 2.94529662 1.87E-13 5.12E-13

ACTG1P22 0.00349347 0.035317657 3.3376571 1.87E-13 5.13E-13

TLR12P 0.006062531 0.022716717 1.905762295 1.91E-13 5.25E-13

MELTF 7.190304461 1.172231897 -2.616794861 1.96E-13 5.37E-13

AC010999.1 0.081502844 0.255947326 1.650924632 2.02E-13 5.52E-13

AL353898.3 0.114663192 0.376761949 1.716250917 2.03E-13 5.57E-13

INTS4P1 0.052147013 0.203051825 1.961191488 2.04E-13 5.59E-13

AC009148.1 0.032591153 0.130331417 1.999632581 2.06E-13 5.65E-13

AL355376.2 1.697040675 0.185066188 -3.1969079 2.07E-13 5.68E-13

AC063977.1 0.012365569 0.114766161 3.214296824 2.10E-13 5.74E-13

AL109933.4 0.557709988 0.078129282 -2.835579841 2.10E-13 5.75E-13

AL133230.2 0.080193864 0.317512581 1.985250003 2.12E-13 5.81E-13

AC012404.2 0.034536514 0.49043401 3.827864671 2.12E-13 5.81E-13

LINC01675 0.01059199 0.075173623 2.827252822 2.13E-13 5.83E-13

COX6B1P5 0.076609555 0.306002067 1.997945159 2.14E-13 5.84E-13

BAGE2 0.03271802 0.007373341 -2.149695039 2.16E-13 5.92E-13

LINC00838 0.000156171 0.008394142 5.748180364 2.18E-13 5.97E-13

TEX15 0.010777577 0.606018266 5.813256484 2.19E-13 5.99E-13

VN1R12P 0.020398203 0.131762082 2.691421301 2.21E-13 6.04E-13

RN7SL704P 0.026148582 0.188739827 2.851594267 2.21E-13 6.05E-13

AL590385.2 0.011357678 0.088153541 2.956350541 2.22E-13 6.08E-13

RNU6-1157P 0.152090689 0.636464629 2.065148508 2.24E-13 6.12E-13

MROH9 0.001572046 0.024519372 3.963206806 2.25E-13 6.14E-13

AC055811.2 0.021053658 0.066746354 1.664618077 2.27E-13 6.19E-13

AL513523.3 0.046038725 0.194477706 2.078685006 2.27E-13 6.20E-13

AC025043.1 0.060718596 0.202106019 1.734901957 2.27E-13 6.21E-13

JSRP1 0.140938841 0.75687628 2.42498824 2.27E-13 6.21E-13

PGLYRP2 0.011587677 0.20144606 4.119730289 2.27E-13 6.21E-13

H4C10P 0.070470797 0.339651082 2.268956017 2.31E-13 6.30E-13

AC106800.1 0.01605582 0.051649981 1.685671473 2.32E-13 6.34E-13

AC244517.3 0.02396615 0.10050173 2.068150247 2.33E-13 6.37E-13

GAPDHP73 0.027662429 0.084042649 1.603193795 2.36E-13 6.44E-13

AC036103.1 0.050782105 0.17718382 1.802854766 2.36E-13 6.44E-13

DCDC2B 0.060301009 0.23508126 1.962905484 2.37E-13 6.47E-13

NLRP2B 0.012501467 0.039374234 1.655154454 2.39E-13 6.51E-13

AC011472.1 0.705543357 2.137253438 1.598951347 2.40E-13 6.53E-13

LINC02539 0.000688343 0.043389046 5.978059027 2.44E-13 6.65E-13

AL359851.1 0.00179824 0.014892692 3.049947361 2.46E-13 6.69E-13

GJA1P1 0.010908843 0.062385297 2.515707972 2.46E-13 6.70E-13

PRMT1P1 0.022630258 0.081885673 1.855358044 2.52E-13 6.85E-13

AP000907.2 0.099392701 0.406925167 2.033551696 2.52E-13 6.86E-13

LINC00685 0.452585078 1.358990851 1.586274821 2.52E-13 6.86E-13

STK19B 0.474415655 1.743404859 1.877684113 2.54E-13 6.90E-13

AL355303.1 0.004016499 0.053502581 3.735597968 2.58E-13 7.01E-13

OR11H7 0.021582671 0.090892528 2.074288267 2.60E-13 7.06E-13

SCGB1C1 0.071724437 0.02098635 -1.773013498 2.62E-13 7.13E-13

AC107993.1 0.042470164 0.196485374 2.209900355 2.64E-13 7.17E-13

AC013391.3 0.002417691 0.092416349 5.256446105 2.68E-13 7.27E-13

AZGP1P1 0.768323084 0.117749617 -2.705990738 2.70E-13 7.34E-13

RPL21P44 0.087672888 0.288016798 1.715950279 2.71E-13 7.35E-13

PLEKHG4B 0.858621018 0.191054602 -2.168036481 2.72E-13 7.39E-13

SCGB3A1 0.2821962 0.891585871 1.659675191 2.78E-13 7.54E-13

Z73965.1 0.07249653 0.206531459 1.510377709 2.79E-13 7.58E-13

AC069503.3 0.09989915 0.031349039 -1.672051168 2.80E-13 7.59E-13

AL049840.7 0.282955951 0.865366204 1.6127333 2.81E-13 7.62E-13

PAEP 0.010618538 6.658253526 9.292414907 2.82E-13 7.65E-13

AC093311.1 0.018000796 0.084210536 2.225940046 2.82E-13 7.66E-13

GFRA3 0.279951049 0.095014652 -1.558952674 2.84E-13 7.71E-13

EPGN 0.38085691 0.044179454 -3.107801565 2.85E-13 7.72E-13

MIR509-1 0.018223036 0.317926874 4.124859723 2.86E-13 7.74E-13

RPL10P12 0.079287135 0.376699495 2.248255394 2.87E-13 7.77E-13

AC020910.3 0.006137012 0.090192558 3.87740017 2.92E-13 7.90E-13

SNORA2A 0.230062549 1.081580297 2.23304272 2.94E-13 7.97E-13

AC013553.1 0.02185241 0.174141972 2.994399679 2.98E-13 8.07E-13

UPK3BP1 0.004633081 0.064278047 3.794282295 2.99E-13 8.08E-13

Z83844.2 0.009643414 0.043477743 2.172661156 3.00E-13 8.12E-13

AC023830.3 0.042601516 0.171931993 2.012861353 3.03E-13 8.20E-13

AL031705.1 0.052757477 0.221974166 2.072944304 3.04E-13 8.22E-13

AC090772.1 0.020852185 0.115169415 2.465487188 3.05E-13 8.23E-13

AC005899.6 0.030520736 0.184097901 2.592611503 3.09E-13 8.34E-13

FUT3 2.890358762 0.618730985 -2.223864387 3.12E-13 8.44E-13

AC008734.1 0.026085514 0.128312114 2.298336604 3.12E-13 8.44E-13

ASS1P9 0.12705673 0.019727782 -2.687172135 3.14E-13 8.48E-13

BEAN1-AS1 0.023407821 0.108396583 2.211256711 3.21E-13 8.66E-13

RN7SL130P 0.269914912 0.844187494 1.645058776 3.21E-13 8.67E-13

AP001056.1 0.015555281 0.08752935 2.492362439 3.23E-13 8.73E-13

FERP1 0.066980373 0.209422478 1.644605978 3.24E-13 8.74E-13

KLRF2 0.010730337 0.112963795 3.396093164 3.27E-13 8.83E-13

NPFFR1 0.015529719 0.09221751 2.570008944 3.29E-13 8.87E-13

KRT84 0.000497899 0.011791639 4.565767433 3.29E-13 8.88E-13

AC089984.2 0.019219955 0.146055646 2.925841262 3.32E-13 8.97E-13

TREML3P 0.011487859 0.08597059 2.90373327 3.33E-13 8.97E-13

AL445471.2 0.000475773 0.016139921 5.084215743 3.33E-13 8.97E-13

AC007278.2 0.004808063 0.034688633 2.850935332 3.36E-13 9.05E-13

TSKS 0.033997054 0.098552505 1.535482787 3.36E-13 9.05E-13

Z82188.2 0.030841083 0.117274088 1.926958951 3.41E-13 9.18E-13

AC008991.1 0.02153308 0.409407659 4.248911476 3.46E-13 9.33E-13

MIR513C 0.016679322 0.376634347 4.497032003 3.47E-13 9.36E-13

AL158151.2 0.030332532 0.178059275 2.55341977 3.48E-13 9.37E-13

LINC02078 0.003690892 0.036641448 3.311435264 3.56E-13 9.59E-13

RPS2P35 0.041582867 0.132425614 1.671121061 3.57E-13 9.60E-13

AC144548.1 0.08271539 0.250557785 1.598915674 3.59E-13 9.67E-13

IFNG-AS1 0.023077246 0.114321694 2.308556201 3.66E-13 9.83E-13

AC121247.2 0.049855498 0.165117869 1.727671746 3.69E-13 9.93E-13

AC100827.4 0.007567111 0.035753294 2.240261655 3.69E-13 9.93E-13

AC091180.5 0.047962231 0.223186734 2.218280597 3.70E-13 9.94E-13

ANKRD63 0.062562781 0.012724463 -2.297699866 3.70E-13 9.96E-13

EFCAB8 0.007233621 0.046932144 2.69778647 3.78E-13 1.02E-12

Z69720.1 0.011354693 0.061096316 2.427796727 3.79E-13 1.02E-12

NPIPA3 0.025420065 0.141391356 2.475654302 3.83E-13 1.03E-12

AC073150.1 0.020903888 0.091134192 2.124221104 3.85E-13 1.03E-12

AL031772.1 0.011332333 0.049595208 2.129755816 3.87E-13 1.04E-12

RNF138P1 0.088036688 0.434767778 2.304068252 3.91E-13 1.05E-12

AL583722.1 0.02777797 0.100957647 1.861737121 3.96E-13 1.06E-12

AL512791.1 0.66579777 1.915916058 1.52487841 3.98E-13 1.07E-12

AC044840.1 0.034367148 0.36437406 3.40631824 4.00E-13 1.07E-12

BIN2P1 0.005381154 0.023549136 2.129686744 4.02E-13 1.08E-12

BMS1P22 0.002138748 0.02386684 3.480169314 4.05E-13 1.09E-12

AC011461.1 0.062127226 0.288901464 2.217279974 4.07E-13 1.09E-12

AC131009.4 0.054949045 0.278329513 2.340627593 4.10E-13 1.10E-12

IL21-AS1 0.00602366 0.112169448 4.218895677 4.14E-13 1.11E-12

NMUR2 0.303846941 0.037966184 -3.000557852 4.15E-13 1.11E-12

AC025176.1 0.039396474 0.213450139 2.437760686 4.17E-13 1.12E-12

AC007998.4 0.011026979 0.033270314 1.593197898 4.19E-13 1.12E-12

AL118523.1 0.057743838 0.016328237 -1.822297933 4.20E-13 1.12E-12

BNIP3P22 0.053897708 0.54748761 3.344530485 4.22E-13 1.13E-12

AL645949.1 0.017499884 0.081530443 2.219993455 4.25E-13 1.14E-12

RN7SL648P 0.02225423 0.683920905 4.941677999 4.28E-13 1.15E-12

PGBD4P1 0.009529675 0.076355569 3.002234442 4.30E-13 1.15E-12

AC021146.11 0.038174318 0.012751488 -1.581936728 4.33E-13 1.16E-12

AC092436.1 0.054768264 0.016034651 -1.772147245 4.44E-13 1.19E-12

CC2D2B 0.034396232 0.113698643 1.724892609 4.47E-13 1.20E-12

AC129929.1 0.908863159 3.077405339 1.75957948 4.60E-13 1.23E-12

G6PC 29.90980479 2.819185278 -3.407268294 4.61E-13 1.23E-12

RPL9P32 0.091462624 0.304001418 1.732823834 4.61E-13 1.23E-12

PLA2G4B 0.035649303 0.133304999 1.902785123 4.61E-13 1.23E-12

RPL12P15 0.053257462 0.154049214 1.532335736 4.65E-13 1.24E-12

CCL25 0.010206668 0.098646997 3.272763129 4.67E-13 1.25E-12

IFNL1 0.007711728 0.06331201 3.037353121 4.69E-13 1.25E-12

AC009093.7 0.006751822 0.051242509 2.923992286 4.75E-13 1.27E-12

AC012254.5 0.124196448 0.470878556 1.92273111 4.75E-13 1.27E-12

ASB9P1 0.098385201 0.384565615 1.966716551 4.85E-13 1.29E-12

AL109933.1 0.304315383 0.031089931 -3.291047933 4.86E-13 1.30E-12

AD001527.1 0.127593695 0.576003892 2.174521519 4.91E-13 1.31E-12

VXN 0.32265325 1.042066362 1.691390692 4.93E-13 1.31E-12

GRM8 0.358724128 1.490924662 2.055260669 4.93E-13 1.31E-12

SNORA3B 0.100526771 0.603037111 2.58466703 4.94E-13 1.32E-12

AC092145.1 0.008812685 0.049663581 2.49453474 5.03E-13 1.34E-12

RPS12P20 0.018934831 0.154475416 3.028262809 5.05E-13 1.34E-12

GOLGA8B 0.635754207 2.620307422 2.043195074 5.06E-13 1.35E-12

TMPRSS6 0.069317915 0.295344512 2.091098637 5.06E-13 1.35E-12

TMPRSS11CP 0.06110075 0.259198268 2.084794075 5.12E-13 1.36E-12

LINC01901 0.001755869 0.023624099 3.75000218 5.19E-13 1.38E-12

FOXP3 0.366837174 1.168878911 1.671913732 5.20E-13 1.38E-12

TEX29 0.098517918 0.351736245 1.836035965 5.25E-13 1.40E-12

AC103982.1 0.078885638 0.268765557 1.768513701 5.36E-13 1.42E-12

AC102953.1 0.046205551 0.14746633 1.674247501 5.37E-13 1.43E-12

MIR4292 0.837193114 2.560691853 1.612901303 5.44E-13 1.45E-12

BDNF 0.143576146 0.633485553 2.141495643 5.45E-13 1.45E-12

AC020604.1 0.043596139 0.145933493 1.74303876 5.49E-13 1.46E-12

TTLL3 0.590351371 1.842802471 1.642255646 5.53E-13 1.47E-12

MAPK15 1.030271925 3.960718847 1.942737129 5.53E-13 1.47E-12

AP001107.4 0.200963108 0.762355744 1.923533694 5.62E-13 1.49E-12

AC008443.6 0.033312973 0.112983642 1.761957887 5.70E-13 1.51E-12

AL133243.4 0.008961162 0.077871541 3.119338458 5.72E-13 1.52E-12

AP002812.2 0.047056013 0.175627645 1.900068965 5.78E-13 1.53E-12

RN7SL431P 0.059403953 0.300717317 2.339777113 5.81E-13 1.54E-12

AC093484.1 0.016952175 0.08886636 2.390167004 5.83E-13 1.54E-12

AC008740.1 0.056313335 0.302825295 2.426937224 5.84E-13 1.55E-12

NARF-AS1 0.02236863 0.096504847 2.109124481 5.88E-13 1.56E-12

AC024940.2 0.008827406 0.03175797 1.847057211 5.97E-13 1.58E-12

RNA5SP108 0.09527334 0.387099108 2.022558507 5.99E-13 1.59E-12

AC093484.4 0.161109073 0.557765226 1.791620251 6.04E-13 1.60E-12

LINC02313 0.166041423 0.547806964 1.72212441 6.21E-13 1.64E-12

RNF14P3 0.030034817 0.12966931 2.11012927 6.23E-13 1.65E-12

RNU6-1280P 0.157872763 0.936596579 2.568665475 6.25E-13 1.65E-12

AC002044.2 0.040551311 0.233044502 2.522785021 6.27E-13 1.66E-12

AC078777.1 0.053950289 0.176475942 1.709768913 6.29E-13 1.66E-12

AC016747.3 0.119158707 0.378848627 1.668737151 6.32E-13 1.67E-12

AC004930.1 0.025189392 0.316404311 3.650881045 6.34E-13 1.67E-12

SULT2B1 3.078149876 0.324451164 -3.245990234 6.37E-13 1.68E-12

AL807752.4 0.006482652 0.074064043 3.514117288 6.38E-13 1.69E-12

AL450468.2 0.021531214 0.007550596 -1.511767138 6.39E-13 1.69E-12

AC008147.4 0.01214783 0.082158781 2.757716188 6.47E-13 1.71E-12

KLK14 0.044285521 0.185001217 2.062627753 6.50E-13 1.72E-12

LINC00955 0.567989473 0.078898096 -2.847801806 6.53E-13 1.73E-12

PCDH11X 0.016677483 0.004224997 -1.98087939 6.54E-13 1.73E-12

ANK1 0.244951047 1.192060163 2.282891686 6.57E-13 1.73E-12

NRXN2 1.134871013 3.371971907 1.571064184 6.61E-13 1.74E-12

H2BC9 0.137485524 0.841267551 2.613284973 6.63E-13 1.75E-12

RPL31P11 0.01436879 0.053070055 1.884959471 6.64E-13 1.75E-12

SPINK4 0.196816392 0.063661172 -1.628364758 6.65E-13 1.75E-12

LINC02416 0.018016471 0.196361636 3.446124742 6.71E-13 1.77E-12

LINC00519 0.061225588 0.205096254 1.744094511 6.90E-13 1.82E-12

AC019117.2 0.131656975 0.788094498 2.581584676 6.95E-13 1.83E-12

FAM214BP1 0.00080305 0.015618453 4.281617368 6.99E-13 1.84E-12

PPIAP72 0.066204868 0.222016041 1.745654701 7.04E-13 1.85E-12

AC025278.1 0.002410625 0.048705489 4.336605459 7.13E-13 1.88E-12

AC114495.2 0.206934065 0.666247321 1.686886671 7.14E-13 1.88E-12

AL356124.2 0.037137216 0.191145088 2.363730563 7.19E-13 1.89E-12

AC010894.4 0.011549367 0.096339786 3.060317923 7.21E-13 1.90E-12

AC017104.3 0.00861566 0.094435606 3.454297739 7.38E-13 1.94E-12

AC002543.1 0.005762411 0.080510656 3.804435208 7.42E-13 1.95E-12

FRZB 14.50754547 45.83801213 1.659741029 7.56E-13 1.99E-12

LINC01907 0.019425152 0.05614496 1.531230641 7.64E-13 2.01E-12

LINC02422 0.030473105 0.139080588 2.190312644 7.68E-13 2.02E-12

AC136475.8 0.010370031 0.079876512 2.945351089 7.69E-13 2.02E-12

TRGV6 0.016053139 0.102597196 2.676063945 7.72E-13 2.03E-12

LINC02544 0.078981742 0.334781267 2.083627705 7.73E-13 2.03E-12

UBA52P5 0.191854102 0.553214852 1.527830275 7.74E-13 2.03E-12

AC020913.2 0.009496009 0.085286188 3.166918866 7.92E-13 2.08E-12

ORM2 0.076579212 2.804391432 5.194591116 7.94E-13 2.08E-12

AC018797.1 0.027020553 0.165525103 2.614920907 7.95E-13 2.09E-12

RPL23AP93 0.008632185 0.098859722 3.517585165 7.96E-13 2.09E-12

KRT86 0.244828783 0.700315552 1.516231951 8.05E-13 2.11E-12

RPL21P123 0.030359801 0.234279826 2.947998495 8.11E-13 2.13E-12

MIR5188 0.087557131 0.378108294 2.110502918 8.22E-13 2.15E-12

AC110619.1 0.196583255 0.067861779 -1.534469271 8.23E-13 2.16E-12

ADAMTS18 0.13645773 0.386673367 1.502661276 8.30E-13 2.18E-12

ZBTB40-IT1 0.110619764 0.368959642 1.737853841 8.30E-13 2.18E-12

AC135983.5 0.006183818 0.042155892 2.769164428 8.36E-13 2.19E-12

AP001381.1 0.026085639 0.229153372 3.134985826 8.46E-13 2.22E-12

RPS2P45 0.006858157 0.065454393 3.254597217 8.47E-13 2.22E-12

AL031320.1 0.008902689 0.072156399 3.018814264 8.60E-13 2.25E-12

TRBJ2-4 0.030405794 0.38169232 3.649991968 8.92E-13 2.33E-12

AC010978.1 0.015487075 0.079605978 2.361812087 9.02E-13 2.36E-12

AL032821.1 0.006910818 0.067800995 3.294377991 9.13E-13 2.39E-12

FOXC2-AS1 0.099378763 0.724179465 2.865337772 9.22E-13 2.41E-12

TRBV21-1 0.02927303 0.257227868 3.135402971 9.27E-13 2.42E-12

AC020916.1 3.358283921 12.75118897 1.924835665 9.40E-13 2.46E-12

LINC01842 0.049247193 0.191920401 1.962394684 9.63E-13 2.52E-12

RPL29P24 0.099923503 0.293993226 1.556886963 9.65E-13 2.52E-12

TRAJ1 0.021891544 0.437100626 4.319519799 9.74E-13 2.54E-12

AP001893.2 0.008608249 0.11188759 3.700186356 9.80E-13 2.56E-12

SNORA5A 0.278544208 1.907501918 2.775706286 9.86E-13 2.57E-12

AC015853.1 0.031769267 0.142016486 2.160354708 9.92E-13 2.59E-12

NFE4 0.027847185 0.483342128 4.117441358 9.97E-13 2.60E-12

AC009779.5 0.033661186 0.205437582 2.609542234 1.00E-12 2.61E-12

AC021818.1 0.003440665 0.06150428 4.159927579 1.00E-12 2.61E-12

FRMD1 1.282117849 0.338503478 -1.921286314 1.01E-12 2.63E-12

Z83843.1 0.54655055 1.609296565 1.558003373 1.02E-12 2.65E-12

AC010201.2 0.34135982 1.024145501 1.585055536 1.02E-12 2.65E-12

OR10AB1P 0.632076137 0.055600049 -3.506940282 1.02E-12 2.65E-12

RN7SL22P 0.002795615 0.127061075 5.50621248 1.03E-12 2.68E-12

AC025884.1 0.148498748 0.501695188 1.756360331 1.03E-12 2.69E-12

AC025627.1 0.300878241 1.372503166 2.189557797 1.03E-12 2.69E-12

AL031600.1 0.195917986 0.820075824 2.065507457 1.04E-12 2.70E-12

CLRN1-AS1 0.005357211 0.023659324 2.142854698 1.04E-12 2.71E-12

LINC01841 0.035594828 0.13265185 1.897905276 1.04E-12 2.72E-12

POU5F1P4 0.009311622 0.076922686 3.046304728 1.05E-12 2.73E-12

AC097634.1 0.032233431 0.132044202 2.034391288 1.05E-12 2.74E-12

AC107373.1 0.014630242 0.090857325 2.634649177 1.06E-12 2.75E-12

VDAC2P3 0.360951374 0.031843162 -3.502749009 1.06E-12 2.75E-12

ACSM4 0.023333466 0.09044103 1.954576784 1.06E-12 2.76E-12

AC011816.2 0.023414889 0.09414144 2.007403728 1.06E-12 2.76E-12

AC025262.1 0.031962365 0.092877311 1.538952041 1.06E-12 2.77E-12

RNU6-1098P 0.206832596 0.83792283 2.018353817 1.06E-12 2.77E-12

LINC02698 0.003316878 0.060580347 4.190952074 1.06E-12 2.77E-12

AC026471.5 0.01760983 0.114099761 2.695842847 1.07E-12 2.79E-12

AC245884.11 0.009529228 0.040690874 2.094274052 1.07E-12 2.79E-12

AC105450.1 0.005379614 0.056577709 3.394659221 1.07E-12 2.79E-12

TRAV38-1 0.050240006 0.288100635 2.5196643 1.08E-12 2.80E-12

NPIPB10P 0.008244396 0.036550859 2.148419597 1.08E-12 2.80E-12

AC100827.2 0.026488705 0.139467735 2.396482188 1.09E-12 2.82E-12

AC244197.2 0.120918853 0.664160648 2.45749305 1.09E-12 2.83E-12

AC015727.1 0.11786545 0.403973364 1.777119293 1.10E-12 2.84E-12

AC004837.2 0.080245995 0.270365897 1.752411882 1.10E-12 2.86E-12

AP000355.1 0.439309797 1.590784216 1.856427574 1.11E-12 2.88E-12

AC018638.7 0.248297049 0.730513764 1.556844342 1.12E-12 2.91E-12

HLA-K 1.95583844 5.756490098 1.557402223 1.12E-12 2.91E-12

AC032044.1 0.070081025 0.229369918 1.710580413 1.13E-12 2.92E-12

CTAGE7P 0.06181557 0.194438699 1.653273214 1.15E-12 2.98E-12

UGT1A2P 0.022626955 0.154616329 2.772578311 1.15E-12 2.99E-12

AGXT 3.632917127 0.822009876 -2.143900824 1.16E-12 3.01E-12

TAS2R10 0.048167983 0.139751456 1.536716882 1.17E-12 3.03E-12

AC015818.2 0.009767809 0.114170722 3.547013964 1.18E-12 3.06E-12

AC084026.1 0.002844933 0.063562434 4.48170761 1.20E-12 3.10E-12

AC009120.3 0.490426906 1.394818293 1.507967155 1.20E-12 3.10E-12

AC073912.2 0.053622199 0.230550012 2.104177437 1.20E-12 3.11E-12

AC053513.2 0.216829319 0.652253033 1.5888719 1.20E-12 3.12E-12

AL592431.2 0.019004603 0.176959648 3.218999622 1.21E-12 3.13E-12

AC078778.1 0.02973299 0.142209608 2.25788249 1.22E-12 3.16E-12

RPL10P9 4.381459824 15.3122633 1.805204007 1.24E-12 3.20E-12

POSTN 7.891605806 23.75144517 1.589624499 1.25E-12 3.23E-12

AC087623.1 0.184244466 0.670013843 1.862569616 1.27E-12 3.28E-12

AC011444.2 0.018619596 0.132804314 2.834408328 1.29E-12 3.32E-12

RPL12P42 0.065960138 0.199158945 1.59425395 1.29E-12 3.33E-12

HERC2P2 0.77392344 2.547209883 1.718655077 1.30E-12 3.36E-12

RNU6-1016P 0.840441776 3.055479543 1.86217904 1.30E-12 3.36E-12

LINC00939 0.042955835 0.164089879 1.933560247 1.31E-12 3.38E-12

AC105339.5 0.060303246 0.238036236 1.98087364 1.32E-12 3.41E-12

RNU4-5P 0.04106436 0.239023358 2.54119288 1.37E-12 3.53E-12

AC008635.2 0.032848869 0.116110791 1.821586472 1.37E-12 3.54E-12

AC022092.1 0.013431718 0.092119217 2.777858249 1.37E-12 3.54E-12

HMGB3P4 0.04280735 0.182197408 2.08957201 1.38E-12 3.55E-12

RNU6-431P 0.123169206 0.48117558 1.965921817 1.39E-12 3.57E-12

AL513534.2 0.709766756 2.271262653 1.678077644 1.39E-12 3.58E-12

AC026469.1 0.242328392 0.074628758 -1.699159872 1.40E-12 3.60E-12

HECW1 1.49880001 0.121962045 -3.619303741 1.40E-12 3.60E-12

AC105429.1 0.036551035 0.154121864 2.076087354 1.40E-12 3.60E-12

AL357497.1 0.02508713 0.162593934 2.696254115 1.43E-12 3.69E-12

HNRNPA1P49 0.090898561 0.295004595 1.698408068 1.44E-12 3.70E-12

AL355796.1 0.038769555 0.369023084 3.250714981 1.45E-12 3.72E-12

AC073324.1 0.001197258 0.035880953 4.90541231 1.45E-12 3.72E-12

AC022558.1 0.12288348 0.3631507 1.563277379 1.47E-12 3.78E-12

ARG1 0.056402044 0.281505714 2.319344853 1.47E-12 3.79E-12

CCNYL4 0.006383648 0.032289413 2.338608214 1.48E-12 3.81E-12

TRAV8-5 0.028466826 0.143607478 2.334777344 1.49E-12 3.83E-12

AP002989.1 0.022517629 0.089287295 1.987399947 1.50E-12 3.85E-12

AP001107.8 0.065399632 0.397667216 2.6042072 1.52E-12 3.90E-12

CASK-AS1 0.033727795 0.136439759 2.016254195 1.54E-12 3.95E-12

DLK1 0.280828639 0.035314676 -2.991350314 1.55E-12 3.98E-12

LCT-AS1 0.005554669 0.017654853 1.668291849 1.56E-12 4.00E-12

AL391095.2 0.0442786 0.159725736 1.850915263 1.59E-12 4.09E-12

RRM2P3 0.170691252 0.576209689 1.755204794 1.61E-12 4.12E-12

GAPDHP51 0.003704504 0.062404555 4.074298875 1.62E-12 4.15E-12

AC068050.1 0.007002728 0.105315525 3.910657344 1.62E-12 4.17E-12

AL356299.3 0.079288104 0.278488203 1.812439889 1.63E-12 4.18E-12

GRAMD4P3 0.00058428 0.028919499 5.629238673 1.65E-12 4.23E-12

ACP4 0.022693179 0.070075785 1.62665731 1.67E-12 4.27E-12

SMIM23 0.006248174 0.081525808 3.705750294 1.68E-12 4.31E-12

AC035139.1 0.017698454 0.143307541 3.017419248 1.69E-12 4.34E-12

RPS12P16 0.010943952 0.244384028 4.480944311 1.72E-12 4.40E-12

AC068533.3 0.053452652 0.205213151 1.940789752 1.72E-12 4.41E-12

LINC01526 0.005452416 0.055156576 3.338565463 1.73E-12 4.43E-12

AC091729.1 0.022181581 0.097082433 2.129848073 1.76E-12 4.52E-12

MIR3176 0.405845286 1.697479296 2.064392217 1.79E-12 4.58E-12

SALRNA2 0.012900362 0.086075893 2.738197685 1.83E-12 4.69E-12

AC010619.3 0.012551377 0.044725466 1.833250816 1.87E-12 4.77E-12

SETP20 0.031992378 0.103207151 1.689742786 1.87E-12 4.78E-12

ANKRD44-IT1 0.01702199 0.204955585 3.589839648 1.88E-12 4.80E-12

AP001020.2 0.032547081 0.121127998 1.895932307 1.89E-12 4.83E-12

SLITRK5 0.034597019 0.377350013 3.447183667 1.90E-12 4.85E-12

AL157770.1 0.042023838 0.125968441 1.583782506 1.91E-12 4.88E-12

NPIPA9 0.008321257 0.030131001 1.856375193 1.92E-12 4.90E-12

CCDC154 0.074344323 0.372737856 2.325866862 1.93E-12 4.92E-12

AC116021.1 0.01128002 0.086769396 2.943416675 1.94E-12 4.95E-12

AC074124.1 0.103218206 0.722236819 2.806774507 1.96E-12 4.99E-12

RNU6-50P 0.098265905 0.454446756 2.209348429 1.99E-12 5.07E-12

XBP1P1 0.09655031 0.278844265 1.530106798 1.99E-12 5.07E-12

RHOA-IT1 0.121514385 0.425607626 1.808396889 2.02E-12 5.14E-12

MS4A6E 0.010615401 0.058785751 2.469307685 2.03E-12 5.16E-12

AC093525.9 0.019642139 0.065957537 1.747585487 2.07E-12 5.28E-12

SNORD83A 0.189347022 0.870061088 2.200083965 2.08E-12 5.30E-12

MIR8058 0.019963806 0.386407229 4.274663422 2.12E-12 5.40E-12

AC092032.1 0.132134298 0.399114618 1.594798125 2.13E-12 5.42E-12

AL133553.1 0.016983542 0.114152837 2.748757406 2.15E-12 5.46E-12

IFITM3P3 0.085848429 0.313183988 1.867146814 2.16E-12 5.51E-12

AC105219.1 0.01766274 0.14694385 3.056483879 2.16E-12 5.51E-12

AC008060.1 0.00221274 0.27821923 6.974244306 2.17E-12 5.53E-12

AC034207.1 0.021911917 0.07418558 1.759423101 2.17E-12 5.53E-12

KRT223P 0.33876326 9.315739501 4.781320975 2.18E-12 5.54E-12

MIR4668 0.251692397 1.156256614 2.199728074 2.18E-12 5.54E-12

AC097717.1 0.003054331 0.01549817 2.343169439 2.18E-12 5.54E-12

HNRNPA1P76 0.002572221 0.056997602 4.469814494 2.19E-12 5.56E-12

AL513523.4 0.010724944 0.040485304 1.916428249 2.19E-12 5.56E-12

GAD2 0.00148173 0.017649566 3.574278644 2.20E-12 5.59E-12

LRRD1 0.004063903 0.01460253 1.845280525 2.21E-12 5.60E-12

POU5F1P6 0.024231933 0.106153041 2.131164284 2.21E-12 5.62E-12

AL022323.1 0.005768857 0.070459353 3.61043377 2.26E-12 5.73E-12

AC008013.1 0.021716305 0.236615319 3.445692931 2.29E-12 5.81E-12

AC104971.2 0.012227684 0.159394462 3.704378438 2.32E-12 5.88E-12

SALRNA1 0.002304319 0.029016655 3.654468645 2.33E-12 5.91E-12

SHISA8 0.080760649 0.02734693 -1.562273637 2.35E-12 5.95E-12

FOSL1P1 0.016919179 0.17598455 3.378717305 2.36E-12 5.97E-12

AC055876.2 0.02657295 0.127052027 2.25738908 2.39E-12 6.06E-12

ME2P1 0.00273188 0.17531449 6.003907553 2.40E-12 6.07E-12

HLA-T 0.078138046 0.226968832 1.538397116 2.40E-12 6.09E-12

DIAPH1-AS1 0.00428622 0.113763552 4.730188636 2.41E-12 6.10E-12

MIR1285-1 0.279502017 1.018080886 1.864921589 2.48E-12 6.28E-12

GOLGA6L2 0.001147942 0.130741362 6.831521568 2.50E-12 6.33E-12

RNU6-343P 0.303801844 1.064779323 1.809351931 2.53E-12 6.40E-12

AC067852.4 0.019965121 0.142286161 2.83324166 2.54E-12 6.42E-12

LINC02360 0.481237939 0.049941934 -3.268426794 2.54E-12 6.42E-12

AC105384.2 0.01533694 0.174251432 3.506087951 2.55E-12 6.44E-12

AL049840.2 0.645291486 1.845984376 1.516367448 2.55E-12 6.46E-12

AC092718.6 0.028690535 0.180273124 2.651537563 2.61E-12 6.60E-12

LINC02408 0.014863254 0.045815373 1.624081768 2.62E-12 6.63E-12

NALT1 0.153686553 0.641720519 2.061954171 2.63E-12 6.65E-12

AC008080.1 0.019118771 0.09664573 2.337716221 2.64E-12 6.68E-12

AL359885.1 0.014874961 0.080716392 2.439975815 2.66E-12 6.73E-12

AC073283.1 0.028988633 0.108915235 1.909646553 2.67E-12 6.74E-12

AL592494.1 0.009244472 0.111476131 3.59200007 2.68E-12 6.78E-12

AC091059.2 0.00982568 0.179429419 4.190715366 2.69E-12 6.79E-12

IL20RB-AS1 0.002629052 0.136931272 5.702765506 2.70E-12 6.81E-12

AC010719.1 0.158760966 0.634456567 1.998665162 2.70E-12 6.81E-12

AP003497.2 0.002692293 0.040569572 3.913490798 2.71E-12 6.84E-12

MIR548P 0.011306763 0.321272553 4.828539931 2.73E-12 6.88E-12

GPR50 0.024522386 0.004473195 -2.454721906 2.73E-12 6.89E-12

AC007326.2 0.000285129 0.007294074 4.677036768 2.75E-12 6.93E-12

AC004461.2 0.032233376 0.212463774 2.720589694 2.75E-12 6.94E-12

RN7SL368P 0.070209149 0.414291435 2.560915054 2.75E-12 6.94E-12

FAM183DP 0.271071726 0.085412994 -1.666147179 2.77E-12 6.99E-12

PDC 0.018197124 0.073704287 2.018038107 2.78E-12 7.01E-12

FNDC11 0.357196872 0.08752331 -2.028980241 2.80E-12 7.05E-12

RN7SL600P 0.09030702 0.480130983 2.410517986 2.80E-12 7.07E-12

AC124319.3 0.054438752 0.213504581 1.971561134 2.81E-12 7.08E-12

WFDC10B 0.018047441 0.232847369 3.689518393 2.86E-12 7.21E-12

DQX1 0.0120239 0.088714367 2.883262803 2.87E-12 7.24E-12

RPL36AP13 0.015025421 0.113259797 2.914158548 2.90E-12 7.30E-12

LINC02582 0.001021939 0.021978778 4.426729652 2.91E-12 7.32E-12

SNORA22 0.125396237 1.770395218 3.819505502 2.91E-12 7.33E-12

AC020594.1 0.211982826 0.613809802 1.533844297 2.93E-12 7.38E-12

LINC02631 0.008724072 0.049410323 2.50173886 2.96E-12 7.44E-12

SNORA9B 0.102304151 0.418342557 2.031820087 2.96E-12 7.45E-12

SAGE1 0.005196784 0.025223315 2.27906688 3.00E-12 7.56E-12

BLZF2P 0.002877453 0.04648023 4.013753109 3.05E-12 7.66E-12

AC007785.3 0.012061762 0.088360034 2.872953355 3.05E-12 7.67E-12

AP000437.1 0.024905893 0.088234873 1.824861816 3.06E-12 7.69E-12

AC145285.3 0.064640032 0.262411413 2.021330652 3.06E-12 7.70E-12

RPL13AP12 0.081814345 0.283543684 1.793145292 3.09E-12 7.76E-12

LINC01556 0.111325828 0.371889196 1.740084489 3.14E-12 7.89E-12

AL513164.1 0.013057602 0.430836864 5.044179809 3.15E-12 7.90E-12

AP000942.1 0.021906631 0.065884153 1.588563856 3.19E-12 8.00E-12

ITIH1 0.013430357 0.840533927 5.967736519 3.21E-12 8.06E-12

CDC42P3 0.021646399 0.117840849 2.444640789 3.22E-12 8.08E-12

ZDHHC4P1 0.005857368 0.092640238 3.983314586 3.23E-12 8.10E-12

AL121809.2 0.020760675 0.006733532 -1.624417918 3.25E-12 8.16E-12

AP006621.4 0.235820988 1.001369746 2.086210744 3.25E-12 8.16E-12

Z98200.2 0.066444658 0.221594925 1.73769971 3.25E-12 8.16E-12

AC009139.1 0.013447028 0.075872683 2.496293169 3.26E-12 8.18E-12

LINC01479 0.010913703 0.057358624 2.393869676 3.26E-12 8.18E-12

AL133467.3 0.00914107 0.002154937 -2.0847176 3.29E-12 8.26E-12

RN7SKP80 0.170387993 1.158571771 2.765451841 3.31E-12 8.29E-12

AC007610.6 0.019830843 0.080243234 2.016633721 3.42E-12 8.57E-12

AC132192.1 0.047848455 0.21077616 2.139167455 3.42E-12 8.58E-12

AL445490.1 0.031833634 0.11706795 1.878722393 3.44E-12 8.62E-12

LYPLA2P3 0.004781265 0.170555379 5.156704161 3.45E-12 8.63E-12

F2 0.225922261 2.47904949 3.455888738 3.47E-12 8.69E-12

NPTN-IT1 0.198570018 0.607257592 1.612660817 3.49E-12 8.74E-12

GAPDHP64 0.015478338 0.047197679 1.608465374 3.54E-12 8.86E-12

RPL12P32 0.022751041 0.100139736 2.13801008 3.56E-12 8.90E-12

AL445248.1 0.076766094 0.245366598 1.676397725 3.57E-12 8.94E-12

AC006539.2 0.01892102 0.119489031 2.658816413 3.61E-12 9.02E-12

AC068669.1 0.011237917 0.050541435 2.169092014 3.68E-12 9.19E-12

AC091153.2 0.007904302 0.079611987 3.33227565 3.69E-12 9.21E-12

RNU6-1048P 0.287291072 0.902975745 1.652174075 3.70E-12 9.25E-12

LINC02150 0.001693572 0.022745199 3.747420707 3.74E-12 9.33E-12

AC092718.7 0.061414394 0.218360141 1.830060804 3.74E-12 9.33E-12

GPR31 0.014203321 0.070352586 2.308375166 3.82E-12 9.53E-12

AL445070.1 0.115285122 0.030961133 -1.896676189 3.90E-12 9.71E-12

AC139491.1 0.007658718 0.047229422 2.624511007 3.92E-12 9.77E-12

AL590822.1 0.088636204 0.768604924 3.116274215 3.93E-12 9.79E-12

CES3 3.94590223 14.38109304 1.865746218 3.96E-12 9.87E-12

ERVFRD-3 0.01566673 0.054270767 1.792471238 4.03E-12 1.00E-11

KRT85 0.059344002 0.01618788 -1.874188164 4.08E-12 1.02E-11

LINC01819 0.36021234 0.04384247 -3.038446634 4.09E-12 1.02E-11

TLR9 0.00595604 0.017405917 1.547152441 4.12E-12 1.03E-11

CLEC4F 0.122448114 0.366599785 1.582035392 4.19E-12 1.04E-11

AP001178.2 0.101110398 0.493745211 2.287835391 4.20E-12 1.04E-11

MIR3945HG 0.04706995 0.157596105 1.743353637 4.21E-12 1.05E-11

AC007569.1 0.041888011 0.15820166 1.917155464 4.23E-12 1.05E-11

RPL26P35 0.017367597 0.267742917 3.946378379 4.30E-12 1.07E-11

TUBBP5 0.814199362 0.275494317 -1.563359534 4.31E-12 1.07E-11

RFPL3S 0.051009078 0.158045633 1.631515238 4.36E-12 1.08E-11

SORCS1 0.578003729 0.131181191 -2.13951792 4.37E-12 1.09E-11

PRSS57 0.035289016 0.16752652 2.247098389 4.43E-12 1.10E-11

LINC01497 0.17492708 0.031745777 -2.462117035 4.43E-12 1.10E-11

AC135178.2 0.070889522 0.296774186 2.065721312 4.47E-12 1.11E-11

AC002044.1 0.077712297 0.555233319 2.836879338 4.49E-12 1.11E-11

AC091946.1 0.013622702 0.154949144 3.507709988 4.50E-12 1.12E-11

AC068790.4 0.142136757 0.464043894 1.706981591 4.53E-12 1.12E-11

AC027279.1 0.038991233 0.189864467 2.283748266 4.55E-12 1.13E-11

HMGB3P7 0.006318116 0.179965321 4.832080669 4.57E-12 1.13E-11

ADCYAP1R1 0.024690917 0.087031869 1.817563349 4.60E-12 1.14E-11

AC079203.1 0.124592013 0.429984928 1.787074497 4.62E-12 1.15E-11

AC020908.3 0.006664989 0.069335268 3.37891492 4.71E-12 1.17E-11

RN7SKP30 0.02816647 0.143040234 2.344370339 4.80E-12 1.19E-11

AC023202.1 0.014797414 0.081315788 2.458190415 4.84E-12 1.20E-11

LINC02225 0.001701929 0.016191282 3.249974453 4.88E-12 1.21E-11

RPS15AP6 0.026806154 0.229122486 3.095482888 4.91E-12 1.22E-11

KIAA0895LP1 0.006553727 0.05971545 3.187716736 4.94E-12 1.22E-11

MIR4257 0.038086649 0.396021823 3.378222681 5.07E-12 1.25E-11

LINC01280 0.001302353 0.035590591 4.772303825 5.07E-12 1.26E-11

GSDMC 0.027824443 0.12298797 2.144092498 5.10E-12 1.26E-11

AL034417.1 0.175876762 0.683296985 1.95794788 5.23E-12 1.29E-11

AP003733.1 0.009993158 0.045012795 2.171322538 5.27E-12 1.30E-11

AC124854.1 1.089440548 5.830667031 2.420073469 5.35E-12 1.32E-11

AC015799.1 0.031281529 0.191073552 2.610745143 5.35E-12 1.32E-11

PHBP13 0.019005364 0.075849136 1.996726066 5.43E-12 1.34E-11

CARMN 0.107672238 0.409489687 1.9271808 5.49E-12 1.35E-11

UCKL1-AS1 0.062513789 0.205793769 1.71895294 5.51E-12 1.36E-11

AC011815.2 0.058864055 0.187195981 1.66909064 5.54E-12 1.37E-11

LINC00434 0.03329354 0.011342497 -1.55350395 5.58E-12 1.38E-11

ADAM12 0.474845348 1.693797274 1.834731586 5.60E-12 1.38E-11

AC011447.6 0.018835863 0.058575176 1.636807222 5.69E-12 1.40E-11

C18orf63 0.008419445 0.034362691 2.029045901 5.73E-12 1.41E-11

PAH 37.24339022 5.445535652 -2.773838534 5.88E-12 1.45E-11

LINC01522 0.056872193 0.311726293 2.454484503 5.99E-12 1.47E-11

Z97985.1 0.028743918 0.089082583 1.631886659 6.01E-12 1.48E-11

LIPN 0.03640515 0.110910758 1.607184865 6.16E-12 1.51E-11

TMEM246-AS1 3.836072087 1.068066228 -1.844628723 6.19E-12 1.52E-11

OR7E2P 0.016074124 0.080537264 2.324916383 6.19E-12 1.52E-11

AL136988.2 0.050003193 0.168089943 1.74914129 6.20E-12 1.52E-11

DNAJB5-DT 0.079374046 0.278804003 1.812512018 6.24E-12 1.53E-11

ATP5MC2P3 0.012246831 0.107905085 3.139282517 6.32E-12 1.55E-11

AC011479.4 0.019789238 0.069562671 1.813597237 6.33E-12 1.55E-11

SNORA59B 0.030660602 0.213643544 2.800747816 6.37E-12 1.56E-11

RNU6-1053P 0.406908222 1.325296027 1.703539308 6.40E-12 1.57E-11

AC002128.1 0.20441707 0.666121531 1.704269738 6.57E-12 1.61E-11

FMO7P 0.001637706 0.036571934 4.480989005 6.62E-12 1.62E-11

AC004801.2 0.121958301 0.356325065 1.546806014 6.66E-12 1.63E-11

RPL21P10 0.088549415 0.265379354 1.583501459 6.69E-12 1.64E-11

AC037487.1 0.028058596 0.099403526 1.824854216 6.69E-12 1.64E-11

AC005072.1 0.009003654 0.088376052 3.295072886 6.71E-12 1.64E-11

AL355916.2 0.144584171 0.424047121 1.552314974 6.72E-12 1.65E-11

AL359510.2 0.083097031 0.241301546 1.53796833 6.73E-12 1.65E-11

HSPA8P19 0.009149156 0.081526556 3.155559543 6.80E-12 1.66E-11

AC093484.2 0.002736752 0.018619301 2.766262398 6.84E-12 1.68E-11

AL590762.4 0.015174671 0.188737381 3.63664306 6.91E-12 1.69E-11

RPS2P1 0.009128688 0.043595212 2.25569024 7.00E-12 1.71E-11

LINCMD1 0.122562657 0.538764447 2.136135178 7.07E-12 1.73E-11

PHYHIP 1.118194497 0.32076167 -1.80159749 7.08E-12 1.73E-11

AL139022.1 0.057926303 0.192437474 1.732099278 7.10E-12 1.74E-11

RPL7P18 0.019244218 0.111457327 2.533994499 7.11E-12 1.74E-11

AC011676.1 0.786472168 0.256174366 -1.618269591 7.19E-12 1.76E-11

AL358473.1 0.007661558 0.036307275 2.244549013 7.22E-12 1.77E-11

TRIM40 0.038875413 0.011674521 -1.73549459 7.35E-12 1.80E-11

HNRNPA1P70 0.013001057 0.080102848 2.623224666 7.36E-12 1.80E-11

BNIP3P23 0.007177841 0.076367395 3.411334981 7.37E-12 1.80E-11

SMARCE1P6 0.01744046 0.078964578 2.178767499 7.43E-12 1.82E-11

NLRP7 0.012358559 0.057764359 2.224669114 7.58E-12 1.85E-11

TMPRSS11A 0.001032963 0.094771889 6.519598492 7.58E-12 1.85E-11

AP002518.2 0.03086171 0.2307015 2.902137473 7.59E-12 1.85E-11

AC020917.2 0.009040713 0.119144992 3.720137977 7.61E-12 1.86E-11

AC026746.1 0.0007254 0.011834842 4.028120006 7.64E-12 1.87E-11

AC004522.3 0.12333468 0.030699651 -2.006284375 7.66E-12 1.87E-11

AC007792.1 0.031434769 0.207364071 2.721732859 7.68E-12 1.87E-11

AC134407.1 0.029998775 0.147070263 2.293530078 7.69E-12 1.88E-11

PM20D1 1.996239577 0.299158411 -2.738303342 7.74E-12 1.89E-11

LINC01888 0.004983426 0.03332747 2.741501985 7.74E-12 1.89E-11

AC090559.2 0.006758714 0.199020188 4.880022259 7.74E-12 1.89E-11

AP001178.1 0.078271207 0.316856328 2.017275227 7.84E-12 1.91E-11

Z84723.1 0.013378021 0.054864645 2.03601207 7.88E-12 1.92E-11

AP005131.3 0.020748028 0.18013625 3.118042396 8.06E-12 1.96E-11

CEACAM20 0.003990586 0.0407535 3.352251311 8.13E-12 1.98E-11

AC116348.5 0.008134439 0.07343299 3.174313633 8.22E-12 2.00E-11

HOXA13 0.07170623 0.386841028 2.431570428 8.23E-12 2.00E-11

OR2B6 0.014551921 0.062690589 2.107039303 8.38E-12 2.04E-11

AC114730.2 0.014317638 0.070466743 2.299148981 8.46E-12 2.06E-11

AP003419.3 0.174521697 0.562227973 1.687748827 8.50E-12 2.07E-11

AL513331.1 0.02012318 0.104573243 2.377583541 8.53E-12 2.08E-11

AC064801.2 0.035514079 0.141403139 1.993351174 8.56E-12 2.08E-11

AL139421.1 0.145582998 0.412085328 1.501101215 8.77E-12 2.13E-11

TGM3 0.025611671 0.09032968 1.818398707 8.82E-12 2.14E-11

MTND4P23 0.01556574 0.082780901 2.410923823 8.90E-12 2.16E-11

AC015849.4 0.035562294 0.167983092 2.239895741 8.97E-12 2.18E-11

AL049795.1 0.071238327 0.214195614 1.5882034 9.01E-12 2.19E-11

AC004678.2 0.025851371 0.120812523 2.224457289 9.01E-12 2.19E-11

AC006487.1 0.007697523 0.056228178 2.868827199 9.09E-12 2.21E-11

RNU1-103P 0.184997814 0.697977604 1.915672524 9.12E-12 2.21E-11

STH 0.009111962 0.108839361 3.578294818 9.21E-12 2.24E-11

AC020558.2 0.191130927 0.566317682 1.567050336 9.48E-12 2.30E-11

AC092755.2 0.056172288 0.175628111 1.64459331 9.48E-12 2.30E-11

ITIH4 0.033955998 0.181245603 2.416207671 9.48E-12 2.30E-11

RNU6-1223P 0.105880317 0.546848944 2.368707949 9.59E-12 2.32E-11

AL022067.1 0.094362041 0.32477799 1.783175331 9.67E-12 2.34E-11

AC099811.1 0.031934111 0.138147545 2.113039745 9.76E-12 2.36E-11

RN7SL812P 0.051429696 0.201969256 1.973462167 9.78E-12 2.37E-11

AL135785.1 0.771334134 0.082604633 -3.22306135 9.97E-12 2.42E-11

MOGAT3 0.397957991 1.956139507 2.297321212 1.01E-11 2.45E-11

LINC00525 0.034055102 0.13556676 1.993060615 1.01E-11 2.45E-11

AC022973.5 0.086001746 0.244464802 1.507188901 1.02E-11 2.46E-11

CCDC60 0.016942507 0.068875284 2.023338949 1.02E-11 2.47E-11

SLX1B 0.003548149 0.019466595 2.455861828 1.02E-11 2.47E-11

AP001628.1 0.081387428 0.347057296 2.092295996 1.03E-11 2.48E-11

AC007546.1 0.176552719 0.591982441 1.745455342 1.03E-11 2.49E-11

LINC00106 0.595310683 2.735853403 2.200276237 1.03E-11 2.49E-11

AL032819.2 0.034400361 0.117353953 1.770370813 1.03E-11 2.50E-11

AC022898.1 0.023185508 0.182618125 2.977534752 1.04E-11 2.50E-11

RPS14P4 0.013725586 0.138784142 3.337903121 1.04E-11 2.52E-11

AL161747.1 0.072011156 0.301232841 2.064586738 1.05E-11 2.53E-11

AL121852.1 0.030914291 0.128934797 2.060295873 1.05E-11 2.53E-11

AC004797.1 0.006610058 0.046028352 2.799787864 1.05E-11 2.53E-11

RNU7-45P 0.457226825 2.16802047 2.245396426 1.05E-11 2.55E-11

AC048341.2 0.78654094 2.522626732 1.681332983 1.06E-11 2.56E-11

TUBA3C 0.019108987 0.166373828 3.122105329 1.08E-11 2.60E-11

GOLGA6L10 0.036392845 0.105655641 1.537643068 1.08E-11 2.61E-11

AL133255.1 0.0366059 0.199802248 2.448424696 1.10E-11 2.66E-11

AP001767.2 0.055228479 0.163397585 1.564902349 1.11E-11 2.67E-11

LINC00272 0.002347176 0.035064305 3.901005135 1.12E-11 2.71E-11

AC129510.1 0.363975128 1.039035257 1.513332836 1.12E-11 2.71E-11

AP001107.3 0.019112432 0.096130967 2.330489859 1.12E-11 2.71E-11

AL645940.1 0.101613925 0.317535696 1.643820669 1.13E-11 2.71E-11

AC099811.6 0.011057981 0.069696415 2.655996518 1.13E-11 2.73E-11

AP001972.4 0.011158012 0.14318799 3.681758584 1.13E-11 2.73E-11

GABBR2 0.030158805 0.108323522 1.84469539 1.14E-11 2.74E-11

HTR5BP 0.017649327 0.054231008 1.619504835 1.14E-11 2.76E-11

AC010536.2 0.082191889 0.518917916 2.658438417 1.15E-11 2.77E-11

RNU6-611P 0.175350911 1.024266305 2.546274027 1.15E-11 2.78E-11

DAO 12.55426448 1.382285341 -3.183050145 1.16E-11 2.80E-11

MIR4635 0.494761926 2.55712854 2.369718293 1.17E-11 2.81E-11

AC107223.1 0.02431792 0.097521647 2.003702684 1.18E-11 2.84E-11

LINC02730 0.06805586 0.009188482 -2.888820915 1.18E-11 2.84E-11

RNU6-1136P 0.183656647 0.560314547 1.609225833 1.18E-11 2.85E-11

AC102945.1 0.01717597 0.107729942 2.648955827 1.20E-11 2.90E-11

AC103783.1 0.020765869 0.377895483 4.185701092 1.21E-11 2.90E-11

AC107219.1 0.218186695 0.746954273 1.775456798 1.21E-11 2.91E-11

AC023302.1 0.314414989 0.098812052 -1.66991108 1.21E-11 2.92E-11

LINC00824 0.002562749 0.028003035 3.449819215 1.24E-11 2.98E-11

AL590006.1 0.026573512 0.150255321 2.499355259 1.24E-11 2.99E-11

CARS1-AS1 0.005326772 0.029309463 2.460033044 1.26E-11 3.02E-11

SNORD15B 0.174419887 6.66530525 5.256034503 1.26E-11 3.03E-11

WDR49 0.101640238 0.027465243 -1.887792727 1.27E-11 3.06E-11

OR13C5 0.016410997 0.00540705 -1.601749278 1.28E-11 3.08E-11

MRPS18AP1 0.047867054 0.210846977 2.139091408 1.29E-11 3.10E-11

AL157762.1 0.049884847 0.168955222 1.759967393 1.30E-11 3.12E-11

TRAJ18 0.023256135 0.364358804 3.96967661 1.30E-11 3.12E-11

RPS20P2 0.035263435 0.178639218 2.340803935 1.30E-11 3.13E-11

AC087359.1 0.005514324 0.050159006 3.185252831 1.32E-11 3.18E-11

AL354813.1 0.012600671 0.050515882 2.003236498 1.32E-11 3.18E-11

RN7SKP78 0.016607767 0.206064501 3.633165944 1.34E-11 3.21E-11

AC068993.1 0.03185839 0.010222516 -1.63992304 1.34E-11 3.22E-11

AP001885.1 0.011565666 0.098466243 3.089780826 1.34E-11 3.22E-11

DOCK4-AS1 0.005476319 0.096791308 4.143599141 1.36E-11 3.25E-11

SLC26A4 3.11408057 0.318452459 -3.289656354 1.36E-11 3.25E-11

AL928654.2 0.738447469 2.173388958 1.557379186 1.36E-11 3.25E-11

LINC02145 0.053089929 0.180561028 1.765976413 1.36E-11 3.25E-11

LINC01714 0.190397275 0.573805656 1.591549355 1.37E-11 3.28E-11

MIR583HG 0.045813558 0.161184032 1.814862304 1.38E-11 3.31E-11

AC012615.4 0.025754019 0.13165876 2.353934021 1.40E-11 3.35E-11

IL37 0.003567921 0.02995099 3.069448053 1.42E-11 3.41E-11

AC087392.3 0.049366556 0.144045156 1.544915245 1.43E-11 3.43E-11

AC132872.2 0.044852527 0.1519352 1.76019497 1.43E-11 3.43E-11

FAT2 0.392867809 0.11089701 -1.824823487 1.43E-11 3.43E-11

AC244100.1 0.006058268 0.044182049 2.866483054 1.44E-11 3.44E-11

IFIT6P 0.003743845 0.037834081 3.33709381 1.49E-11 3.56E-11

AC135050.4 0.036194868 0.143588435 1.988082475 1.51E-11 3.62E-11

RPL23AP55 0.021659871 0.114282062 2.399502405 1.52E-11 3.62E-11

ZBTB20-AS1 0.029177555 0.170341555 2.545499515 1.53E-11 3.64E-11

AC104984.6 0.010870625 0.219572635 4.336191509 1.54E-11 3.67E-11

AL132642.1 0.030852481 0.148305133 2.26511014 1.56E-11 3.71E-11

AL138789.1 0.003969652 0.021922042 2.465297916 1.57E-11 3.74E-11

AC117503.2 0.117871586 0.358500462 1.604758988 1.58E-11 3.77E-11

AL513008.1 0.042307088 0.424146007 3.32558969 1.59E-11 3.78E-11

AC010422.2 0.079124815 0.265622299 1.747174149 1.59E-11 3.79E-11

AL731563.3 0.018195037 0.165426497 3.184573463 1.59E-11 3.80E-11

RNF216-IT1 0.026168355 0.184589648 2.81842652 1.61E-11 3.84E-11

AC013460.1 0.059966902 0.013348958 -2.167439353 1.63E-11 3.88E-11

SUMO1P1 0.008409733 0.076771957 3.190447443 1.64E-11 3.90E-11

AC004448.2 0.003495672 0.024718037 2.821922581 1.64E-11 3.91E-11

AC005632.2 0.005108227 0.067318894 3.720116985 1.65E-11 3.92E-11

AC109597.2 0.015805861 0.123316059 2.963829199 1.68E-11 3.99E-11

AC087501.1 0.029670763 0.160380704 2.434386652 1.68E-11 4.00E-11

PLGLB2 0.009149473 0.032135617 1.812412596 1.69E-11 4.03E-11

RNU4ATAC12P 0.018827601 0.380893536 4.338466685 1.73E-11 4.10E-11

PTPRR 0.13341116 0.400996282 1.587709511 1.73E-11 4.10E-11

CHKB-CPT1B 0.072762262 0.246321006 1.759277361 1.73E-11 4.12E-11

TBL1XR1-AS1 0.019774402 0.144072087 2.865084855 1.75E-11 4.16E-11

AL031777.1 0.107208707 0.403054286 1.910552081 1.78E-11 4.22E-11

HLA-DRB9 0.309947769 0.898673851 1.535772503 1.80E-11 4.28E-11

AL449283.1 0.004106122 0.035390383 3.107509074 1.81E-11 4.29E-11

AC125618.2 0.236736637 0.077110986 -1.618274669 1.81E-11 4.30E-11

LINC01864 0.003493089 0.019626772 2.490247787 1.83E-11 4.34E-11

SLC7A11-AS1 0.001562412 0.010095914 2.69192436 1.84E-11 4.37E-11

CADM3 0.819236038 4.931648471 2.589718882 1.85E-11 4.38E-11

ZSWIM8-AS1 0.01432354 0.053470884 1.900365448 1.87E-11 4.43E-11

AC112496.1 0.192407958 0.58044059 1.592979942 1.88E-11 4.47E-11

LINC02257 0.006929023 0.116864148 4.076036699 1.90E-11 4.50E-11

PI4KAP1 0.359875067 1.054419324 1.550880659 1.90E-11 4.51E-11

RNU6-623P 0.076443899 0.35260354 2.205573679 1.91E-11 4.53E-11

AC008957.2 0.006373121 0.202270723 4.988143631 1.92E-11 4.54E-11

RN7SL23P 0.080234088 0.325616296 2.020885689 1.92E-11 4.56E-11

TPT1P10 0.034792544 0.109746306 1.657322317 1.92E-11 4.56E-11

MAGEA10 0.013121296 0.095837369 2.868678072 1.93E-11 4.57E-11

AC007906.1 0.027929372 0.156163613 2.483203295 1.94E-11 4.59E-11

AC008739.2 0.027922174 0.138540302 2.310822562 1.96E-11 4.64E-11

AP005436.3 0.034994393 0.164974992 2.237051659 1.97E-11 4.67E-11

P4HA3-AS1 0.00150144 0.011258931 2.906651096 1.98E-11 4.69E-11

HMGN2P8 0.015887412 0.212354397 3.740517907 1.98E-11 4.70E-11

TRABD2A 0.122796511 0.399607113 1.702312697 1.99E-11 4.70E-11

LINC02294 8.955562275 2.121126305 -2.077953484 2.01E-11 4.75E-11

AC002519.1 0.012148702 0.046561512 1.938335693 2.02E-11 4.77E-11

TRAV34 0.019049622 0.13850619 2.862116186 2.06E-11 4.86E-11

NPM1P50 0.010293803 0.060970614 2.566337959 2.07E-11 4.90E-11

AC012668.3 0.001095368 0.018862263 4.106014735 2.07E-11 4.90E-11

AC133473.1 0.033885074 0.1872367 2.466141428 2.12E-11 5.01E-11

AC018804.1 0.118054274 0.476517166 2.013077917 2.14E-11 5.05E-11

AL008707.1 0.123474995 0.415506353 1.750651621 2.14E-11 5.05E-11

AL683807.2 0.00427894 0.050090671 3.549216642 2.15E-11 5.07E-11

AL591623.1 0.005567697 0.031722592 2.510357961 2.16E-11 5.09E-11

MYO1H 0.047924626 0.158335127 1.724142271 2.16E-11 5.10E-11

DNAH8-AS1 0.003126417 0.091101934 4.864899652 2.19E-11 5.16E-11

Z93241.1 0.051342081 0.281070073 2.452716186 2.29E-11 5.41E-11

AL583832.1 0.0779928 0.261079871 1.743078383 2.30E-11 5.42E-11

MARK2P8 0.012269911 0.140499445 3.517367698 2.32E-11 5.47E-11

AC100793.3 0.034902394 0.144382065 2.048493639 2.35E-11 5.53E-11

AC012645.2 0.019684197 0.092326464 2.229706402 2.36E-11 5.56E-11

AC011481.1 0.20753463 0.776333208 1.903323909 2.38E-11 5.60E-11

AC019226.1 0.137448829 0.424590731 1.627178263 2.39E-11 5.63E-11

AC245884.4 0.013360904 0.059838847 2.163064697 2.40E-11 5.65E-11

MTATP6P26 0.019016689 0.084017886 2.143430408 2.41E-11 5.68E-11

KRT73 0.003891893 0.02012016 2.37009789 2.42E-11 5.68E-11

AC126118.1 0.105057362 0.341438131 1.700446919 2.42E-11 5.70E-11

DDX39BP1 0.061970447 0.277506452 2.162869031 2.44E-11 5.74E-11

AOAH-IT1 0.013542833 0.101506933 2.905976826 2.44E-11 5.74E-11

YBX1P4 0.030935203 0.131073503 2.083054657 2.46E-11 5.77E-11

RNU6-10P 0.036993042 0.311285262 3.072911412 2.46E-11 5.79E-11

SNORA33 1.123508455 3.743426416 1.736348415 2.50E-11 5.87E-11

NBPF13P 0.003884948 0.027349758 2.815560867 2.51E-11 5.90E-11

AC004486.1 0.156162079 0.572229531 1.87354979 2.51E-11 5.90E-11

BAAT 0.092684061 4.417637677 5.574810024 2.55E-11 5.98E-11

AC073325.2 0.300996484 1.025238014 1.768140337 2.58E-11 6.06E-11

AC004551.1 0.004296986 0.077565338 4.174015034 2.61E-11 6.12E-11

AC091180.6 0.044587252 0.195152643 2.129899828 2.67E-11 6.27E-11

PYCR1 1.066275435 3.393914528 1.670370077 2.67E-11 6.27E-11

AL354989.1 0.067013994 0.218914904 1.707835875 2.68E-11 6.29E-11

AC012462.1 0.00472851 0.044105966 3.221516136 2.69E-11 6.30E-11

AC005674.2 0.268052131 0.76060599 1.504635696 2.70E-11 6.33E-11

AL451164.2 0.033968518 0.118541854 1.803126339 2.71E-11 6.34E-11

AC243960.7 0.008982707 0.056689351 2.657855636 2.74E-11 6.42E-11

AC023509.5 0.005969571 0.126847993 4.409329731 2.74E-11 6.43E-11

AL133373.2 0.005814947 0.036384032 2.645467529 2.75E-11 6.44E-11

AC099677.1 1.318743608 4.291603918 1.702352831 2.78E-11 6.51E-11

SPDYE19P 0.032759438 0.120548495 1.879631102 2.78E-11 6.51E-11

Z98885.2 0.0138229 0.056819528 2.039326524 2.79E-11 6.53E-11

SRRM1P3 0.009467911 0.042274805 2.158680075 2.84E-11 6.65E-11

PDE6A 0.008146666 0.033157382 2.025048396 2.87E-11 6.72E-11

AC003984.1 0.236368805 1.190645968 2.332632946 2.89E-11 6.75E-11

GAPDHP47 0.00496671 0.034191203 2.783262756 2.90E-11 6.79E-11

RNU6-762P 0.234840984 0.748848148 1.672988991 2.91E-11 6.80E-11

AC007224.2 0.078824584 0.297728144 1.917278057 2.91E-11 6.81E-11

OR56B1 0.001872273 0.028260318 3.915915274 2.94E-11 6.88E-11

AL035404.2 0.034081686 0.261890205 2.941893497 2.94E-11 6.88E-11

AC073288.2 0.029003068 0.098494257 1.763834116 2.95E-11 6.90E-11

DES 21.21334589 7.238267128 -1.551255933 2.96E-11 6.92E-11

AC005262.1 0.024696438 0.26332904 3.414491723 2.96E-11 6.93E-11

XKR3 0.013526728 0.056707339 2.067722527 2.99E-11 6.99E-11

AL645568.2 0.003608842 0.036411929 3.334803442 3.00E-11 7.01E-11

CLEC18B 2.434091234 8.341671672 1.776953285 3.02E-11 7.05E-11

CYP4F3 5.655357784 1.225016479 -2.206817142 3.06E-11 7.15E-11

CORO6 0.093570706 0.407864287 2.123960347 3.06E-11 7.15E-11

GAPDHP32 0.059411454 0.188647224 1.666877857 3.07E-11 7.17E-11

MIR3164 0.141361582 0.673699875 2.252715942 3.14E-11 7.34E-11

DNM1P33 0.014926706 0.102765659 2.783390485 3.15E-11 7.36E-11

GABRR1 0.027657904 0.09209648 1.735454178 3.16E-11 7.37E-11

Z96811.1 0.072274722 0.299639462 2.051664571 3.20E-11 7.47E-11

AC074029.3 0.117005294 0.340218397 1.539887355 3.21E-11 7.49E-11

ADGRG3 0.114641721 0.371275757 1.695358945 3.21E-11 7.49E-11

AC090286.3 0.028328303 0.111028997 1.97062043 3.27E-11 7.64E-11

ZDHHC22 0.003723229 0.032772113 3.137842566 3.32E-11 7.74E-11

AL117209.1 0.194589444 0.608899265 1.64577012 3.34E-11 7.78E-11

AC125611.1 0.066061357 0.476373837 2.85021568 3.37E-11 7.85E-11

CYP3A43 0.018771882 0.072600794 1.951412031 3.39E-11 7.90E-11

MROH3P 0.01831022 0.09084542 2.310764669 3.48E-11 8.10E-11

STX19 0.378408502 0.123942603 -1.610272326 3.49E-11 8.13E-11

MTAPP2 0.046660908 0.266429886 2.513469627 3.51E-11 8.17E-11

SNORA58B 0.026421589 0.192802428 2.867334079 3.53E-11 8.21E-11

LINC02469 0.022016026 0.083451381 1.922381863 3.62E-11 8.42E-11

AC015818.5 0.006902434 0.05772667 3.064060844 3.65E-11 8.48E-11

AC067817.2 0.250253489 0.993010851 1.988419302 3.68E-11 8.54E-11

ZSCAN4 0.026704827 0.088885433 1.734846459 3.68E-11 8.55E-11

AL008718.3 0.052979638 0.193170091 1.866361843 3.68E-11 8.56E-11

AC092045.1 0.03222486 0.094600056 1.553666958 3.70E-11 8.59E-11

SLC6A7 0.005235733 0.021410828 2.031877154 3.70E-11 8.60E-11

AC069360.1 0.021415147 0.092654666 2.113232079 3.70E-11 8.60E-11

RNY1P13 0.133820204 0.605588657 2.178042236 3.72E-11 8.63E-11

AC023090.2 0.015565061 0.065994782 2.084040745 3.74E-11 8.67E-11

AL590068.1 0.01496141 0.056503515 1.917094509 3.76E-11 8.72E-11

RNA5SP449 0.049439244 0.238119728 2.267958553 3.78E-11 8.77E-11

CPNE7 0.244287468 1.211761063 2.310453496 3.78E-11 8.78E-11

AC092171.4 0.198323965 0.710168814 1.84030299 3.82E-11 8.86E-11

AC104758.3 0.006327224 0.081376368 3.684965246 3.83E-11 8.87E-11

RNU6-917P 0.017632569 0.20589414 3.545588145 3.95E-11 9.16E-11

SLC45A2 0.05818203 0.260007386 2.159907077 3.97E-11 9.20E-11

GRAMD4P8 0.007288081 0.035790661 2.295972323 4.00E-11 9.26E-11

CCK 0.05200914 0.014491046 -1.843603489 4.06E-11 9.40E-11

ODF2-AS1 0.060157594 0.223793034 1.895346354 4.07E-11 9.42E-11

RN7SL187P 0.006129237 0.099292447 4.017904568 4.08E-11 9.43E-11

AC091959.1 0.028744511 0.25847031 3.168640195 4.12E-11 9.53E-11

AC011498.3 0.003881296 0.051695618 3.735431478 4.13E-11 9.56E-11

AC111170.2 0.011444694 0.077132 2.752650583 4.17E-11 9.65E-11

AC072022.1 0.064347276 0.21162553 1.717562694 4.19E-11 9.69E-11

MGAT3-AS1 0.112894884 0.340259204 1.59165408 4.19E-11 9.69E-11

SNORA68B 0.071197547 0.35232786 2.307019126 4.23E-11 9.77E-11

TRBV11-3 0.02404728 0.147508126 2.616848824 4.24E-11 9.81E-11

AC093525.10 0.015438989 0.050137392 1.699308689 4.26E-11 9.83E-11

AC099681.2 0.00627282 0.055990469 3.157995263 4.26E-11 9.84E-11

NIM1K 0.164269292 0.476549306 1.536562677 4.31E-11 9.94E-11

AACSP1 0.20234816 0.672706112 1.733136633 4.33E-11 9.99E-11

AC245884.9 0.020239368 0.119144239 2.557473015 4.35E-11 1.00E-10

ETV3L 0.003187396 0.027868319 3.128175896 4.36E-11 1.01E-10

PPIAP30 0.026725622 0.141926497 2.408848559 4.48E-11 1.03E-10

SEC31B 0.327714919 1.075291458 1.714214498 4.50E-11 1.04E-10

LINC01095 0.009254911 0.003093486 -1.580985753 4.56E-11 1.05E-10

UBA52P8 0.386495455 1.13251334 1.551004691 4.62E-11 1.07E-10

AC016027.5 0.061779282 0.303070683 2.294459302 4.64E-11 1.07E-10

ENPP2 23.147985 70.05449092 1.597590925 4.69E-11 1.08E-10

AC233992.2 0.00882823 0.047690458 2.433504522 4.71E-11 1.09E-10

AP003469.3 0.014248224 0.080666161 2.50118147 4.72E-11 1.09E-10

AL033519.2 0.015430386 0.287601568 4.220225486 4.86E-11 1.12E-10

AC005921.4 0.013112302 0.09624809 2.875836935 4.91E-11 1.13E-10

AC120057.4 0.155292965 0.656382502 2.079544301 4.95E-11 1.14E-10

PKD1L3 0.016976158 0.05210457 1.617899893 4.97E-11 1.14E-10

RNU6-564P 0.051815713 0.293268255 2.500759361 4.97E-11 1.14E-10

TMEM174 42.01655936 9.471136813 -2.149348521 4.99E-11 1.15E-10

AC008915.2 0.016227188 0.094594735 2.543346873 5.00E-11 1.15E-10

TRBV10-1 0.013784206 0.141419562 3.358893643 5.01E-11 1.15E-10

AL591767.3 0.006793177 0.064098385 3.238129608 5.06E-11 1.16E-10

AC104046.1 0.074177068 0.250893757 1.758031418 5.06E-11 1.16E-10

AC087392.4 0.0496654 0.148209022 1.577320233 5.06E-11 1.16E-10

AC098935.2 0.020282938 0.104083076 2.359396964 5.09E-11 1.17E-10

LINC01771 0.025506059 0.09749009 1.934415576 5.09E-11 1.17E-10

AC022898.2 0.037461245 0.154216848 2.041489624 5.12E-11 1.18E-10

AC090286.4 0.526459611 2.305750411 2.130841596 5.14E-11 1.18E-10

AL157823.2 0.037410155 0.181089545 2.275201414 5.14E-11 1.18E-10

AC011825.2 0.128156525 0.412288736 1.685748109 5.16E-11 1.18E-10

AC092295.1 0.010889595 0.068659518 2.656509478 5.19E-11 1.19E-10

LINC02137 0.005862718 0.024536114 2.065265245 5.24E-11 1.20E-10

RNA5SP228 0.290676254 0.085758715 -1.761058033 5.27E-11 1.21E-10

ACOX2 11.18087994 3.173125389 -1.817057296 5.29E-11 1.21E-10

AC016405.1 0.047758963 0.28203702 2.562041116 5.29E-11 1.21E-10

AC098679.3 0.024960351 0.088342389 1.823467633 5.29E-11 1.21E-10

AL139002.1 0.060616112 0.009289859 -2.705972657 5.32E-11 1.22E-10

CLEC17A 0.080542217 0.234000124 1.538692216 5.32E-11 1.22E-10

PHBP19 0.046268961 0.196551822 2.08679312 5.36E-11 1.23E-10

FOXL2 0.006249549 0.024188356 1.952488672 5.39E-11 1.24E-10

GZMAP1 0.01306284 0.069023446 2.40161787 5.44E-11 1.25E-10

AC023510.1 0.033368058 0.153712866 2.203698279 5.48E-11 1.26E-10

AC020900.1 0.029425313 0.156994412 2.415583551 5.51E-11 1.26E-10

VPS9D1-AS1 0.229626455 0.75562897 1.718389153 5.67E-11 1.30E-10

LINC01013 0.025129614 0.078525272 1.643768502 5.83E-11 1.33E-10

LSINCT5 0.013230245 0.004593034 -1.52632041 5.83E-11 1.34E-10

OCM 0.039546474 0.124133906 1.650276235 5.85E-11 1.34E-10

RDM1P3 0.071581151 0.245077208 1.775584666 5.94E-11 1.36E-10

AC026310.3 0.008502706 0.035370838 2.056566468 5.95E-11 1.36E-10

RNU6-90P 0.062784584 0.347213339 2.467340091 5.96E-11 1.36E-10

AL022334.2 0.012060913 0.088259197 2.871407534 5.99E-11 1.37E-10

LINC02744 0.004108776 0.024169586 2.556412187 6.03E-11 1.38E-10

AC099681.3 0.004010923 0.028967034 2.852405788 6.13E-11 1.40E-10

AC005086.2 0.012505249 0.083490422 2.739076868 6.20E-11 1.42E-10

BLACAT1 0.375469682 0.130431367 -1.525405554 6.23E-11 1.42E-10

MRPL53P1 0.041032192 0.216367705 2.398657034 6.26E-11 1.43E-10

MIR6859-1 0.086435225 0.523918746 2.599651803 6.30E-11 1.44E-10

RPH3A 0.015018771 0.045952196 1.613367011 6.31E-11 1.44E-10

CYP2C8 0.410565796 1.538445777 1.90578825 6.34E-11 1.45E-10

AC016894.1 0.035570624 0.153938473 2.113595644 6.40E-11 1.46E-10

AC103810.1 0.090473723 0.281575877 1.637953007 6.52E-11 1.49E-10

SYTL1 0.372823383 1.091240262 1.549404527 6.52E-11 1.49E-10

AC096708.3 0.036190971 0.216579992 2.581198236 6.52E-11 1.49E-10

ACTN3 0.011524269 0.036654616 1.669319656 6.53E-11 1.49E-10

LINC02037 0.012412587 0.049588312 1.998196234 6.56E-11 1.49E-10

AC011476.2 0.021496349 0.082757408 1.944796788 6.57E-11 1.50E-10

UPK1A 1.058490106 0.167192147 -2.662428789 6.60E-11 1.50E-10

AC093627.2 0.138984905 0.026542862 -2.388532362 6.74E-11 1.53E-10

AC090772.4 0.029529738 0.181778659 2.621942353 6.75E-11 1.54E-10

RN7SL602P 0.008099397 0.134704683 4.05584175 6.81E-11 1.55E-10

AC006254.1 0.054170226 0.188054057 1.795575414 6.87E-11 1.56E-10

SNORD51 0.066855486 0.414425902 2.63199632 6.87E-11 1.56E-10

AL391261.2 0.36509237 0.115325447 -1.662550635 6.88E-11 1.56E-10

PPEF2 0.004791723 0.023346288 2.284576937 6.98E-11 1.59E-10

AC138430.1 0.00223871 0.012371968 2.466335615 6.99E-11 1.59E-10

AKR7A3 27.92207006 9.749449963 -1.51801317 7.07E-11 1.61E-10

AL158196.1 0.078930766 0.236104233 1.580764245 7.08E-11 1.61E-10

JAKMIP2-AS1 0.001640795 0.011011364 2.746526004 7.20E-11 1.64E-10

MARCO 0.513380503 2.577398728 2.327815329 7.27E-11 1.65E-10

AL078644.1 0.010166636 0.075716095 2.896757681 7.40E-11 1.68E-10

AL357874.2 0.056267151 0.196457798 1.80385462 7.43E-11 1.69E-10

AC016747.4 0.017956245 0.057633044 1.682410561 7.44E-11 1.69E-10

AC097637.2 0.014280908 0.044984091 1.655327183 7.49E-11 1.70E-10

AC024937.1 0.047986611 0.153493417 1.677472961 7.51E-11 1.70E-10

PNPLA1 1.718769045 0.322536761 -2.413840195 7.73E-11 1.75E-10

Z82195.2 0.000922184 0.006612331 2.842032188 7.84E-11 1.78E-10

AC011939.2 0.008456496 0.240638367 4.830662878 7.92E-11 1.80E-10

AC008026.3 0.012312747 0.07829481 2.668763953 7.92E-11 1.80E-10

PPIAP51 0.041375373 0.23971207 2.534458319 7.94E-11 1.80E-10

MIR6730 0.183237918 0.671382605 1.873417083 7.99E-11 1.81E-10

AC008967.1 0.017337132 0.170116177 3.294583146 8.08E-11 1.83E-10

AMD1P4 0.004544392 0.055047049 3.598506066 8.12E-11 1.84E-10

MIR514B 0.390470938 1.31106583 1.747453048 8.17E-11 1.85E-10

ARMS2 0.037910412 0.10944086 1.529485433 8.25E-11 1.87E-10

GOLGA8S 0.007578996 0.026478753 1.804756489 8.29E-11 1.88E-10

RNU7-75P 0.103852439 0.697939929 2.748567773 8.52E-11 1.93E-10

AC091180.3 0.040413666 0.116062844 1.521991052 8.53E-11 1.93E-10

AC211476.4 0.047832725 0.19410811 2.020790505 8.53E-11 1.93E-10

U2AF1 0.034715389 0.108765391 1.647572337 8.56E-11 1.94E-10

CBLN4 0.384914935 1.655152095 2.104352239 8.78E-11 1.99E-10

AC008870.3 0.004812828 0.034878047 2.857362463 8.84E-11 2.00E-10

RNU6-1099P 0.241185069 0.782082548 1.697180295 8.89E-11 2.01E-10

AP005212.2 0.059479456 0.265345937 2.157411114 8.92E-11 2.02E-10

RN7SL809P 0.165499822 0.548611778 1.728955928 8.95E-11 2.02E-10

C9orf131 0.007960397 0.05280722 2.729822936 9.02E-11 2.04E-10

MYRF-AS1 0.022705392 0.066603796 1.552569461 9.05E-11 2.04E-10

AC010655.5 0.127297734 0.367286702 1.528699918 9.09E-11 2.05E-10

ADAM29 0.002577902 0.016607975 2.687606593 9.14E-11 2.06E-10

CHCHD2P11 0.013609562 0.105355467 2.952572598 9.15E-11 2.07E-10

AP005060.1 0.003108943 0.095691681 4.94389745 9.24E-11 2.09E-10

AL035420.1 0.028644485 0.086046275 1.586855333 9.39E-11 2.12E-10

AC141586.5 0.032869183 0.09878149 1.587505122 9.54E-11 2.15E-10

LCN1P1 0.070331777 0.21768309 1.629980763 9.62E-11 2.17E-10

AC087761.1 0.012918899 0.110988054 3.102849422 9.67E-11 2.18E-10

AL022323.4 0.003781945 0.030842073 3.02769956 9.67E-11 2.18E-10

AL445435.1 0.029073125 0.15861818 2.447800075 9.79E-11 2.21E-10

RPL7P5 0.003214693 0.051107231 3.990774418 9.79E-11 2.21E-10

AL137186.1 0.037052548 0.135879439 1.874682511 9.79E-11 2.21E-10

TCTE1 0.022716186 0.068223276 1.586543378 9.90E-11 2.23E-10

AC243654.2 0.028207057 0.151831902 2.428346892 1.00E-10 2.26E-10

AC132938.3 0.018650825 0.091627396 2.296539574 1.00E-10 2.26E-10

AL929236.1 0.055903287 0.161792493 1.533139651 1.01E-10 2.28E-10

GOLGA6L4 0.009698262 0.032150761 1.729054685 1.02E-10 2.29E-10

SNORA69 0.038124078 0.243211054 2.673434439 1.02E-10 2.30E-10

RHOXF1 0.137017054 0.447243591 1.706705335 1.04E-10 2.35E-10

TRBV12-5 0.025823992 0.185474442 2.844436488 1.05E-10 2.36E-10

AC120498.2 0.036224045 0.244667768 2.755804487 1.06E-10 2.38E-10

AC092794.2 0.026992111 0.141289469 2.388044243 1.07E-10 2.40E-10

AL021937.3 0.013235791 0.19017853 3.844838066 1.07E-10 2.41E-10

AL590560.3 0.511989417 1.731293808 1.757664681 1.08E-10 2.43E-10

RNU6-610P 0.184218368 0.705299338 1.936818768 1.10E-10 2.47E-10

RPL17P26 0.022446678 0.179052131 2.995805853 1.12E-10 2.51E-10

AC078845.1 0.029023541 0.135072365 2.218437107 1.12E-10 2.52E-10

RNA5SP425 0.036669985 0.333501968 3.185023684 1.12E-10 2.52E-10

RN7SL32P 0.033251636 0.161050742 2.276018073 1.13E-10 2.53E-10

OR7E13P 0.056316083 0.173041875 1.6195023 1.13E-10 2.54E-10

NUCB1-AS1 0.023256879 0.119030077 2.355596775 1.13E-10 2.54E-10

NPM1P35 0.018609091 0.065483367 1.81512094 1.14E-10 2.56E-10

TMEM248P1 0.008697715 0.042749109 2.297185987 1.16E-10 2.60E-10

AC010336.2 0.754044821 0.249261096 -1.596992551 1.16E-10 2.61E-10

CR559946.1 0.018045395 0.075558705 2.06596722 1.17E-10 2.61E-10

AC244093.1 0.007856127 0.048186879 2.616750307 1.18E-10 2.64E-10
[truncated: 195,585 more chars]
