# Supplementary material for: TIMP1 shapes an immunosuppressive microenvironment by regulating anoikis to promote the progression of clear cell renal cell carcinoma
Source: Aging (Albany NY). 2023 Sep 8;15(17):8908–29. doi: 10.18632/aging.205005 (PMC10522382; doi:10.18632/aging.205005)
Supplement: Supplementary Table 3 [file aging-15-205005-s004.docx]

**Supplementary Table 3. Results of univariate Cox regression analysis for ARGs.**

| Unicox | HR | HR.95L | HR.95H | pvalue |
| --- | --- | --- | --- | --- |
| PLK1 | 1.259773595 | 1.197120559 | 1.325705668 | 7.14E-19 |
| PLAUR | 1.066045199 | 1.050103984 | 1.082228412 | 8.81E-17 |
| CDK1 | 1.184223117 | 1.133164782 | 1.237582048 | 5.50E-14 |
| BUB1 | 1.351787855 | 1.247421724 | 1.464885828 | 1.94E-13 |
| BID | 1.225586624 | 1.160564989 | 1.29425115 | 2.60E-13 |
| SHC1 | 1.02181354 | 1.015765152 | 1.027897943 | 1.05E-12 |
| BIRC5 | 1.11516225 | 1.081994382 | 1.149346859 | 1.49E-12 |
| CHEK2 | 1.558259475 | 1.377468194 | 1.762779425 | 1.79E-12 |
| RAD9A | 1.219318822 | 1.152008437 | 1.290562068 | 7.70E-12 |
| AR | 0.769004124 | 0.712711991 | 0.829742378 | 1.27E-11 |
| PBK | 1.266414999 | 1.18225247 | 1.356568914 | 1.68E-11 |
| UBE2C | 1.020154993 | 1.014240125 | 1.026104355 | 1.75E-11 |
| CDKN2A | 1.072104845 | 1.049547285 | 1.095147228 | 1.39E-10 |
| ITGA6 | 0.968812013 | 0.959306974 | 0.97841123 | 3.00E-10 |
| TIMP1 | 1.000852612 | 1.000582974 | 1.001122322 | 5.67E-10 |
| CD44 | 1.009746966 | 1.006650817 | 1.012852637 | 5.99E-10 |
| GNE | 0.870026462 | 0.831736389 | 0.910079268 | 1.34E-09 |
| CDC25C | 1.602554888 | 1.375755885 | 1.866742638 | 1.38E-09 |
| CDKN3 | 1.087037484 | 1.057450726 | 1.11745206 | 3.07E-09 |
| LGALS1 | 1.001560186 | 1.001043493 | 1.002077146 | 3.19E-09 |
| ZNF304 | 0.698403666 | 0.619805879 | 0.786968463 | 3.80E-09 |
| CDK11A | 1.654792186 | 1.398912049 | 1.957476298 | 4.18E-09 |
| BCL2 | 0.933394405 | 0.911984252 | 0.955307194 | 5.82E-09 |
| TUBB3 | 1.609156611 | 1.36843004 | 1.892230455 | 8.73E-09 |
| EZH2 | 1.371114157 | 1.228054942 | 1.5308387 | 1.98E-08 |
| HMGA1 | 1.004716271 | 1.003063469 | 1.006371797 | 2.13E-08 |
| CTNND1 | 0.971760376 | 0.962017978 | 0.981601437 | 2.52E-08 |
| KDR | 0.982109859 | 0.975883125 | 0.988376324 | 2.65E-08 |
| MYBBP1A | 1.14023364 | 1.087304392 | 1.195739449 | 6.25E-08 |
| CEBPB | 1.013715409 | 1.008720377 | 1.018735176 | 6.48E-08 |
| CTNNA1 | 0.974369218 | 0.96521493 | 0.983610327 | 7.00E-08 |
| IGF1R | 0.895976446 | 0.86063692 | 0.932767086 | 8.80E-08 |
| ITGA8 | 0.85006847 | 0.800857695 | 0.902303127 | 9.36E-08 |
| SPHK1 | 1.085170282 | 1.052740616 | 1.118598943 | 1.29E-07 |
| HOTAIR | 1.415938471 | 1.243250395 | 1.612613004 | 1.60E-07 |
| CTNNB1 | 0.978004294 | 0.969851682 | 0.986225438 | 1.91E-07 |
| KLF12 | 0.734411717 | 0.653718041 | 0.825066061 | 2.01E-07 |
| CTBP1 | 1.113316175 | 1.06906057 | 1.159403817 | 2.14E-07 |
| PDPK1 | 0.68091093 | 0.588623328 | 0.787667889 | 2.32E-07 |
| KL | 0.963418301 | 0.949711113 | 0.977323325 | 3.45E-07 |
| SIK2 | 0.893434162 | 0.854941297 | 0.933660129 | 5.31E-07 |
| PIK3R3 | 0.921911572 | 0.892484376 | 0.952309049 | 9.00E-07 |
| DOCK1 | 0.906456448 | 0.87149447 | 0.942821006 | 9.89E-07 |
| SRC | 1.069046255 | 1.040718444 | 1.098145135 | 1.10E-06 |
| CPT1A | 0.952773615 | 0.934342542 | 0.971568263 | 1.21E-06 |
| AKT3 | 0.908092102 | 0.873325674 | 0.944242555 | 1.30E-06 |
| PYCARD | 1.033639509 | 1.019842665 | 1.047623002 | 1.39E-06 |
| DYNLL2 | 0.93511153 | 0.909955503 | 0.960963003 | 1.42E-06 |
| QSOX1 | 1.013845192 | 1.008167864 | 1.01955449 | 1.59E-06 |
| SERPINE1 | 1.000985813 | 1.000581239 | 1.001390552 | 1.78E-06 |
| SLCO1B3 | 5.363031245 | 2.686726745 | 10.70525843 | 1.91E-06 |
| RHOB | 0.997962289 | 0.99712149 | 0.998803798 | 2.10E-06 |
| ANXA2 | 1.011424822 | 1.006682994 | 1.016188985 | 2.16E-06 |
| PRKD1 | 0.817863191 | 0.752513379 | 0.888888115 | 2.22E-06 |
| CALR | 1.002047465 | 1.001195264 | 1.00290039 | 2.46E-06 |
| DAP3 | 1.076588144 | 1.044029721 | 1.110161912 | 2.48E-06 |
| MAPK1 | 0.947773141 | 0.926765378 | 0.969257104 | 2.73E-06 |
| ILK | 0.82158164 | 0.75628357 | 0.892517594 | 3.30E-06 |
| IRF6 | 0.885589886 | 0.841377002 | 0.93212608 | 3.32E-06 |
| ETV4 | 1.184655592 | 1.10171616 | 1.273838873 | 4.75E-06 |
| E2F1 | 1.153147235 | 1.084852918 | 1.225740857 | 4.77E-06 |
| TRAF2 | 1.140180132 | 1.077466607 | 1.206543874 | 5.50E-06 |
| TLN1 | 0.980900209 | 0.97274001 | 0.989128863 | 6.05E-06 |
| EZR | 0.996090428 | 0.994401514 | 0.99778221 | 6.06E-06 |
| SIRT1 | 0.865713038 | 0.812898664 | 0.921958785 | 7.12E-06 |
| SNAI2 | 1.065657675 | 1.036361893 | 1.095781588 | 7.78E-06 |
| MAD2L1 | 1.365676713 | 1.191080654 | 1.565866156 | 7.99E-06 |
| MNX1 | 2.11924576 | 1.523207723 | 2.948516164 | 8.29E-06 |
| TNFRSF1A | 1.021992985 | 1.012245058 | 1.031834785 | 8.63E-06 |
| PITPNC1 | 0.88748324 | 0.841638696 | 0.935824963 | 1.03E-05 |
| PTK2 | 0.879949901 | 0.830968238 | 0.931818801 | 1.21E-05 |
| SMAD4 | 0.820344776 | 0.750669328 | 0.896487344 | 1.23E-05 |
| TLR3 | 0.959308333 | 0.941521227 | 0.97743147 | 1.36E-05 |
| RHOG | 1.027678228 | 1.015091493 | 1.040421034 | 1.41E-05 |
| F3 | 1.013555511 | 1.007323318 | 1.019826262 | 1.88E-05 |
| RHOA | 0.991813061 | 0.988072831 | 0.995567448 | 2.00E-05 |
| ROCK1 | 0.879665517 | 0.829219365 | 0.933180597 | 2.09E-05 |
| CASP9 | 1.239188904 | 1.122475707 | 1.36803775 | 2.15E-05 |
| RB1 | 0.944724443 | 0.920072103 | 0.970037315 | 2.50E-05 |
| PTRH2 | 1.287079278 | 1.143901178 | 1.448178478 | 2.74E-05 |
| PPP1R13B | 0.791855255 | 0.70978523 | 0.883414755 | 2.91E-05 |
| PLAU | 1.010374953 | 1.005488557 | 1.015285095 | 3.01E-05 |
| FER | 0.663883569 | 0.547550229 | 0.804933264 | 3.08E-05 |
| TWIST1 | 1.097536696 | 1.050451448 | 1.146732485 | 3.18E-05 |
| CASP3 | 1.101089295 | 1.052147361 | 1.152307822 | 3.31E-05 |
| JUP | 0.982679041 | 0.974577564 | 0.990847864 | 3.52E-05 |
| ERBB2 | 0.943770617 | 0.918032421 | 0.970230415 | 4.09E-05 |
| SIRT6 | 1.125720699 | 1.063635638 | 1.191429703 | 4.29E-05 |
| CDCP1 | 1.029853313 | 1.015400612 | 1.044511726 | 4.51E-05 |
| PDK4 | 0.997464482 | 0.996245281 | 0.998685175 | 4.73E-05 |
| FOXO3 | 0.924793059 | 0.89050851 | 0.960397563 | 4.98E-05 |
| LTB4R2 | 1.554559348 | 1.254007479 | 1.927145418 | 5.70E-05 |
| PIK3CA | 0.80668172 | 0.726292428 | 0.895968858 | 6.05E-05 |
| MAPK8 | 0.723326713 | 0.616832911 | 0.848206257 | 6.72E-05 |
| CDC42 | 0.979252695 | 0.96917915 | 0.989430944 | 7.07E-05 |
| CEMIP | 1.040600566 | 1.020353238 | 1.061249671 | 7.19E-05 |
| BAG1 | 0.886518538 | 0.835253818 | 0.940929692 | 7.39E-05 |
| BCL2L2 | 0.93902525 | 0.909830942 | 0.969156334 | 9.46E-05 |
| TGFB1 | 1.009681285 | 1.004798335 | 1.014587964 | 9.81E-05 |
| SCRIB | 1.081455735 | 1.039637929 | 1.124955597 | 9.94E-05 |
| CCND1 | 0.996944312 | 0.995407603 | 0.998483394 | 0.000100927 |
| RBL2 | 0.937205911 | 0.906789042 | 0.968643067 | 0.000116891 |
| EDAR | 0.245163426 | 0.119749967 | 0.501921687 | 0.000120305 |
| OCLN | 0.706167123 | 0.591393375 | 0.843215408 | 0.000120862 |
| BRAF | 0.791723545 | 0.702438219 | 0.892357726 | 0.000130523 |
| SKP2 | 1.126143101 | 1.058698987 | 1.197883723 | 0.000163099 |
| CXCL8 | 1.009150785 | 1.004356327 | 1.01396813 | 0.000177576 |
| PPARG | 0.871507949 | 0.810694528 | 0.936883227 | 0.000194125 |
| MTA1 | 1.122386586 | 1.055189566 | 1.193862875 | 0.000246923 |
| EEF1A1 | 0.999339714 | 0.998985319 | 0.999694234 | 0.000262414 |
| PLG | 0.914355781 | 0.871221521 | 0.959625622 | 0.000281786 |
| NQO1 | 1.015720506 | 1.007151225 | 1.024362699 | 0.000308094 |
| F10 | 1.011605209 | 1.005257145 | 1.01799336 | 0.000327522 |
| SH3GLB1 | 0.956975966 | 0.933984859 | 0.980533026 | 0.000393481 |
| BUB3 | 1.09070657 | 1.039576546 | 1.144351349 | 0.000393488 |
| CEACAM4 | 1.161685845 | 1.068506087 | 1.262991405 | 0.000442687 |
| S100A7 | 1.514679288 | 1.201487245 | 1.909511195 | 0.00044293 |
| RAC3 | 1.077076208 | 1.033370374 | 1.122630557 | 0.00044294 |
| OGT | 1.02469954 | 1.010755971 | 1.038835464 | 0.000482246 |
| TFDP1 | 0.936917993 | 0.903019161 | 0.972089367 | 0.000529259 |
| AFAP1L1 | 0.944543273 | 0.914455522 | 0.975620983 | 0.000551823 |
| PTEN | 0.920334335 | 0.877839722 | 0.964886035 | 0.000577466 |
| ITGA2 | 0.9179543 | 0.874017757 | 0.964099516 | 0.000623985 |
| MAP3K1 | 0.901751404 | 0.849820362 | 0.956855861 | 0.000632492 |
| HMCN1 | 0.800450281 | 0.704293489 | 0.909735305 | 0.000652591 |
| PHLDA2 | 1.052575633 | 1.021856804 | 1.084217924 | 0.000697105 |
| MMP13 | 1.02790398 | 1.011664023 | 1.044404632 | 0.000706148 |
| CEACAM1 | 0.901860107 | 0.849467887 | 0.957483697 | 0.000717594 |
| PDCD6IP | 0.923972142 | 0.882564327 | 0.967322714 | 0.00072445 |
| RHOQ | 1.03918159 | 1.016181203 | 1.062702571 | 0.000763765 |
| ITGAV | 0.984975623 | 0.976321736 | 0.993706215 | 0.000773163 |
| NTRK1 | 2.173751212 | 1.380965524 | 3.42165988 | 0.000795201 |
| BNIP3L | 0.989987689 | 0.984161392 | 0.995848477 | 0.000833724 |
| ZEB1 | 0.927630403 | 0.887483175 | 0.969593778 | 0.00087523 |
| HGF | 1.016877851 | 1.006873288 | 1.026981822 | 0.000907269 |
| PTHLH | 1.006465425 | 1.002610724 | 1.010334945 | 0.000995839 |
| BNIP3 | 0.989938632 | 0.983993025 | 0.995920165 | 0.001001663 |
| NDRG1 | 0.997759032 | 0.996420921 | 0.999098939 | 0.001050938 |
| BAK1 | 1.050691278 | 1.019907127 | 1.082404597 | 0.001117411 |
| RPS6KA3 | 0.930381316 | 0.890851402 | 0.971665299 | 0.001123801 |
| APOBEC3G | 1.058192239 | 1.022617249 | 1.095004818 | 0.001187712 |
| MGAT5 | 0.931599595 | 0.892523333 | 0.972386686 | 0.001192144 |
| XAF1 | 1.094933352 | 1.036308918 | 1.156874196 | 0.001236734 |
| MAP2K2 | 1.02640649 | 1.010282489 | 1.042787828 | 0.001254186 |
| PIK3CB | 0.933638309 | 0.895391909 | 0.973518393 | 0.001292818 |
| MMP11 | 1.050188936 | 1.019315892 | 1.081997062 | 0.001296969 |
| ABHD2 | 0.959636693 | 0.935707079 | 0.984178278 | 0.001384851 |
| IQGAP1 | 0.972578618 | 0.956089828 | 0.989351775 | 0.001437304 |
| PIK3C2B | 0.880755161 | 0.814525187 | 0.95237037 | 0.001455084 |
| NRAS | 0.965416447 | 0.944608892 | 0.986682344 | 0.001545588 |
| ITPRIP | 0.968641374 | 0.949692868 | 0.987967945 | 0.001572838 |
| HSPB1 | 1.000780834 | 1.000295948 | 1.001265955 | 0.001595716 |
| CBL | 0.863701494 | 0.78798951 | 0.946688074 | 0.001745736 |
| CSK | 1.047864254 | 1.017392552 | 1.079248606 | 0.001901799 |
| GLI2 | 1.479223673 | 1.152735907 | 1.898182107 | 0.002090388 |
| SESN1 | 0.902483657 | 0.845344264 | 0.963485276 | 0.002107602 |
| KRAS | 0.891058368 | 0.827210141 | 0.959834721 | 0.002361014 |
| TNFRSF10B | 1.021835404 | 1.007687928 | 1.036181504 | 0.002392579 |
| ELK1 | 1.038598607 | 1.013336927 | 1.06449004 | 0.002573787 |
| NFE2L2 | 0.968141928 | 0.94787545 | 0.988841722 | 0.002703912 |
| EDA2R | 0.911432793 | 0.857693616 | 0.968539021 | 0.002781224 |
| SPIB | 1.371602617 | 1.113672254 | 1.689270548 | 0.002949837 |
| MMP2 | 1.00718339 | 1.002415615 | 1.011973841 | 0.00311102 |
| ATF4 | 1.00570392 | 1.001906059 | 1.009516176 | 0.003214683 |
| MAPK3 | 0.96595571 | 0.943932283 | 0.988492978 | 0.003245113 |
| FN1 | 1.001512578 | 1.000500608 | 1.002525571 | 0.003386718 |
| ANXA5 | 1.006371428 | 1.00209985 | 1.010661214 | 0.003427764 |
| XIAP | 0.896026701 | 0.831749839 | 0.96527082 | 0.0038446 |
| GLUD1 | 0.991456578 | 0.985675982 | 0.997271075 | 0.004028779 |
| HSP90B1 | 1.002279343 | 1.000723674 | 1.003837431 | 0.004069173 |
| CDKN1B | 0.974809132 | 0.957883708 | 0.992033623 | 0.004304067 |
| ATF2 | 0.929017441 | 0.883032136 | 0.977397504 | 0.004474562 |
| RAC1 | 1.008560145 | 1.002537651 | 1.014618818 | 0.005281444 |
| PCNA | 1.013527731 | 1.0040007 | 1.023145164 | 0.005294206 |
| GSK3B | 0.910962354 | 0.852301566 | 0.973660549 | 0.006033514 |
| PPP2R5A | 0.957252079 | 0.927847953 | 0.987588041 | 0.006058791 |
| NTRK2 | 0.884994752 | 0.809974715 | 0.966963162 | 0.006865235 |
| PXN | 1.017994652 | 1.004844774 | 1.031316615 | 0.007176511 |
| STK11 | 1.138971403 | 1.035043684 | 1.253334402 | 0.007687102 |
| BST2 | 1.001458825 | 1.000371194 | 1.002547639 | 0.008554539 |
| SETD2 | 0.903208695 | 0.836270028 | 0.975505422 | 0.009564275 |
| MYO5A | 0.878449947 | 0.795959389 | 0.96948955 | 0.010000095 |
| SOD2 | 1.002454547 | 1.000578395 | 1.004334217 | 0.010319424 |
| LATS1 | 0.882003542 | 0.801072073 | 0.971111433 | 0.010560233 |
| CD151 | 1.004303919 | 1.000987909 | 1.007630915 | 0.010923599 |
| TSG101 | 0.950372532 | 0.913617696 | 0.988606015 | 0.011425445 |
| THBS1 | 0.994020431 | 0.989409267 | 0.998653085 | 0.01146799 |
| NOTCH1 | 0.954104414 | 0.91983283 | 0.989652905 | 0.011828029 |
| MMP9 | 1.002251339 | 1.000497728 | 1.004008023 | 0.011839686 |
| CDKN1A | 0.994787679 | 0.990713512 | 0.9988786 | 0.012566427 |
| RHOC | 1.008514036 | 1.001722813 | 1.0153513 | 0.013921398 |
| ID2 | 0.993481131 | 0.988313782 | 0.998675497 | 0.013967399 |
| MTOR | 0.864782675 | 0.769977062 | 0.971261498 | 0.014200466 |
| BRMS1 | 1.027367764 | 1.005432793 | 1.049781278 | 0.014206103 |
| CD36 | 0.98489522 | 0.972782545 | 0.997158717 | 0.015925402 |
| HAVCR2 | 0.989895254 | 0.981734977 | 0.998123361 | 0.016184725 |
| ITGA5 | 1.009479203 | 1.001719712 | 1.017298801 | 0.016557019 |
| ITGB4 | 1.013537663 | 1.00238261 | 1.024816856 | 0.017246068 |
| ACP1 | 0.960032288 | 0.928037407 | 0.99313022 | 0.01834509 |
| FGF2 | 1.05240929 | 1.008427197 | 1.098309642 | 0.0190145 |
| CDK11B | 1.118933373 | 1.018264183 | 1.229555074 | 0.019478635 |
| PPP2CA | 0.971110117 | 0.947509954 | 0.995298103 | 0.019521165 |
| PTK6 | 1.072493835 | 1.010822631 | 1.137927654 | 0.020546479 |
| FASLG | 1.079052199 | 1.011304638 | 1.151338186 | 0.021462361 |
| SATB1 | 0.831521768 | 0.710380133 | 0.973321772 | 0.021644238 |
| BAX | 1.021335326 | 1.002813445 | 1.040199304 | 0.023768997 |
| BIRC3 | 1.006948716 | 1.000906576 | 1.01302733 | 0.024129451 |
| ABHD4 | 1.031427579 | 1.003923343 | 1.059685342 | 0.024838137 |
| TP53 | 1.043641651 | 1.005418381 | 1.083318065 | 0.024844319 |
| PDCD4 | 0.962582326 | 0.931019465 | 0.995215212 | 0.024966014 |
| DAPK1 | 0.956019797 | 0.919025254 | 0.994503523 | 0.025503462 |
| GLO1 | 0.986658726 | 0.975020385 | 0.998435989 | 0.02652042 |
| RBFOX2 | 0.951854711 | 0.911238705 | 0.994281065 | 0.026572397 |
| EPHA2 | 0.981971483 | 0.966202778 | 0.997997537 | 0.027619609 |
| ANKRD13C | 0.900264617 | 0.819638072 | 0.988822272 | 0.028179775 |
| SIRT3 | 0.892450262 | 0.805885724 | 0.988313164 | 0.028831035 |
| IL6 | 1.00437066 | 1.000444661 | 1.008312065 | 0.029077396 |
| CEACAM3 | 1.580124261 | 1.043486811 | 2.392740046 | 0.030692667 |
| BMP6 | 0.925047106 | 0.861785708 | 0.992952354 | 0.031110975 |
| PIK3R1 | 0.966830008 | 0.937592512 | 0.996979234 | 0.031313694 |
| HMOX1 | 0.997769999 | 0.995729217 | 0.999814963 | 0.032588874 |
| PTPN11 | 0.972997884 | 0.948790269 | 0.997823138 | 0.033213215 |
| SLC2A1 | 1.003068312 | 1.000210902 | 1.005933886 | 0.035304505 |
| ACTG1 | 1.000717848 | 1.000048453 | 1.001387692 | 0.035563583 |
| CASP2 | 1.11768529 | 1.005039096 | 1.242957029 | 0.040101698 |
| ARHGEF7 | 0.941700855 | 0.888985591 | 0.997542041 | 0.040983722 |
| CASP6 | 1.072214238 | 1.002842913 | 1.1463843 | 0.041037535 |
| CASP8 | 1.091721474 | 1.002947787 | 1.188352765 | 0.042561735 |
| PIK3R2 | 1.427040314 | 1.010843794 | 2.01459817 | 0.043251921 |
| SESN3 | 0.955083573 | 0.913033396 | 0.99907039 | 0.045451907 |
| ITGA3 | 1.00401021 | 1.000077313 | 1.007958574 | 0.045654894 |
| CLDN1 | 1.006206918 | 1.000110428 | 1.01234057 | 0.045980125 |
| CTTN | 1.020880155 | 1.000332475 | 1.041849902 | 0.046370246 |
| RIPK1 | 0.95464784 | 0.91138259 | 0.999966982 | 0.049837123 |
| CDH1 | 0.988120155 | 0.976367942 | 1.000013826 | 0.050265325 |
| NGF | 1.01899898 | 0.999840563 | 1.038524501 | 0.05195594 |
| ITGB1 | 0.995555373 | 0.991065848 | 1.000065235 | 0.053399472 |
| MAPK11 | 1.024752934 | 0.999637229 | 1.050499667 | 0.053445308 |
| MAVS | 0.954528782 | 0.909827869 | 1.001425905 | 0.057205619 |
| CAV1 | 1.003623803 | 0.999885277 | 1.007376307 | 0.057471134 |
| SP1 | 0.975253729 | 0.950269036 | 1.000895325 | 0.058440594 |
| MYH9 | 0.99732471 | 0.994549218 | 1.000107948 | 0.059558403 |
| CD63 | 1.000960173 | 0.999930392 | 1.001991015 | 0.067637115 |
| SRSF3 | 0.977557967 | 0.953894091 | 1.001808889 | 0.069460095 |
| ITGA4 | 0.948413448 | 0.895619792 | 1.004319106 | 0.069912022 |
| EHMT2 | 1.063020761 | 0.994568441 | 1.136184392 | 0.071925461 |
| IKBKG | 1.513612025 | 0.961917678 | 2.381722899 | 0.073117433 |
| STK38 | 1.034135249 | 0.996529813 | 1.073159779 | 0.075728084 |
| LRP1 | 1.012297266 | 0.998654298 | 1.026126615 | 0.077487764 |
| PRKCQ | 0.927570107 | 0.853271249 | 1.008338562 | 0.077559339 |
| PRKACA | 1.045027359 | 0.994795145 | 1.097796051 | 0.079715703 |
| CCDC178 | 0.68211811 | 0.442722742 | 1.050962762 | 0.082814807 |
| PIK3CG | 0.8977559 | 0.794290665 | 1.014698639 | 0.084274764 |
| SIRPA | 1.006609769 | 0.999099847 | 1.014176142 | 0.084660312 |
| CSPG4 | 0.988339722 | 0.9752209 | 1.001635022 | 0.085370611 |
| S100A4 | 1.001179123 | 0.999830982 | 1.002529082 | 0.086510976 |
| BDNF | 0.806961937 | 0.628987228 | 1.035295374 | 0.091581438 |
| FBLIM1 | 1.013681626 | 0.997671536 | 1.029948637 | 0.094334018 |
| SERPINA1 | 1.000225746 | 0.999960575 | 1.000490987 | 0.095210489 |
| PTGS2 | 1.008253631 | 0.998519354 | 1.018082805 | 0.096791417 |
| TNFRSF12A | 1.002143376 | 0.99960899 | 1.004684188 | 0.097468305 |
| HRAS | 1.009412884 | 0.998208017 | 1.020743526 | 0.099962466 |
| GRHL2 | 0.862792112 | 0.722015309 | 1.031017237 | 0.104405178 |
| PTPN1 | 1.011078974 | 0.997715467 | 1.024621474 | 0.104578999 |
| TPM1 | 0.992634152 | 0.983776008 | 1.001572056 | 0.105985454 |
| LTF | 0.99729427 | 0.993996171 | 1.000603312 | 0.108912054 |
| SFN | 1.003963704 | 0.999103299 | 1.008847753 | 0.110120453 |
| TSC2 | 1.067228193 | 0.984265329 | 1.157183924 | 0.115061294 |
| ZBTB7A | 0.944992027 | 0.88047064 | 1.014241578 | 0.116869345 |
| IFI27 | 1.003728874 | 0.999011295 | 1.008468729 | 0.12151844 |
| CXCR4 | 1.001959826 | 0.999457855 | 1.004468061 | 0.124820959 |
| PTK2B | 1.057331754 | 0.98452373 | 1.135524115 | 0.12564932 |
| NOX4 | 0.974743912 | 0.943195048 | 1.007348051 | 0.12754905 |
| EGFR | 0.993216984 | 0.984531741 | 1.001978846 | 0.12880952 |
| NTF3 | 1.144452886 | 0.956183144 | 1.369792404 | 0.141193422 |
| MIR30C1 | 0.357248246 | 0.090060125 | 1.417123391 | 0.143171165 |
| BCL2L11 | 0.950900764 | 0.888567391 | 1.01760685 | 0.145556407 |
| CRYAB | 1.000384613 | 0.99986609 | 1.000903405 | 0.146029022 |
| MAP2K1 | 0.975063764 | 0.942144421 | 1.009133337 | 0.149554312 |
| EIF2AK3 | 0.94427124 | 0.873359119 | 1.020941048 | 0.149968992 |
| TLE5 | 1.004110313 | 0.998479784 | 1.009772594 | 0.152804283 |
| TP73 | 1.168092374 | 0.942266762 | 1.448039822 | 0.156351175 |
| COL13A1 | 1.155060642 | 0.942714348 | 1.415237915 | 0.164285203 |
| BAG4 | 0.932422514 | 0.843475155 | 1.030749679 | 0.171351132 |
| YAP1 | 0.982496195 | 0.957794501 | 1.007834951 | 0.174070636 |
| TDGF1 | 0.908615452 | 0.789972388 | 1.045077084 | 0.179475781 |
| RELA | 1.028076421 | 0.987101349 | 1.070752388 | 0.182091913 |
| ADCY10 | 1.296278412 | 0.884184052 | 1.900438851 | 0.183721363 |
| MDM2 | 0.97189694 | 0.931747065 | 1.013776911 | 0.185405811 |
| TP63 | 1.848784301 | 0.742083022 | 4.605958216 | 0.187007829 |
| MTDH | 0.98669599 | 0.967187292 | 1.00659819 | 0.188676974 |
| MSLN | 0.997697834 | 0.994243167 | 1.001164505 | 0.192799596 |
| AKT2 | 1.050128922 | 0.975236177 | 1.130773016 | 0.195075868 |
| MAOA | 0.993928262 | 0.984620002 | 1.00332452 | 0.204580511 |
| YWHAZ | 1.005169484 | 0.997117614 | 1.013286374 | 0.208924333 |
| PRKCI | 0.961525501 | 0.903784455 | 1.022955511 | 0.214353902 |
| CCDC80 | 1.011054513 | 0.993645656 | 1.028768376 | 0.214748572 |
| CDH2 | 0.990890559 | 0.97660912 | 1.005380843 | 0.21665741 |
| FOXC2 | 0.979560456 | 0.947694981 | 1.012497383 | 0.220990685 |
| TNC | 1.004992649 | 0.996980087 | 1.013069607 | 0.222686578 |
| PRPF4B | 0.967851146 | 0.918197196 | 1.020190265 | 0.223958192 |
| COL4A2 | 0.998884687 | 0.997087164 | 1.00068545 | 0.224621205 |
| PAK4 | 1.036296488 | 0.977759705 | 1.098337767 | 0.229434708 |
| ONECUT1 | 1.252863774 | 0.865922853 | 1.812710717 | 0.231647567 |
| SPP1 | 1.000094624 | 0.999937494 | 1.00025178 | 0.237898389 |
| VPS37A | 0.961240934 | 0.899684931 | 1.027008569 | 0.241717694 |
| FAS | 0.98087501 | 0.94949205 | 1.013295252 | 0.244466951 |
| MIR204 | 0.325157554 | 0.046396027 | 2.278803613 | 0.258109785 |
| MIR26A1 | 0.505563773 | 0.153746147 | 1.6624464 | 0.261413464 |
| FASN | 1.007108197 | 0.994729207 | 1.019641237 | 0.261660595 |
| MAP3K7 | 1.05310169 | 0.961944002 | 1.152897847 | 0.262691906 |
| PAK2 | 0.98474377 | 0.958456878 | 1.011751613 | 0.265426484 |
| EGF | 0.970325594 | 0.919911268 | 1.023502799 | 0.268473997 |
| BCAR1 | 1.018546523 | 0.98585922 | 1.052317612 | 0.269501585 |
| CDH3 | 1.017827837 | 0.986240892 | 1.050426436 | 0.271938877 |
| MIR181A1 | 0.073768965 | 0.000669182 | 8.132105514 | 0.277271699 |
| DLG1 | 0.963406703 | 0.900143185 | 1.031116484 | 0.282040117 |
| CCR7 | 1.048893592 | 0.960585067 | 1.145320499 | 0.287414803 |
| KRT14 | 1.04973342 | 0.957480812 | 1.150874503 | 0.3010552 |
| PLAT | 0.98478972 | 0.956454626 | 1.013964245 | 0.303492334 |
| SFRP1 | 0.986000822 | 0.959814057 | 1.012902044 | 0.304641585 |
| MCL1 | 0.998278454 | 0.994998673 | 1.001569046 | 0.304795992 |
| HK2 | 1.006886706 | 0.993736275 | 1.020211161 | 0.306216691 |
| MIR30B | 0.57175486 | 0.189802397 | 1.722336626 | 0.320400599 |
| ZNF32 | 1.010507086 | 0.989849748 | 1.031595524 | 0.321270259 |
| MET | 1.002552584 | 0.99747861 | 1.007652368 | 0.324740106 |
| CYCS | 0.996493676 | 0.989467563 | 1.00356968 | 0.330582637 |
| PRDX4 | 1.002911278 | 0.997021004 | 1.008836351 | 0.333407721 |
| MIR200C | 0.067636781 | 0.000278943 | 16.40022592 | 0.336313027 |
| CEACAM8 | 0.282666966 | 0.019349975 | 4.129236032 | 0.355756556 |
| CPEB2 | 0.989595946 | 0.967644896 | 1.012044955 | 0.360811549 |
| SESN2 | 1.018305085 | 0.978900438 | 1.059295925 | 0.367655496 |
| BAD | 1.003274014 | 0.99609527 | 1.010504494 | 0.372319807 |
| ARHGDIB | 1.00134711 | 0.998370543 | 1.004332551 | 0.375455025 |
| BMF | 1.033640813 | 0.960379663 | 1.112490581 | 0.377698046 |
| RANBP9 | 0.977001017 | 0.926874033 | 1.029838956 | 0.386579797 |
| CRABP2 | 1.001090876 | 0.998608644 | 1.003579278 | 0.389373758 |
| CXCL12 | 0.995401097 | 0.984844764 | 1.006070581 | 0.396787046 |
| PPP2R2D | 0.933749427 | 0.79669211 | 1.094385123 | 0.397355761 |
| FBXW7-AS1 | 0.600428071 | 0.17252448 | 2.089638921 | 0.422727009 |
| RPS6KB1 | 1.049472746 | 0.931631786 | 1.182219265 | 0.426839932 |
| CCN1 | 0.999425393 | 0.997971241 | 1.000881665 | 0.439113234 |
| ARHGDIA | 1.003282279 | 0.9948889 | 1.011746469 | 0.444571848 |
| VEGFA | 1.000707031 | 0.99886017 | 1.002557306 | 0.453313659 |
| LPAR1 | 1.016138642 | 0.972772978 | 1.061437522 | 0.471859619 |
| ANGPTL2 | 1.002309248 | 0.996004763 | 1.008653638 | 0.473698804 |
| CEACAM6 | 0.793065576 | 0.419566317 | 1.499055054 | 0.475398315 |
| SPINK1 | 0.997484327 | 0.990505594 | 1.00451223 | 0.481955289 |
| IGF1 | 0.843127176 | 0.523160704 | 1.35878599 | 0.483426358 |
| NOTCH3 | 0.997669472 | 0.991176282 | 1.004205199 | 0.483703463 |
| PAK1 | 0.981782606 | 0.932516624 | 1.03365137 | 0.483968288 |
| TLE1 | 0.98288483 | 0.936452568 | 1.03161935 | 0.484439205 |
| CCN6 | 1.043541853 | 0.925219721 | 1.176995662 | 0.487600876 |
| ZEB2 | 0.976192005 | 0.911461811 | 1.045519207 | 0.491233787 |
| LDHA | 0.999483241 | 0.997993849 | 1.000974855 | 0.496918039 |
| PIN1 | 0.976866174 | 0.912845829 | 1.045376438 | 0.498543588 |
| STAT3 | 0.993373471 | 0.974340207 | 1.01277854 | 0.500585246 |
| SIK1 | 1.043724721 | 0.921492549 | 1.182170484 | 0.500683011 |
| CFLAR | 0.987279412 | 0.950989575 | 1.024954073 | 0.502852158 |
| TRIM31 | 1.15885864 | 0.748087637 | 1.795181848 | 0.509098773 |
| MIR200B | 0.892544339 | 0.636496962 | 1.251593401 | 0.509893316 |
| MIR1827 | 0.86558723 | 0.562806724 | 1.331258531 | 0.51103882 |
| AFP | 1.084514564 | 0.849688661 | 1.384238597 | 0.514620194 |
| ADAMTSL1 | 0.977676303 | 0.913126299 | 1.046789425 | 0.517097317 |
| ELANE | 0.93246949 | 0.753817643 | 1.153461129 | 0.519366084 |
| RAF1 | 1.019682298 | 0.959850046 | 1.083244194 | 0.52754618 |
| SDCBP | 1.003002466 | 0.993636446 | 1.012456771 | 0.531114518 |
| PRKCA | 1.01422462 | 0.970041153 | 1.060420556 | 0.534256388 |
| ENDOG | 0.976935312 | 0.90709084 | 1.05215769 | 0.537521795 |
| NKX2-1 | 2.108561448 | 0.184023976 | 24.16006582 | 0.548799321 |
| MALAT1 | 0.999762373 | 0.998984572 | 1.000540779 | 0.549514202 |
| PARP1 | 1.007568504 | 0.982724325 | 1.033040768 | 0.553907269 |
| LAMA3 | 1.012202564 | 0.971803152 | 1.054281444 | 0.559466515 |
| MYC | 1.001608989 | 0.996157999 | 1.007089807 | 0.563657153 |
| CLU | 0.999832307 | 0.999244635 | 1.000420325 | 0.576116026 |
| MIR107 | 0.727086437 | 0.237375002 | 2.227086602 | 0.576824611 |
| MIR141 | 0.254769375 | 0.001937433 | 33.50176932 | 0.582797316 |
| MIR525 | 0.71463688 | 0.210492417 | 2.426243556 | 0.59006921 |
| HTRA1 | 1.000316495 | 0.999134731 | 1.001499657 | 0.599803943 |
| HIF1A | 1.002157321 | 0.993964503 | 1.010417668 | 0.606877075 |
| SMAD7 | 0.993803052 | 0.968106267 | 1.020181917 | 0.641879765 |
| FADD | 0.980952971 | 0.90291901 | 1.065730946 | 0.649318478 |
| MUC1 | 0.998778077 | 0.993392997 | 1.004192349 | 0.657576075 |
| SLC39A6 | 0.998159826 | 0.990035974 | 1.00635034 | 0.65867383 |
| LAMB3 | 0.997744629 | 0.987774454 | 1.007815439 | 0.65946633 |
| EEF2K | 0.983709406 | 0.914049805 | 1.058677756 | 0.661161358 |
| BRCA2 | 0.896771927 | 0.543387527 | 1.479974876 | 0.669921139 |
| SERPINB1 | 0.996656315 | 0.981369004 | 1.012181765 | 0.671069403 |
| CRYBA1 | 1.107851793 | 0.681328751 | 1.801385298 | 0.679648753 |
| VTN | 1.000499606 | 0.997970746 | 1.003034874 | 0.698888806 |
| KDM3A | 0.987646892 | 0.92602963 | 1.053364116 | 0.705291626 |
| CSNK2A1 | 0.994181138 | 0.963573995 | 1.025760492 | 0.714527368 |
| AKT1 | 0.988490943 | 0.928442921 | 1.052422635 | 0.717335342 |
| LGALS3 | 0.999789281 | 0.998648385 | 1.000931481 | 0.717536783 |
| EDIL3 | 1.00109471 | 0.994609946 | 1.007621753 | 0.741419433 |
| CD24 | 0.999903527 | 0.999328936 | 1.000478448 | 0.74218176 |
| ITGB5 | 0.997450322 | 0.982201021 | 1.012936378 | 0.745348393 |
| TAGLN | 1.000310245 | 0.998418821 | 1.002205252 | 0.748033735 |
| CCN2 | 0.999671281 | 0.997649382 | 1.001697278 | 0.750276434 |
| ABL1 | 0.994956226 | 0.962898155 | 1.028081617 | 0.762191257 |
| CASP10 | 1.015387286 | 0.91878064 | 1.122151791 | 0.764669633 |
| PDGFRB | 1.000938378 | 0.994810469 | 1.007104035 | 0.764670241 |
| RACK1 | 0.999838768 | 0.998508815 | 1.001170493 | 0.812322272 |
| TIAM1 | 1.021246095 | 0.854838354 | 1.220047721 | 0.816798391 |
| CCAR2 | 1.007465412 | 0.945556041 | 1.07342824 | 0.818201588 |
| INHBB | 1.000633615 | 0.993843219 | 1.007470406 | 0.855330344 |
| EPHB6 | 1.007995928 | 0.923529615 | 1.100187558 | 0.858440637 |
| PPP2R1A | 1.001519285 | 0.984811131 | 1.018510907 | 0.859614918 |
| FOXA1 | 0.995355431 | 0.934502619 | 1.060170848 | 0.884998718 |
| BCL2L1 | 0.999509721 | 0.992669995 | 1.006396575 | 0.888678358 |
| TNFSF10 | 0.999820402 | 0.99730012 | 1.002347052 | 0.88907039 |
| MIR200A | 0.99038932 | 0.863335415 | 1.136141282 | 0.8903498 |
| BSG | 0.999939521 | 0.998890464 | 1.000989679 | 0.910084917 |
| LAMC2 | 0.998725103 | 0.975492169 | 1.022511367 | 0.915401058 |
| ANGPTL4 | 1.000028945 | 0.99934869 | 1.000709663 | 0.933558015 |
| LMO3 | 0.989255181 | 0.70954042 | 1.379238992 | 0.949200277 |
| BIN1 | 1.000837903 | 0.974297331 | 1.028101459 | 0.95129649 |
| NTRK3 | 1.01122463 | 0.649384155 | 1.574684636 | 0.960603397 |
| PIP5K1C | 0.998835844 | 0.945273045 | 1.055433717 | 0.966959593 |
| CEACAM5 | 0.986518381 | 0.510805723 | 1.905261574 | 0.967759407 |
| BCL2L15 | 0.993567841 | 0.715320306 | 1.380048974 | 0.969295097 |
| GKN1 | 1.024098521 | 0.220452298 | 4.75739101 | 0.975757835 |
| DAPK2 | 0.997621123 | 0.83540528 | 1.191335425 | 0.97901374 |
| PAK3 | 1.004249853 | 0.675358597 | 1.493307069 | 0.983285646 |
| SMARCE1 | 0.998893003 | 0.89653604 | 1.112935998 | 0.983979233 |
| ITGB3 | 0.999935418 | 0.945089733 | 1.057963922 | 0.998209601 |
| OLFM3 | 1.003237936 | 0.044889366 | 22.42148757 | 0.998372794 |
| GDF2 | 0.990604938 | 2.27E-11 | 43307909264 | 0.999397508 |
