# Supplementary material for: TIMP1 shapes an immunosuppressive microenvironment by regulating anoikis to promote the progression of clear cell renal cell carcinoma
Source: Aging (Albany NY). 2023 Sep 8;15(17):8908–29. doi: 10.18632/aging.205005 (PMC10522382; doi:10.18632/aging.205005)
Supplement: Supplementary Tables 4 and 5 [file aging-15-205005-s005.pdf]

Supplementary Table 4. Genecoef of the modeled ARGs.

Gene	Coef
AFAP1L1	-0.010763683
BNIP3	-6.31E-05
CCND1	-0.000176182
EDA2R	-0.027715245
HAVCR2	-0.000701947
HMOX1	-0.000933778
IRF6	-0.016607677
KL	-0.003320283
LTB4R2	0.151859301
MNX1	0.107179555
NTRK2	-0.008127214
OCLN	-0.057952565
PLAUR	0.02023796
PLG	-0.015738536
PLK1	0.09541327
PTHLH	0.001435625
PYCARD	0.00076028
SERPINE1	6.21E-05
TIMP1	0.000182814
TLR3	-0.003016002
XAF1	0.020367715

Supplementary Table 5. Abbreviations and corresponding words and phrases used in this article.

Anoikis Index (AI)
anoikis-related genes (ARGs)
area under the ROC curve (AUC)
Benjamini-Hochberg (BH)
biological pathways (BP)
cellular components (CC)
Clear cell renal cell carcinoma (ccRCC)
Estimation of Stromal and Immune cells in Malignant Tumor tissues using Expression data (ESTIMATE)
extracellular matrix (ECM)
Gene Expression Omnibus (GEO; http://www.ncbi.nlm.nih.gov/geo/)
Gene Ontology (GO)
Gene set variation analysis (GSVA)
Kaplan-Meier (KM)
Kyoto Encyclopedia of Genes and Genomes (KEGG)
least absolute shrinkage and selection operator (LASSO)
matrix metalloproteinase (MMP)
molecular functions (MF)
Mutation Annotation Format (MAF)
non-negative matrix factorization (NMF)
overall survival (OS)
programmed cell death (PCD)
receiver operating characteristic (ROC)
Renal cell carcinoma (RCC)
The Cancer Genome Atlas (TCGA, https://www.cancer.gov/tcga)
tumor microenvironment (TME)
